# DNA methylation profiling deciphers three EMT subtypes with distinct prognoses and therapeutic vulnerabilities in breast cancer

**DOI:** 10.7150/jca.96096

**Published:** 2024-07-16

**Authors:** Shihao Sun, Shuang Chen, Nan Wang, Zehao Hong, Yi Sun, Yijia Xu, Jiangrui Chi, Xinxing Wang, Lin Li

**Affiliations:** 1 Department of Breast Surgery, the First Affiliated Hospital of Zhengzhou University, Zhengzhou, 450052, Henan, China.; 2 Center of Reproductive Medicine, the First Affiliated Hospital of Zhengzhou University, Zhengzhou, Henan 450052, China.; 3 Zhengzhou University, Henan 450052, China.

**Keywords:** breast cancer, methylation, epithelial-mesenchymal transition, machine learning, molecular subtype

## Abstract

**Background:** Epithelial-mesenchymal transition (EMT), deemed a pivotal hallmark of tumours, is intricately regulated by DNA methylation and encompasses multiple states along tumour progression. The potential mechanisms that drive the intrinsic heterogeneity of breast cancer (BC) via EMT transformation have not been identified, presenting a significant obstacle in clinical diagnosis and treatment.

**Methods:** A total of 7,602 patients have been included in this study. We leveraged integrated multiomics data (epigenomic, genomic, and transcriptomic data) to delineate the comprehensive landscape of EMT in BC. Subsequently, a subtyping classifier was developed through a machine learning framework proposed by us.

**Results:** We classified the BC samples into three methylation-driven EMT subtypes with distinct features, namely, C1 (the mammary duct development subtype with TP53 activation), C2 (the immune infiltration subtype with high TP53 mutation), and C3 (the ERBB2 amplification subtype with an unfavorable prognosis). Specifically, patients with the C1 subtype might respond to endocrine therapy or the p53-MDM2 antagonist nutlin-3. Patients with the C2 subtype might benefit from combined therapeutic regimens involving radiotherapy, PARP inhibitors, and immune checkpoint blockade therapy. Patients with the C3 subtype might benefit from anti-HER2 agents such as lapatinib. Notably, to increase the clinical applicability of the EMT subtypes, we devised a 96-gene panel-based classifier via a machine learning framework.

**Conclusions:** Our study identified three methylation-driven EMT subtypes with distinct prognoses and biological traits to capture heterogeneity in BC and provided a rationale for the use of this classification as a powerful tool for developing new strategies for clinical trials.

## Introduction

Breast cancer (BC) has emerged as the leading type of cancer among women and is characterized by high heterogeneity within malignant breast tissues. According to statistics published by the World Health Organization (WHO), nearly 2.3 million BC cases were diagnosed globally in 2020, and the death rate reached 15.5% in women^1^. The inherent heterogeneity of BC presents significant obstacles for targeted treatments, which range from surgery and chemotherapy to radiotherapy and emerging immunotherapies. BC heterogeneity frequently results in a spectrum of clinical complications, including BC recurrence, metastasis, and drug resistance.

Substantial research has confirmed that epithelial-mesenchymal transition (EMT) is a dynamic and continuous process marked by the transformation from an epithelial phenotype to a mesenchymal phenotype, with a continuum of phenotypes along the tumour progression^2^. Moreover, EMT plays a crucial role in facilitating the metastasis and recurrence of BC and is instrumental in preventing resistance to conventional treatments, including radiation, chemotherapy, endocrine therapy, and targeted regimens^3^, ^4^. EMT may contribute to the heterogeneity of BC. Notably, previous studies have focused on dissecting tumour heterogeneity based on the EMT process at the transcriptomic level^5^, with little attention to the influence of epigenetic reprogramming. As one type of epigenetic reprogramming, DNA methylation is crucial for regulating gene expression and maintaining chromatin structure. Abnormal DNA methylation, which has been identified at the onset of cancer, plays a key role in the metastasis and invasion of BC and several other malignancies^6^-^8^. Prior studies have shown that DNA methylation precisely modulates the plasticity of the EMT process^3^. Nonetheless, the regulatory mechanisms of EMT-related methylation in BC warrant further investigation. An analysis focused on EMT methylation-related features might offer considerable promise for the precise stratification of BC patients, potentially allowing further personalization of treatment strategies.

In the present study, based on 7,602 BC samples, we investigated EMT-related heterogeneity at the methylation level to refine the molecular classification of BC. The three novel EMT subtypes were characterized by distinct clinicopathological features, genomic driver events, biological functions, and immune landscapes. We devised a de novo computational framework for candidate drug identification. To accelerate the clinical application and translation of these findings, a machine learning (ML)-based classification system for identifying EMT subtypes was established. This study offers a paradigm for investigating the pathogenic mechanisms underlying BC and further pinpoints personalized strategies to specifically target vulnerabilities in BC.

## Materials and methods

### Cohort enrollment and data preprocessing

This study focused on analyzing a cohort of 7,602 BC patients with corresponding clinical information from multiple data repositories, including the Gene Expression Omnibus (GEO), the Molecular Taxonomy of Breast Cancer International Consortium (METABRIC), and The Cancer Genome Atlas (TCGA). RNA-seq, copy number, and mutation data from the TCGA were accessed through the National Cancer Genome Atlas (NCI) Genomic Data Commons (GDC) portal (https://portal.gdc.cancer.gov/), and clinical information was retrieved from the cBioPortal database (https://www.cbioportal.org). Survival information of BC patients was obtained from the TCGA Pan-Cancer Clinical Data Resource (TCGA-CDR). DNA methylation data were obtained via the University of California, Santa Cruz (UCSC) Xena Browser (https://gdc.xenahubs.net). In addition, seven BC datasets (GSE1456, GSE20685, GSE24450, GSE58644, GSE58812, GSE7390, and GSE96058) from the GEO database were utilized as validation sets. In this study, we included samples based on the following criteria: (a) diagnosis with histologically confirmed breast cancer; (b) status post-surgery; (c) availability of OS (overall survival) data. Additionally, samples were excluded if their transcriptome data were technical replications. For multi-omics data from the TCGA database, patients without matched transcriptome and methylation data were also removed. The distribution of features in the corresponding cohorts is described in **Table S1**. The detailed data processing procedure for these datasets is described in the **Supplementary Methods**.

### Methylation-driven EMT subtype classification via NMF

We integrated a total of 5023 EMT-associated genes (EMTAGs) from various sources, including the Molecular Signatures Database (MsigDB, http://www.broad.mit.edu/gsea/msigdb/), the EMTome (http://www.emtome.org/), and the dbEMT (http://dbemt.bioinfo-minzhao.org/) database (**Table S2**). Differential methylation analysis of tumour versus adjacent normal samples was performed utilizing the limma package. We considered genes with an absolute beta difference greater than 0.1 and an adjusted p-value less than 0.001 as DNA differentially methylated genes. A total of 339 candidate EMTAGs were identified by intersecting the aforementioned DNA differentially methylated genes with the EMTAGs (**Table S3**). To identify methylation-dependent EMT patterns, we performed non-negative matrix factorization (NMF) on 339 candidate EMTAGs via the nmf package (runs = 100, rank = 2-10; method = 'brunet')^9^. The optimal rank was estimated according to the cophenetic correlation coefficient, the dispersion value, and the combined score of them. Ultimately, we determined three as the optimal rank.

### Collection of previous classical subtypes of BC

To compare the associations between our EMT subtypes and previous classical subtypes, the TCGA-BRCA cohort and two other large cohorts (METABRIC and GSE96058) were reclassified in accordance with prior classification criteria; these included PAM50 subtypes, Breast Cancer Consensus Subtypes (BCCS), Topological Data Analysis (TDA) subtypes and canonical immune subtypes. PAM50 subtypes were identified via the genefu package, which assesses traditional intrinsic biological characteristics^10^. Mathews et al. employed the TDA approach to reclassify BC into 7 robust and interpretable PAM50 subtypes, named TDA subtypes^11^. The BCCS subtype classification system was developed by integrating six distinct unsupervised consensus-based clustering methods. Subsequently, six BC consensus subtypes were derived independent of the statistical methodology. In this study, BCCS subtyping was performed via the BCCSclassifier package. To investigate the BC immune microenvironment in our study, the six immune subtypes were analysed utilizing iAtlas (https://www.cri-iatlas.org/). To quantify the correlation between each EMT cluster and multiple molecular subtypes, we performed one-hot encoding of the EMT clusters and other molecular subtype levels and further calculated the Pearson correlation coefficients among these classification systems.

### Derivation of the EMT classifier

We conducted an EMT classifier via our proposed step-by-step ML framework. The detailed procedure for developing the ML-based classifier is described in the **Supplementary Methods.**

### Transition map of the EMT subtypes

To generate the transition map of EMT subtypes, we utilized the Potential of Heat-diffusion for Affinity-based Transition Embedding (PHATE) and the Uniform Manifold Approximation and Projection (UMAP) algorithm to project the data in a low-dimensional space. This process ensured intuitive visualization of EMT transcriptional programs in a 2D space.

### Molecular characterization of the EMT subtypes

Gene set variation analysis (GSVA) analysis was executed to quantify the activity scores of 85 EMT gene sets. For depicting the EMT status and characterizing the hybrid E/M phenotype (E: epithelial, M: mesenchymal), we computed the scores of three previously established EMT signatures (EMT76GS, EMTKS, and EMT score). The significance of enrichment was evaluated utilizing default parameters and the criteria was set as adjust p*-*value <0.05. In addition, to comprehensively assess tumor proliferation, we extracted the G0 arrested score, proliferation rate, and CA20 score from previous publications.

### Immune cell deconvolution and clustering

Kassandra algorithm was applied to quantify the tumour proportation and immune cell composition of each sample. DNA methylation beta values combined with signature gene data were subjected to CIBERSORT analysis for the deconvolution of immune cell populations via MethylCIBERSORT package. As depicted in one previous study, we classified BC samples into hot and cold immune phenotypes according to the immune cell populations obtained from MethylCIBERSORT analysis^12^.

### The tumour immune microenvironment of the EMT subtypes

Through utilizing multiomics data, the expression patterns of immunomodulatory genes among EMT subtypes were compared. We acquired various variables that have been previously linked to the tumour immune microenvironment (TIME), including the cytolytic activity (CYT) score^13^, IFN-γ signature score^14^, T-cell-inflamed activity (TCIA)^14^, extent of intratumoural heterogeneity (ITH) value, T-cell exhaustion signature score^15^, and T-cell receptor (TCR) diversity score^16^. Additionally, a comprehensive set of tumour neoantigen-related parameters was retrieved, encompassing the aneuploidy score, cancer testis antigen score, extent of homologous recombination deficiency (HRD), indel neoantigen, and tumour mutational burden (TMB)^17^. Immune cycle-related gene sets (Step 1: Release of cancer antigens; Step 2: Cancer antigen presentation; Step 3: Priming and activation; Step 4: Trafficking of immune cells to tumours; Step 5: Infiltration of immune cells into tumours; Step 6: Recognition of cancer cells by T cells; Step 7: Killing of cancer cells) were obtained from previous studies^18^, ^19^. The cancer immunity cycle program was measured through the single sample gene set enrichment analysis (ssGSEA) approach.

### Immunotherapy response prediction

Existing immunotherapy-treated cohorts with therapeutic response information were collected, comprising GSE91061 (melanoma)^20^, GSE165252 (esophageal cancer)^21^, GSE100797 (melanoma)^22^, GSE173839 (HER2-negative BC)^23^, GSE35640 (melanoma)^24^, and Tuba N Gide (melanoma)^25^. The Subnetwork Mappings in Alignment of Pathways (Submap) analysis was conducted to predict the immune response via GenePattern tools.

### Genomic alteration characteristics related to EMT subtypes

Detailed methods in this part are depicted in **Supplementary Methods.**

### Evaluation of drug sensitivity in the clinical cohort

To extend the study to the prediction of targeted therapies for each subtype, a computational pipeline was applied (**Fig. 6A**)^26^. Comprehensive pharmacogenomic datasets including CTRP and PRISM, aggregated comprehensive information on drug response and molecular profiles from human cancer cell lines, which enabled precise prediction of drug response in clinical samples^26^. Before conducting drug response prediction, the expression data of primary tumour samples were purified according to tumour purity estimates reported by Hoadley et al.^27^ to correct potential signal obscuration from stromal cells in the tumour microenvironment. This purification process was adopted via the MOFA package. As previously mentioned^26^, the pRRophetic package was employed to predict subtype-based sensitive drugs. In detail, the ridge regression model was trained on the mRNA expression profiles, and the drug response data of cancer cell lines with optimal predictive accuracy were evaluated via default 10-fold cross-validation. We applied the purified tumor expression profiles into this model to calculate drug response in clinical samples, ultimately identifying candidate-targeted drugs for each individual subtype. Apart from the abovementioned algorithm-based drug predictions, we also computed the radiosensitivity index (RSI) and endocrine therapy sensitivity scores in light of previous studies to corroborate the accuracy of our drug predictions^28^, ^29^.

### Delineation of the EMT landscape in the C1 subtype

Recognizing the dynamic transition of the EMT procedure, we executed dimensionality reduction based on graph learning to unveil the inherent structure and distribution of individual samples. Following reducing dimension and sorting, the EMT landscape was ultimately evolved. This entire process was conducted via the monocle package.

### Statistical analysis

Fisher's exact test was implemented for categorical variables, whereas Student's t-test, the Wilcoxon rank-sum test, the ANOVA test, and the Kruskal-Wallis test were conducted for continuous variables. Spearman correlation was used for comparison of continuous versus continuous variables. Survival analysis was performed by utilizing Kaplan‒Meier curves and log-rank tests. To correct for multiple tests, the p-values were adjusted to the false discovery rate (FDR) utilizing the Benjamini-Hochberg method where appropriate. Unless otherwise indicated, all the statistical tests were two-tailed. Significance levels are indicated by asterisks (*p <0.05; **p <0.01; ***p <0.001, ****p <0.0001). The statistical and bioinformatics analyses described above were carried out with R software (version 4.2.1).

## Results

### Unsupervised clustering identifies three distinct methylation-related EMT subtypes

**Figure 1:**
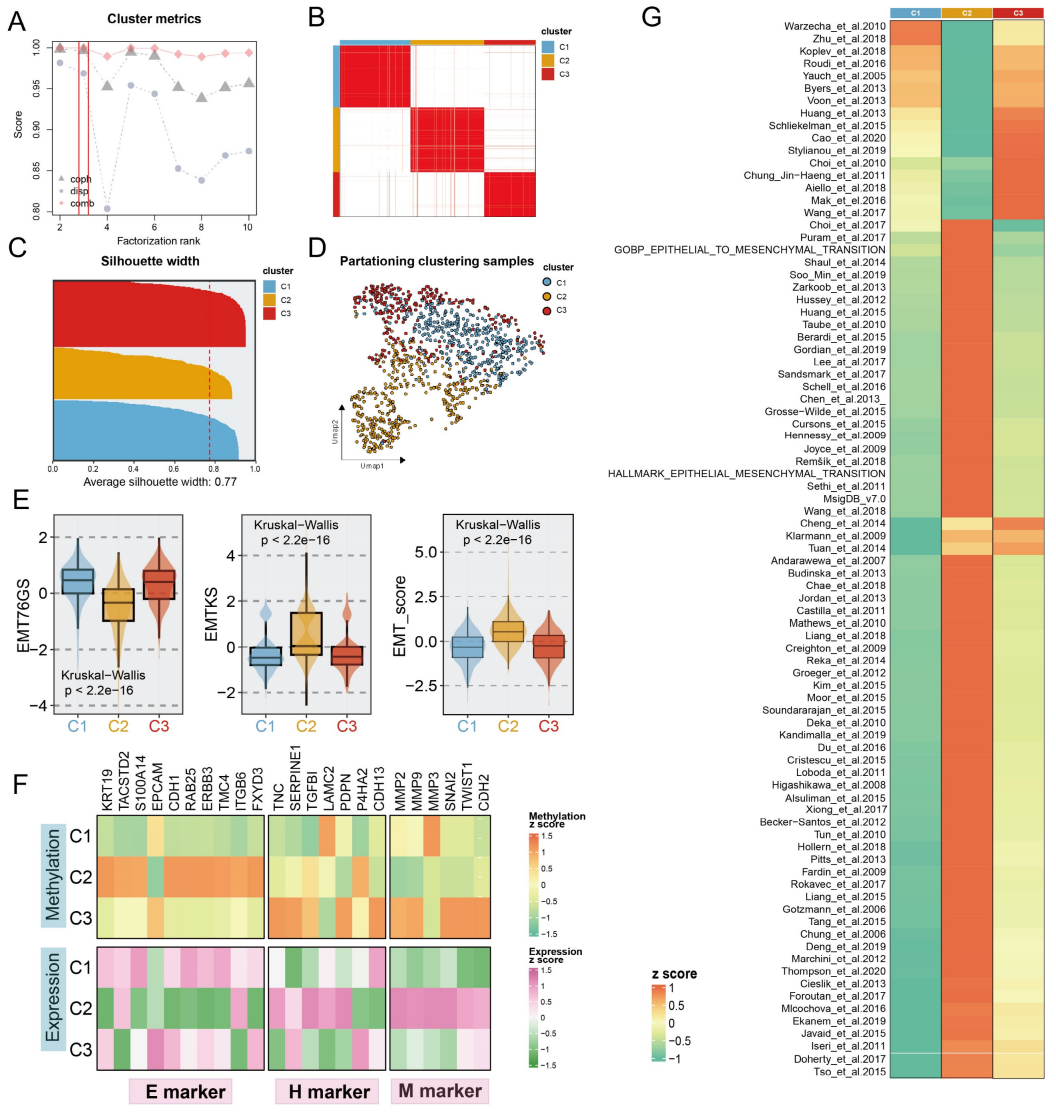
** Identification of distinct Methylation-EMT subtypes. A.** Multiple metrics to determine the optimal number of clusters presented by different shapes (cophenetic: grey dot, dispersion: purple dot). The optimal rank was chosen when the combination metric (red dot) occurred to steep drop off. **B.** Heatmap of consensus matrix with rank =3, mapping three subtypes labeled with different colors. **C.** The silhouette width of unsupervised clustering based on NMF method in methylation data when rank =3. **D.** UMAP plot of methylation expression profiles colored by distinct subtypes. **E.** EMT metrics of the samples in different subtypes.** F.** The normalized mean expression and DNA methylation levels of the 27 genes across EMT subtypes are indicated by the color gradient. E (epithelial) markers H(hybrid) markers, and M (mesenchymal) markers are displayed in the left, middle, and right panels, respectively. **G.** Heatmap of enrichment scores of EMT gene sets among subtypes.

To decode the EMT process in BC via a methylation-based analytical platform, we utilized the NMF algorithm to cluster BC into distinct subgroups based on the expression levels of BC-special EMTAGs (**Fig. 1B**; see **Supplement Methods**). According to dispersion, cophenetic coefficient, and combination of them, three methylation-based EMT subtypes with an average silhouette-width of 0.77 were identified and termed C1 (386), C2 (394), and C3 (275) (**Fig. 1A, 1C**). In addition, UMAP was conducted to project all the samples into a low-dimensional space, demonstrating significant discrimination (**Fig. 1D**). Unsupervised NMF clustering using the methylation profile revealed three EMT subgroups with strikingly different methylation patterns (**Fig. S1A**). Consistent with the previously reported continuity of the EMT process, we observed a potential EMT transformational spectrum from the methylation patterns of three subtypes, as evidenced by PHATE analysis (**Fig. S1B**). Subsequently, three EMT scoring metrics were employed to further quantify the EMT status for the subtypes. Compared to C1 and C3 tumours, C2 tumours were characterized by high EMTKS and high EMT scores but low EMT76GS scores (**Fig. 1E**). These findings revealed that the mesenchymal state was enriched mainly in the C2 subtype, whereas the C1 and C3 subtypes were more closely related to the epithelial state. GSVA further indicated that three distinct transcriptional programs were encoded by the different subtypes (**Fig. 1G**). DNA methylation participates in the regulation of EMT, thereby promoting tumour proliferation and metastasis. Thus, we compiled a list of epithelial, hybrid EMT, and mesenchymal markers from previous literature to explore EMT programmes in subtypes from multiomics (methylation and transcription) perspectives. At the transcriptome level, higher expression of H and M markers and lower expression of E markers were mainly observed in the C2 subtype. Notably, although no significant difference in quantitative EMT metrics was found between C1 and C3, it was found that C3, unlike C1, had increased H and M marker expression and rigorously regulated methylation, which may be related to the less favorable prognosis of the C3 subtype (**Fig. 1F, 2A**). In summary, these findings indicated that the progression of EMT programs was progressively transformed from C1 subtype (E states) to C3 subtype (H states), culminating in C2 subtype (M states).

**Figure 2:**
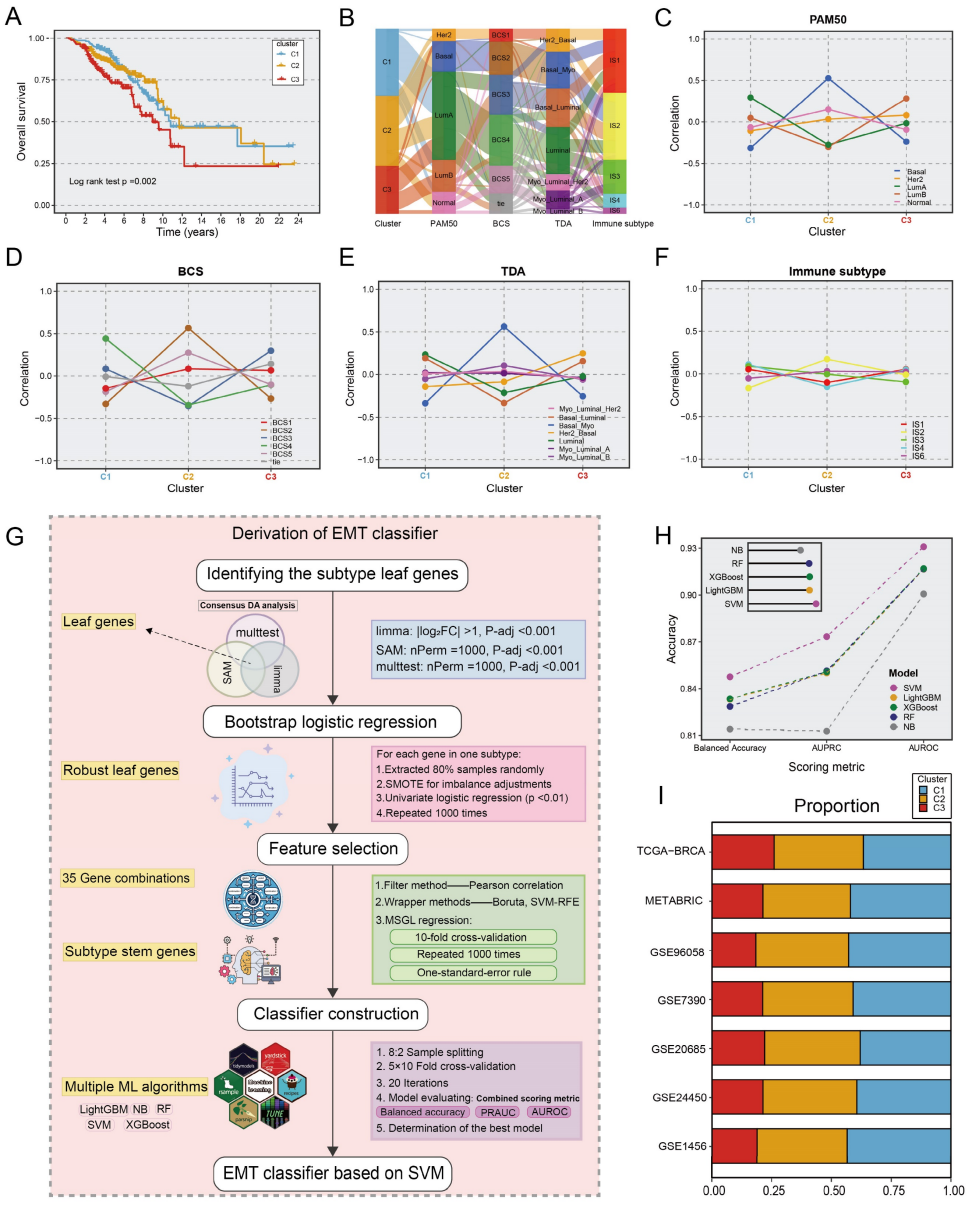
** The construction of EMT classifier. A.** Kaplan-Meier plot of overall survival among the three EMT subtypes in TCGA cohort. Log-rank test. **B.** Sankey plot showed the connection between EMT subtypes and other classical classifications, comprising PAM50, BCS, TDA, and immune subtypes.** C-F.** Correlation between EMT subtypes and other classical molecular clusters. Each panel indicated a molecular input to our EMT subtypes. **G.** The workflow of building an EMT classifier based on our ML framework. **H.** The scoring metrics for five classifiers evaluated the accuracy of the model, including balanced accuracy, AUPRC, and AUROC.** I.** Barplots showed comparable fractions of samples being assigned to each subtype in the training cohort and 6 validation datasets.

Survival analysis suggested that the C3 subtype had the worst overall survival (OS) prognostic outcome (p=0.002; **Fig. 2A**). In addition, we further explored the relevance of our subgroups to clinical features and canonical molecular subtypes. The C1 subtype was linked to luminal A-like BC, which included the luminal A, BSC4, and basal/luminal subtypes. The C2 subtype was associated with the Basal, BCS2, Basal/myoepithelial as well as IS2, which indicated stromal invasion, immune infiltration, and a poor prognosis. The C3 subtype was characterized by advanced tumour stage and older age, associated with the luminal B, BCS3, Basal/luminal, and HER2/ luminal subtypes, which denoted the worst prognosis (**Fig. 2B-F, Fig. S2**). Similar clinical characteristics among the three subtypes were also observed in two other large cohorts, the METABRIC and GSE90685 datasets (**Table S4**). In brief, the biological attributes exhibited by the other classifications were well captured into our three subtypes.

### ML architecture for deriving the EMT classifier

To identify EMT subtypes in a variety of cohorts and facilitate clinical translation, an ML-based classifier was developed, as described in **Figure 2G**. We first identify unbiased differential genes associated with the three subtypes utilizing consensus differential analysis (DA) strategy, which involves the intersection of genes obtained via three DA algorithms: Significance Analysis of Microarrays (SAM), resampling-based multiple hypothesis testing (multtest), and limma. Subsequently, we conducted quality control via logistic regression analysis with 1000 iterations, yielding 513 leaf genes (**Table S5**). The sequential feature selection framework (SFS) for ML was deployed to recognize subtype stem genes (SSGs). (1) Initially, a filtering procedure based on Pearson correlation coefficients was applied to eliminate collinearity. (2) Wrapper methods including boruta and support vector machine recursive feature elimination (SVM-RFE) were adopted to retain highly informative genes. (3) Multinomial sparse group-lasso (MSGL) regression analysis for running 1000 times with 10-fold cross-validation to identify the most robust gene panel (**Table S6**). Through the SFS framework, the 96-gene panel was deemed as SSGs and was inputted into multiple ML methodologies (**Table S7**). For the five ML algorithms used to predict EMT subtypes, multiple scoring metrics including balance accuracy, AUROC, and AUPRC were used for model evaluation to select the optimal classifier after 5×10 fold stratification and shuffled cross-validation via the 80% training and 20% test split. Ultimately, the SVM model exhibited remarkable performance in terms of model performance competition and was regarded as the most stable model for identifying EMT subtypes (Fig. 2H). When applying classifiers to gene expression data from different platforms, the ratios of subtypes were comparable across the TCGA-BRCA training cohort and six distinct validation datasets. As shown in **Figure 2I**, the ratio of subtypes showed similar trends across multiple cohorts (**Table S1**). Consistent with prior findings, patients with the C3 subtype had shorter OS (p <0.05 in six cohorts). In biological scenarios, subtype transformation and correlation analysis also showed consistent trends in two large cohorts, METABRIC and GSE96058 (**Fig. S3-S4**). Thus, the SVM-based classifier is a powerful tool for interpretation of EMT subtypes and has high clinical application value. To further augment the transformation and verification of the classifier in clinical scenarios, we conducted an R package entitled “BCEMTSclassifier”, which is accessible under the website https://github.com/LovelyMonkey123/BCEMTSclassifier.

### Comprehensive molecular characteristics related to EMT subtypes

**Figure 3:**
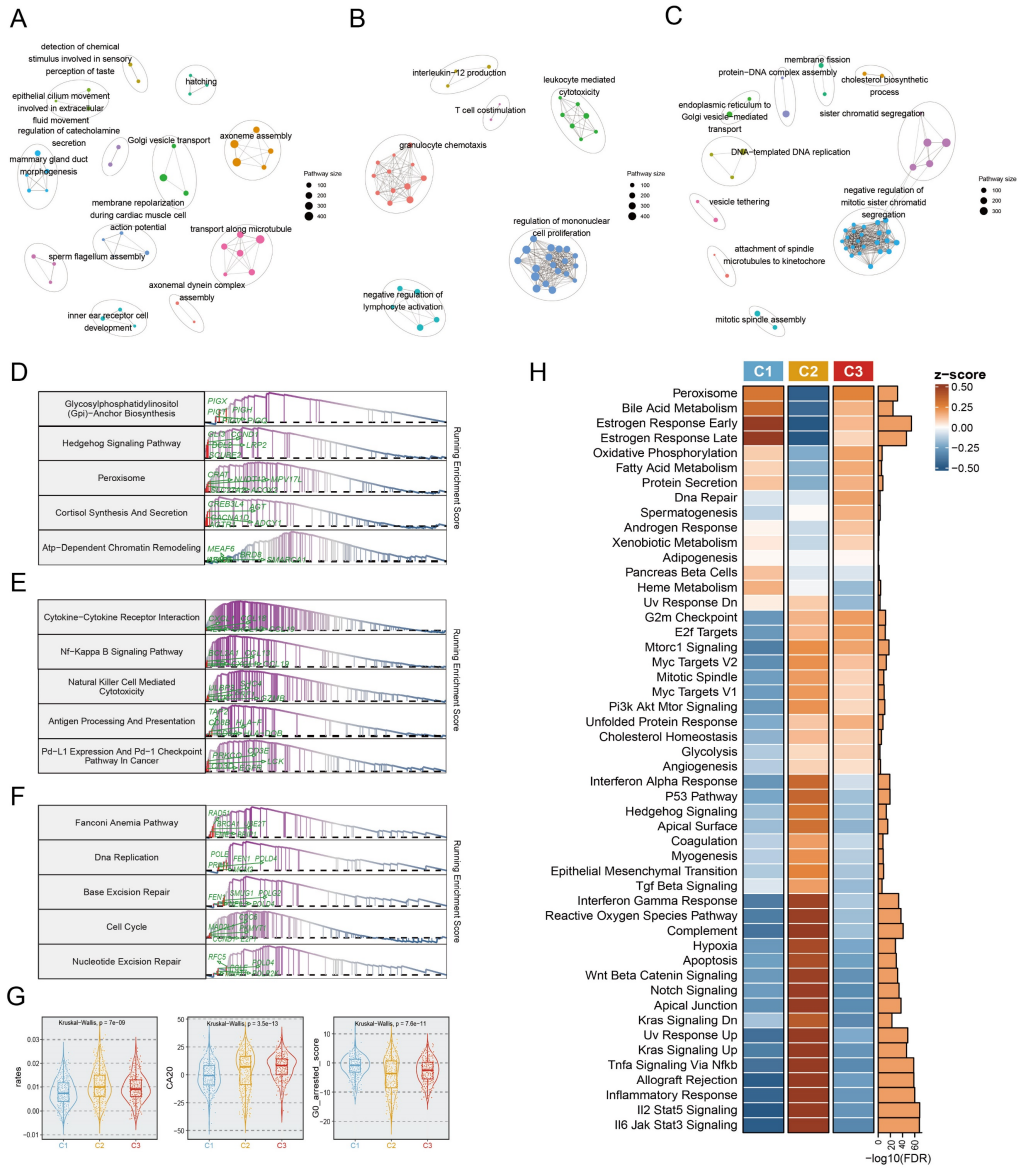
** Biological characterization of comprehensive characterization in EMT subtypes. A-C.** Enrichment map of statistically significant, nonredundant GO categories in C1 (A), C2 (B), and C3 (C), respectively. Nodes in the network represented pathways and were colored by associated subpopulations. Enrichment maps were generated via GSEA for the top 50 pathways with NES>0 and FDR <0.05 in each subtype.** D-F.** GSEA plots of the top 5 KEGG pathways in C1 (D), C2 (E), and C3 (F), respectively (FDR<0.05).** G.** Boxplot depicting the proliferation rates, CA20, and G0 arrested scores for each subtype.** H.** Heatmap of the normalized GSVA enrichment score about cancer hallmark pathways. The FDR was shown in the right barplot.

To further investigate the predominant molecular characteristics of the EMT subtypes, we employed Gene Set Enrichment Analysis (GSEA) for GO and KEGG entries, which yielded striking concurrence. In particular, the C1 subtype was linked to the enrichment of mammary epithelial development related pathways, encompassing mammary ductal morphogenesis, epithelial cilium motility, and Golgi vesicle transport (**Fig. 3A**). Notably, the proteasome pathway was prominently activated in the C1 subtype (**Fig. 3D**). The C2 subtype was enriched in immunologic pathways, primarily involving T cell co-stimulation, immune cell-mediated cytotoxicity, immune cell activation, and antigen processing and presentation (**Fig. 3B, 3E**). Importantly, the C3 subtype was characterized by upregulation of DNA replication and DNA‒protein complex assembly (**Fig. 3C**), the fanconi anemia pathway, and excision repair (**Fig. 3F**). Significant differences in the dominant features of the three subtypes were further revealed and proven through oncogenic hallmark pathway analysis (**Fig. 3H**). Tumor proliferation, a typical indication of tumorigenesis, was dramatically upregulated in the C2 and C3 subtypes (**Fig. 3H**). Moreover, centrosome amplification (CA), cell cycle arrest, and the cell proliferation rate are regarded to have immeasurable impacts on tumour proliferation. Based on this, we found that both C2 and C3 tumours showed higher proliferation rates, and CA20 scores, but lower G0 arrested scores (Fig. 3G).

We also explored in more detail the relationships between multiple states of EMT programs (C1, C2, and C3) and metastatic potential. Four metastasis-related gene sets were retrieved from previous research^30^-^33^, aiming to assess the metastatic potential of BC. ssGSEA analysis of these gene sets revealed that the activity scores of metastatic gene sets exhibited marked upregulation in the C2 subtype compared to C1 and C3 (**Figure. S5A-E**). In addition, the metastatic potential of BC was further evaluated using a risk score system encompassing nine metastasis-related genes, which was conducted by Xiao et al^34^. Here we found that patients in the C2 subtype showed a significantly higher risk of metastasis as indicated by their higher proportion in the high-risk group compared to patients in other subtypes (**Fig. S5F**).

### Immune states and therapy for the EMT subtypes

**Figure 4:**
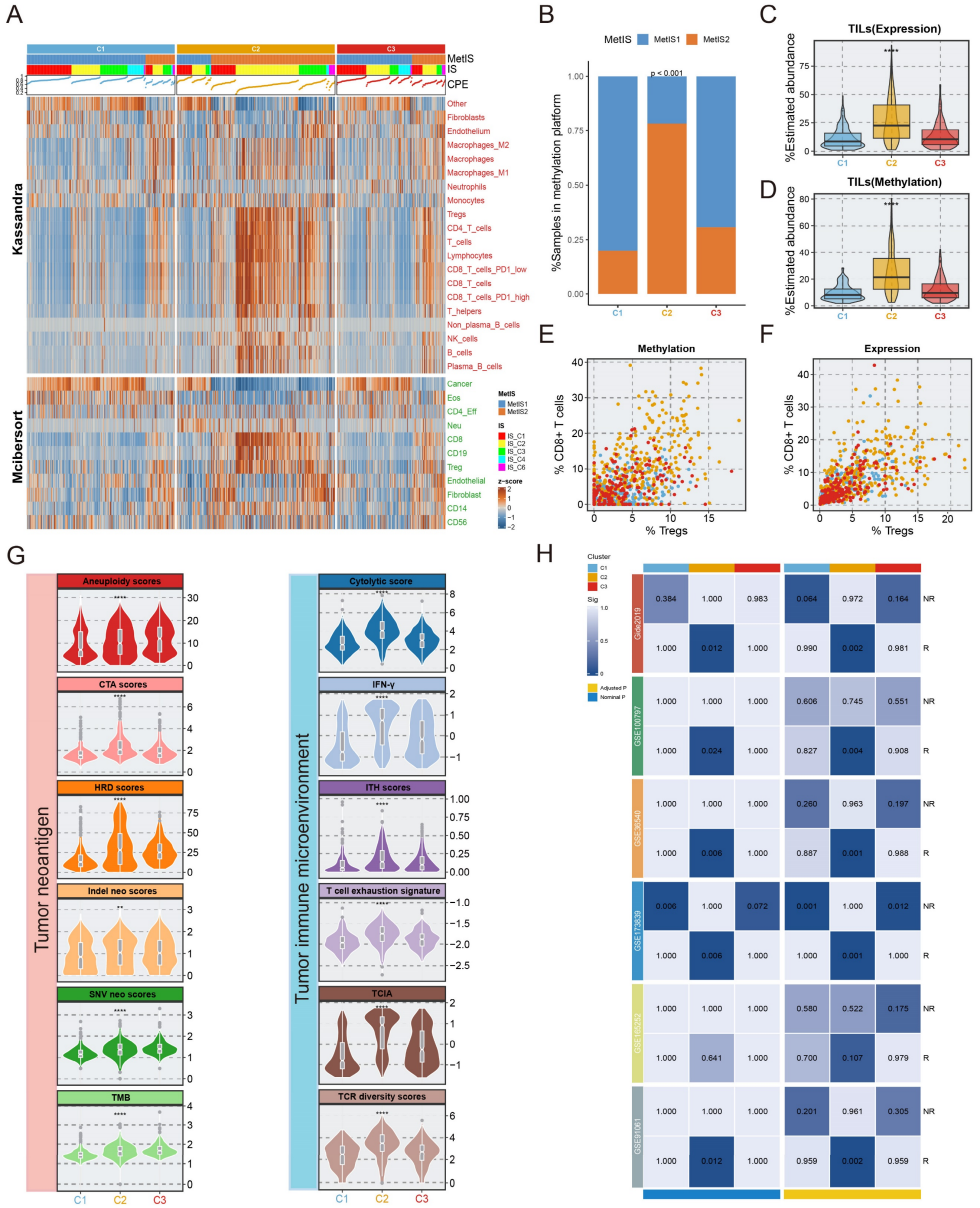
** Characterization of the immune environment in three subtypes.** The immune characteristics and therapy prediction of three subtypes. **A.** The correlation between the EMT subtypes and immune infiltrating cells from multi-omics views. **B.** The difference between MetIS subtypes and our EMT subtypes. **C-D.** The relative abundance of TILs was estimated in three subtypes via transcriptome, methylation deconvolution algorithm.** E-F.** Scatter plots depicting the CD8 T cell/Treg ratio of the EMT subtypes. **G.** Boxplot showing the difference of twelve factors of two distinct classes linked to tumor neoantigens and tumor microenvironment in three subtypes.** H.** Submap analysis was used to explore the response of immunotherapy in three subtypes.

Given that the EMT subtyping was independent of methylation platforms (**Fig. S6**), immune microenvironmental analyses were performed only with samples retrieved from the 450k methylation sequencing platform. For the precise reconstruction of TIME, we implemented the deconvolution algorithms including Kassandra and MethylCIBERSORT utilizing transcriptome and methylation data, respectively. Combining PAM clustering with the composition of immune cells determined by MethyCIBERSORT, the tumours were stratified into two subtypes, immunologically cold (MetIS1) and hot (MetIS2) (**Fig. S7**). As presented in **Figure 4A** and **4C-D**, C2 tumours exhibited greater tumour-infiltrating lymphocytes (TILs) and an increased percentage of purity-estimated consensus measurements (CPE). In addition, C2 tumours had a higher percentage of MetIS2 and IS2 subtypes than other EMT subtypes (**Fig. 4A-4B**). A greater ratio of CD8 T cells to Tregs, a favourable marker for immune-thermal tumours and immunotherapy, was observed in C2 versus other EMT subtypes (**Fig. 4E-4F**).

In addition, twelve variables from 2 different categories related to (1) tumour neoantigens and (2) the TIME have been used to predict immunotherapy efficacy in a variety of tumours. We compared these factors among the EMT subtypes, and the results revealed that nearly all metrics were higher in the C2 subtype, the exceptions were TMB, SNV neo score, and aneuploidy score, which increased in rank from C1 to C3 (**Fig. 4G**). Moreover, representative steps of the cancer immune cycle were employed, including antigen release, induction and activation, recruitment and infiltration of immune cells, cancer cell recognition, and cancer cell killing. The results showed that scores of these steps were significantly elevated in C2 compared with C1 and C3 (p < 0.05 in all seven steps, **Fig. S8**).

In clinical oncology, immunomodulators are potential agonists and antagonists for tumor immunotherapy. We integrated multiomics data to compare the expression and regulatory roles of immunomodulators in EMT subtypes, revealing that C2 had the highest expression of immunomodulators (**Fig. S9**). Submap analysis also showed that the C2 subtype exhibited gene expression patterns similar to immunoreactive subtypes in six discrete immunotherapy cohorts (**Fig. 4H**). In conclusion, C2 patients may derive clinical benefit from immunotherapy.

### Mutational landscape of EMT subtypes

**Figure 5:**
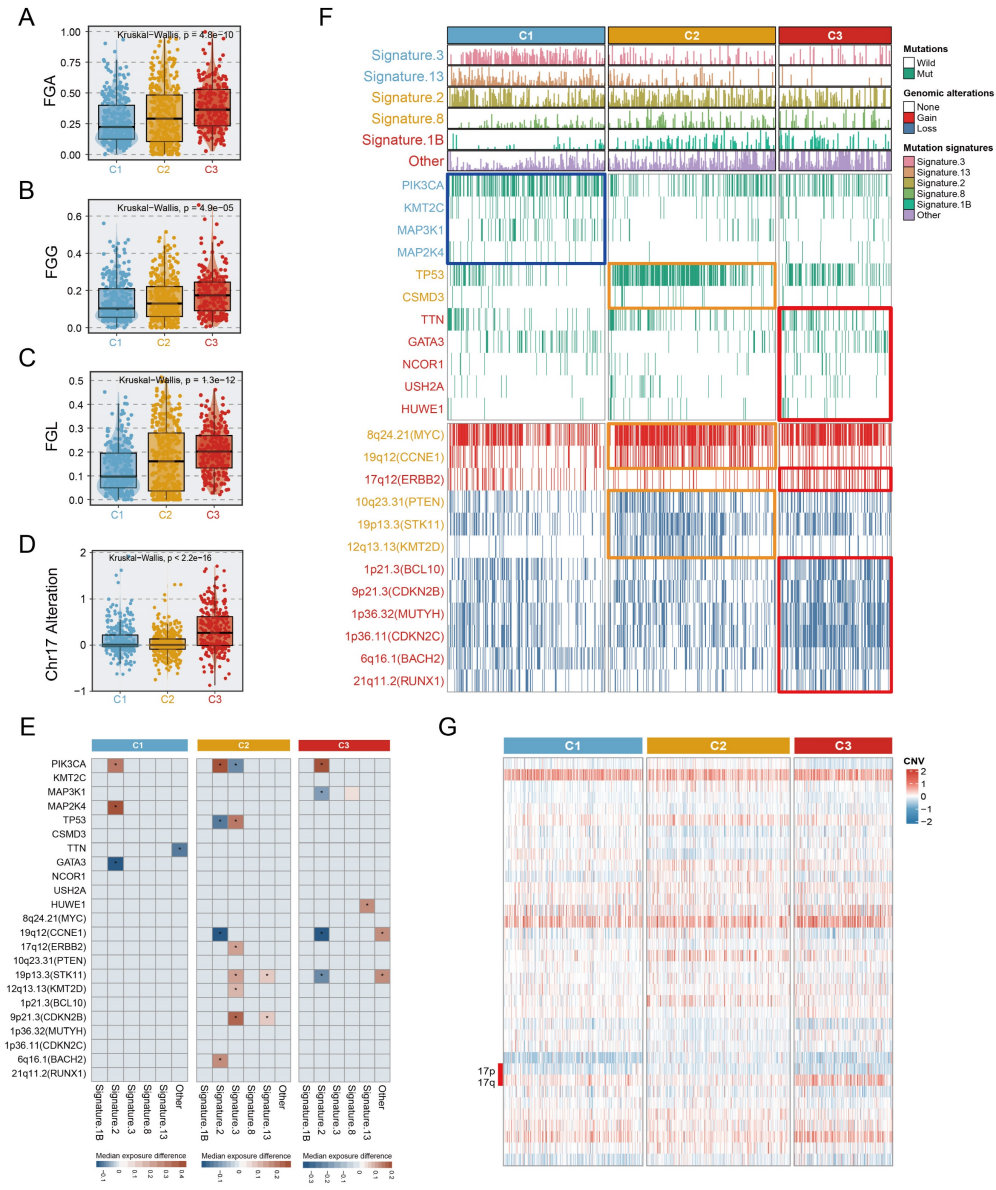
** Multi-omics alteration characteristics of three subtypes. A-C.** The fraction of genome alteration (FGA), the fraction of genome gained (FGG), and the fraction of genome lost (FGL) in three subtypes. **D.** The differential distribution of chr17 alteration in three subtypes. **E.** Heatmaps depicting the increase (value >0) or decrease (value <0) in mutational signature prevalence in samples harbouring gene mutations or copy number changes. The color gradient indicates the median change in exposure compared to wild type. Only significant changes of >10% (in either direction) were retained with Wilcoxon rank-sum two-sided test p-value <0.05, which was marked using stars. **F.** Genomic alteration landscape in three subtypes. Subtype-enriched events (mutation or copy number alteration) were labeled the color of the corresponding subtype. Fisher's exact test.** G.** The global copy number alteration landscape of three subtypes was shown via heatmap.

We investigated the mutation and copy number landscapes of the three subtypes to discern subtype-specific genomic events. Differences in chromosome abundance and content were observed in the three subtypes (**Fig. S10**). Chromosomal instability, characterized by perturbations in the fraction of genome altered (FGA), the fraction of genome gained (FGG), and the fraction of genome lost (FGL), exhibited significant variances across the three subtypes, with notable upregulation in C3 (**Fig. 5A-C**). Additionally, we noted that extensive alterations in chromosome 17 (ERBB2 amplification), were distinctive to the C3 (**Fig. 5D, 5G**). In terms of mutation signatures, C1 was enriched mainly in the BRCA1/2 mutation-related (Signature.3) and APOBEC-related (Signature.13) signatures, C2 was characterized by APOBEC-related signature (Signature.2) and Signature.8, and C3 was enriched predominantly in the age-related signature (Signature.1B). PIK3CA, a hotspot mutation in BC, occurred in C1 at a high frequency (50.6%, p <0.001), representing a potential clinical benefit of PIK3 inhibitors. Other activating mutations with >10% change, such as KMT2C (13%, p =0.004) and MAP3K1 (16%, p < 0.001) were also involved in C1. We observed the elevation of TP53 mutations in C2 (61%, p <0.001), which was associated with high immunogenic characteristics^35^. Somatic GATA3 mutations were found prominently in C3 (21%, p <0.001) and C1 (19%), consistent with the epithelial characteristics of these subtypes^36^. Integrated analysis, leveraging the OncoKB database, which contains information on cancer-driving genes and a computational pipeline, was performed in this study to identify cis-driver cytoband segments (**Supplementary Methods**, **Table S9**). Specifically, C2 was significantly associated with frequent amplification of CCNE1 (40.9%, p <0.001), while ERBB2 amplification was predominantly present in C3 (31.4%), and significantly associated with this subtype (p <0.001). Despite the fact that a high prevalence of MYC amplification (reaching 72.7%) was observed in C2, widespread MYC amplification patterns of >50% also occurred across all three subtypes. Further, we realized that not only copy number amplification, but also copy number deletion events were more common in C2 and C3, compared to C1; the C3 subtype was predominantly linked to the deletion of genes involved in tumour proliferation and DNA repair processes^37^-^39^, encompassing BCL10, CDKN2B, MUTYH, CDKN2C, BACH2, and RUNX1 (**Fig. 5F**). Genomic alterations affecting several events were linked to the age-related (Signature.1B), APOBEC-related (Signature.2/13), BRCA1/2 mutations (Signature.3), and other signatures (**Fig. 5E**). Surprisingly, although the phenotypic age distributions of the three subtypes differed significantly, fluctuations in Signature.1B among subtypes were not interfered with subtype-specific driver events, implying that age-associated Signature.1B persisted independently of other mutational features. Most notably, the incidence of Signature.2 indicated subtle and quite distinct associations in subtypes. For example, the PIK3CA mutation was strongly associated with an elevated incidence of Signature.2 in three subtypes, whereas in C1, the MAP2K4 mutation and wildtype GATA3 were linked with an increase of Signature.2. Intriguingly, the opposite trend was uncovered about the associations of PIK3CA and TP53 with Signature.2, suggesting that distinct mutation events mediate consistent pathways. In both C2 and C3, CCNE1 alterations showed a consistent correlation with Signature.2 (**Fig. 5E**).

### EMT dynamic landscape of BC via pseudo-temporal analysis

To reveal the underlying structures of the distribution of individual patients, the EMT trajectory was segmented, and key EMT subprogrammes relying on our three subtypes were revealed. Actually, the trajectories with descriptive states unveiled the dynamics of EMT programs. The epithelial-like C1 and C3 subtypes were positioned at the beginning of the pseudotime trajectory. Surprisingly, due to the heterogeneity of basal-like BC, the C2 subtype was placed at the terminal end of two distinct EMT-related branches (**Fig. S10A**). Although the C1 subgroup had the favourable prognosis, we observed that the samples of the C1 subtype were distributed in multiple branches of the EMT trajectory, which showed significant intro-subtype heterogeneity within C1 (**Fig. S11B**). C1 was further divided into three subtypes according to the location of the samples within the EMT trajectory (C1a-c), which showed apparent differences in EMT transcriptional status and methylation expression patterns (**Fig. S11C-F**). Survival analysis showed that there was poorer clinical outcome occurred in C1b, comparing to C1a and C1c (log-rank, p = 0.038; **Fig. S11G**). Overall, these findings indicated that our dynamic landscape analysis provided complementary benefits for further deciphering EMT programmes.

### Identification of subset-specific therapeutic agents in EMT subtypes

**Figure 6:**
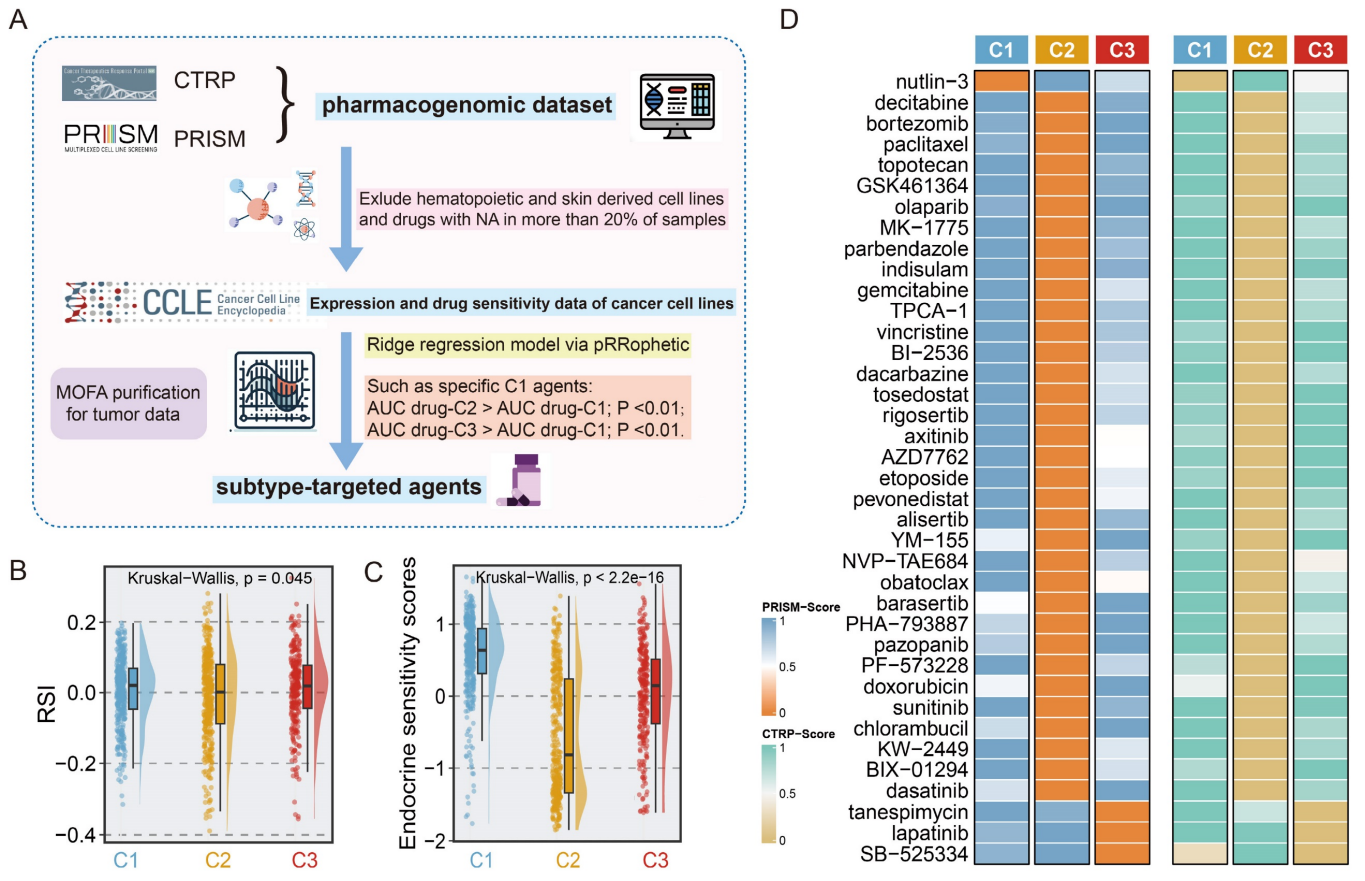
** Identification of subset-specific therapeutic agents in EMT subtypes. A.** Scheme of computational pipeline for identifying therapeutic agents. **B-C.** Boxplot comparing the RSI (**B**), and endocrine sensitivity score (**C**) in three subtypes. Kruskal-Wallis test. **D.** Candidate agents for three subtypes were depicted through heatmap. PRISM-Score and CTRP-Score represent the normalized drug sensitivity score via min-max transformation.

To determine the potential vulnerabilities of and therapeutic options for patients with different EMT subtypes, we harnessed a computational framework to identify potential targeted drugs for each subtype by leveraging pharmacogenomic datasets including CTRP and PRISM (**Fig. 6A**). Given the potential impact of confounding signals originating from the tumour microenvironment on drug therapy, the MOFA technique was utilized to correct tumour purity, yielding normalized tumour expression profiles as the input for the next drug prediction. Employing drug prediction pipeline, 38 subtype-specific targeted agents were identified (**Fig. 6B**, **Table S10**). Notably, the candidate agents showed significant alignment with the genomic and molecular vulnerabilities specific to the corresponding subtypes. For example, nutlin-3, a C1-targeted agent, was engineered to target the TP53 wild-type, a feature that was abundant in C1. Meanwhile, C2 was characterized by extensive activation of oncogenic pathways, suggesting a broader range of therapeutic possibilities, as shown in Figure 6B. In line with the strikingly elevated expression of ERBB2 in C3 (**Fig. S12H**), lapatinib was identified as a potential targeted agent for C3.

Next, we gathered information on several treatment response-associated signatures to facilitate precise subtype-based treatment in clinical scenarios. Unexpectedly, the highest endocrine sensitivity score was observed in C1 and corresponded to elevated luminal-related gene expression in this subtype (**Fig. 6D**, **Fig. S12A-G**). C2 had the lowest RSI, which indicated that radiotherapy is a promising clinical option for patients with this subtype (**Fig. 6C**). Altogether, these results delineated that for patients with distinct EMT subtypes, a more precise and tailored target strategy is imperative.

## Discussion

**Figure 7:**
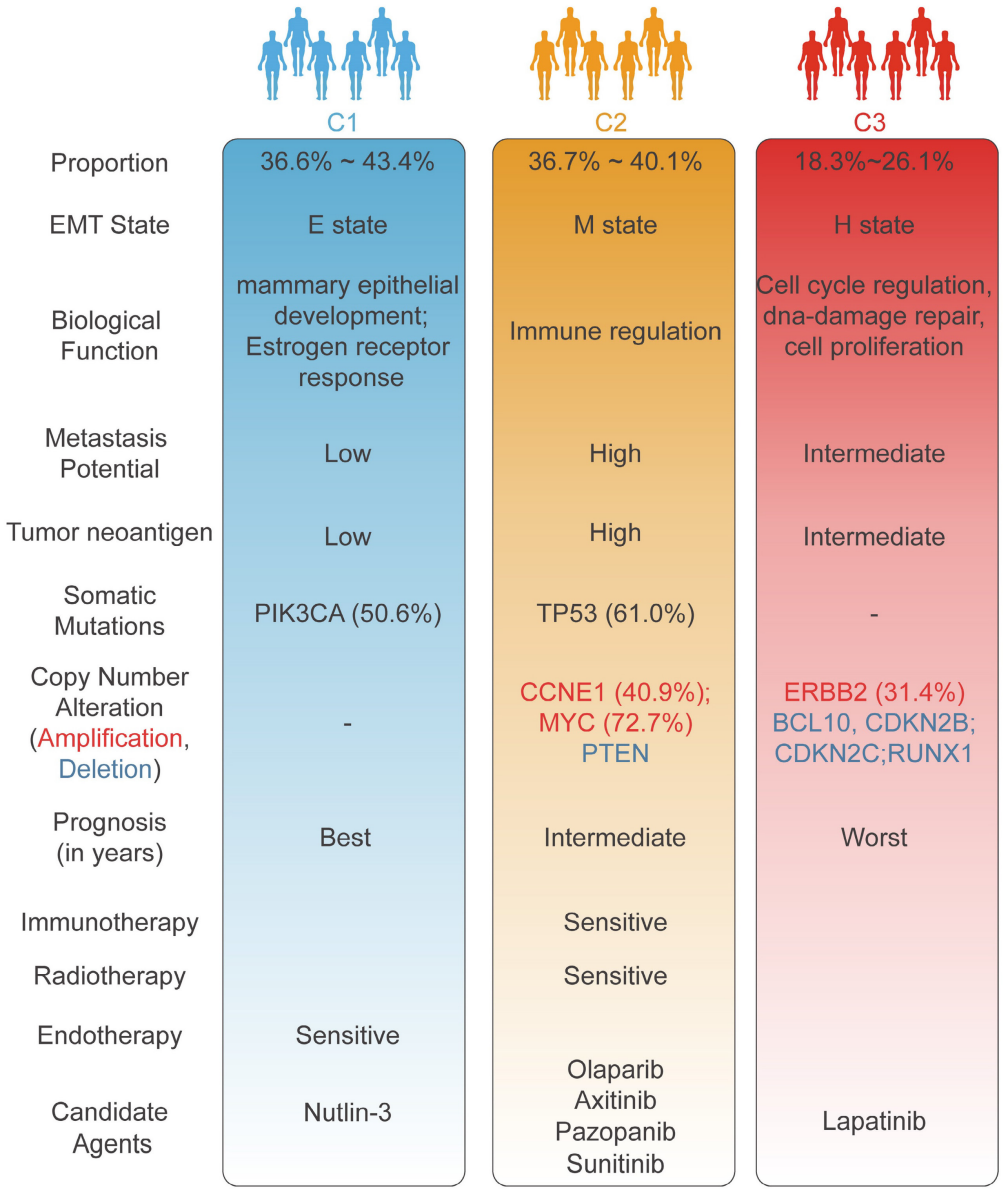
The overview of different characteristics unique to individual EMT subtype.

BC is a clinically heterogeneous disease. An in-depth comprehension of epigenetic heterogeneity in BC could greatly improve the stratification of populations and reveal opportunities for precision therapies. In contrast to prior efforts to unravel the complexity of EMT in multiple tumours at the transcriptome level^40^, ^41^, our study delineated EMT subtypes from a methylation standpoint to decode the intrinsic heterogeneity of BC. Multiomics data were employed to assess the clinicopathological features and molecular mechanisms underlying the EMT subtypes. Integrated EMT-based analysis clearly classified BC samples into three subtypes with distinct clinical characteristics, biological phenotypes, genomic variants, and immune landscapes. Pseudo-temporal analysis was employed to further dissect the intra-cluster heterogeneity of EMT subtypes. Large-scale drug identification frameworks were utilized to identify potential targeted drugs that may be effective in treating specific subtypes. In addition, based on the ML pipeline proposed in our study, the EMT classifier was developed to enhance clinical utility and feasibility. Comprehensive molecular and biological features unique to each EMT subtype identified through our analysis are shown in **Figure 7**.

The C1 (mammary duct development) subtype was distinguished by a high PIK3CA mutation frequency, TP53 activation, and a favorable prognosis, aligning with the characteristics of the luminal A, BSC4, and basal/luminal classifications. In response to previous studies, our study also demonstrated that mammary gland duct development and cancer progression are modulated mainly by ER alpha-mediated increases in estrogen levels^42^. Accumulating research has unveiled that PIK3CA mutations predominantly occur in ER+ BC with a favorable prognosis^43^, ^44^; these features are consistent with the features identified in C1. Overexpression of luminal-related genes and elevated endocrine sensitivity scores indicate sensitivity to endocrine therapy for the C1 subtype in this study^29^, ^45^, ^46^. The low proliferative activity of C1 implied that commonly utilized chemotherapy drugs, such as paclitaxel which primarily acts on the proliferation pathway in BC, might encounter limitations in their effectiveness. With the goal of further improving the clinical outcomes of C1, we employed a ridge regression model and identified a potential therapeutic drug for C1: nutlin-3. TP53 is activated in the C1 subtype, and the efficacy of nutlin-3 in treating TP53 wild-type BC was confirmed in earlier studies^47^. More importantly, a subgroup of patients in the C1 subtype (C1b) exhibited an unfavorable prognosis than other subclusters (C1a and C1b) through EMT dynamic landscape analysis, suggesting some degree of intro-subtype heterogeneity.

The C2 (immune infiltration) subtype, was recognized by a high prevalence of TP53 mutation, extensive genomic alterations, and markedly increased metastatic potential, and was linked to the Basal, BCS2, basal/myoepithelial, and IS2 (interferon-γ subtype) classifications from previous literature. C2 exhibited co-inactivation of key tumour suppressor genes, particularly TP53 and PTEN, which induced widespread activation of various oncogenic pathways, notably the PI3K/AKT/mTOR and P53 pathways^48^-^50^. We also observed that CCNE1 amplification drove the promotion of proliferative pathways and contributed to an enhanced mesenchymal phenotype in C2^49^, ^51^. Additionally, the amplification of CCNE1 and MYC indicated worse clinical outcomes in metastatic tumours^52^. Intriguingly, the loss of function of KMT2D, a histone methyltransferase, mediates genomic damage, thereby accelerating cancer progression^53^. In line with this, the TIME was remodeled to upregulate tumour immunogenicity^53^, emerging the proficient immune response of the C2 subtype. Our research demonstrated that tumours with high HRD scores and low RSI scores tended to be C2 tumours, which suggested that the combination regimens of PARP inhibitors with radiotherapy and immunotherapy may conquer clinical obstacles 23. In this context, olaparib was identified as an outstanding regimen in C2 patients. Stromal-enriched tumours indicate elevated vascular infiltration in C2, consistent with the targeted effects of various angiogenesis inhibitors including axitinib, pazopanib, and sunitinib.

The C3 (ERBB2 amplification) subtype, defined by obvious ERBB2 amplification, was predominantly associated with the conventional luminal B, BCS3, basal/luminal, and HER2/luminal classifications. GATA3-inactivating mutations are known to promote metastatic invasion in BC^36^, ^54^, a phenomenon reflecting the attributes of the C3 subtype, which is characterized by advanced clinicopathological grade and early metastasis. The elevated frequency of deletions of multiple tumour suppressor genes contributed to the high DNA repair capacity and cell proliferation activity of C3, further enhancing malignant tumour progression. We utilized our computational pipeline to pinpoint ERBB2 (also known as HER2 or neu) amplification as the most significant genomic event in C3, and our results also indicated that the mRNA expression of ERBB2 was markedly upregulated in C3 than C1 and C2. Correspondingly, our drug identification pipeline suggested lapatinib, a dual-targeted HER receptor tyrosine kinase inhibitor^55^, as a personalized treatment option for C3 samples. To expand the therapeutic options for patients in the C3 subtype, tanespimycin was also identified as a second-line treatment regimen.

Beyond subtype heterogeneity, our study revealed an inherent phenotypic continuum among EMT subtypes, wherein a transition occurs from the C1 (relative epithelial state) to C3 (relative hybrid state), followed by the C2 (relative mesenchymal state) subtype, as evidenced by PHATE analysis. Our findings indicated that during this dynamic transformation, the EMT program was increasingly activated with the disturbance of genomic alterations and the enrichment of immune infiltration, concurrently with a gradual decline in tumor purity. This evolutionary pattern was critical in tumor progression as it enhanced metastatic potential and modulated immune components. The EMT process was closely interactive with the immune microenvironment within the tumour. The C2 subtype had high immune cell infiltration and adequate enhancement of anti-tumour immune program, as evidenced by the significantly elevated immune-related pathways. These observations suggested that heightened EMT activation might foster immune infiltration and augment response to immunotherapy. As previously discussed, the genomic alterations in the three subtypes were associated with their unique therapeutic vulnerabilities. However, the alteration patterns of FGA, FGG, and FGL did not consistently align with the transformation pattern of the EMT process across the three subtypes. Conversely, we observed that the percentage of genomic alterations followed a similar trend of deteriorating clinical outcomes, progressing from C1 through C2, culminating in the highest accumulation in C3. C3, representing an intermediate EMT state, is characterized by the poorest prognosis and the richest genomic alterations. In summary, the EMT classification system was confirmed as a powerful tool for dissecting the heterogeneity and dynamic nature of the EMT process.

Our research delineated an EMT classification framework for BC, which precisely captured the molecular and clinicopathological characteristics in each individual subtype. This framework could facilitate the development of personalized treatment strategies tailored to each EMT subtype. Based on our proposed ML pipeline, a 96-gene-based EMT classifier was developed, aiming to streamline the clinical implementation of the EMT classifying system in real-world scenarios. This study paves the way for unraveling the complexities of EMT heterogeneity in BC and lays the groundwork for precision in both tumour classification and treatment. While our classifying system holds promise, it is hindered by the absence of real-world validation. To address these challenges, the R package, BCEMTSclassifier was conducted. More importantly, we have designed a forthcoming clinical study aimed at refining the validation of EMT subtype classification at the single-cell level, with the goal of elucidating underlying molecular mechanisms and ultimately facilitating clinical translation in the future.

## Supplementary Material

Supplementary methods, figures and tables.

## Abbreviations

BCCS: Breast Cancer Consensus Subtypes
BC: Breast cancer
CYT: cytolytic activity
EMTAGs: EMT-associated genes
EMT: epithelial-mesenchymal transition
FDR: false discovery rate
GEO: Gene Expression Omnibus
GSVA: gene set variation analysis
GDC: Genomic Data Commons
HRD: homologous recombination deficiency
ITH: intratumoral heterogeneity
MsigDB: Molecular Signatures Database
METABRIC: Molecular Taxonomy of Breast Cancer International Consortium
MSGL: Multinomial sparse group-lasso
NMF: Non-negative matrix factorization
PHATE: Potential of Heat-diffusion for Affinity-based Transition Embedding
CPE: purity-estimated consensus measurements
OS: overall survival
RSI: radiosensitivity index
multtest: resampling-based multiple hypothesis testing
SAM: Significance Analysis of Microarrays
ssGSEA: single sample gene set enrichment analysis
Submap: Subnetwork Mappings in Alignment of Pathways
SVM-RFE: support vector machine recursive feature elimination
TCIA: T cell-inflamed activity
TCR: T cell receptor
TCGA-CDR: TCGA Pan-Cancer Clinical Data Resource
FGA: The fraction of genome alteration
FGG: the fraction of genome gained
FGL: the fraction of genome lost
TDA: Topological data analysis
TILs: tumor-infiltrating lymphocytes
TIME: tumor immune microenvironment
TMB: tumor mutational burden
UMAP: Uniform Manifold Approximation and Projection
University of California, Santa Cruz: UCSC
WHO: World Health Organization
