# Supplementary material for: DNA methylation profiling deciphers three EMT subtypes with distinct prognoses and therapeutic vulnerabilities in breast cancer
Source: J Cancer. 2024 Jul 16;15(15):4922–38. doi: 10.7150/jca.96096 (PMC11310866; doi:10.7150/jca.96096)
Supplement: Supplementary file 1 — Supplementary methods, figures and tables. [file jcav15p4922s1.zip › Supplementary Figures.pdf]

Supplement Figures

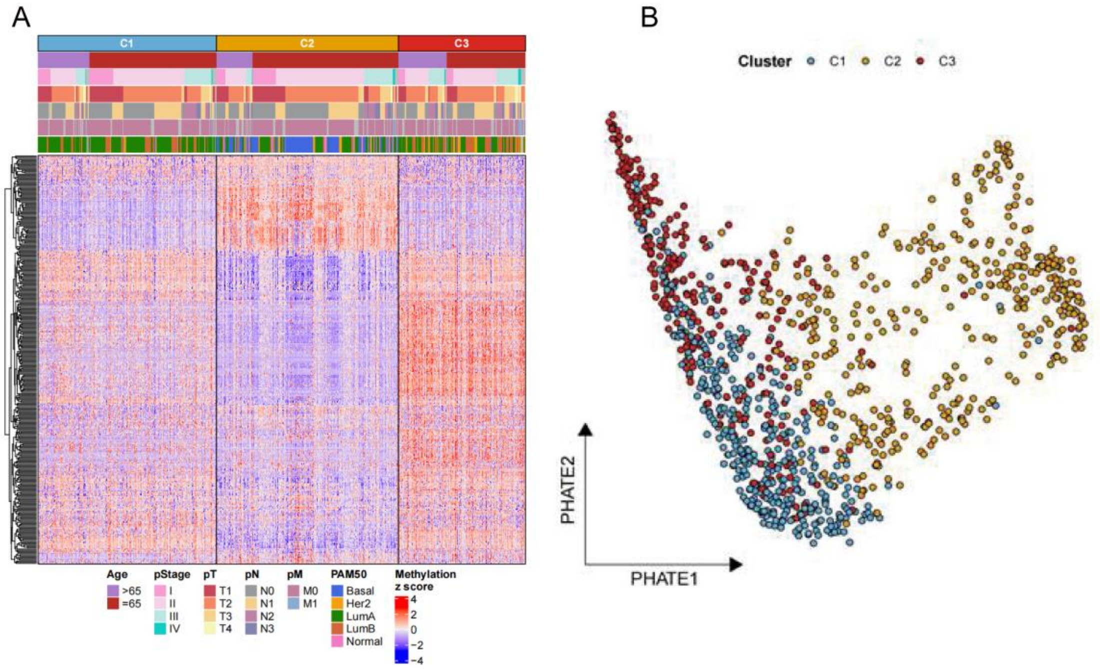

Fig. S1: The DNA methylation profiling and transitional spectrum of EMT subtypes. (A) Heatmap showing the unsupervised NMF clustering of the BC samples using DNA methylation profiling. (B) PHATE plot illustrating the transitional spectrum of EMT subtypes. Each sample is colored according to the EMT subtype.

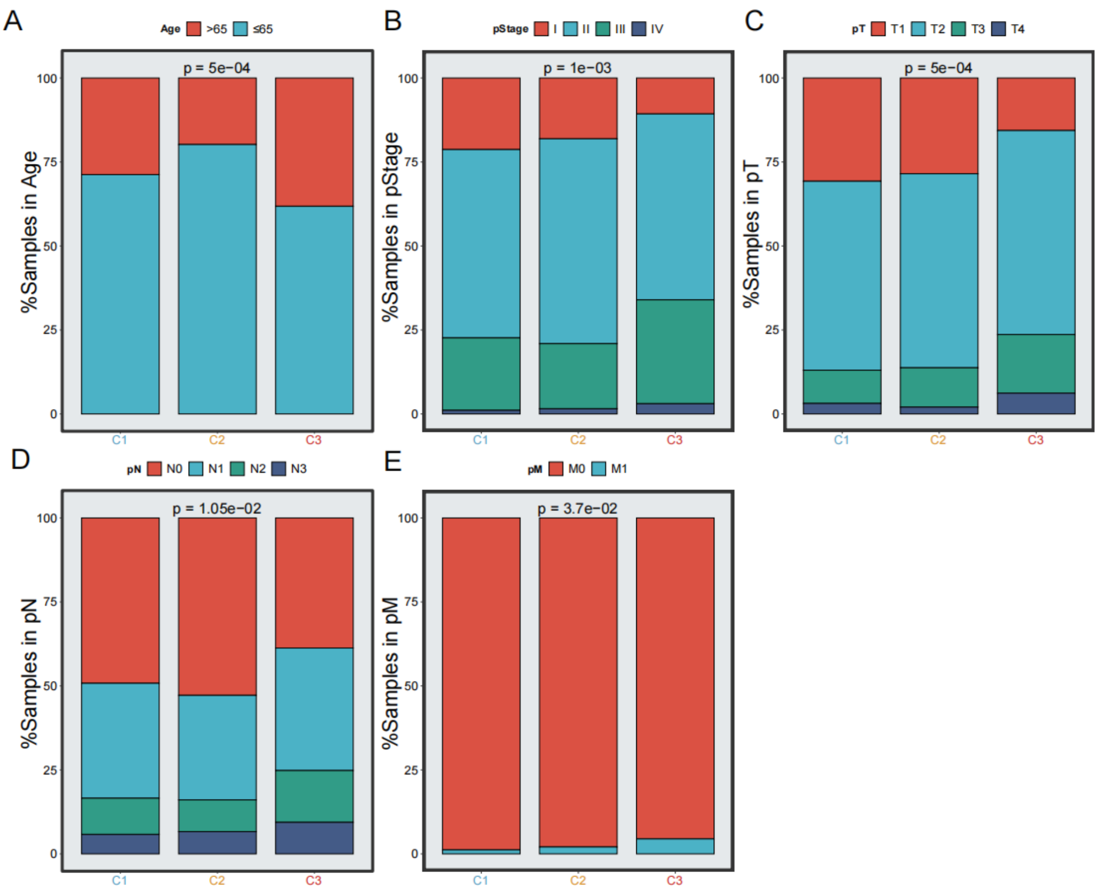

Fig. S2: Barplots showed the distribution of age (A), pathologic stage (pStage) (B), pathologic T (pT) (C), pathologic N (pN) (D), pathologic M (pM) (E). Fisher's exact test.

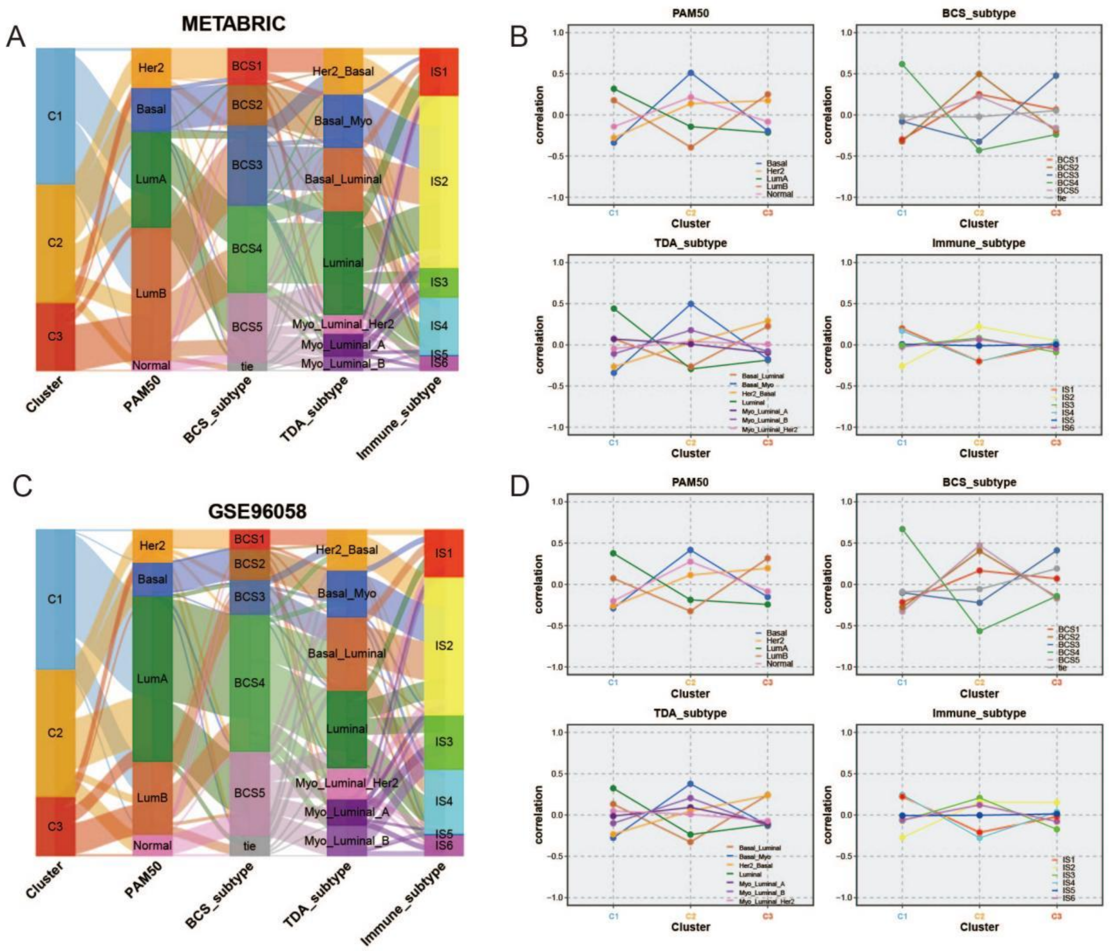

Fig. S3: The underlying transition and correlation between EMT subtypes and four typical classifications in METABRIC (A, B) as well as GSE96058 (C, D) cohorts. Pearson correlation test.

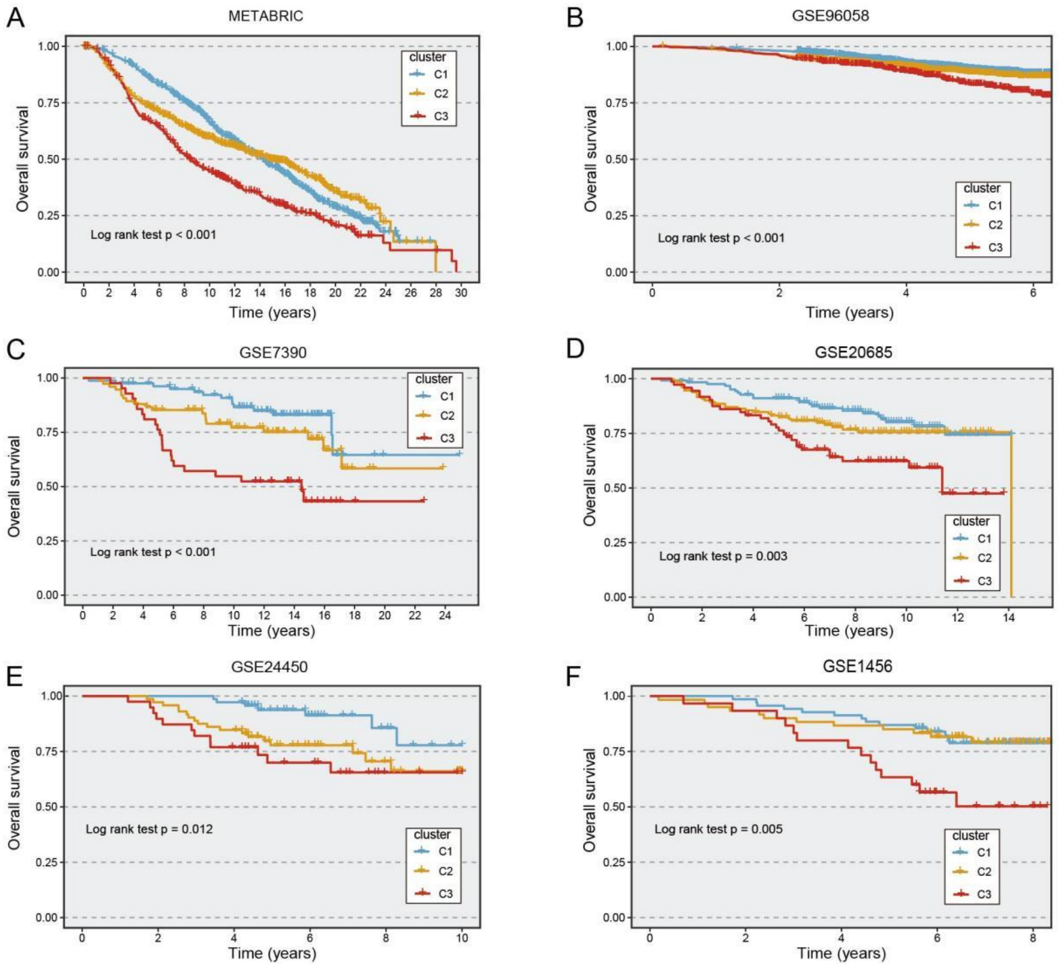

Fig. S4: Survival analysis of OS in six validation cohorts (A-F). Log-rank test.

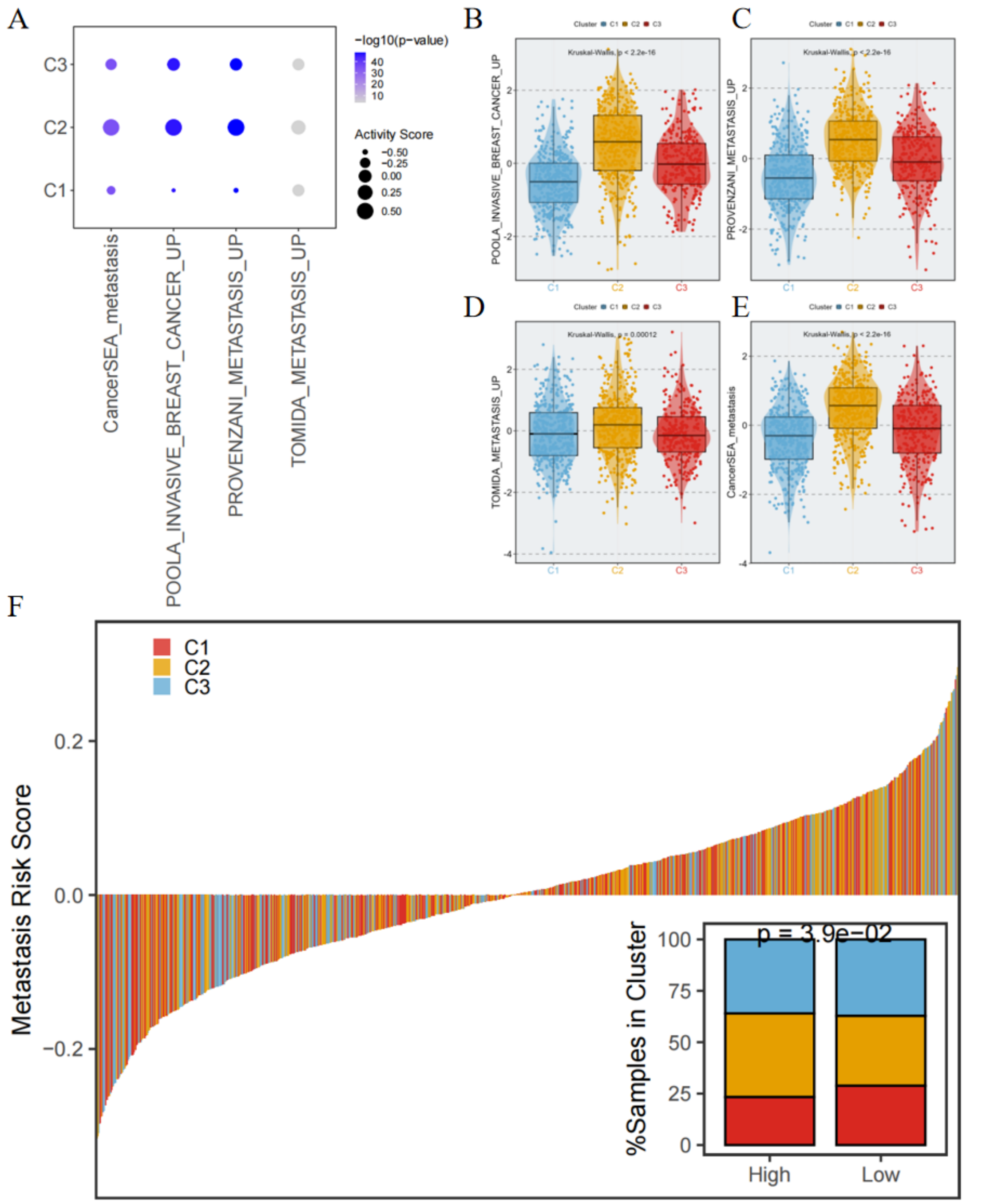

Fig. S5: The evaluation of metastasis potential in three EMT subtypes. A. The dot plot showing the average activity score of metastasis-related genesets in three EMT subtypes. The dot color represents the -log10(p-value). Kruskal-Wallis test. **B-E.** The difference of POOLA_INVASIVE_BREAST_CANCER_UP (B), PROVENZANI_METASTASIS_UP **(C)**, TOMIDA_METASTASIS_UP **(D)**, and

CancerSEA_metastasis **(E)** activity scores among three subtypes was depicted by boxplot. Kruskal-Wallis test. **F.** The risk prediction of BC metastasis potential in TCGA-BRCA patients, comparing among three EMT subtypes. The BC samples were split into high-risk and low-risk groups according to the median value of risk score and the stack barplot showing the proportion of samples from distinct EMT subtypes in two risk groups. Fisher's exact test.

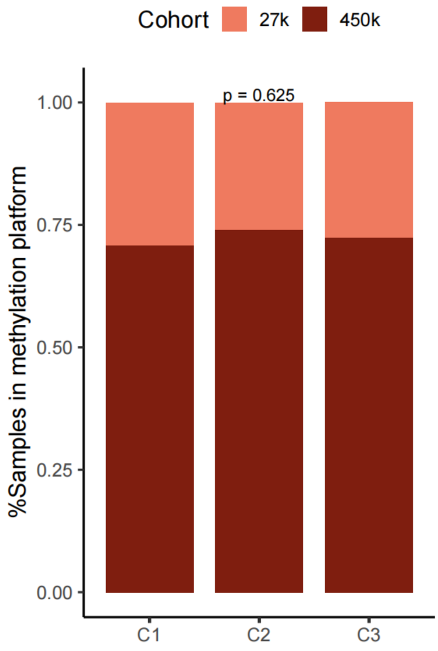

Fig. S6: The proportion of samples from distinct methylation platforms in three subtypes. Fisher's exact test.

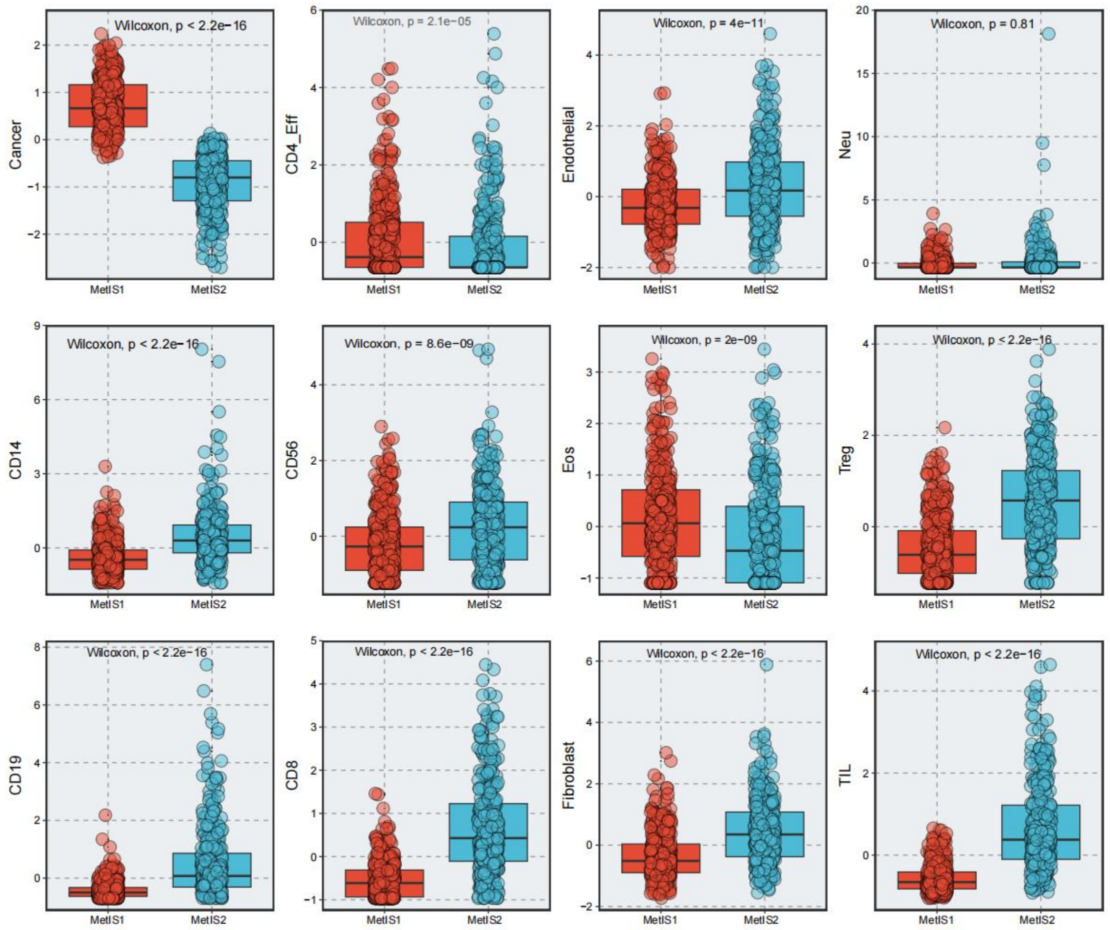

Fig. S7: Boxplots comparing the immune cell populations in the tumors characterized as immunologically cold (MetIS1) versus hot (MetIS2) subtype. Wilcoxon rank sum test.

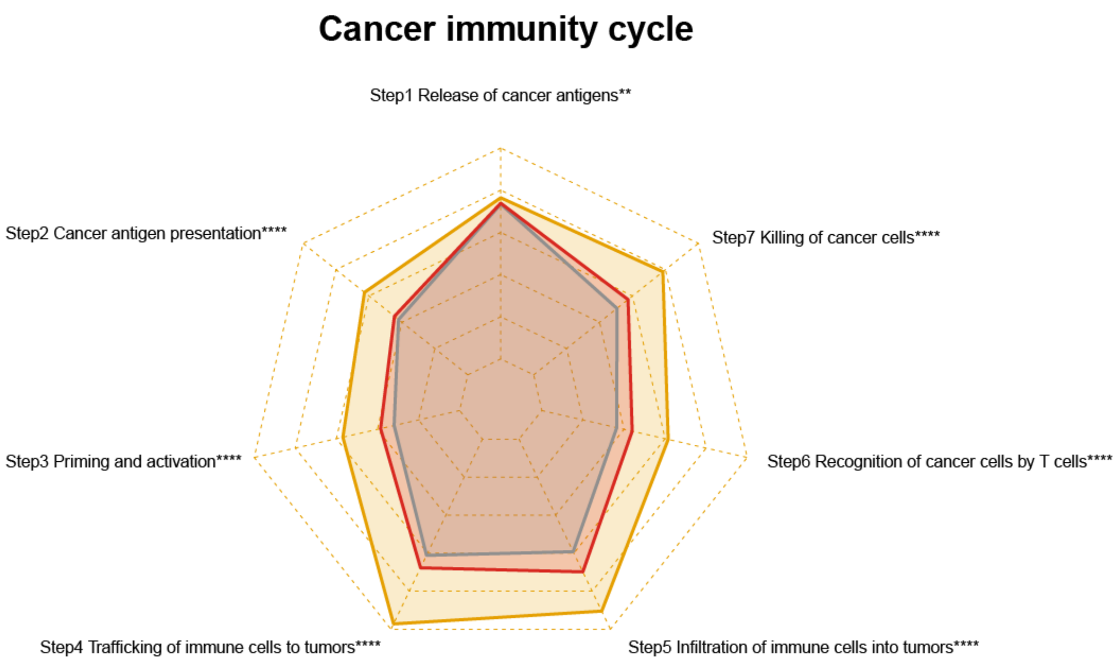

Fig. S8: Radar plots illustrated the cancer immunity cycle patterns in three subtypes. Kruskal-Wallis test.

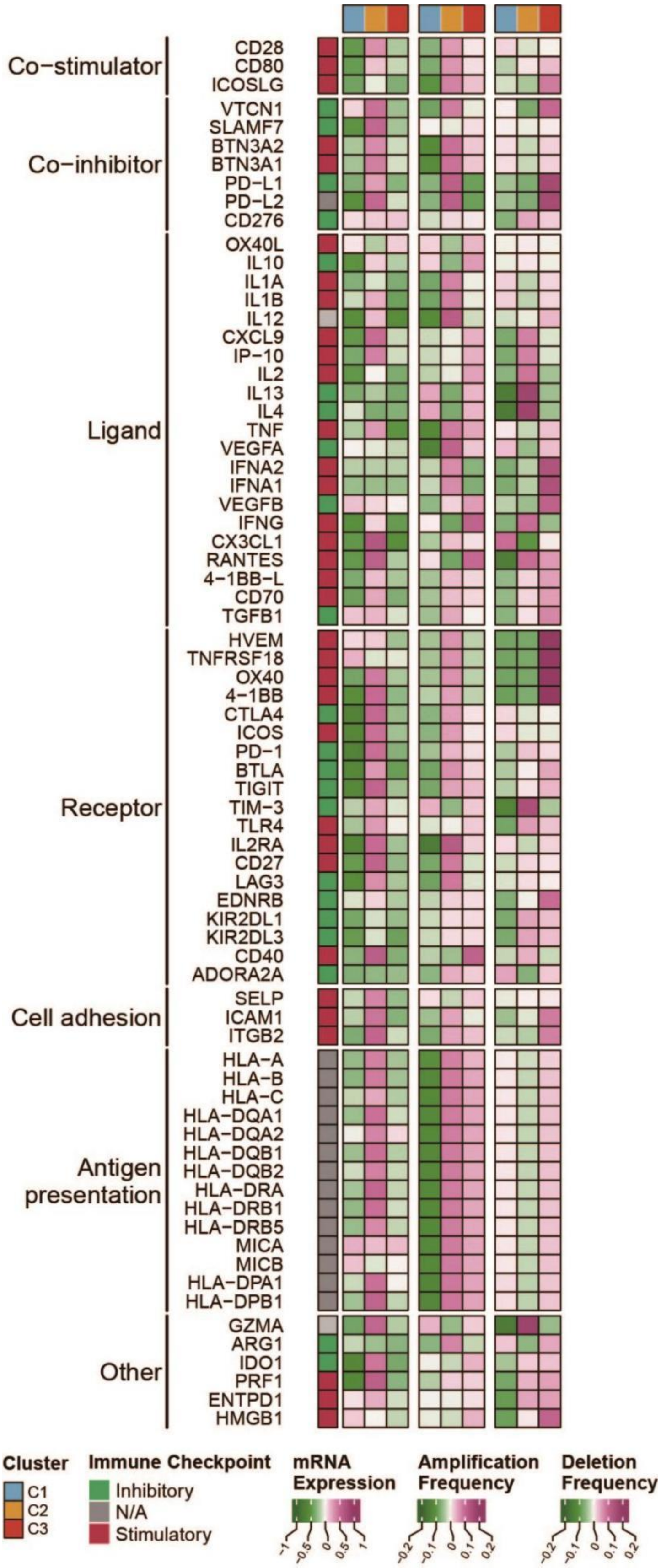

Fig. S9: The differences of immune modulators among EMT subtypes.

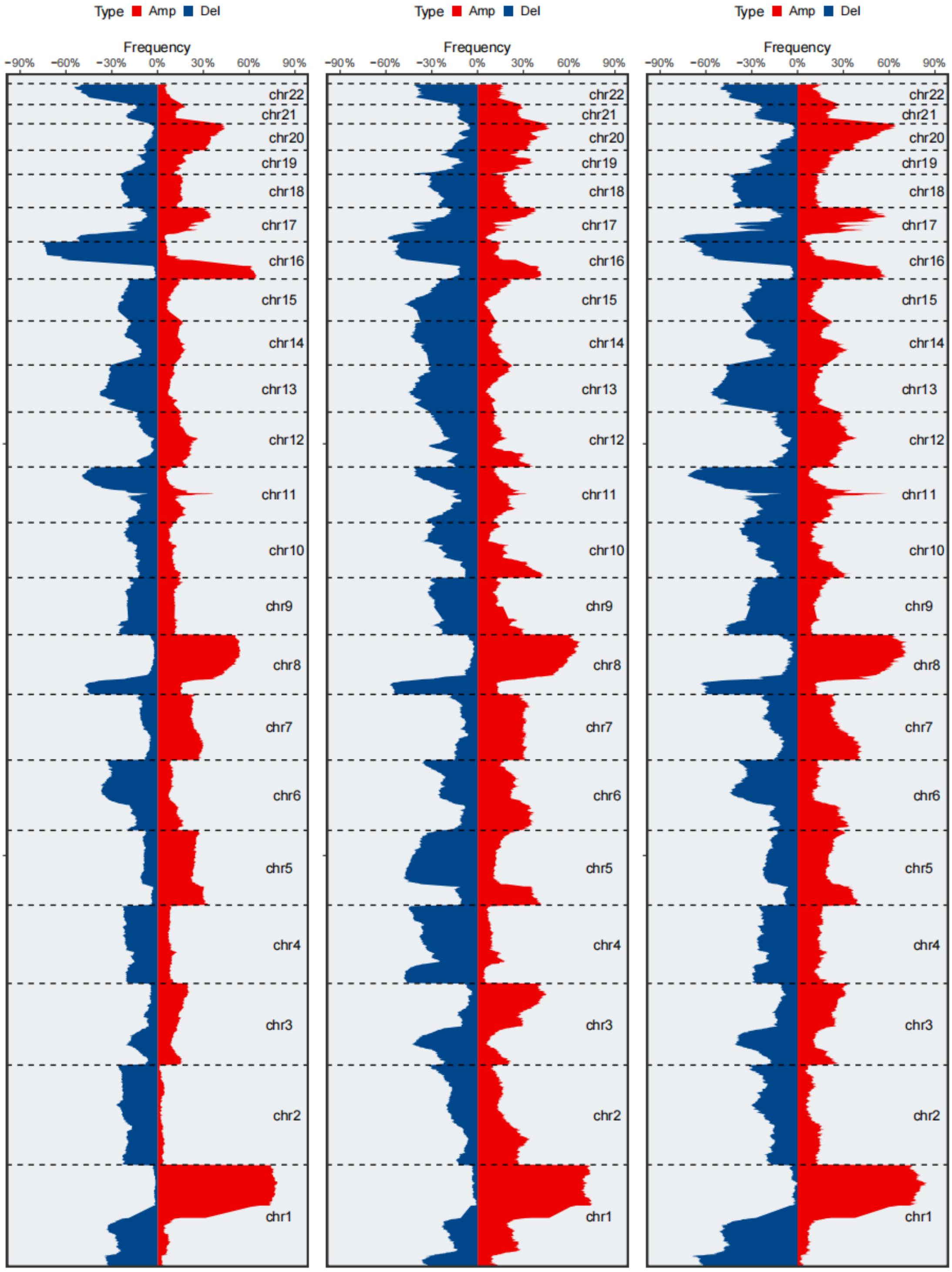

Fig. S10: Arm-level somatic copy-number alteration (SCNA) events in C1-C3 (left, C1; middle, C2; right, C3). Red denotes amplifications and blue denotes deletions.

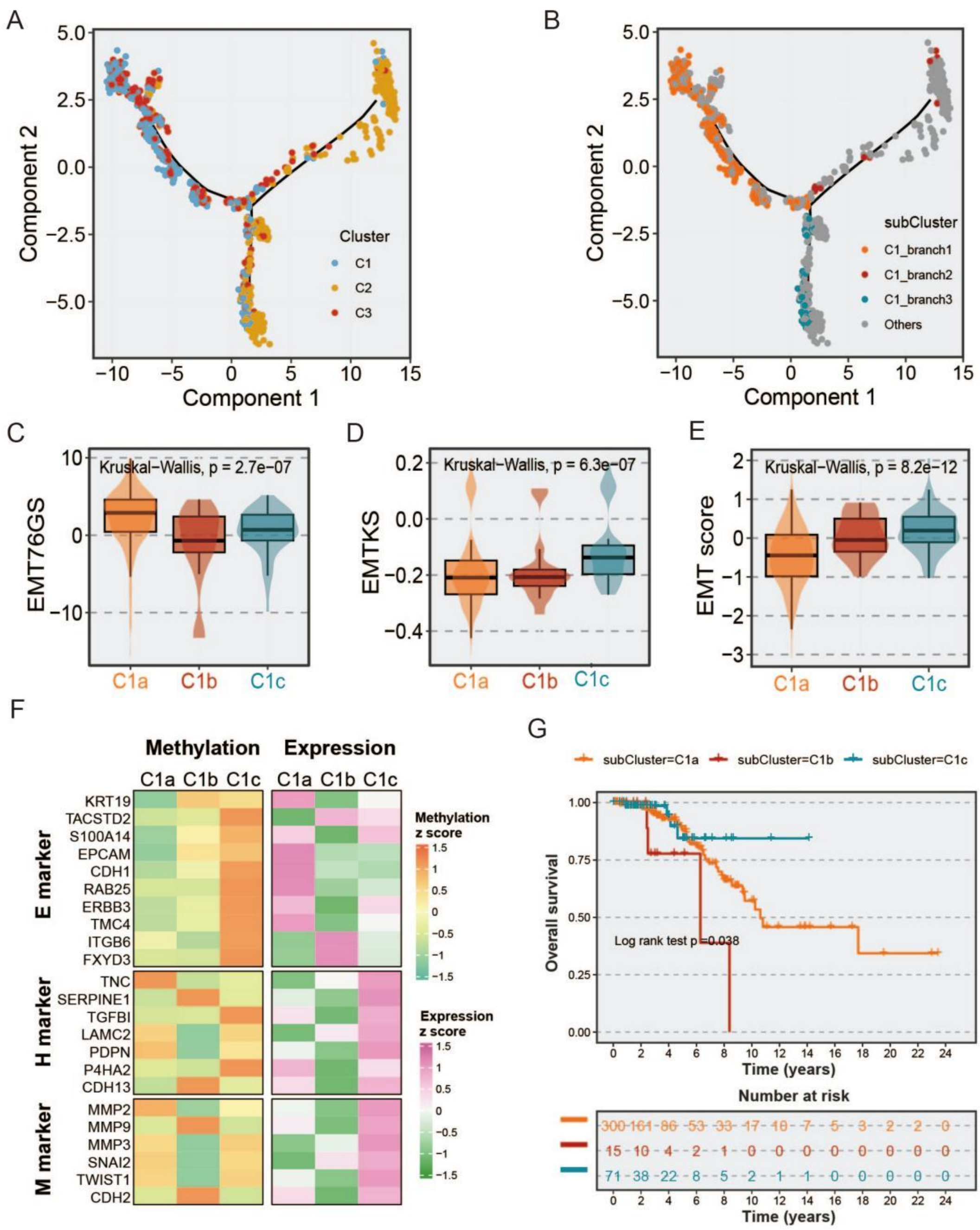

Fig. S11: The dynamic landscape of EMT subtypes. A. The EMT dynamic landscape: each point represents a sample with colors corresponding to the EMT subtype. **B.** Patients of C1 were further stratified into three C1 subprogrammes (C1a-c) based on their location in the EMT dynamic landscape. **C-E.** EMT metrics of the samples in C1a-c. **F.** The normalized mean expression and DNA methylation levels of the 27 genes across C1a-c were indicated by the color gradient. The E (epithelial) markers, H (hybrid) markers, and M (mesenchymal) markers were displayed in the top, middle, and bottom panels, respectively. **G.** Survival analysis of C1a-c.

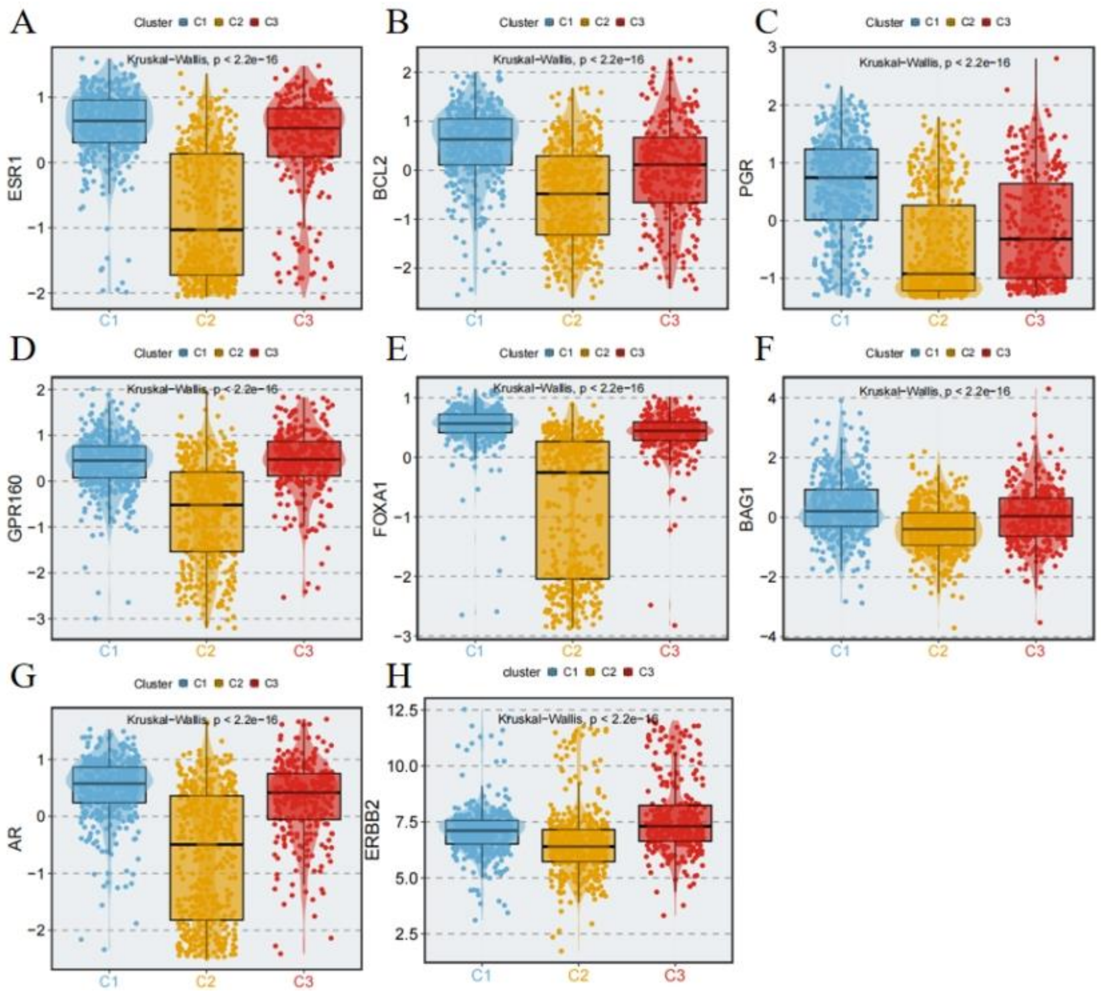

Fig. S12: Boxplot showing the difference of BC-related genes expression in the three EMT subtypes. A-G. The expression of luminal-related genes in the three EMT subtypes. **H.** The expression of HER2 was significantly higher in the C3 subtype compared to C1 and C2 subtypes. Kruskal-Wallis test.
