# Supplementary material for: DNA methylation profiling deciphers three EMT subtypes with distinct prognoses and therapeutic vulnerabilities in breast cancer
Source: J Cancer. 2024 Jul 16;15(15):4922–38. doi: 10.7150/jca.96096 (PMC11310866; doi:10.7150/jca.96096)
Supplement: Supplementary file 1 — Supplementary methods, figures and tables. [file jcav15p4922s1.zip › Table S1.pdf]

Table S1: Baseline distribution of BC cohorts presented in this work								
	Overall	TCGA	METABRIC	SCAN-B(GSE96058)	GSE7390	GSE20685	GSE24450	GSE1456
N	7602	1055	1979	3273	107	327	183	312
Platform								
Affymetrix-GPL570	327 (4.6%)					327 (100%)		
Affymetrix-GPL96	357 (5.0%)				198 (100%)			159 (100%)
Illumina-GPL6947	183 (2.6%)						183 (100%)	
Illumina-microarray	1979 (27.6%)		1979 (100%)					
Illumina-RNAseq	4328 (60.3%)	1055 (100%)		3273 (100%)				
EMT subtype								
C1	2963 (41.3%)	386 (36.6%)	831 (42.0%)	1400 (42.8%)	81 (40.9%)	124 (37.9%)	72 (39.3%)	69 (43.4%)
C2	2733 (38.1%)	394 (37.3%)	727 (36.7%)	1274 (38.9%)	75 (37.9%)	131 (40.1%)	72 (39.3%)	60 (37.7%)
C3	1478 (20.6%)	275 (26.1%)	421 (21.3%)	599(18.3%)	42 (21.2%)	72 (22.0%)	39 (21.3%)	30 (18.9%)
