# Supplementary material for: DNA methylation profiling deciphers three EMT subtypes with distinct prognoses and therapeutic vulnerabilities in breast cancer
Source: J Cancer. 2024 Jul 16;15(15):4922–38. doi: 10.7150/jca.96096 (PMC11310866; doi:10.7150/jca.96096)
Supplement: Supplementary file 1 — Supplementary methods, figures and tables. [file jcav15p4922s1.zip › Table S10.pdf]

Table S10A. Subtype-specific targeted drugs obtained derived from drug screening pipeline based on CTRP databases.

C1	C2	C3
**nutlin-3**	**GSK461364**	**lapatinib**
ABT-737	**BI-2536**	**tanespimycin**
nilotinib	**paclitaxel**	**SB-525334**
tamoxifen	**AZD7762**	sirolimus
BRD1378	**vincristine**	panobinostat
PLX-4720	**dasatinib**	neratinib
BRD-K92856060	**parbendazole**	ciclopirox
Ch-55	**MK-1775**	KU-0063794
AM-580	**gemcitabine**	brefeldin A
Compound 1541A	**rigosertib**	BRD-K45681478
BRD5468	**PHA-793887**	MK-2206
GANT-61	**barasertib**	tandutinib
TGX-221	**topotecan**	BRD-K80183349
BRD-K26531177	**pevonedistat**	PRIMA-1
BRD-K29313308	**alisertib**	KHS101
sildenafil	**doxorubicin**	SB-431542
AZD6482	**YM-155**	MI-1
ML083	**etoposide**	BRD9876
BRD-K37390332	**TPCA-1**	azacitidine
ciclosporin	**bortezomib**	trifluoperazine
vorapaxar	**decitabine**	Repligen 136
WAY-362450	**chlorambucil**	ML203
SJ-172550	**pazopanib**	PF-543
veliparib	**obatoclax**	masitinib
BRD-K16147474	**KW-2449**	BRD-K52037352
	**axitinib**	pandacostat
	**tosedostat**	imatinib
	**olaparib**	
	**NVP-TAE684**	
	**PF-573228**	

sunitinib
indisulam
dacarbazine
BIX-01294
SB-743921
leptomycin B
methotrexate
triazolothiadiazine
CR-1-31B
KX2-391
clofarabine
SR-II-138A
daporinad
SNS-032
STF-31
narciclasine
CAY10618
3-Cl-AHPC
vandetanib
CD-437
ceranib-2
BRD-K70511574
cytarabine hydrochloride
ouabain
pluripotin
SCH-79797
GW-843682X
BRD-K61166597
FQI-2
BRD-K66453893
PD318088
YK 4-279
erastin

SNX-2112
Merck60
LY-2183240
nakiterpiosin
GMX-1778
NVP-231
linifanib
BMS-536924
foretinib
isoevodiamine
momelotinib
mitomycin
curcumin
bosutinib
selumetinib
PL-DI
1S
ML210
NSC23766
saracatinib
ISOX
birinapant
CHM-1
manumycin A
ruxolitinib
cytochalasin B
bleomycin A2
ML311
fluorouracil
triptolide
PF-184
BRD-K34222889
AZD7545

KU-60019
BRD-K66532283
linsitinib
NSC632839
JQ-1
tamatinib
Ki8751
ML320
XL765
lovastatin
Compound 7d-cis
BMS-345541
bendamustine
RG-108
AT7867
piperlongumine
BRD-K63431240
SB-225002
MST-312
LE-135
MLN2238
erlotinib
gefitinib
AZ-3146
TG-101348
NSC 74859
bardoxolone methyl
BRD-K51490254
WP1130
L-685458
SRT-1720
BRD-K19103580
cerulenin

SKI-II
BRD-K11533227
phloretin
crizotinib
nintedanib
ML239
necrostatin-1
BRD-K88742110
betulinic acid
BRD-K02492147
procarbazine
VER-155008
BRD-K17060750
NSC95397
epigallocatechin-3-monogallate
quizartinib
BRD-K13999467
ML031
KU-55933
WZ8040
IC-87114
GDC-0879
BRD4132
lomeguatrib
CI-976
ML006
GSK-3 inhibitor IX
BRD9647
BRD-A02303741
pifithrin-mu
HLI 373
Mdivi-1
BRD1835

silmitasertib
CHIR-99021
BRD8899
pyrazolanthrone
itraconazole
semagacestat
SR1001
CAY10576
etomoxir

Table S10B. Subtype-specific targeted drugs derived from drug screening pipeline based on PRISM databases.

**C1**	**C2**	**C3**
**nutlin-3**	**GSK461364**	**lapatinib**
sulfamethazine	**BI-2536**	**tanespimycin**
epinastine	**paclitaxel**	**SB-525334**
indirubin	**AZD7762**	napabucasin
INH1	**vincristine**	bexarotene
guanidine	**dasatinib**	linifanib
resatorvid	**parbendazole**	LY456236
methylphenidate	**MK-1775**	ku-0063794
propoxycaine	**gemcitabine**	repaglinide
Ro-10-5824	**rigosertib**	beclomethasone-dipropionate
idasanutlin	**PHA-793887**	ecamsule-triethanolamine
AMG-232	**barasertib**	imiquimod
CGM097	**topotecan**	ribociclib
amprolium	**pevonedistat**	PF-05212384
buthionine-sulfoximine	**alisertib**	DMH1
niridazole	**doxorubicin**	tamibarotene
cytarabine	**YM-155**	azodicarbonamide
PF-3845	**etoposide**	buphenine
Ro-90-7501	**TPCA-1**	RG108
tiletamine	**bortezomib**	tecastemizole
ICI-162846	**decitabine**	theophylline

sulfasalazine
endo-IWR-1
ilomastat
UK-383367
lonafarnib
LY2784544
tyrphostin-AG-494
BAY-K-8644-(s)-(-)
cefdinir
TG100-115
SR-27897
AVL-292
cefditoren-pivoxil
KD025
metoxibutropate
lanatoside-c
thiram
cyt387
bifemelane
BMS-986020
abemaciclib
mefexamide
palbociclib
formestane
monensin
ribavirin

chlorambucil
pazopanib
obatoclax
KW-2449
axitinib
tosedostat
olaparib
NVP-TAE684
PF-573228
sunitinib
indisulam
dacarbazine
BIX-01294
fludarabine-phosphate
FR-139317
marimastat
crenolanib
odanacatib
docetaxel
VE-822
epothilone-b
volasertib
dofetilide
irinotecan
tivantinib
emetine
daunorubicin
azacitidine
indoprofen
dinaciclib
CR8-(R)
pardoprunox
ispinesib

oprozomib
CX-4945
adaprev
BMS-599626
ifosfamide
acivicin
5-hydroxymethyl-tolterodine
kenpaullone
thiamine
spermine
vismodegib
CP-724714
maxacalcitol
butamben
PKI-179
osimertinib
4-iodo-6-phenylpyrimidine
tucatinib
azilsartan
tanshinone-i
uridine
metronidazole
PD-407824
alfacalcidol
pentamidine
GW-3965
halofantrine
oxazepam
quinethazone
sodium-tanshinone-ii-a-sulfonate
alpelisib
dihydroergocristine
clotrimazole

enocitabine
astemizole
dolastatin-10
vinflunine
fosbretabulin
LY2606368
ixabepilone
rubitecan
oxymatrine
talazoparib
selinexor
tedizolid-phosphate
flubendazole
paliperidone
ixazomib
PF-477736
belinostat
raltitrexed
ABT-751
bitopertin
VLX600
podophyllotoxin
LY2603618
ZK-93426
LY2183240
pilaralisib
litronesib
verubulin
cabazitaxel
filanesib
ganetespib
isofloxythepin
carfilzomib
lurasidone
CNX-2006
azomycin-(2-nitroimidazole)
methyldopa
MEK1-2-inhibitor
CGS-15943
amfenac
beta-lapachone
taselisib
amoxicillin
halobetasol-propionate
JIB04
sulconazole
voxtalisib
LY2109761
mifepristone
AZD1480

triapine
vindesine
BIIB021
afobazole
EPZ-5676
nilotinib
harringtonine
tozasertib
tofogliflozin
AT-7519
BX-912
D-64131
AZD3463
vidarabine
deferasirox
cediranib
anguidine
bicalutamide
AT13387
cytochalasin-b
NVP-AUY922
AZD6482
SU3327
OTS167
TW-37
AVN-944
CCMI
homoharringtonine
teriflunomide
oseltamivir-phosphate
TAK-901
amonafide
bardoxolone-methyl

tiotidine
10-hydroxycamptothecin
floxuridine
MK-5108
pralatrexate
vatalanib
cholecalciferol
ZM-447439
cycloheximide
alvocidib
hexaminolevulinate
teniposide
CEP-37440
indiplon
pirenperone
azathioprine
dirithromycin
2-methoxyestradiol
amsacrine
genz-644282
alectinib
combretastatin-A-4
TAME
niraparib
talmapimod
VX-765
tioguanine
PRT062070
SCH-900776
CID-5458317
vinblastine
indibulin
atorvastatin

fleroxacin
tazemetostat
hyoscyamine
BMS-754807
tepoxalin
MK-0812
benzydamine
broxyquinoline
regorafenib
temozolomide
LY2801653
valrubicin
GGTI-298
dovitinib
erteberel
batimastat
SC-144
flumethasone
saxagliptin
doxifluridine
LY2334737
golvatinib
oxiracetam
triclabendazole
chlorquinaldol
GW-842166
bromosporine
taltobulin
danusertib
ciclopirox
SB-218078
lerisetron
AZ960

nizatidine
sertindole
GSK650394
epothilone-a
echinomycin
9-aminoacridine
RAF265
BNC105
CP-673451
oxyphencyclimine
idoxuridine
bekanamycin
PF-03814735
caffeic-acid
vemurafenib
3-deazaneplanocin-A
barasertib-HQPA
1-azakenpaullone
genipin
BF2.649
fenbendazole
AC-264613
mepivacaine
NMS-E973
GSK1070916
CMPD-1
lappaconite
betamethasone
kifunensine
triciribine
idazoxan
oxyquinoline
PHA-848125

estrone
letrozole
BMS-387032
nithiamide
vorinostat
lidocaine
SNS-314
ZM-306416
carvedilol
desonide
alvespimycin
tideglusib
panobinostat
cimetidine
TG-02
ginkgolide-a
carmofur
SU014813
puromycin
pelitinib
eltrombopag
naftifine
ribitol
GSK429286A
brequinar
digoxigenin
gambogic-acid
temazepam
BI-D1870
mozavaptan
cetrimonium
TG-101209
nemorubicin

cyclosporin-a
DPI-201106
oxprenolol
PP-1
FLI-06
UNC0631
orotic-acid
KW-2478
AMG900
ponatinib
levocetirizine
cinromide
YM-976
JTE-607
romidepsin
danazol
10-deacetylbaccatin
AZ-628
UNC2250
BAY-11-7085
thiocolchicoside
torcetrapib
STA-5326
GDC-0980
resminostat
FK-866
PP242
P276-00
AZD8330
phenazone
lasalocid
vanoxerine
clonazepam

CB-10-277
LGX818
salvianolic-acid-B
toremifene
4-(4-fluorobenzoyl)-1-(4-phenylbutyl)-piperidine
gilteritinib
nimorazole
NSC-632839
thiomersal
mitoxantrone
LY2874455
PRT062607
LDN-57444
doxycycline
acesulfame-potassium
tacrolimus
navitoclax
temocapril
alexidine
