# Supplementary material for: DNA methylation profiling deciphers three EMT subtypes with distinct prognoses and therapeutic vulnerabilities in breast cancer
Source: J Cancer. 2024 Jul 16;15(15):4922–38. doi: 10.7150/jca.96096 (PMC11310866; doi:10.7150/jca.96096)
Supplement: Supplementary file 1 — Supplementary methods, figures and tables. [file jcav15p4922s1.zip › Table S2.pdf]

Table S2. 5023 EMTAGs used in this study

ABI3BP	ACTA2	ADAM12	ANPEP	APLP1	AREG	BASP1	BDNF	BGN	BMP1	CADM1	CALD1	CALU	CAP2	CAPG	CCN1	CCN2	CD44	CD59	CDH11
CDH2	CDH6	COL11A1	COL12A1	COL16A1	COL1A1	COL1A2	COL3A1	COL4A1	COL4A2	COL5A1	COL5A2	COL5A3	COL6A2	COL6A3	COL7A1	COL8A2	COLGALT	COMP	COPA
CRLF1	CTHRC1	CXCL1	CXCL12	CXCL6	CXCL8	DAB2	DCN	DKK1	DPYSL3	DST	ECM1	ECM2	EDIL3	EFEMP2	ELN	EMP3	ENO2	FAP	FAS
FBLN1	FBLN2	FBLN5	FBN1	FBN2	FERMT2	FGF2	FLNA	FMOD	FN1	FOXC2	FSTL1	FSTL3	FUCA1	FZD8	GADD45A	GADD45B	GAS1	GEM	GJA1
GLIPR1	GPC1	GPX7	GREM1	HTRA1	ID2	IGFBP2	IGFBP3	IGFBP4	IL15	IL32	IL6	INHBA	ITGA2	ITGA5	ITGAV	ITGB1	ITGB3	ITGB5	JUN
LAMA1	LAMA2	LAMA3	LAMC1	LAMC2	LGALS1	LOX	LOXL1	LOXL2	LRP1	LRRC15	LUM	MAGEE1	MATN2	MATN3	MCM7	MEST	MFAP5	MGP	MMP1
MMP14	MMP2	MMP3	MSX1	MXRA5	MYL9	MYLK	NID2	NNMT	NOTCH2	NT5E	NTM	OXTR	P3H1	PCOLCE	PCOLCE2	PDGFRB	PDLIM4	PFN2	PLAUR
PLOD1	PLOD2	PLOD3	PMEPA1	PMP22	POSTN	PPIB	PRRX1	PRSS2	PTHLH	PTX3	PVR	QSOX1	RGS4	RHOB	SAT1	SCG2	SDC1	SDC4	SERPINE1
SERPINE2	SERPINH1	SFRP1	SFRP4	SGCB	SGCD	SGCG	SLC6A8	SLIT2	SLIT3	SNAI2	SNTB1	SPARC	SPOCK1	SPP1	TAGLN	TFPI2	TGFB1	TGFBI	TGFBR3
TGM2	THBS1	THBS2	THY1	TIMP1	TIMP3	TNC	TNFAIP3	TNFRSF1	TNFRSF12	TPM1	TPM2	TPM4	VCAM1	VCAN	VEGFA	VEGFC	VIM	WIPF1	WNT5A
SNAI1	ZEB1	CDH1	TWIST1	CTNNB1	STAT3	HIF1A	AKT1	MIR200C	ZEB2	SMAD3	EGFR	MIR200B	SMAD2	MIR200A	NOTCH1	MIR21	ILK	GSK3B	NFKB1
HMGA2	CXCR4	TP53	EGF	MTDH	MAPK1	BMI1	PTEN	MTOR	RAC1	MMP9	GLI1	MET	SMAD4	KLF4	MYC	SMAD7	SIRT1	CTGF	CCR7
PROM1	TGFB2	PIK3CA	MIR34A	RHOA	AR	CD274	FOXM1	YAP1	SRC	MIR30A	MIR205	MALAT1	JAK2	WWTR1	TNF	RELA	MAPK3	MIR221	MIR145
HGF	TWIST2	TP63	NANOG	SP1	S100A4	PTK2	MAPK7	MUC1	HMGB1	EZH2	ESR1	HOTAIR	CAV1	SOX2	BMP7	BMP4	SHH	PTGS2	POU5F1
KRAS	ID1	H19	FGFR1	FOXQ1	MTA1	GRHL2	TRPS1	ADAM17	T	AURKA	BMP2	CCL18	CCL2	ROCK1	MIR429	ESRP1	PPARG	NTRK2	EPCAM
MIR93	MIR204	MIR203A	MIR186	MIR155	MIR148A	HDAC1	KDM1A	AGER	NUMB	WNT1	EZR	TLR4	TCF3	STK11	UCA1	CCL21	CCL20	LEF1	PAX2
DDR2	YBX1	MCAM	MIR26B	MIR23A	MIR214	MIR19A	MIR150	MIR141	MIR124-1	MIR101-1	LCN2	IL17A	IL1B	IGF1R	IGF1	HSF1	HNF4A	FOXA1	ERBB2
EGR1	KLF8	NES	LINC-ROR	MIR300	NUAK1	DCLK1	CLDN1	RUNX2	TET1	TP73	THBD	TGFBR2	TGFB3	STIM1	SRF	BRCA1	SOX9	SOX4	FSCN1
SIX1	SATB1	BCL2L1	OVOL2	MKL1	NDRG2	EIF5A2	PARD3	PRKCI	AXL	FBXW7	TRIM33	PAK1	OSM	ROR2	MSX2	MIR9-1	MIR30C2	MIR30C1	MIR29C
MIR29B1	MIR26A1	MIR137	MIR130B	MIR10B	MIR106B	MIR106A	MIRLET7	LEP	L1CAM	RHOC	CYR61	BIRC5	HSPA5	HOXA13	ANXA2	PDCD4	GATA3	ALK	FLOT2
FOXO3	FOXC1	FGFR2	AKT2	EPAS1	DNMT1	CUX1	CTNND1	CTBP1	MAPK14	NLRP3	CYTOR	AGR2	NDRG1	CDKN1B	CD24	TNFSF15	GDF15	TP53INP1	TM4SF5
WNT3A	WISP2	NRP1	ACTL6A	CUL4A	FZD7	FOSL1	PDGFD	VASH2	TBL1XR1	TNFAIP8L	YWHAG	YY1	XBP1	WT1	VDR	CCR2	MIR655	NR2C2	TIMP2
TGFBR1	TGFA	TERT	MIR630	MIR590	TCF4	BSG	SPRR2A	SKIL	SMURF2	SFRP2	CXCL5	CCL5	BCL2	RB1	PTPN11	MIR491	MIR485	CEMIP	SALL4
TMPRSS4	MAPK8	PRKCA	MEG3	TUG1	SEMA4C	PIN1	PGF	SERPINF1	GOLM1	PAWR	MIR375	MIR361	NODAL	ATM	MUC4	MMP7	FOXO4	MAP3K4	MAP3K3
MDM2	MIR96	MIR31	MIR222	MIR218-1	MIR181A1	MIR194-1	MIR185	MIR182	MIR181A2	MIR17	MIR143	MIR130A	MIR128-1	MIR10A	JUNB	IGFBP7	HSPB1	HSPA4	HOXB7
FOXA2	HMOX1	HDGF	HAS2	SENP1	RGCC	NEAT1	GPC3	USP22	FOXO1	EFEMP1	VWCE	F2R	ELF5	EPHA2	EDN1	JAG1	DLX2	CTSL	PARP1
CRKL	TXNIP	SPRY2	ADAM10	TRIM28	NAMPT	MIR1271	HDAC6	ZBTB33	CDC42	HS3ST3B1	SETDB1	HDAC4	UBE3C	BAG3	MAGED1	CD36	ABCG2	NTN1	ADIPOQ
RNF8	ACVR1	CCNA2	SQSTM1	SPHK1	HDAC3	WISP3	NRP2	RUNX3	STC2	RUNX1	TNFSF11	BHLHE40	LGR5	HAVCR2	AFAP1L2	DYRK2	PITPNM3	AXIN2	AXIN1
NCOA3	ACTN4	ESRP2	PEAK1	CA9	YWHAZ	KDM6A	TUFT1	TPBG	TP53BP2	TLE1	TIAM1	TGFB1I1	TEAD1	TCF21	MIR616	MIR612	MAP3K7	STAT5B	STAT5A
STAT1	BRAF	SRI	SOX5	BMP6	SKP2	SIM2	SDC2	CCL19	CEACAM	RPS6KB1	ALX4	RNH1	CCND1	KDM5A	RAF1	CIP2A	MTA3	MIR506	MIR497
MIR494	MIR489	PBXIP1	PAK5	CTNNBIP	KLK6	PRSS8	PROP1	MAP2K1	ERBIN	BTBD7	PRKAA1	TRIM62	RNF111	IL17RD	PKD1	CDK14	SUFU	IL23A	MAGEC2
HOOK1	PDCD1	CRIM1	EGFL7	PCBP1	F11R	FURIN	PEBP1	P2RY2	NFKBIA	NF1	NCL	CEACAM	MIR196B	MIR373	MIR135B	ZFAS1	ASCL1	MGAT3	MDK
MCL1	MIR34C	MIR33A	MIR30D	MIR301A	MIR27A	MIR24-1	MIR224	MIR223	MIR218-2	MIR206	MIR20A	MIR199A1	MIR193A	MIR191	MIR187	MIR183	MIR16-1	MIR15B	MIR15A
MIR153-1	MIR146A	MIR135A1	MIR134	MIR132	MIR124-3	MIR124-2	MIR122	MIR101-2	MIR100	LMNA	LGALS3	LASP1	LAMA5	KIT	CD82	JARID2	ITGB4	ITGA3	AQP5
AQP3	IL11	CXCR2	MACC1	HES1	PRMT1	HRAS	HOXB9	HOXA10	HMGB3	HIC1	HDAC2	H2AFX	GSN	UHRF1	CPS1-IT1	HIPK2	GRN	GPR32	ANG
GLI2	GJB1	NOX1	LRIG1	SLC39A6	FZD2	FUT4	BRD4	SIRT3	DICER1	SATB2	BOP1	EPB41L3	KDM4B	FGF9	FGF1	ETV4	ESRRA	ESR2	ERG
EPS8	EPO	EPHB2	GKN2	TET3	AHR	E2F1	HBEGF	DLX4	DAB2IP	PAQR3	CTSZ	GLIPR2	SLC30A7	RBFOX3	CSNK2B	CRYAB	LINC0026	CRK	CREB1
CLDN7	CLDN4	CLU	UHRF2	EHMT2	PROKR1	WASF3	CFTR	KHDRBS1	TRIM16	CEBPB	CEBPA	HOXB13	TACC3	CDX2	FAM3C	CDKN2A	CDK5	NPTN-IT1	SPRY4-IT1
ZEB2-AS1	MIR1236	MIR675	FGF19	RBM8A	MRC2	GAB2	CD151	HDAC9	SEMA3E	VGLL4	CD63	MICAL2	ISG15	PITPNM1	CXCL14	ONECUT2	ROCK2	RASAL2	EIF2AK3
MAP4K4	QKI	HAND1	CYP7B1	MUC16	FOXP2	ZFYVE9	SLC9A3R	KL	LHX2	IGSF8	NREP	CD14	NOG	LRRFIP1	MARVEL	ARHGEF2	SMC3	LATS1	NMI
AOC4P	PSTPIP1	SOCS3	HS6ST2	CCNG2	CDKL2	BTRC	CCND2	TIMELESS	NR1I2	KAT2B	INPP4B	SCEL	TNFRSF1	TNFSF10	TNFSF12	CBR1	EED	ABCC3	CBLB
EIF3I	IRS2	SCIN	IKBKG	PIK3R3	AJUBA	PPM1D	GEMIN2	USMG5	SEMA7A	LOXL3	HINT2	SPZ1	TEAD2	FOXN1	CUL3	KAT8	SPOP	MARVEL	ITCH
YPEL3	CAPZA1	CAPNS1	SMC1A	USP9X	VANGL1	TRIM11	WNT5B	IFT88	SETD7	PTP4A2	MED28	ELL3	NUBPL	FBXO11	MICALL2	LIN28A	TRPM8	DDR1	ZYX
ZNF217	PCGF2	ZNF143	MIR802	MIR454	MZF1	ZFP36	WNT11	WNT6	VTN	VSNL1	VHL	VCP	UCP2	UCHL3	TYRO3	TYMS	TXN	TUFM	C5AR1
PHLDA2	TSC2	TSC1	MIR663A	TRPC5	ACTG2	TP53BP1	TNXB	TNS1	TLN1	TLE4	TJP1	NR2F2	TFCP2	TDGF1	MIR92B	MIR646	MIR639	MIR638	TCF7
MIR573	TBX3	TBX2	TAZ	KLF5	ST14	SREBF1	SOX3	SON	SOD2	MIR487B	STK33	SLC2A1	SKP1	SKI	ST8SIA1	ST6GAL1	SIAH2	SIAH1	SHC1
CPEB1	AKTIP	SFTPC	SOX17	SEMA4A	ELSPBP1	GOLPH3	CX3CL1	CCL25	CCL22	CCL3	SERPINB	ATXN1	SALL1	S100P	S100A9	S100A8	S100A6	S100A2	ROS1
RORC	EXOC4	BCL6	RGS3	RDX	HAS2-AS1	RBP2	SENP2	NTN4	RASA1	PRUNE1	CXCL16	PTPRZ1	NKX3-2	PTPN14	PTPN6	SCUBE2	EPB41L5	PTN	STIM2
MTUS1	MKL2	MIR522	MIR517C	MIR520G	MIR452	RAB22A	CAMK1D	TBX20	POGLUT1	PROX1	AJAP1	PRKCQ	HDAC8	PAG1	KDM3A	FOXJ2	PRKCE	PRKAA2	FERMT1
MIR449A	LGR4	BRF2	IMP3	PTPA	PPP1R8	GIPC2	POMC	ERRFI1	MBD3	PLS3	PLAGL2	PLA2G4A	PIK3R1	SERPINI1	ABCB1	STK26	WWOX	UIMC1	CYB5R1

ACKR4	PDGFB	LIMA1	PRRX2	PDE4A	UBR5	PLAC8	TFDP3	PCSK1	ING4	ANGPTL4	PCMT1	GMNN	PCDH9	PBX3	PARD6A	NEUROG:	IL22	PRDX1	DUXAP9
OPRM1	MIR424	MIR382	MIR381	NUCB2	ROR1	NTRK3	NPPA	NOV	NOTCH4	NOTCH3	NME1	NKX6-1	NFIL3	NFIC	MYL2	MYD88	MYCN	MYBL2	MYB
TRIM37	MUC2	MST1R	MSN	MIR345	MIR331	MIR326	MMP19	MMP13	MMP11	MMP8	MLLT3	ASCL2	MITF	CXCL9	MEF2D	MDM4	ART1	SMAD9	ARRB1
MIR7-1	MIR30E	MIR30B	MIR302A	MIR29A	MIR26A2	MIR25	MIR24-2	MIR23B	MIR217	MIR216A	MIR211	MIR208A	MIR199A2	MIR190A	MIR181B1	MIR153-2	MIR152	MIR149	MIR144
MIR136	MIR129-1	MIR125B1	MIRLET7(	MIRLET7I	MIRLET7I	LYN	LTBP1	LRP6	LMNB1	LIMS1	LIF	LGALS9	USP17L9P	RHOG	NANOGP8	KRT19	KMT5A	KRT18	KRT17
KRT8	KRT7	KIF5B	KDR	KCNN4	KCNH1	JAK3	JAG2	ITGB6	IRS1	IRF6	AQP9	ITGA6	FOXK2	IL18	IL6R	IL4	MCRIP1	IRGM	NANOS3
IDH2	IDH1	IRF8	RAB43	HTN1	HSP90AA	HSPB2	BIRC2	HRG	HPGD	HOXD9	APBB1	HOXA9	HNRNPAI	NR4A1	HK2	HIP1	EHD2	ANXA5	H2AFZ
ANXA1	REPIN1	OLA1	CARD10	GSK3A	BRD7	TBK1	FHOD1	GRIN1	TUSC7	CAVIN1	GPI	GOLGA2	GLRX	GLS	EML4	GLO1	GLS2	AGO2	DKK3
BBC3	LYPD3	GJB2	AMHR2	GH1	GATA6	GATA1	GAPDH	SIN3A	LETMD1	BRMS1	ARMC8	TXN2	SPDEF	FSCN2	CBY1	CERS6	NR5A2	FSHR	IL27
ZMYND8	CCNDBP1	PADI4	MMD	SCRIB	SUZ12	NCSTN	ARHGEF1	NEDD4L	FMR1	EXOC7	FLT1	KDM6B	PLXND1	PEG10	SNW1	ALDOB	CEP164	FKBP5	PHLDA1
XRN2	FHL2	FHL1	SCUBE3	FCN2	ZEB1-AS1	FBP1	FASN	ALDH1A1	F3	F2RL2	F2	ETV1	ETS2	ETS1	ESRRB	ALAD	ERN1	ERF	EPHB3
EPHA8	EPHA4	EP300	ENO1	ENG	FBXO45	RPL22L1	ELK3	ELK1	CYP4Z1	ELAVL1	EIF5A	EIF4G1	EIF4E	EIF2S1	RMST	EFNB2	EEF1D	EDNRA	ECT2
DVL2	AGTR1	DNMT3B	DDX5	DAPK1	CYP3A5	PEBP4	ZNF746	CYP1B1	CMTM8	CSPG4	CSK	CSF2	CRP	CRMP1	FOXR2	CREBBP	ADM	KLF6	COL8A1
KLF17	CNTN1	MSI2	CCR6	CCR5	CLK2	LRG1	CKS2	BATF2	CIRBP	MGLL	VSIG4	EGLN3	PKP3	RASSF1	PTP4A3	BVES	FOXN3	PIM2	EHD1
PPARGC1	PGRMC1	HPSE	CCR9	MTHFD2	KDM5B	GNA13	LEFTY1	PDPN	CXCL13	CEACAM.	FST	MAD2L2	TAB1	RACK1	PAK4	CDKN1A	SPRY1	MSLN	PSME3
LINC0118(	CDK3	G3BP1	TRAP1	CDH13	NR1H3	MICA	OCLN	DNAJB6	MIR1181	CDH5	FRAT1	PDCD6IP	HDAC5	MIR124-2I	MIR875	MIR888	CDH1	CDH11	CLDN2
CLDN4	DDR2	EPCAM	ESRP1	FAP	GLI1	KRT18	KRT19	KRT20	KRT7	KRT8	LUM	MUC1	PALLD	PDGFRB	PDPN	POSTN	SPARC	TNC	CLDN3
CLDN7	DSP	FN1	FOXC2	MMP2	MMP3	SNAI1	SNAI2	SOX10	TWIST1	TWIST2	VIM	ZEB1	ZEB2	ADH1B	AKR1C2	AOPEP	CA2	CADM3	CD24
CDH1	CELSR2	COBL	COL4A2	CRIP2	CXADR	CXCL1	DCN	DKK1	FBLN5	FGF2	FGFBP1	FN2	FPR1	FPR2	FSTL1	FXYD3	GLDC	IGFBP7	IL1RL1
IL2RA	ITGA10	KRT15	MME	PALMD	PCSK1	PLAT	S100A8	SERPINA1	SERPINE2	SFRP1	SLC39A8	SRPX	ST14	ST6GALN	SULF1	VCAN	WNT5A	ACKR3	ATF3
B2M	BCL3	BMP2	CCN2	CD68	CDH12	CDH15	CDH2	CFH	CHKA	COL6A1	COL6A2	CTSZ	CXCL6	CYP1B1	CYP2F1	DUSP1	EGR2	F3	GALK1
GAS1	GRB7	HTRA1	ID2	ID4	IFIT1	IFIT3	IFITM3	INHBA	IRF6	IRF7	ISG15	ITPR1	JUP	KITLG	KLF2	KRT14	KRT5	LEF1	MMP12
MMP13	PADI2	PCOLCE	PDGFRA	PHGDH	PKP1	PLA2G7	PLK2	PMP22	PTGS1	RNASET2	SDC2	SERPINB5	SLPI	STAT1	STAT5A	TGFB3	TGM2	THBS1	TIMP3
UPP1	ZNF239	AHNAK	AKT1	BMP1	COL1A2	EGFR	FZD7	ILK	ITGA5	ITGB1	JAG1	MMP9	MST1R	NOTCH1	PTK2	SMAD2	STAT3	STEAP1	TGFB1
TMEM132	WNT11	CDH11	DKK3	FZD1	IGF1	IGFBP5	LTBP1	MMP14	NRP1	NRP2	NXN	PLAU	PRICKLE1	SFRP2	SFRP4	THBS2	THBS4	ADGRF1	ADGRG1
ANKRD22	ANTXR2	AP1M2	AXL	BSPRY	C1ORF116	CARD6	CDH3	CDS1	CRB3	ELMO3	ENPP5	EPB41L5	EPHA1	EPN3	EPPK1	ERBB3	EVPL	F11R	GALNT3
GALNT5	GRHL1	GRHL2	HNMT	ITGB6	KDF1	KLC3	KRTCAP3	LIX1L	MAL2	MAPK13	MPP7	MPZL2	PATJ	PPARG	PRR5-ARI	PRSS22	PRSS8	RAB25	RBMPMS
S100A14	SCNN1A	SERINC2	SH3YL1	SHROOM.	SPINT2	SSH3	STAP2	TACSTD2	TC2N	TJP3	TMC4	TMEM125	TMEM30I	TMEM45I	TNFRSF2	TSKU	XXYLT1	ECM1	FBN2
FUCA1	GPC1	LAMA2	LRP1	PVR	CAV1	CTNND1	FSCN1	MIR-200	MIR-369-5	MIR-450A	MIR-542-5	MIR155	MIR203A	MIR23B	MIR29C	MIR370	TCF4	TGFB2	GSC
TJP1	ALDH1A1	ASPA	BMP7	CTNNB1	CXCL13	DAB2IP	ERBB2	ESR2	FOXA2	FOXP3	FZD4	GLI3	GSK3B	IGF1R	IGF2	ITGA6	MMP7	PTCH1	PTCH2
PTPRN2	SHH	SOX9	SRC	TCF3	WNT5B	ADAM9	AEBP1	ANGPTL2	ASAP1-IT	ATP2B1	CALM1	CDKN1A	CLEC11A	COL1A1	COL3A1	COL5A1	COL6A3	DYRK2	EMILIN1
GNS	HSPG2	IL13RA1	LAMA4	LAMB1	LIMS1	LINC0031	LRRC32	MAFB	MRC2	MRPL52	NID2	NNMT	PDE8A	PHTF2	PKD2	PLIN3	PLXDC1	RABGGTA	RIPOR1
SEC23A	SEMA5A	SHOX2	SKAP2	TGFB1I1	THY1	TPP1	ABCA12	ABCC4	ACSL4	AGPAT5	AGR2	AIF1L	ANLN	ANP32E	ANXA1	ANXA9	AP1S2	ARL4A	ARSJ
ATCAY	AURKB	BIRC3	CAMK4	CCDC82	CCDC88A	CD83	CEBPA	CELSR1	CENPW	CGAS	CHN1	CIP2A	CKMT1A	CKMT1B	CKS2	CLDND1	CLSPN	COMMD8	CTNNAL1
DCBLD2	DDX60	DDX60L	DKFZP58(	DNAJB4	DNER	DPH3	DUBR	E2F7	ECHDC1	EFNA1	ELF3	ESRP2	EST_AA2	EST_AA4	EST_AA5	EST_AA6	EST_AI19	FABP5	FAM92A
FAM92A1	FLJ32810	FLJ36445	GALNT1	GBP1	GCA	GGCT	GNAI1	GOLGA2F	GORAB	GULP1	HDAC9	HID1	HJURP	HMGA2	ICA1	IFI44	IFIT2	IGF2BP3	IGSF3
IGSF9	IKBIP	IMPA1	KIF21A	KRT8P45	L2HGDH	LACTB	LAD1	LIFR	LLGL2	LNX2	LOC12698	LOC20245	LOC38969	LOC40158	LOC44028	LRRC1	LYN	LYST	MACC1
MAK16	MAP3K1	MAP7D3	MARVELI	MCAM	MGAT5B	MLPH	MPZL3	MREG	MSX2	MYB	MYBL1	MYH14	MYO5BP2	NCAPG	NOL8	OCLN	OSTM1	PAG1	PBK
PDP1	PIK3R3	PKP3	PLEKHF2	PMAIP1	PNMA2	POLK	POT1	PPM1L	PREX1	PRKCH	PROM2	PRR15L	PRXL2A	PTTG1	PTTG3P	PUM3	RAD18	RB1CC1	RBBP8NL
RBM47	RND3	RP2	RP6-213H	SACS	SAMD9	SAPCD2	SCML1	SELENBP	SHTN1	SLC29A2	SLC9A3R	SMC5	SOAT1	SPDEF	SPDL1	SPINT1	STRIP2	SUSD5	SYNE2
SYNM	TBC1D30	TOM1L1	TPD52	TSPAN1	TSPAN13	TSPAN15	TTC27	TTK	UBLCP1	USP33	VAMP8	WDCP	WDR19	WDR47	XM_1655	XM_3746	XM_4968	YBX2	ZG16B
ZMYM1	ZNF788P	ZNF860	ACP1	CYP27B1	CYP4F11	DDR1	FADS1	GNAL	IL1RN	MAP7	PTPN3	RGS4	SNCA	TRIM29	WWC1	DAB2	FMO1	GBP3	HIF1A
MTHFD2	PPIC	PROCR	SYTL1	MMP1	SDC1	SMAD3	ACTA1	AHNAK	ANGPTL4	BCL2	BCL9	CALCR	CALD1	CAMK2N	CASP3	CAV2	CCNB2	CCND1	CD36
CD47	CDC42	CLDN12	CLDN23	COL5A2	DACT1	DESI1	DHFR	DLG1	DSC2	EGF	ELANE	ESR1	EZH1	EZH2	FGF1	FGF5	FHL1	FLNA	GADD45A
GADD45E	GNG11	HRG	IGFBP4	ITGAV	ITGB3	LAMB3	LOXL2	MAP1B	MITF	MMP10	MSN	MTA3	NLK	NODAL	NPPB	NRG1	NUDT13	PARP1	PDGFB
PLEK2	PLG	PTP4A1	PXN	RAC1	RGS2	SCARB2	SCRIB	SERPINE1	SNAI3	SOX11	SPP1	TFPI	TFPI2	TIMP1	TMEFF1	VPS13A	WEE1	WNT2B	WT1
YWHAG	ACPP	ADAM23	ADAMTS	ADGRF1	AFF3	AGR3	AK5	AKAP12	ALDH3B2	ALPK2	ANK3	ANKRD1	ANTXR1	ANXA6	AOX1	AQP3	ARHGAP8	ARHGDIE	ARMCX1
ATP2C2	ATP8B2	ATP8B3	B3GNT3	BDNF	BICC1	BICDL2	BLNK	BMERB1	BNC2	BVES	C11ORF52	C1ORF21(	C1S	CAP2	CAVIN1	CCL2	CDH4	CEACAM:	CEACAM6
CGN	CLDN11	CLIP3	CMTM3	CNKSR1	CNTNAP2	COL12A1	COL4A1	COLGALT	CPA4	CTAGE4	CYBRD1	DAPP1	DENND2I	DIO2	DLC1	DMKN	DOCK10	DPYSL3	EDIL3
EHF	ELF5	ELOVL2	EML1	EMP3	EPDR1	ERP27	EVI2A	F2R	FA2H	FAAH2	FAM110C	FAM171A	FAM83A	FAT4	FBN1	FBP1	FGD2	FGFR1	FLRT2
FUT1	FUT3	GCNT3	GFPT2	GJB6	GLIPR1	GLIPR2	GNB4	GOLT1A	GPC6	GPR176	GPR87	GPX2	GRAMD2.	GREM1	GSDME	HAS2	HEG1	HOOK1	HS3ST1

HS3ST3A	HS6ST2	IL13RA2	ILDR1	INAVA	INPP4B	JAM3	KIRREL1	KLK10	KLK5	KLK6	KLK8	KRT16	LAMA3	LAMC2	LCN2	LCP1	LGALS1	LHFPL6	LIPG
LOX	LRATD1	LRATD2	MARVEL	MB	MBNL3	MISP	MRAS	MSRB3	MUC20	MYO5B	NAP1L3	NAV3	NDN	NECTIN4	NEGR1	NEXN	NID1	NPNT	NUDT11
OR2A4	OVOL2	P3H2	PAK6	PAPPA	PDE7B	PLA2G10	PLAGL1	POPDC3	POU2F3	PPL	PPP1R14C	PRKD1	PRR15	PRR16	PTAFR	PTGIS	PTX3	RAPGEF5	RASEF
RASGEF1	RBM24	RBMS3	RBPMS2	RECK	RFLNB	RFTN1	S100A9	S100P	SCEL	SH2D3A	SIRPA	SLC2A3	SLC47A1	SLC6A14	SORBS2	SPINK5	SPRR1A	SPRR1B	SPRR3
SRGN	ST3GAL2	STEAP4	STX19	SYDE1	SYK	SYT7	TBXA2R	TMC5	TMEM15	TMEM47	TMPRSS1	TMPRSS1	TMPRSS4	TMSB15A	TNFRSF1	TOX3	TRPA1	TTC22	TTC28
TTC39A	TTC9	TTLL7	TUB	TUBA1A	UCHL1	VTCN1	WDR72	WFDC2	WIPF1	ZCCHC24	ZFPM2	A2M	AARD	ABLIM1	ACSS1	ACTBL2	ADRA1B	AHNAK2	ALDH1A1
ARHGEF2	ARID5B	ARL4D	ARTN	BCAM	BCAR3	C16ORF7	CAPG	CARD10	CD22	CD274	CD44	CDCP1	CIB1	CLIP4	COL13A1	COL17A1	COTL1	CTSB	CXCL8
CYB5B	CYP51A1	CYSTM1	DHCR7	DOCK2	DSG2	DTX4	DUSP7	DYSF	ECM1	EFHD2	EFNB1	ELK3	EMP1	EPHX4	EPS8	ETS2	FAM167B	FAM214B	FAM25A
FDFT1	FGFR2	FOSL1	FOXQ1	FRMD5	FTSJ1	FURIN	FUT8	GAD1	GALE	GEM	GJB3	GPR158	GSTM3	GSTM4	GUCY1A1	HHEX	HHLA1	HMGA1	HMGCS1
HMOX1	HMSD	HSD17B7	IGFBP6	IL1R2	IL4R	INSIG1	IQCN	ISG20	ITGBL1	KCNMA1	KCNN4	KLF10	KLF15	KRT4	LBH	LEMD1	LIF	LPCAT1	LRRD1
LY6E	LYPD1	MAG	MALL	MAP4K4	MGLL	MGP	MSMO1	MT2A	MUC16	MVD	MYCN	MYOF	NIBAN1	NOG	NPR3	NTNG1	OCIAD2	PADI1	PADI3
PCED1B	PCSK9	PDZK1IP1	PHLDA1	PHLDA2	PIR	PLAUR	PLP2	PMEPA1	POTEI	PPM1J	PTHLH	PTPRH	QSOX1	RAB20	RAC2	RHOC	RPS6KA4	RUNX1	S100A16
S100A2	S100A3	S100A4	S100A5	S100A6	SCARA3	SERPINB1	SERPINB2	SERPINB	SFN	SH2D5	SLC1A4	SLC37A2	SMIM29	SMO	SOCS2	SOSTDC1	STRA6	SYTL2	TBC1D2
TGFA	TGFBI	TINAGL1	TMEM37	TMPRSS3	TNFAIP3	TNFSF9	TNNC1	TPBG	ULBP2	WARS1	WNT4	WNT6	XDH	ZNF185	CDH2	HGF	HOXA5	CXCL10	IFI16
IFI27	IFI30	IFIH1	IFITM1	IFITM2	IRF1	IRF9	IRGM	MX1	OAS1	OAS2	OAS3	PHYIN1	PSME1	SOCS1	STAT2	ABCA1	ABCA12	ABHD17C	ABLIM3
ACKR3	ADAM19	ADAMTS	ADAMTS	ADAP1	ADH1A	ADH1C	ADIRF	ADORA2	AGAP2-A	AKAP1	AKR1B10	AKT3	ALDH3A1	ALDH3A2	ALOX5A	AMY1C	ANKRD1	ANKRD2	ANKRD20A2
ANO6	AP1S3	APCDD1L	APOC1	ARHGAP	ARHGAP	ARHGAP	ARHGEF4	ARHGEF4	ASPHD1	B4GALNT	BC017676	BEND6	BET1L	BIK	BMF	BPGM	BX004987	C11ORF8	C12ORF75
C15ORF6	C17ORF9	C19ORF3	C1ORF22	C2ORF15	C5AR1	CA11	CARHSP1	CASZ1	CAVIN2	CBLC	CBR3	CCDC57	CCDC74B	CCDC80	CCDC84	CCDC88C	CD9	CDC42BP	CEP170
CES4A	CFD	CFI	CGB3	CGNL1	CHCHD10	CHDH	CHRNA9	CHST2	CHSY1	CLDN1	CLMN	CMTM8	CMTR1	CNPY4	COBLL1	COL16A1	COL27A1	COL8A1	COMTD1
CPEB1	CPLX1	CSF1R	CTSC	CTSD	CTSL	CYFIP2	CYP4X1	DAPK2	DBNDD2	DDAH1	DEFB103	DHRS9	DIXDC1	DNAH11	DNAJB5	DOK7	DOP1A	DST	DYRK3
E2F2	ECHDC2	ECSCR	EDN1	EEF1A2	EIF1AX	ELL3	ENKUR	ENTPD2	ENTPD8	EPB41L4	EPHA10	EPS8L1	EPS8L2	ETS1	EVA1A	EXOC6	EXPH5	FAAH	FAM114A1
FAM184A	FAM201A	FAM225A	FAM225B	FAM92A1	FBLIM1	FBXO32	FCSK	FDXR	FGFR3	FILIP1L	FLJ22536	FLJ25917	FLJ36031	FLJ45248	FNDC4	FRMD6	FTL	GABARA	GALNT10
GATM	GBP5	GJA1	GLB1L2	GLS2	GMPR	GNA12	GNB5	GPD1L	GPD2	GPR68	GRHL3	GRTP1	GSE1	GSN	GSR	HCAR3	HKDC1	HRK	HS3ST3B1
HSD11B2	HSPA4L	ICAM1	IER3	IFFO1	IGFBP2	IKZF2	IL18	IL1R1	IL20RA	IL24	IL32	IL4I1	IL6R	INPP5D	IRF5	ITGB2	JAG2	JARID2	JAZF1
JCAD	KATNAL	KCNK2	KCNK5	KCNQ5-I	KDELR3	KIAA1217	KIF3C	KRTAP19	LAMA5	LARP6	LIMCH1	LINC0023	LINC0031	LINC0208	LMCD1	LOC1005	LRRC15	LRRC73	LRRC8C
LSR	LTBP2	LY6D	MAF	MAGED2	MAN1A1	45352	MCRIP1	MCUB	MDGA1	MELTF	MERTK	METTL7A	MEX3B	MFAP2	MFSD6	MGC2328	MGC4294	MGC4473	MIR193BHG
MIR31HG	MIR9-3H	MLLT11	MROH6	MSANTD	MSC-AS1	MXRA7	MYH16	MYO5C	NAGK	NAV1	NCK2	ND1	NFIB	NOS1AP	NOXA1	NPDC1	NPHS2	NPR2	NR6A1
NRCAM	NREP	NRIP3	NT5E	NUP210	ODAPH	OFD1	OLFML2A	OR5L2	P3H1	P3H3	P4HA2	P4HA3	PAEP	PAPSS2	PARD6B	PCP2	PDE4DIP	PDIA6	PDLIM3
PDLIM7	PERP	PGM3	PHLDB3	PHOSPH	PIK3C2B	PIK3CD	PIM3	PKIG	PKP2	PLAAT3	PLAAT4	PLEKHA6	PLEKHG4	PLEKHO1	PLOD2	PLS1	PLXNB1	PM20D2	PODNL1
PPFIBP2	PPP1R9A	PPTC7	PRICKLE	PROC	PRODH	PRRG2	PSCA	PSMD2	PTGES	PTPN22	PXDC1	RAB13	RAB17	RAB26	RAB31	RAI14	RCAN2	RDM1	RGCC
RGS17	RGS18	RHOV	RHPN1	RINL	RNF144B	RNF2	RNF43	ROBO4	ROR1	RP11-327	RP11-551	RP11-60I3	RP11-849	RTP4	RUBCNL	RUNX2	S1PR4	SAMD10	SAMD12
SBK1	SCG5	SDR16C5	SEC14L2	SEC24A	SEC24D	SELENO	SERPINB	SERPINB	SERPINH1	SH2D2A	SH3RF2	SHISAL1	SHISAL2A	SHMT1	SKIL	SLAMF8	SLC12A7	SLC16A5	SLC22A4
SLC22A5	SLC25A1	SLC25A1	SLC26A2	SLC27A2	SLC29A1	SLC2A14	SLC30A3	SLC7A5	SLCO2A1	SLIT2	SLIT3	SMAD7	SMARCA	SMIM22	SMIM3	SMPDL3B	SNHG12	SNHG18	SNTA1
SNTB1	SOCS3	SORL1	SORT1	SPARCL1	SPHK1	SPOCD1	SPRY1	SPTBN2	SPTLC3	ST3GAL1	ST5	STARD3N	STEAP3	STON1	SUSD2	TBC1D8	TBL1X	TENM2	TEX29
THEM6	TLCD4	TLR2	TM7SF2	TMED7-T	TMEM121	TMEM13	TNFAIP6	TOB1	TPST1	TPST2	TRAF4	TRAK1	TREM1	TUBA3C	TUBA4A	TUBA8	TUBB3	UNC5B-A	VASN
VAV3	VEGFC	YIF1B	Z83851.1	ZDHHC11	ZDHHC14	ZDHHC23	ZNF474	ZNF488	ZNF697	ZNF775	CDK4	ACTA2	BCL2A1	BMPR1A	CASP7	CASP9	COL4A3	EZR	MKI67
ACTN1	ADAM12	ALDH1A3	ALDH5A1	AMIGO2	ANKLE2	APBB2	AREG	ARFGAP1	ARHGAP	ARNTL	ATP8B1	BHLHE40	BMPR2	C1ORF11	C3ORF52	CD59	CDK14	CEBPD	CFB
CHST11	CITED2	COL7A1	CP	CRLF1	CYB5A	CYTH1	DAAM1	DEFB1	DEPTOR	DHRS2	DOCK4	DSE	DUSP10	EPAS1	EPB41L4	EPHB2	EREG	ERMP1	FERMT2
FHOD3	FLJ10357	FLJ14213	FLJ20273	FOXD1	FSTL3	GAL	GASK1B	GDF15	GPRC5C	GRB10	HPGD	HRH1	HSF2BP	IL11	IMPA2	INHBB	JUN	JUNB	KCNJ15
KLF7	MANSC1	MAP1LC3	MATN3	MBOAT2	MBP	MICAL2	MN1	MTUS1	MYL9	MYO10	NCF2	NEDD9	NKX3-1	NR2F2	NUAK1	PDGFA	PDGFC	PDK4	PEA15
PEG10	PID1	PODXL	PTPN21	PTPRK	RAB38	RALA	SCG2	SEMA3C	SERPINB1	SIK1	SLC16A7	SLCO4A1	SLN	SMURF2	SORD	SOX2	SPOCK1	SQOR	SRRD
STC1	SULT1A1	SYBU	TAGLN	TAGLN2	TBX3	TFAP2A	TIMP2	TJP2	TMCC1	TNFAIP2	TNS1	TP53I3	TPD52L1	TPM1	TPM4	TUFT1	VGLL3	XYLT1	ZNF365
ACTN1	ADGRF4	AFAP1L2	ARHGEF1	BEAN1	BICDL1	CERCAM	CHST3	DBN1	EEPD1	GALNT2	KCTD11	LTBP3	LTBP4	MAPRE2	METRNL	MLXIP	MUC5AC	MUC5B	NCOR2
NKAIN4	PPP1R13L	PPP1R18	PXN-AS1	RHOD	SAMD4A	SHANK3	TGFBR1	THRB	TRIO	TRMT10A	TSPAN2	WNT7A	ZFP36L1	BRCA1	CCL7	CCT6A	CENPA	CHEK1	CHRNB1
CLU	COL18A1	CSF1	DCK	FBLN2	GATA2	GLG1	HMGB1	LY75	MAP4	MCM3	MPDZ	NCAM1	NR2F1	OAT	ODC1	PAFAH1B	PLA2G4A	PLK4	PROS1
RBBP6	RBL1	RPSA	SMAD1	SMARCD	TEAD2	TOP1	TPP2	TRAF3	ZFX	CDKN2C	FBLN1	IGFBP3	PLPP3	PRKCA	PTGER2	SYNE1	SYT11	ALDH1A3	ANXA8
CCN3	FAM20A	FST	GGT5	GPM6B	HHIP	IL1B	KRT17	KRT6A	KRT6B	KRT6C	NTSR1	PAMR1	PDGFRL	SAA1	SNED1	STXBP6	SULT1B1	TP63	SNAI1
ADAM23	CCN1	CCT3	CREG1	CYP24A1	DPM1	FLJ32053	FY8	JRKL	KLC2	LPL	PCNA	PI3	POU2F1	PRTN3	PSMG1	RFNG	STK17A	SVIL	TNFRSF11B
TRPM7	UBN1	ACOT1	ADGRA2	ADPRH	APBB1	ARHGEF1	ARL6IP5	ATP6V1C	B3GNT9	BCL11A	BLMH	BNIPL	C11ORF2	C1QTNF6	CALML3	CALU	CCDC102	CCDC120	CCL9
CCR2	CDH13	CEACAM	CELF4	CFL2	CFP	CHRNA1	CLEC4A	CLIC3	CSGALNA	CYSLTR1	CYSRT1	DEDD2	DNM1	DOK1	EFEMP2	EHBP1L1	ELOVL7	ENDOU	ENO3
EPB41L2	EXPI	F13A1	FAM83H	FCGR2B	FCGRT	FCRLS	FKBP7	FKBP9	FLNC	FOLR2	FXYD5	FZD6	GAMT	GJB2	GPR34	GPR85	GSDMA	GZMH	HIP1R

HLX	HTRA3	IDE	IL17RE	ITGB1BP1	JDP2	KCNK1	LOXL1	LOXL3	LPAR1	LRBA	LSP1	LTC4S	LTF	MARVELI	MATN2	MEDAG	MRC1	MXRA8	NAAA
NBL1	NEK9	NFATC1	NIPAL2	NRROS	NRTN	OLFM1	OSR1	OTUD7B	OVOL1	PHKA1	PHLDB1	PKD1	PLA2G15	PLEKHA3	PLEKHG6	PLEKHH1	PLXND1	POF1B	POFUT2
PPIF	PRRX1	PRRX2	PTPRF	RAP1B	RHPN2	RIPK4	RNF157	RRAD	SDR42E1	SELENON	SEMA7A	SH3GLB1	SLC5A9	SNX20	SNX6	SNX9	SPI1	SPTBN1	ST8SIA4
STARD10	STK17B	TFAP2C	TGFBR2	THRSP	TJAP1	TMEM184	TMEM54	TMEM79	TMPRSS2	TNK1	TRAF1	TSPAN4	TTYH2	VSIG4	WAS	WNK1	AP1M2	ARHGEF5	CD99L2
CTAGE6	SLC44A2	ZNF165	CD99L2	281047401	ASAP1	BAZ1A	CAST	D3ERTD2	DPP8	EIF5A2	FAM3C	GNA13	ITGBA	JAK2	KIAA1841	KPNA3	MLF1	PHF20L1	PIP4K2A
PKIA	RBMS1	TCF12	TMEM167	TMEM65	TWSG1	UBXN2A	ZBTB44	ZNF266	YAP1	ABCC4	ABR	AQP9	ARHGEF6	CCDC50	CD52	CD93	CHST7	CITED1	CLGN
CORO1A	CSPG4	CTSH	CXCL12	CYBA	DPYSL2	ELMO1	EPB41L3	EPHA4	FGF13	GDF6	GYPC	HCK	HEXA	HEY1	HSD17B1	ITGA11	ITM2A	JAM2	JAML
KAT2B	KHDRBS3	LAMP2	LDHA	LDHB	LDHC	LIN7B	LY96	MBNL1	MDFIC	MET	METRNL-	MMP11	MOXD1	MYO5A	NCKAP1L	NDRG1	NDRG2	NECTIN3	NPL
NR0B1	PCDH18	PHACTR2	PRDM5	PRDX1	PTPRB	PTPRM	RAB27A	RASSF4	RCN3	RET	RGL1	RNF130	RRAGD	SH3KBP1	SLC7A8	SLC9A6	SMARCA4	SMARCD3	SMPD1
SOX17	ST3GAL6	STX11	THBD	TMEM88	UQCRFS1	VAV1	ADGRL2	AFP	ALB	ANXA2	ARHGAP4	ARHGEF7	BMP4	CAVIN3	CCND2	CCND3	CDHR5	CXCR4	ENG
FGF9	FLRT3	FOXA3	FOXP1	FYN	GLI4	HEY2	ID1	IGF2-AS	ITGB4	ITGB5	PTN	RHOBTB3	SHC1	SKAP1	TMSB4X	VIL1	FOXC1	BCL2L1	FGF8
RHOB	VTN	ADAP1	ALS2CL	ANXA3	ARRDC1	BAIAP2L1	C6ORF132	CAMSAP3	CD46	CHMP4C	CMTM4	CNNM4	CRYBG2	CXCL16	CYB561	EPB41L1	F2RL1	FAM83B	FAM83F
FHDC1	FOXA1	FRK	GSTO2	HDHD3	HOOK2	ITPKC	KIAA1211	KIAA1522	KIAA1671	KLF5	LIPH	LOC10050	LYPD3	MYO6	P2RY2	PLEKHA7	PLEKHG3	PLPP2	PRKCZ
PRRG4	PWWP2B	RNF39	SDC4	SLC12A8	SLC44A3	SLC52A3	STYK1	SULT2B1	SYNE4	USP43	VGLL1	VWA1	AGER	CAV1	CBR1	CCN6	CDKN2A	EDNRA	EGR1
ETV4	FLT1	FOXM1	GATA3	HBEGF	HOXB7	HOXB9	HS6S72	HSP90AA	IDH2	KIT	KL	L1CAM	MYC	POU5F1	PROM1	PTGS2	RUNX3	SIM2	TCF21
TDGF1	TGFBR3	VCAM1	VDR	VEGFA	VSNL1	VWCE	ADAM12	ADAMTS	ADAMTS	AP1G1	CNOT1	CNRIP1	COL10A1	DYNC1LI	OLFML2B	BAMBI	COMP	LAMC3	MIR141
MIR200A	MIR200B	MIR200C	MIR429	MYD88	TLR4	ABCF3	ARHGEF9	AVPR1A	BMX	CAPN6	CNIH3	DIO3	DLGAP1	DONSON	EXOC3L2	GCNA	GNB1	GUCY2F	HCAR1
HTR2A	IRX3	MAGEA8	MIR-346	MIR-507	MIR542	MYPOP	QRFPR	C3ORF52	CLVS2	FREM1	KMO	MIR-30A-	MIR-30A-	MIR-30C	MIR-30E-	MIR-630	MIR130B	MIR17	MIR192
MIR193B	MIR215	MIR30B	MIR30D	MIR30E	PCDHB10	SCD	SEPTIN14	SLC17A3	SLC22A24	SLC25A29	ZNF343	FN1	ABI3BP	ACTA2	ANPEP	APLP1	BASP1	BGN	CADM1
CDH6	COL11A1	COL5A3	COL8A2	COLGALT	COPA	CTHRC1	ECM2	ENO2	FAS	FMOD	FZD8	GPX7	IL15	IL6	ITGA2	LAMA1	LAMC1	MAGEE1	MCM7
MEST	MFAP5	MSX1	MXRA5	MYLK	NOTCH2	NTM	OXTR	PCOLCE2	PDLIM4	PFN2	PLOD1	PLOD3	PPIB	PRSS2	SAT1	SGCB	SGCD	SGCG	SLC6A8
SMIM6	TNFRSF12	TPM2	ANK2	CDH17	CDH13	ADAM19	ARPC4	CD151	EIF4EBP1	FHL2	FOCAD	HSPB1	ITGA3	KIAA1549	LEFTY2	MGC1733	NPC2	NPTX1	PAWR
PCDH1	PDLIM2	PGRMC2	PIK3IP1	PLSCR3	PPP1R14B	RSU1	TAX1BP3	TLL2	TUBA4B	ADGRE5	NECTIN1	NECTIN2	AFTPH	LNX1	UROD	ADAMTS	ANO1	APCDD1	BHLHE41
BST2	BTBD11	C5ORF66	CACNA2D	CBS	CCDC170	CD109	CES3	COL9A3	CPLX2	CRABP2	CST1	CYP3A7	DKK4	DTX3	DYNC1I1	EDAR	EDARADD	FAM131B	FAM86KP
FBXO27	FGD5	FGF20	FIBCD1	FOXL2	GGN	GPSM3	H2AJ	HILPDA	HOXB2	HOXC10	HPDL	IGF2BP1	IL36G	INHBE	ISYNA1	KRT74	LINC0269	LMO2	LOXL4
MRI1	MROH8	MT1A	NAT16	NOTUM	NPIPB9	NPW	NRN1	NRSN2	OBSL1	PARVB	PAX6	PCSK1N	PDE3B	PLIN2	PRF1	PTP4A3	RAMP1	RAPGEF3	RASA3
RASGRF1	RBM20	RIMKLB	SAMD11	SCN5A	SESN3	SLC16A6	SLC17A9	SLC25A27	SLC38A5	SLC5A1	SLC6A16	SPRED3	STC2	TEX19	TGFBR3L	TMED7	TMEM163	TNFRSF11	TRPV2
TUBB2B	VPREB3	ZNF704	ZSWIM5	BAD	IKBKG	LPAR2	MAP2K3	PDPK1	RAB10	RAB5C	RIPK3	SIRT1	WNT10B	FZD2	NKD2	PLCB2	AHNAK2	C10ORF9	CDX1
CDX2	DDC	GPA33	MEIS2	NR1I2	OPMP22	TRABD2A	WWTR1	ACYP1	ARPC2	ATOX1	BCAP29	BCAP31	BCAR1	BCLAF1	C9ORF78	CALB2	CALM3	CASK	CBX3
CBX5	CCDC124	CCT2	CDC37	CDV3	CFDP1	CHMP4B	CNDP2	CNN3	CYCS	DAG1	DBI	DDX39A	DEK	DHPS	DNAJA1	DUT	DYNLT1	EEA1	EEF1B2
EIF3G	EIF3H	EIF3I	ERAP1	FARSA	FKBP4	GANAB	GDI1	GIPC1	GOLGA4	GPI	GRB2	GSPT1	H4-16	HDDC2	HEXIM1	IFI35	IMPDH2	IPI008872	JPT2
KARS1	KIAA0174	LRRFIP1	MAP7D1	MECP2	MED15	MTHFS	MYCBP	MYL12B	NAA50	NRDC	NSFL1C	OGFR	OSTF1	OXSR1	P4HA1	PAK2	PFN1	POGLUT3	PPID
PRR14L	PSMC4	PSMC6	PSMD4	PSMD9	RAB11B	RAB6A	RABEP1	RPL10A	RPL3	RPLP1	RPRD1B	RPSAP19	RTF1	SAP30BP	SH3GLB2	SKP1	SP100	SRSF1	STIP1
SUB1	SUMO1	TAF1C	TBCB	TCOF1	TMOD3	TPD52L2	TRIP10	TTC1	TTLL3	TXLNA	TXNDC12	UAP1	VASP	VBP1	WBP11	ZYX	NFKB2	PDGFD	AK5
AKR1B1	BCAT1	CA12	CHI3L1	COX7A1	DPYD	DSEL	ENPP1	ENPP2	EXT1	GBE1	GLT8D2	GPX8	GXYLT2	MGST1	PAM	PDE1C	PLCB4	PLPP4	PTGR1
UGCG	ABCB6	ABCC6	ABCG1	ABL2	ACADSB	ACBD5	ACSM3	ACY1	ADA	ADARB1	AHCYL1	AHR	AIMP1	AKR1C3	ALDH3B1	ALDH6A1	ALDH7A1	ALDH9A1	ANAPC13
ANG	APBA3	APMAP	ARHGAP4	ARNT2	ARRB1	ARRDC4	ASB8	ASH1L-A	ASS1	ATM	ATP6V0E	ATXN1	AUH	AVPI1	AXIN2	AZIN2	B4GALT4	BABAM2	BATF2
BCAT2	BCL6	BICD1	BIVM	BMP2K	BNIP3	C1ORF53	C1R	C1RL	C5	CABLES1	CABYR	CACNA1D	CAMK2D	CAPN2	CBFB	CBL	CD302	CD3EAP	CDC14B
CDC25B	CDC42EP	CDC42SE	CDKN1B	CDPF1	CDR2L	CEMIP	CEMIP2	CHI3L2	CLEC18A	CLIC1	CLN8	CLUAP1	CNKSR3	COL4A4	COQ7	COQ8A	CRISPLD2	CRY1	CRYL1
CRYZL2P	CSGALNA	CSRP1	CST7	CTCFL	CTDSPL	CTPS1	CTSO	CXCL2	CXCL3	CXXC5	CYP2U1	DAPK1	DCBLD1	DCXR	DDIT4L	DGLUCY	DHRS13	DHRS3	DIP2B
DIPK1A	DNAJC6	DPY19L1	DUSP6	EBPL	ECH1	EEF2K	EFEMP1	EFNB2	EID3	EIF4B	EIF4E3	EML4	ENAH	EPHB1	EPHX2	ESAM	EVA1C	EXOC3	FAH
FAM107B	FAM111A	FAM13A	FAM171B	FAM89B	FAM8A1	FAT3	FBXO38	FHL3	FHOD1	FLOT1	FMNL2	FOXF2	FUT9	FYCO1	GALM	GALT	GCFC2	GCH1	GCHFR
GCLM	GDA	GLI2	GLIS3	GLRX	GLS	GMDS	GPALPP1	GPAM	GPAT3	GPC4	GPCPD1	GRAMD2	GRB14	GSTA4	HDHD5	HECA	HHIPL2	HIP1	HMBS
HSBP1L1	HSDL2	HYAL3	HYI	HYKK	IER2	IFRD1	IL27RA	ILVBL	IRF2BP2	JADE2	JMJD1C	KCNB1	KCNG1	KCTD15	KCTD3	KCTD6	KIAA1755	KMT5B	LANCL1
LAT2	LETMD1	LGR4	LHPP	LIMK2	LIMS2	LOC10013	LOC73010	LUZP1	LXN	LY6G6C	LY6G6D	MAOA	MAP2K6	MAP3K5	MAST4	MCEE	MEG3	MEGF9	MICALL1
MIR22HG	MIR600H	MMD	MMUT	MOB3B	MR1	MRPL54	MRPS6	MTAP	MYLIP	MYO1B	NAMPT	NANOS1	NCALD	NCEH1	NCOA3	NEAT1	NHS	NIPAL3	NMB
NNT	NQO1	NRAS	NSMCE4A	NT5DC1	NUDT16L	ORAI2	OSR2	PAXIP1-A	PCDH17	PCK1	PCNX1	PER2	PGD	PGM2L1	PHLPP1	PHTF1	PIAS1	PITPNC1	PKDCC
PKIB	PKM	PLCXD1	PLEKHA8	PLEKHH2	PMM1	PNKD	PNMA1	POLR2L	PPARA	PPP1R3B	PPP1R9B	PRDX2	PRKAB2	PRKAG2	PRXL2B	PSMB8	PTGER4	RAB40B	RAP1GDS1
RASGRP1	RCL1	RDH10	REXO2	RGS20	RIMKLA	RNASE4	RNASEL	RNF141	RNFT2	RPL5	RPLP0	S100A11	SALL2	SASH1	SAV1	SCAMP1-A	SECTM1	SEMA3B	SH3BP4
SLC12A2	SLC18B1	SLC20A1	SLC27A3	SLC2A1	SLC2A12	SLC2A8	SLC35F2	SLC4A4	SLC7A1	SLC7A6	SMOX	SNAP23	SOCS5	SOD2	SPECC1	SPSB1	SPTSSA	SSBP2	STEAP2
STK32B	STMN3	STX17	STXBP5	TBL1XR1	TCEA3	TFEB	THSD7A	TIGAR	TMEM106	TMEM205	TMEM41A	TMEM97	TNFRSF14	TRIM24	TSGA10	TSNAX	TSPAN12	TSPAN7	TSPAN8

TST	TTC32	TXNIP	UBASH3B	UBE2C	UBE2L6	UBTD1	ULK1	UPK1B	USP13	UTP25	UTRN	VAMP5	VAV2	WDR1	WWOX	YIPF5	YOD1	ZBTB46	ZC3H12A
ZDHHC2	ZMYND1	ZNF331	ZNF395	ZNF585A	AR	ATF2	BMI1	CD34	KLK3	PECAM1	PGR	SP1	BMI1	QKI	RLN2	ADTRP	ALOX15B	ANXA8L1	ARHGAP25
BDKRB2	BIN1	CA9	CAMK2B	CCDC92	COPZ2	CREB3L1	CST6	CTSV	DEPP1	DPT	DSG3	E2F5	ENOX1	ERVMER	FADS2	FAT2	FLJ20366	FZD3	GLYR1
HS3ST2	IRX4	KANK2	KIAA0040	KIAA0888	KIZ	KLK7	KRT81	LGALS7	MYO1D	NAIP	NEBL	NEFM	NMU	NUP62CL	OLFML3	POLR3G	PPM1D	RHBDF2	RNF128
RPS6KA1	RTEL1	S100A7	SCCPDH	SEPTIN6	SLC2A9	SLC6A10	SLC6A10	SNX10	STAC	STARD13	SYNC	TMEM40	TNFRSF6	TNS3	TRAM2	TSHZ1	UGDH	ZBED2	ZBTB38
MALAT1	RRAS	RUNDC3	ABCC3	ABHD11	ANK2	ARHGAP	ASPN	AZGP1	BCAS1	C1ORF54	C4ORF19	CCL8	CD163	CD2AP	CEACAM	CHRDL1	CLEC2B	CLIC4	COL14A1
COL15A1	COLEC12	CORO2A	CRYAB	CSF2RB	CSRP2	CTSK	DHCR24	FAM174B	FGL2	FLI1	FUT2	FXYD6	GALNT7	GIMAP4	GIMAP6	GPRC5A	GUCY1B1	GZMK	IL10RA
ISLR	KCNJ8	MEOX2	MFAP4	MS4A4A	MS4A6A	MYH10	NR3C1	OR7E14P	PALM2AK	PDZRN3	PLLP	PLN	PLXNC1	PTGDS	PTK6	PTPRC	RAB11FIP	RAPGEFL	RARRES2
RUNX1T1	SAMSN1	SERPINF1	SERPING	SLC22A1	SLC35A3	SLC44A4	SOBP	SPAG1	SYNE3	SYNGR2	TFF1	TFF3	TRPC1	TRPM4	TUBB6	ADA	PAFAH1B	PAFAH2	SRM
TNXB	UPP2	VLDLR	ADAM10	ADAM17	ADCY1	ADCY2	ADCY4	ADCY5	ADCY6	ADCY7	ADCY8	ADCY9	AKT1S1	AKT2	AP2A1	AP2A2	AP2B1	AP2M1	AP2S1
BAD	CALM2	CHUK	CLTA	CREB1	CSK	EPN1	EPS15	EPS15L1	FOXO1	FOXO3	FOXO4	GAB1	GRK2	GSK3A	HGS	ITPR2	ITPR3	KRAS	LRIG1
MAP2K1	MAP2K2	MAPK1	MAPK3	MAPKAP	MDM2	NR4A1	PDE1A	PDE1B	PIK3CA	PIK3R1	PLCG1	PRKACA	PRKACB	PRKAR1A	PRKAR1B	PRKAR2A	PRKAR2B	PRKCD	PRKCE
PTEN	RAF1	RPS27A	RPS6KB2	SH3GL2	SH3KPB1	SOS1	SPRY2	STAM	STAM2	THEM4	TRIB3	TSC22D4	YWHAB	ACAA1	ACOT2	ADGRA3	ADORA1	AFAP1	ALYREF
AMOTL1	APOO	ARAP1	ARHGAP	ARMC9	ATP5PB	BAZ2A	BOK	BRI3BP	BTBD19	CAND1	CASP2	CBX6	CCDC33	CCNA2	CCNB1	CDCA8	CDH24	CENPO	CLEC1A
COA7	COPS3	COQ2	COX20	COX7B	CPD	CPE	CSRNP2	CTDSP2	CTNNA1	CYP2E1	CYP2S1	DDX23	DNMBP	DRAM1	EDEM2	EEF1E1	EFCAB14	EHD2	ERCC6L
FAM120B	FAM20C	FLNB	FMNL3	FN3K	FNBP1	GLRX3	GOLPH3	GPX5	GTF3C6	H6PD	HACD2	HAVCR1	HERPUD1	HES2	HINT3	HLA-F	HNRNPU	HSDL1	HYLS1
IDUA	ILF3	INF2	IQSEC2	IRF2BPL	KIAA2026	KIF11	KLF3	KLHL24	KPNA2	LIPK	LMNB1	LRP5	LSM5	MACROH	MAD2L1	MAPKAP	MAPRE3	MBTPS1	MBTPS2
MCM10	MCM2	MCM4	MFSD14B	MOAP1	MRM2	MRPL19	MRPL48	MRPS15	MRTO4	MSR1	MTCH2	MTHFD1	MYBL2	MYOM3	MYRF	NAA16	NCAPH	NDC1	NDUFAF8
NDUFV2	NETO2	NINL	NOL10	NUDT4	OIP5	OPRL1	OTUD5	PARK7	PCMTD2	PDLIM5	PGGHG	PLXNB2	PNRC1	POGLUT2	POLA2	PRPF40B	PSMD5	PSME3	PTPN13
RAET1E	RANBP1	RANBP3	RAP1GAP	RASSF3	RBBP7	RFC3	RFWD3	RRBP1	RRM2	SAMD9L	SART3	SCUBE1	SEMA4C	SEPTIN10	SH3PXD2	SLC37A3	SLC44A1	SMC1A	SOD1
SPAG5	SPATA13	SPATS2L	SSRP1	STAT6	STK39	SUPT16H	SUSD6	TIGD2	TIMELES	TK1	TMA16	TMEM167	TMPO	TOMM40	TRIM32	TRIM9	TXNRD1	UBE2B	UBQLN2
UNC93B1	USP7	VAPA	VDAC3	VEZF1	VPS54	VWA7	WDR34	WDR62	WFS1	ZER1	ZFYVE1	ZNF517	ZNF862	ZWINT	ABCB8	ABI1	ACAA1	ACOX1	ACSL1
ACSL3	ACYP1	ADAL	ADAM15	ADD1	ADGRG6	AGAP3	AGTPBP1	AHSA2P	AK2	AKAP11	AKAP13	ALG9	ANAPC16	ANKHD1	ANKRD1	ANXA11	APH1B	APLP2	ARFGAP2
ARFIP1	ARHGAP1	ARHGAP1	ARHGEF1	ARMCX6	ARMT1	ARNTL2	ATG4B	ATP11C	ATP13A2	ATP13A3	ATP2C1	ATXN2	BAZ2B	BDP1	BIRC5	BNIP2	BOLA2	BORA	BRD8
BRD9	BUD23	C12ORF4	CAM2G	CAMK2G	CAMKK2	CANX	CAPRIN2	CARS2	CCAR1	CCDC66	CCNB1IP1	CCNL1	CCNT2	CCNYL1	CCP110	CD164	CD320	CDC42BP	CDCA3
CDCA7L	CDK10	CDK11B	CDKAL1	CELF1	CENPI	CEP41	CERS5	CERT1	CHORDC	CIAO2A	CKLF	CLASP1	CLEC2D	CLIP1	CLK2	CLPB	CLSTN1	CMTR2	COA1
COA8	CPEB2	CRAMP1	CRCP	CREBBP	CSDE1	CSPP1	CUTC	DAGLB	DBF4B	DBNL	DCAF10	DCAF16	DCLRE1C	DCUN1D	DDX19B	DDX50	DENND4	DEPDC1	DEPDC5
DHRS4	DNAJC16	DNAJC8	DNM1L	DOCK7	DOCK9	DPH5	DPP7	DTX2	DYRK4	ECI2	ECT2	EEF1AKM	EHBP1	EHMT2	EIF2AK4	EIF4A2	ELOVL1	ENOSF1	ERC1
ETFA	ETNK2	EXD2	EXOC7	EXOG	EXTL2	FAM126A	FAM136A	FAM13B	FANCA	FANCB	FANCI	FARSB	FBXO44	FDPS	FIP1L1	FNIP1	FOXJ3	FPGS	FRA10AC1
FRG1	G3BP1	G3BP2	G6PC3	GALNS	GALNT11	GOLGA2	GOLIM4	GOLT1B	GOSR2	GPBP1	GPR180	GTF2H1	HACL1	HAUS4	HERC3	HINT1	HIPK3	HMGCR	HPS5
HUWE1	IDS	IFT88	IL1RAP	IMP3	INTS9	IQCB1	KATNA1	KDM3A	KDM4C	KIAA1191	KIF13A	KIF23	KLC1	KLHL12	KLHL42	KLHL5	KRIT1	LAS1L	LGALS8
LMO7	LRP8	LRRCC1	LUC7L2	MACF1	MAEA	MAGI1	MAP3K7	MAPK14	MARCHF	MARK2	MARK3	MATR3	MCCC2	MEAF6	METTL5	METTL8	MFF	MGA	MICAL1
MINDY3	MINK1	MKNK1	MLLT10	MORF4L2	MPI	MPRIP	MPZL1	MRPL21	MRPL22	MRPL32	MRPL42	MTA1	MTERF4	MTMR2	MTO1	MTRR	MYO18A	MYO9A	NABP1
NAE1	NASP	NBEAL2	NBPF15	NCAPG2	NCSTN	NDUFAF6	NDUFB5	NEMP1	NFAT5	NFKBIE	NFRKB	NGLY1	NIPA1	NIPA2	NKTR	NOP58	NPHP3	NSMCE2	NT5C2
NTHL1	NUBP1	NUDT5	NUF2	NUMB	NXPE3	OGDH	OLA1	OPA1	OPTN	OSBPL3	OSBPL6	OSBPL9	PACRGL	PAQR3	PARL	PARP11	PARP6	PATL1	PCBP2
PCCA	PCMT1	PDCD10	PDIA3	PDZK1	PHC3	PHKB	PICALM	PIMREG	PIP5K1A	PKP4	PLAA	PLEKHA1	PLSCR1	PNISR	POLR3H	PPFIBP1	PPIP5K1	PPIP5K2	PPM1K
PPP1R7	PPP2R5D	PPP3CB	PPP4R3A	PRC1	PRPF40A	PRPSAP2	PRRC2B	PSAT1	PSMA4	PTP4A2	PTPA	QDPR	RABEPK	RABGGT	RAD17	RAD54B	RALGAP	RALGPS2	RAPGEF2
RARS1	RASAL2	RBBP5	RBFOX2	RBM25	RBM26	RBM3	RBM34	RCC1	RELCH	REPS1	RER1	RFC5	RHOT1	RIPK2	RMDN1	RNF10	RNF111	RNF14	RNF41
RPAP3	RPP14	RPP30	RPS18	RPUSD3	RRAGB	RSRC2	RSRP1	RTKN2	RUBCN	RWDD1	SBNO1	SCAMP1	SDCCAG	SEC11A	SEC16A	SEC24B	SEC31A	SEC61A2	SEPT15
SEPTIN2	SF1	SF3B1	SH3TC2	SINHCAF	SLC11A2	SLC25A2	SLC30A6	SLC35G2	SLC3A2	SLC4A7	SLC50A1	SLC66A3	SLC7A2	SLIRP	SLK	SNX11	SNX4	SPATS2	SPIDR
SPIN1	SPPL2A	SRGAP2	SRRM1	SSBP3	STK26	STK3	STX2	STX3	SUGT1P1	SUMF2	SUPT20H	TACC1	TAF2	TAZ	TBC1D13	TBC1D16	TBC1D23	TBC1D3	TBPL1
TCERG1	TCF7L2	TERF1	TIA1	TLE4	TM7SF3	TMED3	TMEM12	TMEM161	TMEM214	TMEM26	TMEM263	TMEM39	TMEM45	TMX2	TNIP1	TOMM20	TOPBP1	TP53BP2	TRERF1
TRMO	TRNAU1A	TSNARE1	TSPAN5	TTC9C	TTLL5	TUT7	TWF1	UBA3	UBE2D3	UBE2K	UBP1	UCHL5	UMPS	USP37	USP4	USP8	VEZT	VPS13B	VTA1
WAC	WAPL	WASHC2	WDFY3	WDR11	WDR73	WSB1	XAF1	YBX3	YIPF1	YPEL5	ZBTB37	ZDHHC16	ZFAND1	ZNF148	ZNF207	ZNF410	ZNF473	ZNF544	ZNF655
ZNF692	AGR2	GPX3	HOXA10	CHD1	ACADS	CAPN13	CWH43	PEX11G	ZNF57	ACVRL1	ADAM15	ADIPOR1	AKNA	ALX1	AMELX	APLF	BAMBI	BCL9L	BMP5
CLASP1	CLASP2	CRB2	CUL7	DACT3	DAG1	DDX17	DLG5	EFNA1	EMP2	EOMES	EPHA3	FAM83D	FGF8	FOXF2	FUZ	GCNT2	GSC	HEY1	HEY2
HEYL	HPN	ISL1	KBTBD8	KLHL12	LDLRAD4	MARK1	MIR379	MIR519D	NKX2-1	NOLC1	OLFM1	PDCD6	PEF1	PHLDB1	PHLDB2	POFUT2	POLR1B	PPP2CA	PPP3R1
RBPJ	RFLNB	RTN4	SDCBP	SDHAF2	SERPINB	SP6	SPRED1	SPRED2	SPRED3	TASOR	TBX5	TCF7L2	TCOF1	TMEM10	USF3	VASN	WNT16	WNT2	WNT4
WNT8A	ZNF703	ZNF750																	
