# Supplementary material for: DNA methylation profiling deciphers three EMT subtypes with distinct prognoses and therapeutic vulnerabilities in breast cancer
Source: J Cancer. 2024 Jul 16;15(15):4922–38. doi: 10.7150/jca.96096 (PMC11310866; doi:10.7150/jca.96096)
Supplement: Supplementary file 1 — Supplementary methods, figures and tables. [file jcav15p4922s1.zip › Table S3.pdf]

Table S3. A total of 339 candidate methylation-EMTAGs obtained from Differential methylation analysis

FGFR1	EMILIN1	LEP	SCG5	SERPING1	S100A5	DDR2	APCDD1	SOX17	MAPK13
TMEM184A	EHF	ACVRL1	S100A8	BCAS1	EPB41L1	CBFB	IFFO1	FUT2	CDH5
ACVR1	SPARCL1	IGF1	SPDEF	FSTL1	CXCL6	PHLDB1	FAM107B	PTPRN2	XDH
DAPP1	CHST3	EOMES	KL	SYNGR2	MACF1	GYPC	SPRR1B	ESR1	EEF1A2
EFEMP2	LRRFIP1	TFF1	NDUFAF6	CTSK	EXOC6	SEMA4A	LGALS8	ANXA9	ENPP2
AJAP1	VAMP5	TUBB6	OPRM1	GAMT	LYN	CD9	CLDN4	CLEC11A	MFAP4
OGDH	CTNNAL1	LRRC15	CDX2	PDGFRB	COL5A2	HS3ST2	CX3CL1	RAB25	ARHGAP32
COL11A1	FOXA2	CPLX2	C1S	ONECUT2	NID2	LEFTY2	TAGLN	TRPM8	T
SFRP2	XAF1	KRT4	S100A14	TRIP10	GNA13	TBX20	CRMP1	CD34	SERPINB5
FCGRT	TMEM158	THBS4	TP73	WNT2	RGS17	DNAJC6	SLC52A3	LAMA1	SLC35G2
AKR1B1	MARVELD1	CDH4	LCP1	CRB2	PDPN	RAB31	SCEL	AGR2	CFTR
HHIPL2	SCARA3	MYLK	PTK6	SLIT2	FAP	EFEMP1	NUAK1	KLK3	ALX1
BNIPL	MCAM	KRT19	STC2	CYP24A1	IL23A	CLIC3	PDE1C	SYDE1	RUNX1
DLC1	CFH	SOSTDC1	TGFBR1	PRKAR1B	QRFPR	TNF	CCL18	FXYD3	SEMA7A
GIPC2	CSF2	CLIP4	ELN	KRT8	HAND1	KIF5B	COL1A2	SFRP1	CYFIP2
SRGN	LAMA2	GRIN1	RTEL1	PLAT	SCUBE3	CRP	MED15	RRAD	DSE
SGCB	CXCR2	ADIPOQ	HOXB13	EPHA10	PAM	NEUROG3	HDAC9	KCNB1	STXBP6
CALCR	NCALD	TERT	SLC2A3	CNTNAP2	CCL7	TRIM9	NRN1	DYNC1I1	NKX6-1
MMP2	HTRA3	CXCL1	PKP3	GPR180	CLDN11	NPR2	MME	BNIP3	ETV1
NES	CHST2	TOB1	TMEM100	CDKL2	ITGB6	EIF5A2	STAT5A	SULT2B1	FURIN
CBLC	IRF8	EGF	ETNK2	GALNT5	EGFR	CDH13	POMC	NR5A2	IL32
BLNK	PRICKLE2	COLEC12	NOTCH4	ALK	MMP14	NLRP3	ACTG2	COL12A1	CXCL12
A2M	LPAR1	ALX4	S100P	HYLS1	AXL	KIT	CXADR	EPB41L3	SPRR3
PPP1R14C	FCN2	ACTA1	STMN3	ELF5	SLC30A3	SREBF1	ACSS1	ABCG1	CCL2
TMPRSS2	FIP1L1	GPX7	ALOX15B	SCUBE1	STK33	PLXDC1	ASPHD1	SLC44A4	IL1B
PAMR1	IGFBP3	CNRIP1	MMP13	SMAD9	EREG	SGCD	CLEC1A	BMP2	CWH43
VCAM1	SFTPC	PTN	COL14A1	CYB5R1	SH3GL2	FAM13A	OSR1	WDR72	NTNG1
CLEC2B	SULF1	PTGER2	CCL8	ARHGDIB	TSPAN2	RUNX1T1	TCF4	LIN28A	MEOX2
PRRX2	FBLN2	MMP7	S100A9	WNT7A	NRG1	RIPK3	PLA2G7	ADAMTS2	FUT9
AKAP12	EPHA8	PRKD1	BATF2	GLT8D2	ANPEP	TSPAN8	TPM1	VCAN	ZNF746
GCNT2	PAEP	SLN	ABCG2	MOXD1	SERPINB2	NTM	EPHB1	SGCG	PCK1
WNT10B	CMTM3	NAV3	FGF2	ADAM23	PDE1A	SLC2A9	RBFOX2	ST8SIA4	RRM2
HTR2A	NR3C1	FBXO27	S100A2	LDHC	PICALM	TNFSF9	EPO	IL20RA	MYBL2
PTHLH	ADAMTSL2	KRT7	TMPRSS3	FKBP9	SEC61A2	PPARG	SPP1	CYBA	
