# Supplementary material for: DNA methylation profiling deciphers three EMT subtypes with distinct prognoses and therapeutic vulnerabilities in breast cancer
Source: J Cancer. 2024 Jul 16;15(15):4922–38. doi: 10.7150/jca.96096 (PMC11310866; doi:10.7150/jca.96096)
Supplement: Supplementary file 1 — Supplementary methods, figures and tables. [file jcav15p4922s1.zip › Table S4.pdf]

Table S4 The clinical baseline parameters among three EMT subtypes from three multi-center cohorts, including TCGA, METABRIC, and GSE96058.

Table S4A, clinical baseline characteristics among three EMT subtypes in TCGA dataset.

Characteristics	Overall (n = 1055)	EMT Clusters<br>Cluster1 (n = 386)	Cluster2 (n = 394)	Cluster3 (n = 275)	P
**Age, n (%)**					<0.001
>65	294 (27.87)	111 (28.76)	78 (19.80)	105 (38.18)	
≤65	761 (72.13)	275 (71.24)	316 (80.20)	170 (61.82)	
**Gender, n (%)**					0.006
Female	1,043 (98.86)	380 (98.45)	394 (100.00)	269 (97.82)	
Male	12 (1.14)	6 (1.55)	0 (0.00)	6 (2.18)	
**PAM50, n (%)**					<0.001
Basal	186 (17.83)	5 (1.32)	176 (44.67)	5 (1.86)	
Her2	77 (7.38)	12 (3.16)	32 (8.12)	33 (12.27)	
LumA	544 (52.16)	282 (74.21)	134 (34.01)	128 (47.58)	
LumB	197 (18.89)	79 (20.79)	15 (3.81)	103 (38.29)	
Normal	39 (3.74)	2 (0.53)	37 (9.39)	0 (0.00)	
**pStage, n (%)**					<0.001
I	177 (17.35)	79 (21.29)	70 (18.09)	28 (10.69)	
II	589 (57.75)	208 (56.06)	236 (60.98)	145 (55.34)	
III	236 (23.14)	80 (21.56)	75 (19.38)	81 (30.92)	
IV	18 (1.76)	4 (1.08)	6 (1.55)	8 (3.05)	
**pT, n (%)**					<0.001
T1	273 (25.95)	118 (30.73)	112 (28.50)	43 (15.64)	
T2	610 (57.98)	216 (56.25)	227 (57.76)	167 (60.73)	
T3	132 (12.55)	38 (9.90)	46 (11.70)	48 (17.45)	
T4	37 (3.52)	12 (3.13)	8 (2.04)	17 (6.18)	
**pN, n (%)**					0.012
N0	497 (47.88)	187 (49.21)	207 (52.81)	103 (38.72)	
N1	349 (33.62)	130 (34.21)	122 (31.12)	97 (36.47)	
N2	119 (11.46)	41 (10.79)	37 (9.44)	41 (15.41)	
N3	73 (7.03)	22 (5.79)	26 (6.63)	25 (9.40)	
**pM, n (%)**					0.039
M0	878 (97.66)	332 (98.81)	332 (97.94)	214 (95.54)	
M1	21 (2.34)	4 (1.19)	7 (2.06)	10 (4.46)	
**Race, n (%)**					<0.001

Characteristics	Overall (n = 1055)	Cluster1 (n = 386)	Cluster2 (n = 394)	Cluster3 (n = 275)	P
		**EMT Clusters**			
american indian or alaska native	1 (0.09)	0 (0.00)	1 (0.25)	0 (0.00)	
asian	57 (5.40)	20 (5.18)	21 (5.33)	16 (5.82)	
black or african american	180 (17.06)	44 (11.40)	92 (23.35)	44 (16.00)	
not reported	81 (7.68)	34 (8.81)	16 (4.06)	31 (11.27)	
white	736 (69.76)	288 (74.61)	264 (67.01)	184 (66.91)	
**Survival_status, n (%)**					0.026
Alive	906 (85.88)	343 (88.86)	339 (86.04)	224 (81.45)	
Dead	149 (14.12)	43 (11.14)	55 (13.96)	51 (18.55)	

Table S4B, clinical baseline characteristics among three EMT subtypes in METABRIC dataset.

Characteristics	Overall (n = 1979)	C1 (n = 831)	C2 (n = 727)	C3 (n = 421)	P
		**EMT Clusters**			
**Age, n (%)**					<0.001
<=65	1,160 (58.62)	417 (50.18)	514 (70.70)	229 (54.39)	
>65	819 (41.38)	414 (49.82)	213 (29.30)	192 (45.61)	
**Menospausal_status, n (%)**					<0.001
Post	1,555 (78.58)	692 (83.27)	503 (69.19)	360 (85.51)	
Pre	424 (21.42)	139 (16.73)	224 (30.81)	61 (14.49)	
**Grade, n (%)**					<0.001
1	169 (8.94)	118 (15.11)	39 (5.58)	12 (2.92)	
2	771 (40.77)	449 (57.49)	197 (28.18)	125 (30.41)	
3	951 (50.29)	214 (27.40)	463 (66.24)	274 (66.67)	
**Intclust, n (%)**					<0.001
1	139 (7.02)	30 (3.61)	22 (3.03)	87 (20.67)	
10	226 (11.42)	4 (0.48)	216 (29.71)	6 (1.43)	
2	72 (3.64)	39 (4.69)	14 (1.93)	19 (4.51)	

Characteristics	Overall (n = 1979)	EMT Clusters C1 (n = 831)	C2 (n = 727)	C3 (n = 421)	P
3	290 (14.65)	176 (21.18)	86 (11.83)	28 (6.65)	
4ER-	83 (4.19)	1 (0.12)	75 (10.32)	7 (1.66)	
4ER+	260 (13.14)	82 (9.87)	157 (21.60)	21 (4.99)	
5	190 (9.60)	13 (1.56)	86 (11.83)	91 (21.62)	
6	85 (4.30)	26 (3.13)	8 (1.10)	51 (12.11)	
7	189 (9.55)	142 (17.09)	26 (3.58)	21 (4.99)	
8	299 (15.11)	271 (32.61)	7 (0.96)	21 (4.99)	
9	146 (7.38)	47 (5.66)	30 (4.13)	69 (16.39)	
**Claudin_subtype, n (%)**					<0.001
Basal	209 (10.56)	1 (0.12)	198 (27.24)	10 (2.38)	
claudin-low	218 (11.02)	1 (0.12)	215 (29.57)	2 (0.48)	
Her2	224 (11.32)	25 (3.01)	85 (11.69)	114 (27.08)	
LumA	699 (35.32)	522 (62.82)	96 (13.20)	81 (19.24)	
LumB	475 (24.00)	242 (29.12)	40 (5.50)	193 (45.84)	
NC	6 (0.30)	5 (0.60)	0 (0.00)	1 (0.24)	
Normal	148 (7.48)	35 (4.21)	93 (12.79)	20 (4.75)	
**Histological_subtype, n (%)**					<0.001
Ductal/NST	1,490 (77.00)	599 (73.32)	542 (77.32)	349 (83.69)	
Lobular	146 (7.55)	56 (6.85)	68 (9.70)	22 (5.28)	
Medullary	25 (1.29)	0 (0.00)	23 (3.28)	2 (0.48)	
Metaplastic	2 (0.10)	0 (0.00)	2 (0.29)	0 (0.00)	
Mixed	211 (10.90)	124 (15.18)	50 (7.13)	37 (8.87)	
Mucinous	23 (1.19)	18 (2.20)	2 (0.29)	3 (0.72)	
Other	17 (0.88)	6 (0.73)	8 (1.14)	3 (0.72)	
Tubular/ cribriform	21 (1.09)	14 (1.71)	6 (0.86)	1 (0.24)	
**ER_status, n (%)**					<0.001

Characteristics	Overall (n = 1979)	EMT Clusters C1 (n = 831)	C2 (n = 727)	C3 (n = 421)	P
Negative	474 (23.95)	4 (0.48)	408 (56.12)	62 (14.73)	
Positive	1,505 (76.05)	827 (99.52)	319 (43.88)	359 (85.27)	
**HER2_status, n (%)**					<0.001
Negative	1,732 (87.52)	810 (97.47)	609 (83.77)	313 (74.35)	
Positive	247 (12.48)	21 (2.53)	118 (16.23)	108 (25.65)	
**Survival_status, n (%)**					<0.001
Died of Disease	646 (32.64)	218 (26.23)	249 (34.25)	179 (42.52)	
Died of Other Causes	496 (25.06)	267 (32.13)	119 (16.37)	110 (26.13)	
Living	837 (42.29)	346 (41.64)	359 (49.38)	132 (31.35)	
**Nottingham_prognosis_index, Mean (SD)**	4.01 (1.16)	3.59 (1.07)	4.26 (1.15)	4.42 (1.09)	<0.001

Table S4C, clinical baseline characteristics among three EMT subtypes in GSE96058 dataset.

Characteristics	Overall (n = 3273)	EMT Clusters C1 (n = 1400)	C2 (n = 1274)	C3 (n = 599)	P
**Age, n (%)**					<0.001
<=65	1,790 (54.69)	732 (52.29)	762 (59.81)	296 (49.42)	
>65	1,483 (45.31)	668 (47.71)	512 (40.19)	303 (50.58)	
**PAM50, n (%)**					<0.001
Basal	339 (10.36)	2 (0.14)	334 (26.22)	3 (0.50)	
Her2	327 (9.99)	12 (0.86)	181 (14.21)	134 (22.37)	
LumA	1,657 (50.63)	1,013 (72.36)	495 (38.85)	149 (24.87)	
LumB	729 (22.27)	361 (25.79)	68 (5.34)	300 (50.08)	
Normal	221 (6.75)	12 (0.86)	196 (15.38)	13 (2.17)	
**Nottingham_histologic_grade, n (%)**					<0.001
G1	496 (15.44)	285 (20.50)	193 (15.59)	18 (3.08)	
G2	1,532 (47.70)	838 (60.29)	487 (39.34)	207 (35.45)	
G3	1,184 (36.86)	267 (19.21)	558 (45.07)	359 (61.47)	
**ER_status, n (%)**					<0.001
Negative	241 (7.84)	3 (0.22)	214 (19.40)	24 (4.17)	
Positive	2,832 (92.16)	1,391 (99.78)	889 (80.60)	552 (95.83)	
**PR_status, n (%)**					<0.001
Negative	386 (13.13)	37 (2.73)	278 (26.30)	71 (13.40)	
Positive	2,554 (86.87)	1,316 (97.27)	779 (73.70)	459 (86.60)	
**HER2_status, n (%)**					<0.001
Negative	2,731 (86.67)	1,293 (95.92)	1,022 (83.50)	416 (71.85)	
Positive	420 (13.33)	55 (4.08)	202 (16.50)	163 (28.15)	
**Ki67_status, n (%)**					<0.001

Characteristics	Overall (n = 3273)	EMT Clusters<br>C1 (n = 1400)	 C2 (n = 1274)	 C3 (n = 599)	P
Negative	643 (41.48)	352 (52.07)	247 (40.10)	44 (17.05)	
Positive	907 (58.52)	324 (47.93)	369 (59.90)	214 (82.95)	
**Lymph_node_status, n (%)**					<0.001
Negative	2,013 (63.30)	890 (65.68)	811 (65.04)	312 (53.98)	
Positive	1,167 (36.70)	465 (34.32)	436 (34.96)	266 (46.02)	
**Survival_status, n (%)**					<0.001
Alive	2,937 (89.73)	1,284 (91.71)	1,143 (89.72)	510 (85.14)	
Dead	336 (10.27)	116 (8.29)	131 (10.28)	89 (14.86)	
