# Supplementary material for: DNA methylation profiling deciphers three EMT subtypes with distinct prognoses and therapeutic vulnerabilities in breast cancer
Source: J Cancer. 2024 Jul 16;15(15):4922–38. doi: 10.7150/jca.96096 (PMC11310866; doi:10.7150/jca.96096)
Supplement: Supplementary file 1 — Supplementary methods, figures and tables. [file jcav15p4922s1.zip › Table S5.pdf]

Table S5. The leaf genes of three subtypes derived from consensus differential analysis									
**C1 markers**									
ADAMDEC1	TRIM29	IL34	BCL2A1	SPOCK2	NKG7	GBP5	LTB	ART3	GLYATL2
CAPN6	CD3D	CSTA	JCHAIN	KRT6A	UGT8	LAD1	GAL	SLPI	CXCL11
AIF1L	PTX3	CCL5	CCL19	LYZ	KLK5	CT83	SLC6A14	CXCL10	CDCA7
MSLN	KRT17	DEFB1	CRYAB	PRAME	CCL13	KLK6	PADI2	S100A7	CDH3
HRCT1	EN1	S100B	LAMP3	INAVA	A2ML1	PKP1	CHI3L1	KRT5	MARCO
CXCL13	GZMB	SOX10	CBX2	CD79A	IDO1	HAPLN3	MMP7	KRT6B	PPP1R14C
PI3	FOXC1	STAC2	ELF5	CXCL9	FABP7	SFRP1	KRT81	RARRES1	CCL18
PSAT1	KRT16	CALML5	S100A9	GABRP	VGLL1	S100A8	FDCSP	TFF1	AGR3
ESR1	PGR	SCUBE2	TFF3	GFRA1	ANKRD30A	BMPR1B	NAT1	NPY1R	AGR2
CA12	SERPINA11	PIP	FOXA1	PTPRT	DNAJC12	TPRG1	MAPT	SUSD3	GATA3
SLC44A4	STC2	CLIC6	CHAD	DNALI1	CLSTN2	SCGB2A2	DEGS2	SLC7A2	GREB1
AKR7A3	CAPN8	MLPH	NKAIN1	AFF3	TBC1D9	GP2	AGTR1	CPB1	SYT13
ABAT	SLITRK6	PDZK1	SERPINA6	AR	SLC39A6	ZMYND10	THSD4	ERBB4	SCGB1D2
ST8SIA6	EEF1A2	KIF12	SLC27A2	KCNE4	GRIK3	MYB	UBXN10	RERG	SPDEF
PRR15	CCDC170	KCNJ3	HPN	FGF10	SLC7A8	LRG1	TMEM26	CCDC74A	CHST8
FSIP1	ELOVL2	RBM24	XBP1	REEP6	LONRF2	DIO1	GRPR	SYT9	SLC16A6
KCND3	RIMS4	TUBA3D	TTC6	PGLYRP2	ZG16B	NME5	CXCL14	CYP4X1	PARD6B
LMX1B	ADAMTS15	DACH1	TSPAN1	IL6ST	PKIB	CST9	KCNK15	VWA2	KCNJ11
C5AR2	IGFALS	TMC5	NXNL2	TMPRSS6	SYTL5	C4B	SRARP	AZGP1	ADGRB2
NEK10	C4A	NPNT	BPIFB1	ZNF552	ANXA9	INSYN2A	ADCY1	CYP2A6	RGS22
PHYHD1	RET	CST5	PTGER3	SLC40A1	BCL2	SYBU	CHRD	SCNN1A	BEX1
IGF1R	SEMA3B	TPSG1	CELSR1	RAI2	DOK7	ACADSB	GRIA2	LYPD6	FBP1
LRP2	GAMT	REPS2	WFDC2	RSPH1	SERPINA5	PREX1	ZNF703	EVL	FGD3
STMND1	RALGPS2	IL20	CYP4B1	C9orf152	FAM234B	HSPA2	C9orf116	ACOX2	TTC36
NTN4	KLHDC9	NOSTRIN	CACNG4	RLN2	PHGR1	ANKRD30B	PPP1R3C	MRPS30	C1orf21
CARTPT	RASEF	SLC1A1	SMIM22	KIAA0040	SYT17	STK32B	GLRB	ABCC8	VAV3
INPP5J	CACNA1D	CCDC74B	CACNA2D2	RHBDL1	MUC1	CYP4Z1	UGCG	PCP2	TMC4
TBX3	KCNF1	COX6C	NELL2	THPO	GASK1B	ELAPOR1	MPV17L	CAPN13	TMEM63C
LYPD6B	CCDC160	LRRC46	BMERB1	SEC14L2	FMO5	PLPPR3	SYT1	MISP	DYNLRB2
PLIN5	CELSR2	GJA1	CEACAM6	TTC39A	LRRC6	DUSP4	TUBA3E	PLAT	SLC7A4
**C2 markers**									
KITLG	RGS11	SULT2B1	SREBF1	WFS1	KIF16B	SDR16C5	BCAS4	SCCPDH	PLEKHF2
LRBA	SAMD15	SEMA3C	CCNP	CHST8	ABCA3	NECAB3	MINDY1	MEGF9	HSPA2

ASTN2	RORC	THPO	LRRC56	TOB1	TJP3	ASCL1	RAMP1	ELOVL5	RMND1
KCTD3	CDYL2	ETNK2	BHLHE40	HOXB2	CCDC125	ZBTB42	TRH	SLC22A5	RSPH1
NOSTRIN	PTK6	NOVA1	ENTPD8	KCND3	RGL3	RABEP1	TCEAL1	SEMA3F	SYTL4
C17orf58	ELP2	GSTM3	RHOB	KCNF1	CHRD	KCTD6	TMEM145	PRR36	ALCAM
EFCC1	RAB17	RBM24	PBX1	DYNLRB2	KLHDC7A	PLIN5	AZGP1	P4HTM	TESMIN
MPV17L	KDM4B	KIAA0040	STK32B	BMERB1	FAM174B	MISP3	ERBB3	TTC36	ADGRB2
LYPD6	CYP4X1	ECM1	ABCC8	MUC1	LFNG	FAM214A	EPN3	NUDT12	EVL
IGFALS	PGLYRP2	PRR15L	RARA	FMO5	SLC19A2	RTN4RL1	SYT17	MRPS30	ACOT4
ATP8B1	NXNL2	ST8SIA6	PHGR1	MACIR	NEURL1	CERS6	METRN	GALNT7	MSX2
DOK7	CGA	PPP1R3C	ARSG	BCL2	C10orf82	SLC4A8	TUBA3D	RGS22	SLC2A10
F7	ADCY1	UGCG	GRIK3	C9orf116	PLAAT2	CAPN9	CREB3L4	C4A	BPIFB2
C4B	RND1	BAIAP3	TMC4	CYP4B1	CPLX1	UBXN10	AGTR1	PCP2	FBXL16
PRRT3	PRRT1B	SMIM14	RAB30	LRRC46	PREX1	GASK1B	RAB27B	SLC9A3R1	MCCC2
MAGED2	PRLR	FGFR3	RAB26	ANKRD30B	KRT18	TMC5	WWP1	TBX3	TMEM63C
BCAM	LYPD6B	ARFGEF3	ENPP1	SRMS	CACNA1H	CST5	REEP1	DIO1	SEMA3B
REPS2	IGF1R	CXXC5	INSYN2A	PDZK1	PLPPR3	HPX	INPP4B	SYTL2	SYT1
GPRC5A	SYT9	KLHDC9	CCDC74A	RETREG1	TTC39A	RALGPS2	IL6ST	HPN	SIDT1
RASEF	TPSG1	GALNT6	ABCC11	SYBU	WNK4	ELAPOR1	TSPAN13	CACNA2D2	TOX3
GRPR	PKIB	IL20	PTPRT	ACOX2	SERPINA6	GPR160	LONRF2	SYT13	TMPRSS6
CELSR1	VAV3	NPY1R	SUSD3	STARD10	TNNT1	COX6C	HCAR1	KCNE4	SLITRK6
SMIM22	SCNN1A	PARD6B	HID1	CLGN	GAMT	STC2	CLEC3A	ABAT	INPP5J
TPRG1	SYTL5	LRG1	BPIFB1	MSMB	CCND1	ACADSB	SRARP	STMND1	GDF15
ZMYND10	KIF12	PVALB	ADAMTS15	C5AR2	RERG	TTC6	CAPN13	CACNG4	ELOVL2
ZNF552	GP2	CHAD	C9orf152	SLC7A8	KCNJ11	RIMS4	LMX1B	FAM234B	SLC27A2
MYB	REEP6	CAPN8	SCGB1D2	DHRS2	ARMT1	CMBL	CEACAM6	CST9	ZG16B
MISP	NPNT	FBP1	CCDC170	ZNF703	GREB1	KCNK15	ANXA9	PIP	SERPINA5
RET	DACH1	XBP1	BCAS1	ERBB4	PGR	CEACAM5	SLC16A6	MAPT	SCGB2A2
THSD4	ANKRD30A	SLC7A2	DNALI1	AR	AFF3	SLC39A6	FSIP1	TSPAN1	CLSTN2
NKAIN1	BMPR1B	SERPINA11	CPB1	DEGS2	DNAJC12	TBC1D9	KCNJ3	EEF1A2	AKR7A3
SCUBE2	PRR15	NAT1	SPDEF	MLPH	GATA3	CA12	SLC44A4	GFRA1	FOXA1
TFF3	AGR2	ESR1	TFF1	AGR3	FDCSP	GABRP	SFRP1	VGLL1	KRT16
SOX10	KRT5	STAC2	KRT81	KRT6B	KRT17	KLK5	PSAT1	PROM1	MMP7
PPP1R14C	ELF5	RARRES1	CHI3L1	FOXC1	PI3	SLPI	FABP7	S100B	SLC34A2
KLK6	KRT14	TRIM29	SERPINB5	EN1	CRYAB	DEFB1	KLK7	PKP1	AQP5
HAPLN3	BBOX1	FOLR1	CRABP1	S100A9	HRCT1	CHI3L2	PTX3	SLC6A14	SAA1

CAPN6	S100A8	CT83	CD79A	NCCRP1	CCL19	MSLN	LAMP3	PADI2	CALML5
CXCL13	IL34	UGT8	CDH3	FAM171A1	ART3	KCNN4	JCHAIN	ID4	LCN2
DSC3	CCL18	GSTP1	MARCO	SOSTDC1	KCNK5	CXCL1	A2ML1	GZMB	IDO1
RGMA	SYNM	EGFR	IRX1	GLYATL2	CX3CL1	ROPN1B	HORMAD1	DSG3	LTB
KRT15	ROPN1	WNT6	LY6D	CP	GAL	LTF	CXCL9	SMOC1	KRT23
KRT6A	CALB2	TTYH1	GJB3	FERMT1	KLK10	LEMD1	ACTG2	OSR1	CCL5
COL9A3	RUNX3	IL12RB2	SOX8	HLA-DOB	NKG7	SPOCK2	S100A2	SYT8	LGALS2
PLA2G2D	CCL13	CDCA7	EPHB6	SPIB	OR2I1P	CD3D	IGF2BP2	POU2AF1	MS4A1
FGFBP1	TCF7L1	GPRIN2	CD79B	PHGDH	MID1	BCL2A1	STAC	FMO2	LCK
IL7R	ITM2C	MET	WIF1	B3GNT3	CCL21	CHODL	BCL11A	CD3E	GLIPR2
GSTA1	PRAME	OLFM4	LDHB	KRT7	PM20D2	C6orf15	S100A7	B3GNT5	RASAL1
PRF1	MFGE8	LOXL4	CMTM7	VPREB3	CXCL10	CD7	FAM107A	SEL1L3	TNFRSF17
VTCN1	NFIB	ASS1	MMP12	SHC4	CD2	CHST2	DSC2	TAFA3	SH2D2A
KIT	SOD3	GBP1	CXCL2	IL2RG	MAL	KLF5	GBP5	NCMAP	FABP5
CLEC10A	KCNG1	RGS2	FZD9	CLCN4	CHST3	MAML2	PCSK1N	GZMA	SIT1
SLAMF6	GPM6B	IRF4	ADAMDEC1	CYP39A1	PCOLCE2	PLA2G4A	FSCN1	MUC15	SOX11
CHRDL1	NCOA7	CST7	LIMD2	DEPP1	FOSL1	PRKX	SBSN	LMO4	SLAMF7
UCHL1	JAK3	CPA4	CCR7	GSDMC	COTL1	IL32	CXCL11	CTSW	SAA2
MOB3B	TINAGL1	AIM2	CD6	RAB7B	RCAN1	BOC	SMCO4	CD5	MELTF
ANXA1	IL27RA	MT1M	MRAS	CD52	ANXA3	ULBP3	RHCG	PRKCQ	PDZK1IP1
LAMB3	CD38	PDE9A	WNT10A	CD247	YBX3	UNC13D	PIM1	LYZ	CSRP2
CDK6	TMEM158	INAVA	MT1X	PLAAT1	SIRPG	VSNL1	LAMC2	CR2	S1PR4
TOX	BIRC3	EDN1	PTGS2	GPR183	LGR6	GFRA3	TNNI2	CDKN2A	PLEKHF1
GABRE	ZIC1	CXCR3	TIGIT	OLFM2	CA9	TCL1A	TMEM71	CD27	DOK2
BCL11B	CD19	MTHFD1L	PLAGL1	PLCG2	ARL4C	GZMM	LAD1	CCL17	NXN
UBE2E3	TNFRSF13C	EFNA5	PRNP	KRT86	MGAT3	CSN3	IL2RA	C3	CTLA4
GNLY	ZAP70	L3MBTL4	PPP1R1B	MDFI	LBP	ALPL	CD96	CD8A	DKK1
PDCD1	CYRIA	APBA2	NKX1-2	HPDL	HLA-F	FAT2	KLHDC7B	AIF1L	SH2D1A
CCNE1	PLA2G2A	CORO1A	IKZF3	PLA2G7	PLEKHG1	OBP2B	TGFA	ST8SIA1	ANXA8L1
GPR171	SERPINE2	THEMIS2	SRSF12	SLC43A3	RASSF4				

C3 markers

CAPN6	SLC5A1	MYBPC1	WFDC2	NCCRP1	ITGB8	CMTM7	CLIC6	TMPRSS3	VTCN1
DEFB1	FOXI1	EN1	GSTP1	ELF5	CITED4	FOXC1	DSC3	PI3	CRYAB
SAA1	CRABP1	SOSTDC1	GPRIN2	ACTG2	BBOX1	SCGB3A1	LGR6	TTC22	PPP1R14C
PDZK1IP1	OBP2B	KLK6	S100B	KLK7	IRX1	FOLR1	ID4	CHI3L1	MMP7

LAMC2	VANGL2	ZBTB18	AQP5	KRT81	VGLL1	KRT15	TRIM29	KRT16	SLPI
SERPINB5	KRT6B	STAC2	KLK5	CHI3L2	KRT14	KRT17	PROM1	KRT23	KRT5
SLC34A2	SOX10	LTF	SFRP1	FDCSP	GABRP	AGR2	DHRS2	TFF3	SPDEF
AGR3	CEACAM5	FOXA1	MSMB	BCAS1	PVALB	ESR1	PRR15	MLPH	TFF1
GATA2	KCNJ3	ZIC2	TSPAN1	SLC44A4	EEF1A2				
