# Supplementary material for: DNA methylation profiling deciphers three EMT subtypes with distinct prognoses and therapeutic vulnerabilities in breast cancer
Source: J Cancer. 2024 Jul 16;15(15):4922–38. doi: 10.7150/jca.96096 (PMC11310866; doi:10.7150/jca.96096)
Supplement: Supplementary file 1 — Supplementary methods, figures and tables. [file jcav15p4922s1.zip › Table S6.pdf]

Table S6. The gene combination and corresponding occurring frequency during the 1000 iterations.	
Gene combination	Frequency
FAM171A1 ITM2C EPHB6 S100B PLCG2 PTPRT MID1 CBX2 TSPAN13 NME5 MAL CD52 CLCN4 GSTP1 VGLL1 ZBTB18 ERBB3 TTC22 SOX10 FSCN1 LMX1B SMCO4 C3 ABAT PSAT1 SEMA3F LDHB MYB MGAT3 SLC39A6 C1orf21 P4HTM GATA2 TESMIN TOX CCL17 PTGER3 LIMD2 ANXA1 RHBDL1 TOB1 PGR MISP CACNA1D HSPA2 SLC34A2 CSTA CELSR2 GPRIN2 KIAA0040 KCTD3 GPR183 IKZF3 GRIK3 PRNP SLC7A4 MOB3B KRT23 FGFBP1 KCNN4 ADGRB2 ABCC8 LRRC6 CCND1 CXCL14 DEPP1 ERBB4 SYT17 ID4 DNAJC12 YBX3 NPY1R MCCC2 TBX3 MINDY1 SOD3 SLC7A8 FGFR3 INAVA PLEKHF1 CXCL1 JCHAIN LAMC2 SEL1L3 MT1M CSRP2 RLN2 WWP1 ACOX2 IGFALS COX6C FOXI1 GRIA2 CHST8 GFRA1	67
FAM171A1 ITM2C EPHB6 S100B PLCG2 PTPRT MID1 CBX2 TSPAN13 NME5 MAL CD52 CLCN4 GSTP1 VGLL1 ZBTB18 ERBB3 TTC22 SOX10 FSCN1 LMX1B SMCO4 C3 ABAT PSAT1 SEMA3F LDHB MYB MGAT3 SLC39A6 C1orf21 P4HTM GATA2 TESMIN TOX CCL17 PTGER3 LIMD2 ANXA1 RHBDL1 TOB1 PGR MISP CACNA1D HSPA2 SLC34A2 CSTA CELSR2 GPRIN2 KIAA0040 KCTD3 GPR183 IKZF3 GRIK3 PRNP SLC7A4 MOB3B KRT23 FGFBP1 KCNN4 ADGRB2 ABCC8 LRRC6 CCND1 CXCL14 DEPP1 ERBB4 SYT17 ID4 DNAJC12 YBX3 NPY1R MCCC2 TBX3 MINDY1 SOD3 SLC7A8 FGFR3 INAVA PLEKHF1 CXCL1 JCHAIN LAMC2 SEL1L3 MT1M KRT18 CSRP2 AQP5 RLN2 WWP1 ACOX2 IGFALS COX6C FOXI1 GRIA2 CHST8 GFRA1 AGTR1	63
FAM171A1 CHST2 ITM2C EPHB6 S100B PLCG2 PTPRT MID1 CBX2 TSPAN13 NME5 MAL CD52 CLCN4 GSTP1 VGLL1 ZBTB18 ERBB3 SOX10 FSCN1 LMX1B SMCO4 EPN3 C3 ABAT PSAT1 SEMA3F LDHB MYB MGAT3 SLC39A6 C1orf21 P4HTM GATA2 TESMIN TOX CCL17 PTGER3 LIMD2 ANXA1 RHBDL1 TOB1 PGR MISP CACNA1D HSPA2 SLC34A2 CSTA CELSR2 GPRIN2 KIAA0040 KCTD3 GPR183 IKZF3 GRIK3 PRNP SLC7A4 MOB3B KRT23 FGFBP1 KCNN4 ADGRB2 ABCC8 LRRC6 CCND1 CXCL14 DEPP1 ERBB4 SYT17 ID4 DNAJC12 BBOX1 YBX3 NPY1R MCCC2 TBX3 MINDY1 SOD3 SLC7A8 FGFR3 INAVA PLEKHF1 CHI3L2 CXCL1 JCHAIN LAMC2 SEL1L3 PI3 MT1M KRT18 CSRP2 AQP5 RLN2 WWP1 ACOX2 IGFALS COX6C FOXI1 GRIA2 CHST8 GFRA1 AGTR1	61
FAM171A1 CHST2 ITM2C EPHB6 S100B PLCG2 PTPRT MID1 CBX2 TSPAN13 NME5 MAL CD52 CLCN4 GSTP1 VGLL1 ZBTB18 ERBB3 TTC22 SOX10 FSCN1 LMX1B SMCO4 C3 ABAT PSAT1 SEMA3F LDHB MYB MGAT3 SLC39A6 C1orf21 P4HTM GATA2 TESMIN TOX CCL17 PTGER3 LIMD2 ANXA1 RHBDL1 TOB1 PGR MISP CACNA1D HSPA2 SLC34A2 CSTA CELSR2 GPRIN2 KIAA0040 KCTD3 GPR183 IKZF3 GRIK3 PRNP SLC7A4 MOB3B KRT23 FGFBP1 KCNN4 ADGRB2 ABCC8 LRRC6 CCND1 CXCL14 DEPP1 ERBB4 SYT17 ID4 DNAJC12 YBX3 NPY1R MCCC2 TBX3 MINDY1 SOD3 SLC7A8 FGFR3 INAVA PLEKHF1 CHI3L2 CXCL1 JCHAIN LAMC2 SEL1L3 PI3 MT1M KRT18 CSRP2 AQP5 RLN2 WWP1 ACOX2 IGFALS COX6C FOXI1 GRIA2 CHST8 GFRA1 AGTR1	60

FAM171A1 ITM2C EPHB6 S100B PLCG2 PTPRT MID1 CBX2 TSPAN13 NME5 MAL CD52 CLCN4 GSTP1 VGLL1 ZBTB18 ERBB3 TTC22 SOX10 FSCN1 LMX1B SMCO4 C3 ABAT PSAT1 SEMA3F LDHB MYB MGAT3 SLC39A6 C1orf21 P4HTM GATA2 TESMIN TOX CCL17 PTGER3 LIMD2 ANXA1 RHBDL1 TOB1 PGR MISP CACNA1D HSPA2 SLC34A2 CSTA CELSR2 GPRIN2 KIAA0040 KCTD3 GPR183 IKZF3 GRIK3 PRNP SLC7A4 MOB3B KRT23 FGFBP1 KCNN4 ADGRB2 ABCC8 LRRC6 CCND1 CXCL14 DEPP1 SYT17 ID4 DNAJC12 YBX3 NPY1R MCCC2 TBX3 MINDY1 SOD3 SLC7A8 FGFR3 INAVA PLEKHF1 CXCL1 JCHAIN LAMC2 MT1M CSRP2 RLN2 WWP1 ACOX2 IGFALS COX6C FOXI1 GRIA2 CHST8 GFRA1 AGTR1	58
FAM171A1 CHST2 ITM2C EPHB6 S100B PLCG2 PTPRT MID1 CBX2 TSPAN13 NME5 MAL CD52 CLCN4 GSTP1 VGLL1 ZBTB18 ERBB3 SOX10 FSCN1 LMX1B SMCO4 EPN3 C3 ABAT PSAT1 SEMA3F LDHB MYB MGAT3 SLC39A6 C1orf21 P4HTM GATA2 TESMIN TOX CCL17 PTGER3 LIMD2 ANXA1 RHBDL1 TOB1 CA12 PGR MISP CACNA1D HSPA2 SLC34A2 CSTA CELSR2 GPRIN2 KIAA0040 KCTD3 GPR183 IKZF3 GRIK3 PRNP SLC7A4 MOB3B KRT23 FGFBP1 KCNN4 ADGRB2 ABCC8 LRRC6 CCND1 CXCL14 DEPP1 ERBB4 SYT17 ID4 DNAJC12 BBOX1 YBX3 NPY1R MCCC2 TBX3 MINDY1 SOD3 SLC7A8 FGFR3 INAVA PLEKHF1 CHI3L2 CXCL1 JCHAIN LAMC2 SEL1L3 PI3 MT1M KRT18 CSRP2 AQP5 RLN2 WWP1 ACOX2 IGFALS COX6C FOXI1 GRIA2 CHST8 GFRA1 AGTR1 RMND1 FAM107A	57
FAM171A1 ITM2C EPHB6 S100B PLCG2 PTPRT MID1 CBX2 TSPAN13 NME5 MAL CD52 CLCN4 GSTP1 VGLL1 ZBTB18 ERBB3 TTC22 SOX10 FSCN1 LMX1B SMCO4 EPN3 C3 ABAT PSAT1 SEMA3F LDHB MGAT3 SLC39A6 C1orf21 P4HTM GATA2 TESMIN TOX CCL17 PTGER3 LIMD2 ANXA1 RHBDL1 TOB1 PGR MISP CACNA1D HSPA2 SLC34A2 CSTA CELSR2 GPRIN2 KIAA0040 KCTD3 GPR183 IKZF3 GRIK3 PRNP SLC7A4 MOB3B KRT23 FGFBP1 KCNN4 ADGRB2 ABCC8 LRRC6 CCND1 CXCL14 DEPP1 SYT17 ID4 DNAJC12 YBX3 NPY1R MCCC2 TBX3 MINDY1 SOD3 SLC7A8 FGFR3 INAVA PLEKHF1 CXCL1 JCHAIN LAMC2 MT1M CSRP2 RLN2 ACOX2 IGFALS COX6C FOXI1 GRIA2 CHST8 GFRA1 AGTR1	53
FAM171A1 ITM2C EPHB6 S100B PLCG2 PTPRT MID1 CBX2 TSPAN13 NME5 MAL CD52 CLCN4 GSTP1 VGLL1 ZBTB18 ERBB3 TTC22 SOX10 FSCN1 LMX1B SMCO4 EPN3 C3 ABAT PSAT1 SEMA3F LDHB MGAT3 SLC39A6 C1orf21 P4HTM GATA2 TESMIN TOX CCL17 PTGER3 LIMD2 ANXA1 RHBDL1 TOB1 MISP CACNA1D HSPA2 SLC34A2 CSTA CELSR2 GPRIN2 KIAA0040 KCTD3 GPR183 IKZF3 GRIK3 PRNP SLC7A4 MOB3B KRT23 KCNN4 ADGRB2 ABCC8 LRRC6 CCND1 CXCL14 DEPP1 SYT17 ID4 DNAJC12 YBX3 NPY1R TBX3 MINDY1 SLC7A8 FGFR3 PLEKHF1 CXCL1 JCHAIN LAMC2 MT1M RLN2 IGFALS COX6C FOXI1 GRIA2 CHST8 GFRA1 AGTR1	45

FAM171A1 CHST2 ITM2C EPHB6 S100B PLCG2 PTPRT MID1 CBX2 TSPAN13 NME5 MAL CD52 CLCN4 GSTP1 VGLL1 ZBTB18 SLC22A5 ERBB3 PIM1 SOX10 FSCN1 LMX1B SMCO4 EPN3 C3 ABAT PSAT1 SEMA3F LDHB MYB MGAT3 SLC39A6 C1orf21 P4HTM GATA2 TESMIN TOX CCL17 PTGER3 LIMD2 ANXA1 RHBDL1 TOB1 CA12 PGR MISP CACNA1D HSPA2 SLC34A2 CSTA CELSR2 HPN GPRIN2 KIAA0040 KCTD3 GPR183 IKZF3 GRIK3 PRNP SLC7A4 MOB3B KRT23 FGFBP1 KCNN4 ADGRB2 ABCC8 LRRC6 CCND1 CXCL14 DEPP1 ERBB4 SYT17 ID4 DNAJC12 AR BBOX1 YBX3 NPY1R MCCC2 DNALI1 TBX3 MINDY1 ITGB8 SOD3 SLC7A8 FGFR3 RALGPS2 INAVA PLEKHF1 CHI3L2 CXCL1 JCHAIN LAMC2 SEL1L3 PI3 MT1M KRT18 CSRP2 AQP5 RLN2 WWP1 ACOX2 IGFALS COX6C FOXI1 GLRB GRIA2 CHST8 GFRA1 AGTR1 RMND1 FAM107A	40
FAM171A1 CHST2 ITM2C EPHB6 S100B PLCG2 PTPRT MID1 CBX2 TSPAN13 NME5 MAL CD52 CLCN4 GSTP1 VGLL1 ZBTB18 SLC22A5 ERBB3 PIM1 SOX10 FSCN1 LMX1B SMCO4 EPN3 C3 ABAT PSAT1 SEMA3F LDHB MYB MGAT3 SLC39A6 C1orf21 P4HTM GATA2 TESMIN TOX CCL17 PTGER3 LIMD2 ANXA1 RHBDL1 TOB1 CA12 PGR MISP CACNA1D HSPA2 SLC34A2 CSTA CELSR2 HPN GPRIN2 KIAA0040 KCTD3 GPR183 IKZF3 GRIK3 PRNP SLC7A4 MOB3B KRT23 FGFBP1 KCNN4 ADGRB2 ABCC8 LRRC6 CCND1 CXCL14 DEPP1 ERBB4 SYT17 ID4 DNAJC12 AR BBOX1 YBX3 NPY1R MCCC2 TBX3 MINDY1 ITGB8 SOD3 SLC7A8 FGFR3 RALGPS2 INAVA PLEKHF1 CHI3L2 CXCL1 JCHAIN LAMC2 SEL1L3 PI3 MT1M KRT18 CSRP2 AQP5 RLN2 WWP1 ACOX2 IGFALS COX6C FOXI1 GLRB GRIA2 CHST8 GFRA1 AGTR1 RMND1 FAM107A	39
FAM171A1 ITM2C EPHB6 S100B PLCG2 PTPRT MID1 CBX2 TSPAN13 NME5 MAL CD52 CLCN4 GSTP1 VGLL1 ZBTB18 ERBB3 TTC22 SOX10 FSCN1 LMX1B SMCO4 EPN3 C3 ABAT PSAT1 SEMA3F LDHB MGAT3 SLC39A6 C1orf21 P4HTM GATA2 TESMIN TOX CCL17 PTGER3 LIMD2 ANXA1 RHBDL1 TOB1 PGR MISP CACNA1D HSPA2 SLC34A2 CSTA CELSR2 GPRIN2 KIAA0040 KCTD3 GPR183 IKZF3 GRIK3 PRNP SLC7A4 MOB3B KRT23 FGFBP1 KCNN4 ADGRB2 ABCC8 LRRC6 CCND1 CXCL14 DEPP1 SYT17 ID4 DNAJC12 YBX3 NPY1R MCCC2 TBX3 MINDY1 SOD3 SLC7A8 FGFR3 INAVA PLEKHF1 CXCL1 JCHAIN LAMC2 MT1M CSRP2 RLN2 IGFALS COX6C FOXI1 GRIA2 CHST8 GFRA1 AGTR1	37
FAM171A1 CHST2 BCL11A ITM2C HLA_DOB EPHB6 S100B PLCG2 PTPRT MID1 CBX2 TSPAN13 NME5 MAL CD52 CLCN4 GSTP1 VGLL1 ZBTB18 SLC22A5 ERBB3 PIM1 SOX10 SPIB FSCN1 LMX1B SMCO4 EPN3 C3 ABAT PSAT1 SEMA3F LDHB MYB MGAT3 SLC39A6 C1orf21 P4HTM GATA2 TESMIN TOX CCL17 PTGER3 LIMD2 THEMIS2 ANXA1 RHBDL1 TOB1 CA12 PGR MISP CACNA1D HSPA2 SLC34A2 CSTA CELSR2 HPN GPRIN2 KIAA0040 KCTD3 IKZF3 GRIK3 PRNP SLC7A4 MOB3B KRT23 FGFBP1 KCNN4 ADGRB2 ABCC8 LRRC6 CCND1 CXCL14 DEPP1 ERBB4 SYT17 ID4 DNAJC12 AR AGR2 BBOX1 YBX3 NPY1R MCCC2 TBX3 MINDY1 ITGB8 SLC7A8 FGFR3 RALGPS2 INAVA PLEKHF1 CHI3L2 CXCL1 JCHAIN LAMC2 SEL1L3 PI3 MT1M KRT18 CSRP2 AQP5 RLN2 WWP1 ACOX2 IGFALS COX6C FOXI1 PRKX GLRB GRIA2 CHST8 GFRA1 AGTR1 RMND1 FAM107A	35

FAM171A1 ITM2C EPHB6 S100B PLCG2 PTPRT MID1 CBX2 TSPAN13 NME5 MAL CD52 CLCN4 GSTP1 VGLL1 ZBTB18 ERBB3 TTC22 SOX10 FSCN1 LMX1B SMCO4 EPN3 C3 ABAT PSAT1 SEMA3F LDHB MGAT3 SLC39A6 C1orf21 P4HTM GATA2 TESMIN TOX CCL17 PTGER3 LIMD2 ANXA1 RHBDL1 TOB1 PGR MISP CACNA1D HSPA2 SLC34A2 CSTA CELSR2 GPRIN2 KIAA0040 KCTD3 GPR183 IKZF3 GRIK3 PRNP SLC7A4 MOB3B KRT23 KCNN4 ADGRB2 ABCC8 LRRC6 CCND1 CXCL14 DEPP1 SYT17 ID4 DNAJC12 YBX3 NPY1R MCCC2 TBX3 MINDY1 SOD3 SLC7A8 FGFR3 INAVA PLEKHF1 CXCL1 JCHAIN LAMC2 MT1M CSRP2 RLN2 IGFALS COX6C FOXI1 GRIA2 CHST8 GFRA1 AGTR1	35
FAM171A1 CHST2 BCL11A ITM2C HLA_DOB EPHB6 S100B PLCG2 PTPRT MID1 CBX2 TSPAN13 NME5 MAL CD52 CLCN4 GSTP1 VGLL1 ZBTB18 SLC22A5 ERBB3 PIM1 SOX10 SPIB FSCN1 LMX1B SMCO4 EPN3 C3 ABAT PSAT1 SEMA3F LDHB MYB MGAT3 SLC39A6 C1orf21 P4HTM GATA2 TESMIN TOX CCL17 PTGER3 LIMD2 ANXA1 RHBDL1 TOB1 CA12 PGR MISP CACNA1D HSPA2 SLC34A2 CSTA CELSR2 HPN GPRIN2 KIAA0040 KCTD3 IKZF3 GRIK3 PRNP SLC7A4 MOB3B KRT23 FGFBP1 KCNN4 ADGRB2 ABCC8 LRRC6 CCND1 CXCL14 DEPP1 ERBB4 SYT17 ID4 DNAJC12 AR AGR2 BBOX1 YBX3 NPY1R MCCC2 TBX3 MINDY1 ITGB8 SLC7A8 FGFR3 RALGPS2 INAVA PLEKHF1 CHI3L2 CXCL1 JCHAIN LAMC2 SEL1L3 PI3 MT1M KRT18 CSRP2 AQP5 RLN2 WWP1 ACOX2 IGFALS COX6C FOXI1 PRKX GLRB GRIA2 CHST8 GFRA1 AGTR1 RMND1 FAM107A	34
FAM171A1 CHST2 ITM2C HLA_DOB EPHB6 S100B PLCG2 PTPRT MID1 CBX2 TSPAN13 NME5 MAL CD52 CLCN4 GSTP1 VGLL1 ZBTB18 SLC22A5 ERBB3 PIM1 SOX10 SPIB FSCN1 LMX1B SMCO4 EPN3 C3 ABAT PSAT1 SEMA3F LDHB MYB MGAT3 SLC39A6 C1orf21 P4HTM GATA2 TESMIN TOX CCL17 PTGER3 LIMD2 ANXA1 RHBDL1 TOB1 CA12 PGR MISP CACNA1D HSPA2 SLC34A2 CSTA CELSR2 HPN GPRIN2 KIAA0040 KCTD3 GPR183 IKZF3 GRIK3 PRNP SLC7A4 MOB3B KRT23 FGFBP1 KCNN4 ADGRB2 ABCC8 LRRC6 CCND1 CXCL14 DEPP1 ERBB4 SYT17 ID4 DNAJC12 AR AGR2 BBOX1 YBX3 NPY1R MCCC2 TBX3 MINDY1 ITGB8 SOD3 SLC7A8 FGFR3 RALGPS2 INAVA PLEKHF1 CHI3L2 CXCL1 JCHAIN LAMC2 SEL1L3 PI3 MT1M KRT18 CSRP2 AQP5 RLN2 WWP1 ACOX2 IGFALS COX6C FOXI1 PRKX GLRB GRIA2 CHST8 GFRA1 AGTR1 RMND1 FAM107A	34
FAM171A1 CHST2 ITM2C EPHB6 S100B PLCG2 PTPRT MID1 CBX2 TSPAN13 NME5 MAL CD52 CLCN4 GSTP1 VGLL1 ZBTB18 SLC22A5 ERBB3 PIM1 SOX10 SPIB FSCN1 LMX1B SMCO4 EPN3 C3 ABAT PSAT1 SEMA3F LDHB MYB MGAT3 SLC39A6 C1orf21 P4HTM GATA2 TESMIN TOX CCL17 PTGER3 LIMD2 ANXA1 RHBDL1 TOB1 CA12 PGR MISP CACNA1D HSPA2 SLC34A2 CSTA CELSR2 HPN GPRIN2 KIAA0040 KCTD3 GPR183 IKZF3 GRIK3 PRNP SLC7A4 MOB3B KRT23 FGFBP1 KCNN4 ADGRB2 ABCC8 LRRC6 CCND1 CXCL14 DEPP1 ERBB4 SYT17 ID4 DNAJC12 AR BBOX1 YBX3 NPY1R MCCC2 DNALI1 TBX3 MINDY1 ITGB8 SOD3 SLC7A8 FGFR3 RALGPS2 INAVA PLEKHF1 CHI3L2 CXCL1 JCHAIN LAMC2 SEL1L3 PI3 MT1M KRT18 CSRP2 AQP5 RLN2 WWP1 ACOX2 IGFALS COX6C FOXI1 PRKX GLRB GRIA2 CHST8 GFRA1 AGTR1 RMND1 FAM107A	32

FAM171A1 ITM2C EPHB6 S100B PLCG2 PTPRT MID1 CBX2 TSPAN13 NME5 MAL CD52 CLCN4 GSTP1 VGLL1 ZBTB18 ERBB3 TTC22 SOX10 FSCN1 LMX1B SMCO4 EPN3 C3 ABAT PSAT1 SEMA3F LDHB MGAT3 SLC39A6 C1orf21 P4HTM GATA2 TESMIN TOX CCL17 PTGER3 LIMD2 ANXA1 RHBDL1 TOB1 MISP CACNA1D HSPA2 SLC34A2 CSTA CELSR2 GPRIN2 KIAA0040 KCTD3 GPR183 IKZF3 GRIK3 PRNP SLC7A4 MOB3B KRT23 KCNN4 ADGRB2 ABCC8 LRRC6 CCND1 CXCL14 DEPP1 SYT17 ID4 DNAJC12 YBX3 NPY1R MCCC2 TBX3 MINDY1 SLC7A8 FGFR3 INAVA PLEKHF1 CXCL1 JCHAIN LAMC2 MT1M CSRP2 RLN2 IGFALS COX6C FOXI1 GRIA2 CHST8 GFRA1 AGTR1	32
FAM171A1 ITM2C EPHB6 S100B PLCG2 PTPRT MID1 CBX2 TSPAN13 NME5 MAL CD52 CLCN4 GSTP1 VGLL1 ZBTB18 ERBB3 TTC22 SOX10 FSCN1 LMX1B SMCO4 EPN3 C3 ABAT PSAT1 SEMA3F LDHB MGAT3 SLC39A6 C1orf21 P4HTM GATA2 TESMIN TOX CCL17 PTGER3 LIMD2 ANXA1 RHBDL1 TOB1 MISP CACNA1D HSPA2 SLC34A2 CSTA CELSR2 GPRIN2 KIAA0040 KCTD3 GPR183 IKZF3 GRIK3 PRNP SLC7A4 MOB3B KRT23 KCNN4 ADGRB2 ABCC8 LRRC6 CCND1 CXCL14 DEPP1 SYT17 ID4 DNAJC12 YBX3 NPY1R MCCC2 TBX3 MINDY1 SOD3 SLC7A8 FGFR3 INAVA PLEKHF1 CXCL1 JCHAIN LAMC2 MT1M CSRP2 RLN2 IGFALS COX6C FOXI1 GRIA2 CHST8 GFRA1 AGTR1	31
FAM171A1 CHST2 BCL11A ITM2C RUNX3 HLA_DOB EPHB6 S100B PLCG2 PTPRT MID1 CBX2 TSPAN13 NME5 MAL CD52 CLCN4 GSTP1 VGLL1 ZBTB18 SLC22A5 ERBB3 SPDEF CHODL PIM1 SOX10 SPIB FSCN1 LMX1B SMCO4 EPN3 C3 ABAT KCND3 PSAT1 SEMA3F WNT6 LDHB MYB SLC16A6 MGAT3 LAMP3 SLC39A6 PLA2G4A C1orf21 P4HTM GATA2 TESMIN TOX CCL17 PTGER3 LIMD2 THEMIS2 ANXA1 RHBDL1 TOB1 CA12 PGR MISP CACNA1D HSPA2 SLC34A2 CSTA CELSR2 HPN GPRIN2 KIAA0040 KCTD3 IKZF3 GRIK3 PRNP SLC7A4 MOB3B KRT23 FGFBP1 KCNN4 ADGRB2 ABCC8 LRRC6 CCND1 CXCL14 DEPP1 ERBB4 SYT17 ID4 ZNF552 DNAJC12 AR AGR2 BBOX1 YBX3 NPY1R MCCC2 TBX3 MINDY1 ITGB8 SLC7A8 FGFR3 RALGPS2 INAVA PLEKHF1 CHI3L2 CXCL1 JCHAIN LAMC2 SEL1L3 CHRDL1 FABP7 PI3 MT1M KRT18 CSRP2 AQP5 RLN2 WWP1 TINAGL1 ACOX2 IGFALS COX6C FOXI1 PRKX GLRB PLA2G7 GRIA2 CHST8 GFRA1 KLK10 AGTR1 RMND1 FAM107A	26
FAM171A1 CHST2 BCL11A ITM2C HLA_DOB EPHB6 S100B PLCG2 PTPRT MID1 CBX2 TSPAN13 NME5 MAL CD52 CLCN4 GSTP1 VGLL1 ZBTB18 SLC22A5 ERBB3 PIM1 SOX10 SPIB FSCN1 LMX1B SMCO4 EPN3 C3 ABAT KCND3 PSAT1 SEMA3F WNT6 LDHB MYB SLC16A6 MGAT3 SLC39A6 C1orf21 P4HTM GATA2 TESMIN TOX CCL17 PTGER3 LIMD2 THEMIS2 ANXA1 RHBDL1 TOB1 CA12 PGR MISP CACNA1D HSPA2 SLC34A2 CSTA CELSR2 HPN GPRIN2 KIAA0040 KCTD3 IKZF3 GRIK3 PRNP SLC7A4 MOB3B KRT23 FGFBP1 KCNN4 ADGRB2 ABCC8 LRRC6 CCND1 CXCL14 DEPP1 ERBB4 SYT17 ID4 DNAJC12 AR AGR2 BBOX1 YBX3 NPY1R MCCC2 TBX3 MINDY1 ITGB8 SLC7A8 FGFR3 RALGPS2 INAVA PLEKHF1 CHI3L2 CXCL1 JCHAIN LAMC2 SEL1L3 PI3 MT1M KRT18 CSRP2 AQP5 RLN2 WWP1 TINAGL1 ACOX2 IGFALS COX6C FOXI1 PRKX GLRB GRIA2 CHST8 GFRA1 KLK10 AGTR1 RMND1 FAM107A	22

FAM171A1 CHST2 BCL11A ITM2C HLA_DOB EPHB6 S100B PLCG2 PTPRT MID1 CBX2 TSPAN13 NME5 MAL CD52 CLCN4 GSTP1 VGLL1 ZBTB18 SLC22A5 ERBB3 PIM1 SOX10 SPIB FSCN1 LMX1B SMCO4 EPN3 C3 ABAT PSAT1 SEMA3F LDHB MYB SLC16A6 MGAT3 SLC39A6 C1orf21 P4HTM GATA2 TESMIN TOX CCL17 PTGER3 LIMD2 THEMIS2 ANXA1 RHBDL1 TOB1 CA12 PGR MISP CACNA1D HSPA2 SLC34A2 CSTA CELSR2 HPN GPRIN2 KIAA0040 KCTD3 IKZF3 GRIK3 PRNP SLC7A4 MOB3B KRT23 FGFBP1 KCNN4 ADGRB2 ABCC8 LRRC6 CCND1 CXCL14 DEPP1 ERBB4 SYT17 ID4 DNAJC12 AR AGR2 BBOX1 YBX3 NPY1R MCCC2 TBX3 MINDY1 ITGB8 SLC7A8 FGFR3 RALGPS2 INAVA PLEKHF1 CHI3L2 CXCL1 JCHAIN LAMC2 SEL1L3 PI3 MT1M KRT18 CSRP2 AQP5 RLN2 WWP1 ACOX2 IGFALS COX6C FOXI1 PRKX GLRB GRIA2 CHST8 GFRA1 KLK10 AGTR1 RMND1 FAM107A	22
FAM171A1 CHST2 BCL11A ITM2C RUNX3 HLA_DOB EPHB6 S100B PLCG2 PTPRT MID1 CBX2 TSPAN13 NME5 MAL CD52 CLCN4 GSTP1 VGLL1 ZBTB18 SLC22A5 ERBB3 SPDEF CHODL PIM1 SOX10 SPIB FSCN1 LMX1B SMCO4 EPN3 C3 ABAT KCND3 PSAT1 SEMA3F WNT6 LDHB MYB SLC16A6 MGAT3 LAMP3 SLC39A6 PLA2G4A C1orf21 P4HTM GATA2 TESMIN TOX CCL17 PTGER3 LIMD2 THEMIS2 ANXA1 RHBDL1 TOB1 CA12 PGR MISP CACNA1D HSPA2 SLC34A2 CSTA CELSR2 HPN GPRIN2 KIAA0040 KCTD3 IKZF3 GRIK3 PRNP SLC7A4 MOB3B KRT23 FGFBP1 KCNN4 ADGRB2 ABCC8 LRRC6 CCND1 CXCL14 DEPP1 ERBB4 SYT17 ID4 ZNF552 DNAJC12 AR AGR2 BBOX1 YBX3 NPY1R MCCC2 TBX3 MINDY1 ITGB8 SLC7A8 FGFR3 RALGPS2 INAVA PLEKHF1 CHI3L2 CXCL1 JCHAIN LAMC2 SEL1L3 CHRDL1 PI3 MT1M KRT18 CSRP2 AQP5 RLN2 WWP1 TINAGL1 ACOX2 IGFALS COX6C FOXI1 PRKX GLRB GRIA2 CHST8 GFRA1 KLK10 AGTR1 RMND1 FAM107A	20
FAM171A1 CHST2 BCL11A ITM2C HLA_DOB EPHB6 S100B PLCG2 PTPRT MID1 CBX2 TSPAN13 NME5 MAL CD52 CLCN4 GSTP1 VGLL1 ZBTB18 SLC22A5 ERBB3 PIM1 SOX10 SPIB FSCN1 LMX1B SMCO4 EPN3 C3 ABAT PSAT1 SEMA3F WNT6 LDHB MYB SLC16A6 MGAT3 SLC39A6 C1orf21 P4HTM GATA2 TESMIN TOX CCL17 PTGER3 LIMD2 THEMIS2 ANXA1 RHBDL1 TOB1 CA12 PGR MISP CACNA1D HSPA2 SLC34A2 CSTA CELSR2 HPN GPRIN2 KIAA0040 KCTD3 IKZF3 GRIK3 PRNP SLC7A4 MOB3B KRT23 FGFBP1 KCNN4 ADGRB2 ABCC8 LRRC6 CCND1 CXCL14 DEPP1 ERBB4 SYT17 ID4 DNAJC12 AR AGR2 BBOX1 YBX3 NPY1R MCCC2 TBX3 MINDY1 ITGB8 SLC7A8 FGFR3 RALGPS2 INAVA PLEKHF1 CHI3L2 CXCL1 JCHAIN LAMC2 SEL1L3 PI3 MT1M KRT18 CSRP2 AQP5 RLN2 WWP1 ACOX2 IGFALS COX6C FOXI1 PRKX GLRB GRIA2 CHST8 GFRA1 KLK10 AGTR1 RMND1 FAM107A	19
FAM171A1 ITM2C EPHB6 S100B PLCG2 PTPRT MID1 CBX2 TSPAN13 NME5 MAL CD52 CLCN4 GSTP1 VGLL1 ZBTB18 ERBB3 TTC22 SOX10 FSCN1 LMX1B SMCO4 EPN3 C3 ABAT PSAT1 SEMA3F LDHB MGAT3 SLC39A6 C1orf21 P4HTM GATA2 TESMIN TOX CCL17 PTGER3 LIMD2 ANXA1 RHBDL1 TOB1 MISP CACNA1D HSPA2 SLC34A2 CSTA CELSR2 GPRIN2 KIAA0040 KCTD3 GPR183 IKZF3 GRIK3 PRNP SLC7A4 MOB3B KRT23 KCNN4 ADGRB2 ABCC8 LRRC6 CCND1 CXCL14 DEPP1 SYT17 ID4 DNAJC12 YBX3 NPY1R MCCC2 TBX3 MINDY1 SLC7A8 FGFR3 INAVA PLEKHF1 CXCL1 JCHAIN LAMC2 MT1M RLN2 IGFALS COX6C FOXI1 GRIA2 CHST8 GFRA1 AGTR1	19

FAM171A1 CHST2 BCL11A ITM2C HLA_DOB EPHB6 S100B PLCG2 PTPRT MID1 CBX2 TSPAN13 NME5 MAL CD52 CLCN4 GSTP1 VGLL1 ZBTB18 SLC22A5 ERBB3 SPDEF PIM1 SOX10 SPIB FSCN1 LMX1B SMCO4 EPN3 C3 ABAT KCND3 PSAT1 SEMA3F WNT6 LDHB MYB SLC16A6 MGAT3 LAMP3 SLC39A6 C1orf21 P4HTM GATA2 TESMIN TOX CCL17 PTGER3 LIMD2 THEMIS2 ANXA1 RHBDL1 TOB1 CA12 PGR MISP CACNA1D HSPA2 SLC34A2 CSTA CELSR2 HPN GPRIN2 KIAA0040 KCTD3 IKZF3 GRIK3 PRNP SLC7A4 MOB3B KRT23 FGFBP1 KCNN4 ADGRB2 ABCC8 LRRC6 CCND1 CXCL14 DEPP1 ERBB4 SYT17 ID4 DNAJC12 AR AGR2 BBOX1 YBX3 NPY1R MCCC2 TBX3 MINDY1 ITGB8 SLC7A8 FGFR3 RALGPS2 INAVA PLEKHF1 CHI3L2 CXCL1 JCHAIN LAMC2 SEL1L3 CHRDL1 PI3 MT1M KRT18 CSRP2 AQP5 RLN2 WWP1 TINAGL1 ACOX2 IGFALS COX6C FOXI1 PRKX GLRB GRIA2 CHST8 GFRA1 KLK10 AGTR1 RMND1 FAM107A	18
FAM171A1 ITM2C EPHB6 S100B PLCG2 PTPRT MID1 CBX2 TSPAN13 NME5 MAL CD52 CLCN4 GSTP1 VGLL1 ZBTB18 ERBB3 TTC22 SOX10 FSCN1 LMX1B SMCO4 EPN3 C3 ABAT PSAT1 SEMA3F LDHB MGAT3 SLC39A6 C1orf21 P4HTM GATA2 TESMIN TOX CCL17 PTGER3 LIMD2 ANXA1 RHBDL1 TOB1 MISP CACNA1D HSPA2 SLC34A2 CSTA CELSR2 GPRIN2 KIAA0040 KCTD3 GPR183 IKZF3 GRIK3 PRNP SLC7A4 MOB3B KRT23 KCNN4 ADGRB2 ABCC8 LRRC6 CCND1 CXCL14 DEPP1 SYT17 ID4 DNAJC12 YBX3 NPY1R TBX3 SLC7A8 FGFR3 PLEKHF1 CXCL1 JCHAIN LAMC2 MT1M RLN2 IGFALS COX6C FOXI1 GRIA2 CHST8 GFRA1 AGTR1	13
FAM171A1 ITM2C HLA_DOB EPHB6 S100B PLCG2 PTPRT MID1 CBX2 TSPAN13 NME5 MAL CD52 CLCN4 GSTP1 ZBTB18 ERBB3 TTC22 SOX10 SPIB FSCN1 LMX1B SMCO4 EPN3 C3 ABAT PSAT1 SEMA3F LDHB MGAT3 SLC39A6 C1orf21 P4HTM GATA2 TESMIN TOX CCL17 PTGER3 LIMD2 ANXA1 RHBDL1 TOB1 MISP CACNA1D HSPA2 SLC34A2 CSTA CELSR2 GPRIN2 KIAA0040 KCTD3 GPR183 IKZF3 GRIK3 PRNP SLC7A4 MOB3B KRT23 KCNN4 ADGRB2 ABCC8 LRRC6 CCND1 CXCL14 DEPP1 SYT17 ID4 DNAJC12 YBX3 NPY1R SLC7A8 FGFR3 PLEKHF1 CXCL1 JCHAIN LAMC2 MT1M RLN2 IGFALS COX6C FOXI1 GRIA2 CHST8 GFRA1 AGTR1	8
FAM171A1 ITM2C EPHB6 S100B PLCG2 PTPRT MID1 CBX2 TSPAN13 NME5 MAL CD52 CLCN4 GSTP1 ZBTB18 ERBB3 TTC22 SOX10 FSCN1 LMX1B SMCO4 EPN3 C3 ABAT PSAT1 SEMA3F LDHB MGAT3 SLC39A6 C1orf21 P4HTM GATA2 TESMIN TOX CCL17 PTGER3 LIMD2 ANXA1 RHBDL1 TOB1 MISP CACNA1D HSPA2 SLC34A2 CSTA CELSR2 GPRIN2 KIAA0040 KCTD3 GPR183 IKZF3 GRIK3 PRNP SLC7A4 MOB3B KRT23 KCNN4 ADGRB2 ABCC8 LRRC6 CCND1 CXCL14 DEPP1 SYT17 ID4 DNAJC12 YBX3 NPY1R TBX3 SLC7A8 FGFR3 PLEKHF1 CXCL1 JCHAIN LAMC2 MT1M RLN2 IGFALS COX6C FOXI1 GRIA2 CHST8 GFRA1 AGTR1	6

FAM171A1 ITM2C HLA_DOB EPHB6 S100B PLCG2 PTPRT MID1 CBX2 TSPAN13 NME5 MAL CD52 CLCN4 GSTP1 ZBTB18 ERBB3 TTC22 SOX10 FSCN1 LMX1B SMCO4 EPN3 C3 ABAT PSAT1 SEMA3F LDHB MGAT3 SLC39A6 C1orf21 P4HTM GATA2 TESMIN TOX CCL17 PTGER3 LIMD2 ANXA1 RHBDL1 TOB1 MISP HSPA2 SLC34A2 CSTA CELSR2 GPRIN2 KIAA0040 KCTD3 GPR183 IKZF3 GRIK3 PRNP SLC7A4 MOB3B KRT23 ADGRB2 ABCC8 LRRC6 CCND1 CXCL14 DEPP1 SYT17 ID4 DNAJC12 YBX3 NPY1R SLC7A8 FGFR3 PLEKHF1 CXCL1 JCHAIN LAMC2 MT1M RLN2 IGFALS COX6C FOXI1 GRIA2 CHST8 GFRA1 AGTR1	5
FAM171A1 ITM2C HLA_DOB EPHB6 S100B PLCG2 PTPRT MID1 CBX2 TSPAN13 NME5 MAL CD52 CLCN4 GSTP1 ZBTB18 ERBB3 TTC22 SOX10 FSCN1 LMX1B SMCO4 EPN3 C3 ABAT PSAT1 SEMA3F LDHB MGAT3 SLC39A6 C1orf21 P4HTM GATA2 TESMIN TOX CCL17 PTGER3 LIMD2 ANXA1 RHBDL1 TOB1 MISP CACNA1D HSPA2 SLC34A2 CSTA CELSR2 GPRIN2 KIAA0040 KCTD3 GPR183 IKZF3 GRIK3 PRNP SLC7A4 MOB3B KRT23 ADGRB2 ABCC8 LRRC6 CCND1 CXCL14 DEPP1 SYT17 ID4 DNAJC12 YBX3 NPY1R SLC7A8 FGFR3 PLEKHF1 CXCL1 JCHAIN LAMC2 MT1M RLN2 IGFALS COX6C FOXI1 GRIA2 CHST8 GFRA1 AGTR1	2
FAM171A1 ITM2C HLA_DOB EPHB6 S100B PLCG2 PTPRT MID1 CBX2 TSPAN13 NME5 MAL CD52 CLCN4 GSTP1 ZBTB18 ERBB3 TTC22 SOX10 FSCN1 LMX1B SMCO4 EPN3 C3 ABAT PSAT1 SEMA3F LDHB MGAT3 SLC39A6 C1orf21 P4HTM GATA2 TESMIN TOX CCL17 PTGER3 LIMD2 ANXA1 RHBDL1 TOB1 MISP CACNA1D HSPA2 SLC34A2 CSTA CELSR2 GPRIN2 KIAA0040 KCTD3 GPR183 IKZF3 GRIK3 PRNP SLC7A4 MOB3B KRT23 KCNN4 ADGRB2 ABCC8 LRRC6 CCND1 CXCL14 DEPP1 SYT17 ID4 DNAJC12 YBX3 NPY1R SLC7A8 FGFR3 PLEKHF1 CXCL1 JCHAIN LAMC2 MT1M RLN2 IGFALS COX6C FOXI1 GRIA2 CHST8 GFRA1 AGTR1	2
FAM171A1 ITM2C HLA_DOB EPHB6 S100B PLCG2 PTPRT MID1 CBX2 TSPAN13 NME5 MAL CD52 CLCN4 GSTP1 ZBTB18 ERBB3 TTC22 SOX10 FSCN1 LMX1B SMCO4 EPN3 C3 ABAT PSAT1 SEMA3F LDHB MGAT3 SLC39A6 C1orf21 P4HTM GATA2 TESMIN TOX CCL17 PTGER3 LIMD2 ANXA1 RHBDL1 TOB1 MISP HSPA2 SLC34A2 CSTA CELSR2 GPRIN2 KIAA0040 KCTD3 GPR183 IKZF3 GRIK3 PRNP SLC7A4 MOB3B KRT23 ADGRB2 ABCC8 LRRC6 CCND1 CXCL14 DEPP1 SYT17 ID4 DNAJC12 YBX3 NPY1R SLC7A8 FGFR3 PLEKHF1 JCHAIN LAMC2 MT1M RLN2 IGFALS COX6C FOXI1 GRIA2 CHST8 AGTR1	2
FAM171A1 ITM2C HLA_DOB EPHB6 S100B PLCG2 PTPRT MID1 CBX2 TSPAN13 NME5 MAL CD52 CLCN4 GSTP1 ZBTB18 ERBB3 TTC22 SOX10 LMX1B SMCO4 EPN3 C3 ABAT SEMA3F LDHB MGAT3 SLC39A6 C1orf21 P4HTM GATA2 TESMIN TOX CCL17 PTGER3 LIMD2 ANXA1 RHBDL1 TOB1 MISP HSPA2 SLC34A2 CSTA CELSR2 GPRIN2 KIAA0040 KCTD3 GPR183 IKZF3 GRIK3 PRNP SLC7A4 MOB3B KRT23 ADGRB2 ABCC8 LRRC6 CCND1 CXCL14 DEPP1 SYT17 ID4 DNAJC12 YBX3 NPY1R SLC7A8 FGFR3 PLEKHF1 JCHAIN MT1M RLN2 IGFALS COX6C FOXI1 GRIA2 CHST8 AGTR1	1

FAM171A1 ITM2C HLA_DOB EPHB6 S100B PLCG2 PTPRT MID1 CBX2 TSPAN13 NME5 MAL CD52 CLCN4 GSTP1 ZBTB18 ERBB3 TTC22 SOX10 LMX1B SMCO4 EPN3 C3 ABAT SEMA3F LDHB MGAT3 SLC39A6 C1orf21 P4HTM GATA2 TESMIN TOX CCL17 PTGER3 LIMD2 ANXA1 RHBDL1 TOB1 MISP HSPA2 SLC34A2 CSTA GPRIN2 KIAA0040 KCTD3 GPR183 IKZF3 GRIK3 PRNP SLC7A4 MOB3B ADGRB2 ABCC8 LRRC6 CCND1 CXCL14 DEPP1 SYT17 ID4 DNAJC12 BBOX1 YBX3 NPY1R SLC7A8 FGFR3 PLEKHF1 JCHAIN MT1M RLN2 IGFALS COX6C FOXI1 PLA2G7 GRIA2 CHST8 AGTR1	1
FAM171A1 ITM2C HLA_DOB EPHB6 S100B PLCG2 PTPRT MID1 CBX2 TSPAN13 NME5 MAL CD52 GSTP1 ZBTB18 ERBB3 TTC22 SOX10 SMCO4 EPN3 SEMA3F MGAT3 C1orf21 GATA2 TESMIN TOX CCL17 PTGER3 LIMD2 ANXA1 RHBDL1 TOB1 HSPA2 SLC34A2 CSTA CELSR2 GPRIN2 KIAA0040 KCTD3 GPR183 IKZF3 GRIK3 PRNP SLC7A4 MOB3B ABCC8 LRRC6 CXCL14 DEPP1 SYT17 ID4 DNAJC12 BBOX1 YBX3 NPY1R FGFR3 JCHAIN MT1M RLN2 IGFALS COX6C FOXI1 PLA2G7 GRIA2 CHST8	1
