# Supplementary material for: DNA methylation profiling deciphers three EMT subtypes with distinct prognoses and therapeutic vulnerabilities in breast cancer
Source: J Cancer. 2024 Jul 16;15(15):4922–38. doi: 10.7150/jca.96096 (PMC11310866; doi:10.7150/jca.96096)
Supplement: Supplementary file 1 — Supplementary methods, figures and tables. [file jcav15p4922s1.zip › Table S7.pdf]

Table S7. 96 SSGs used to build subtype classification system.

FAM171A	ITM2C	EPHB6	S100B	PLCG2	PTPRT	MID1	CBX2	TSPAN13
MAL	CD52	CLCN4	GSTP1	VGLL1	ZBTB18	ERBB3	TTC22	SOX10
LMX1B	SMCO4	C3	ABAT	PSAT1	SEMA3F	LDHB	MYB	MGAT3
C1orf21	P4HTM	GATA2	TESMIN	TOX	CCL17	PTGER3	LIMD2	ANXA1
TOB1	PGR	MISP	CACNA1[illegible]	HSPA2	SLC34A2	CSTA	CELSR2	GPRIN2
KCTD3	GPR183	IKZF3	GRIK3	PRNP	SLC7A4	MOB3B	KRT23	FGFBP1
ADGRB2	ABCC8	LRRC6	CCND1	CXCL14	DEPP1	ERBB4	SYT17	ID4
YBX3	NPY1R	MCCC2	TBX3	MINDY1	SOD3	SLC7A8	FGFR3	INAVA
CXCL1	JCHAIN	LAMC2	SEL1L3	MT1M	CSRP2	RLN2	WWP1	ACOX2
COX6C	FOXI1	GRIA2	CHST8	GFRA1	AGTR1			

SSG: Subtype stem gene

NME5
FSCN1
SLC39A6
RHBDL1
KIAA0040
KCNN4
DNAJC12
PLEKHF1
IGFALS
