# Supplementary material for: DNA methylation profiling deciphers three EMT subtypes with distinct prognoses and therapeutic vulnerabilities in breast cancer
Source: J Cancer. 2024 Jul 16;15(15):4922–38. doi: 10.7150/jca.96096 (PMC11310866; doi:10.7150/jca.96096)
Supplement: Supplementary file 1 — Supplementary methods, figures and tables. [file jcav15p4922s1.zip › Table S8.pdf]

Table S8A. The quantified classifier performance based on SVM algorithm.

bal_accuracy	pr_auc	roc_auc
0.845278668	0.862665596	0.926472275
0.838048326	0.872504255	0.928552739
0.853791912	0.877408379	0.9332113
0.864519351	0.895058548	0.941795408
0.841435173	0.870532532	0.928057048
0.838446593	0.868775065	0.928693924
0.851755936	0.880349363	0.935637326
0.844037506	0.876191968	0.933331648
0.858935451	0.880671217	0.934676623
0.852486383	0.878769838	0.933706603
0.843758811	0.862988368	0.926328023
0.841126589	0.874620817	0.928872385
0.840254812	0.869541278	0.92724277
0.847762935	0.871041545	0.9304317
0.850489414	0.870412782	0.931207636
0.842393608	0.875566844	0.931698914
0.848051899	0.867590473	0.927938759
0.844062346	0.864484898	0.926765191
0.85139016	0.877087673	0.933275049
0.854809975	0.871574856	0.930897729

Table S8B. The quantified classifier performance based on Random Forest algorithm.

bal_accuracy	pr_auc	roc_auc
0.824671108	0.843021471	0.912510967
0.825550992	0.850738852	0.913985764
0.825060698	0.852127035	0.917758788
0.844516478	0.874729949	0.92717415
0.825196633	0.846856232	0.912884558
0.82120312	0.843588103	0.912080201
0.822117601	0.851875493	0.918611665
0.822749123	0.856596568	0.919168385
0.834904407	0.857029183	0.919429274
0.840170478	0.854488375	0.919796244

0.826946926	0.84540897	0.914255394
0.823813908	0.859093929	0.917274875
0.825667659	0.848641895	0.914717202
0.830572277	0.84692629	0.91593084
0.828684133	0.847492732	0.917067133
0.837150513	0.855176757	0.918139945
0.820218205	0.84088372	0.909741068
0.829062804	0.847666159	0.914179626
0.826565258	0.853629715	0.918750081
0.835451219	0.853111908	0.919342029

Table S8C. The quantified classifier performance based on LightGBM algorithm.

bal_accuracy	pr_auc	roc_auc
0.833092577	0.841915512	0.913375865
0.827238511	0.846037698	0.912264866
0.83393368	0.854912479	0.920671021
0.853957918	0.879071142	0.931063414
0.825745746	0.842362356	0.910140718
0.826918362	0.842202399	0.910255964
0.83693366	0.859064076	0.923294932
0.82831071	0.85237675	0.918399091
0.839744508	0.851076364	0.917361235
0.838701304	0.853056662	0.919404116
0.832544009	0.845111146	0.914560988
0.835970607	0.860120705	0.920710384
0.830871086	0.841336361	0.911033899
0.822860703	0.848489422	0.914781259
0.834123352	0.850250599	0.917270632
0.831389875	0.848588611	0.914715537
0.825748223	0.832406292	0.90652974
0.83073067	0.847962837	0.913850808
0.83395156	0.85445459	0.919566089
0.840140318	0.852314703	0.920651066

Table S8D. The quantified classifier performance based on Xgboost algorithm.

bal_accuracy	pr_auc	roc_auc
0.831407286	0.835835074	0.909252988
0.82530175	0.846456949	0.91231397
0.836948235	0.859214306	0.92238806
0.851614945	0.880475342	0.932726889
0.823970946	0.844762765	0.912098866
0.82742155	0.84415216	0.911712611
0.839580293	0.857975439	0.922814126
0.833134888	0.85224882	0.919418227
0.837592343	0.856481755	0.91945128
0.844823392	0.857591035	0.920383808
0.832049742	0.842966938	0.913130706
0.832261104	0.860866407	0.919408384
0.829266513	0.846249603	0.912324919
0.830691285	0.855214022	0.918397472
0.839219134	0.852498607	0.91865421
0.830959017	0.844193527	0.914709032
0.827139033	0.830592465	0.90528435
0.82652563	0.845224097	0.913954042
0.831224376	0.854210875	0.919116003
0.837735894	0.849262625	0.920028122

Table S8E. The quantified classifier performance based on NB algorithm.

bal_accuracy	pr_auc	roc_auc
0.815008191	0.806478816	0.897206789
0.812618218	0.815924463	0.899015422
0.814204035	0.805611825	0.900265952
0.825903927	0.82783215	0.908215023
0.816702214	0.817432462	0.9008568
0.805408367	0.801815379	0.895453616
0.810335272	0.802431889	0.896401321
0.801349973	0.815740698	0.901934726
0.812740926	0.823531722	0.906675462
0.823644287	0.824535504	0.906039869
0.814746589	0.81011468	0.897934072

0.809036953	0.807559188	0.897068458
0.811464974	0.814097573	0.901994128
0.815830305	0.802114978	0.899151427
0.81942621	0.814825686	0.903087349
0.825376252	0.818488988	0.903299005
0.809264824	0.805862434	0.89486285
0.809001725	0.810473466	0.898017293
0.813950752	0.813226938	0.90255389
0.815561719	0.815716077	0.904222482
