# Supplementary material for: DNA methylation profiling deciphers three EMT subtypes with distinct prognoses and therapeutic vulnerabilities in breast cancer
Source: J Cancer. 2024 Jul 16;15(15):4922–38. doi: 10.7150/jca.96096 (PMC11310866; doi:10.7150/jca.96096)
Supplement: Supplementary file 1 — Supplementary methods, figures and tables. [file jcav15p4922s1.zip › Table S9.pdf]

Table S9. Significant copy number losses and gains associated to each subtype.

Cluster	Event	Cytoband	Wide Peak Boundaries	Q Value	Residual Q Value	Frequency	Genes In Wide Peak
	Deletion	16q23.3	chr16:71576783-90338345	0.00043317	0.0004413	74.93403694	AP1G1;AFG3L1P;APRT;ZFHX3;GAS8-AS1;CA5A;CBFA2T3;CDH13;CDH15;COX4I1;CTRB1;CYBA;DHODH;DPEP1;FANCA;FOXF1;FOXL1;FOXC2;GALNS;GAS8;GCSH;GLG1;HP;HPR;HSBP1;HSD17B2;IRF8;KARS;MAF;MC1R;CHST6;MVD;CHMP1A;PLCG2;PSMD7;RPL13;SPG7;GAN;SLC7A5;CDK10;MBTPS1;TAF1C;USP10;BCAR1;VPS9D1;KIAA0513;PIEZO1;DHX38;IST1;ATP2C2;CLEC3A;MPHOSPH6;EMC8;TUBB3;CFDP1;PRDM7;GABARAPL2;MON1B;TCF25;PHLPP2;ZCCHC14;GSE1;ATMIN;COTL1;MLYCD;ADAT1;CHST5;CPNE7;IL17C;ANKRD11;OSGIN1;GINS2;TRAPPC2L;WWOX;BCO1;TERF2IP;NECAB2;KLHDC4;DEF8;TXNL4B;BANP;RFWD3;ZNF821;ZDHHC7;CENPN;CMC2;JPH3;VAT1L;TLDC1;WFDC1;MTHFSD;DBNDD1;FA2H;TMEM231;WDR59;KLHL36;FBXO31;CMIP;CDT1;MAP1LC3B;PMFBP1;DYNLRB2;HSDL1;CRISPLD2;SPIRE2;ZNF469;ZNRF1;CNTNAP4;MARVELD3;CENPBD1;ZNF276;KCNG4;SDR42E1;PKD1L2;RNF166;C16orf46;DNAAF1;SPATA2L;SPATA33;ZC3H18;CDYL2;TMEM170A;SLC38A8;SLC22A31;FLJ30679;LOC146513;ZFPM1;ADAD2;ZFP1;ADAMTS18;SNAI3-AS1;LINC00311;LDHD;MLKL;ZNF778;ACSF3;LINC00304;HCCAT5;LOC283922;NUDT7;SNAI3;LOC339059;FAM92B;ATXN1L;PKD1L3;CTU2;C16orf47;PABPN1L;LOC400548;FENDRR;LOC400553;LOC400558;C16orf74;NPIPB15;CTRB2;LOC440390;CLEC18B;SNORD68;SNORD71;LINC00917;VPS9D1-AS1;LOC100129617;LOC100129697;URAHP;SYCE1L;LOC100287036;LOC100289580;MIR1910;SNORA70D;MIR3182;LINC01568;LOC100506281;LINC01082;C16orf95;MIR4720;MIR4722;MIR4719;MIR5093;MIR5189;PRCAT47;FAM157C;LINC01081;LOC101927793;LOC101927817;LINC01572;LOC101928035;LOC101928203;LINC01229;LINC01227;LOC101928417;LOC101928446;LOC101928557;LOC101928614;LOC101928659;LOC101928682;LOC101928708;LOC101928737;LOC101928880;MIR6504;MIR6775;MIR7854;MIR8058;MIR6774;MAFTRR;LOC102724084;LOC102724163;LOC102724344;LOC102724467;FOXC2-AS1;LOC105371414;LOC105376772
	Deletion	17p12	chr17:11993313-12552764	1.35E-07	1.78E-07	52.50659631	MAP2K4;MIR744
	Deletion	11q23.1	chr11:8918723-128525437	3.75E-08	0.0001568	49.60422164	ACAT1;ACP2;ACRV1;ACTN3;ADM;GRK2;APLNR;ALDH3B1;ALDH3B2;AMPD3;BIRC2;BIRC3;APOA1;APOA4;APOC3;ARCN1;ARHGAP1;PHOX2A;ARL2;ARNTL;ARRB1;ATM;FXYD2;BAD;BBS1;CCND1;BDNF;CXCR5;SERPING1;CAPN5;VPS51;MRPL49;ZNHIT2;MPPED2;MYRF;TMEM258;DAGLA;CALCA;CALCB;CAPN1;CASP1;CASP4;CASP5;CAT;CBL;SERPINH1;CD3D;CD3E;CD3G;CD5;CD6;MS4A1;MS4A3;CD44;CD59;CFL1;CTSC;CHEK1;CHKA;CHRM1;CHRM4;CLNS1A;CNTF;COPB1;COX8A;CPT1A;CRY2;CRYAB;CST6;CSTF3;CTNND1;CTSW;DDB1;DDB2;DDX6;DDX10;DHCR7;DLAT;DLG2;DPAGT1;DRD2;EEF1G;EIF4G2;ELF5;MARK2;CTTN;ESRRA;ETS1;EXT2;F2;FANCF;FAU;MS4A2;FDX1;FEN1;FGF3;FGF4;FKBP2;FOLH1;FOLR1;FOLR2;FOLR3;FSHB;FTH1;FUT4;SLC37A4;LRRC32;GAS2;GIF;GNG3;GRIA4;GRIK4;GRM5;GSTP1;GTF2H1;GUCY1A2;H2AFX;HMBS;SLC29A2;HSPA8;HSPB2;DNAJC4;HTR3A;IGHMBP2;IL10RA;IL18;INCENP;INPPL1;STT3A;CD82;KCNA4;KCNC1;KCNJ11;KRTAP5-9;LDHA;LDHC;FADS1;FADS3;LMO2;VWA5A;LRP4;LRP5;LTBP3;CAPRIN1;MAP6;MCAM;MDK;MEN1;SCGB2A1;SCGB2A2;MAP3K11;KMT2A;MMP1;MMP3;MMP7;MMP8;MMP10;MMP12;MMP13;MRE11A;MTNR1B;MYBPC3;MYO7A;MYOD1;NCAM1;NDUFC2;NDUFS3;NDUFV1;NDUFS8;NELL1;NNMT;NPAT;NRGN;NUCB2;NUMA1;OMP;OSBP;OVOL1;P2RX3;P2RY2;P2RY6;PAFAH1B2;PAK1;PAX6;PC;PDE2A;PDE3B;PGA5;PGR;PIK3C2A;PLCB3;POLR2G;POU2AF1;PPP1CA;PPP2R1B;PPP2R5B;PRCP;PRG2;THAP12;PSMA1;PSMC3;PTH;PTPRCAP;PTPRJ;PTS;NECTIN1;PYGM;RAB3IL1;RAB6A;MAP4K2;RAD9A;RAG1;RAG2;RAPSN;RARRES3;RBM4;RCN1;RDX;RELA;DPF2;SNORD15A;ROM1;RPS3;RPS6KB2;RPS13;RPS25;SAA1;SAA2;SAA3P;SAA4;SC5D;SCN2B;SCN4B;SDHD;ST3GAL4;SIPA1;SLC1A2;SLC3A2;SLN;SORL1;SPI1;SPTBN2;SRPRA;SSRP1;STX3;STX5;ABCC8;TAGLN;TCN1;TEAD1;TECTA;THRSP;THY1;TM7SF2;TPH1;TRAF6;TRPC6;TSG101;TYR;UCP2;UCP3;SCGB1A1;UPK2;UVRAG;VEGFB;BEST1;WEE1;WNT11;WT1;SF1;ZFPL1;ZNF143;ZBTB16;ZNF202;CSRP3;PDHX;FOSL1;CUL5;PICALM;FZD4;BBOX1;PPFIA1;SLC43A1;DGKZ;CHST1;API5;MADD;OR7E2P;JRKL;CTSF;EED;FADD;BANF1;ZPR1;MTMR2;RPS6KA4;AIP;SYT7;SART1;USP2;SLC6A5;FIBP;PCSK7;HTR3B;ZW10;MTA2;UBE2L6;SNORD29;SNORD31;SNORD30;SNORD28;SNORD27;SNORD26;SNORD25;SNORD22;MMP20;UBE4A;SLC22A6;SLC22A8;NRXN2;LPXN;PEX16;FADS2;MED17;MAPK8IP1;TP53I11;EI24;PITPNM1;RIN1;TESMIN;FEZ1;MICAL2;CTR9;CEP57;ATG13;SPCS2;CKAP5;ARHGEF17;GAB2;C2CD2L;FCHSD2;FGF19;CCS;RCE1;NAALAD2;NAALADL1;KCNE3;NR1H3;IL18BP;DPP3;USH1C;KCNK7;HIPK3;RBM7;PRMT3;MPZL2;RASGRP2;GLYAT;CDK2AP2;TCIRG1;RTN3;MRVI1;PRG3;YAP1;SPON1;RBM14;CDC42EP2;EIF3M;NXF1;KAT5;HYOU1;IPO7;SSSCA1;HTATIP2;DRAP1;TAF6L;ATP5L;SCGB1D2;SCGB1D1;CELF1;POLD3;OR5I1;STARD10;OR7E14P;NEU3;ME3;GPR83;LYVE1;YIF1A;SRSF8;EHD1;C11orf58;STIP1;CLP1;SF3B2;CCDC85B;B4GAT1;PRSS23;PLA2G16;TREH;HPS5;PTGDR2;SLCO2B1;RRAS2;CEP164;ZP1;SHANK2;KDM2A;ENDOD1;SWAP70;EXPH5;ATG2A;PHLDB1;GANAB;FAM168A;DTX4;SIK2;DENND5A;NUP160;FNBP4;ARHGEF12;SIK3;CLCF1;VSIG2;BACE1;FAM89B;SNHG1;POLA2;TRIM29;RAB38;CADM1;FLRT1;MTCH2;PANX1;FJX1;KIAA1549L;PRDX5;POU2F3;ABTB2;BRMS1;TEX40;CHRDL2;PAMR1;ANAPC15;ZDHHC5;ATL3;TSKU;HINFP;REXO2;C2CD3;TKFC;TENM4;DKFZP434K028;B3GAT3;FBXO3;SERGEF;EHF;OR5L2;PPP1R14B;OR8G2;OR8B8;OR8G1;TIMM10;TIMM8B;MYEOV;BSCL2;OR8B2;ELP4;SNORD14A;CHORDC1;DKK3;RAB30;PRPF19;DCPS;C11orf54;AAMDC;MACROD1;SLC43A3;COMMD9;DDX25;PACSIN3;SAC3D1;SNX15;RHOD;EFEMP2;NOX4;KCNK4;RNF141;CDON;UBXN1;APIP;TMX2;GAL;SIDT2;KMT5B;HSD17B12;TMEM216;COA4;SLC15A3;PHF21A;MS4A4A;WT1-AS;TRAPPC4;PPME1;CABP2;HIKESHI;CWC15;TRMT112;TMEM138;PCF11;MRPL48;RSF1;SPA17;FXYD6;C11orf24;CNTN5;SIAE;C11orf71;ZDHHC13;ROBO4;WDR74;SLC35F2;RAB39A;TRIM44;BTG4;NXPE4;SYTL2;ANKRD49;MS4A12;MRPL16;SDHAF2;SSH3;TTC12;TMEM132A;HRASLS2;LAMTOR1;USP47;VPS37C;ANO1;NADSYN1;FAM86C1;C11orf57;NAT10;CCDC87;PPP6R3;UEVLD;RNF121;LIN7C;ACER3;SLC35C1;TCP11L1;LGR4;ELMOD1;SOX6;CDC42BPG;FOXRED1;OTUB1;AMBRA1;PACS1;KDM4D;KBTBD4;PARVA;TTC17;SCN3B;VPS11;TMEM126B;SLC22A11;TEX12;CRTAM;TMPRSS4;C11orf16;TMEM9B;NRIP3;ASCL3;GPR137;IFT46;SMCO4;EMSY;PRDM11;CABP4;SLC17A6;TRIM49;CD248;CORO1B;SCYL1;DSCAML1;GRAMD1B;USP35;CEP126;ARHGAP20;CARNS1;SYT13;USP28;LRRC4C;SCUBE2;POLD4;KRTAP5-8;PLEKHB1;MS4A7;ZBED5;CREBZF;CARD18;CCDC90B;CCDC81;AASDHPPT;ALX4;PKNOX2;FAM111A;ANO3;MMP27;ABCG4;ROBO3;MS4A6A;MS4A5;C11orf1;KLC2;TUT1;MRPL11;T[illegible]
	Amplification	20q13.2	chr20:53513903-53743037	1.03E-16	2.23E-11	44.85488127	ZNF217;LOC101927770;LOC105372672
	Deletion	11q25	chr11:119345522-135086622	6.79E-08	6.23E-05	43.7994723	ACRV1;APLP2;CHEK1;ETS1;FLI1;GRIK4;HSPA8;STT3A;KCNJ1;KCNJ5;VWA5A;NFRKB;NRGN;OPCML;NECTIN1;SC5D;ST3GAL4;SORL1;SRPRA;ST14;TECTA;THY1;ZNF202;BARX2;USP2;EI24;FEZ1;ARHGAP32;ADAMTS8;IGSF9B;NCAPD3;ARHGEF12;VSIG2;TRIM29;POU2F3;OR8G2;OR8B8;OR8G1;OR8B2;ACAD8;B3GAT1;DCPS;ZBTB44;THYN1;DDX25;NTM;CDON;SPA17;SIAE;ROBO4;FOXRED1;SCN3B;CRTAM;PRDM10;GRAMD1B;PKNOX2;TP53AIP1;ROBO3;FAM118B;MSANTD2;CLMP;C11orf63;CCDC15;PUS3;JAM3;KIRREL3;RPUSD4;TBRG1;UBASH3B;SNORD14C;SNORD14D;SNORD14E;GLB1L2;ESAM;VPS26B;GLB1L3;TIRAP;PANX3;TMEM45B;PATE1;ADAMTS15;C11orf45;HYLS1;TMEM218;SLC37A2;OR8B12;OR8G5;OR10G8;OR10G9;OR10S1;OR6T1;OR4D5;TBCEL;TMEM136;SPATA19;HEPACAM;OAF;OR8D1;OR8D2;OR8B4;KIRREL3-AS3;C11orf44;MIR4697HG;LOC283177;TMEM225;OR8D4;LOC341056;BSX;OR6X1;OR6M1;OR10G4;OR10G7;OR8B3;OR8A1;MIR100HG;PATE2;PATE4;ST3GAL4-AS1;SNX19;LOC403312;MIRLET7A2;MIR100;MIR125B1;BLID;LINC00167;HEPN1;LOC646522;LOC649133;LOC100128239;PATE3;MIR3167;USP2-AS1;LOC100507283;SENCR;LOC100507431;LOC100507548;MIR4697;MIR4493;KIRREL3-AS2;NTM-IT;LOC101929208;LOC101929227;LOC101929340;LOC101929427;LOC101929473;LOC101929497;LOC101929517;LOC101929538;LINC01395;NTM-AS1;LOC101929653;MIR7641-2;MIR6090;MIR8052;LOC102724301;LOC103611081;PKNOX2-AS1;LOC105369532;STT3A-AS1
	Amplification	20q13.13	chr20:49654240-50555349	4.14E-07	0.00035555	42.48021108	CEBPB;PTPN1;SNAI1;UBE2V1;B4GALT5;SPATA2;SLC9A8;RNF114;LINC01270;TMEM189;TMEM189-UBE2V1;LINC01272;TRERNA1;LINC01273;CEBPB-AS1;LINC01271;LOC105372653
	Amplification	8p11.21	chr8:42112638-42143464	1.71E-17	1.96E-08	39.31398417	[LOC105379393]
	Amplification	8p11.23	chr8:37693889-37752987	1.40E-35	2.38E-28	38.52242744	ERLIN2;ZNF703;LOC728024;LOC101929622;LOC102723701
	Amplification	8p11.21	chr8:40245537-40787268	6.58E-17	0.0001663	37.73087071	ZMAT4

C1	Deletion	13q14.2	chr13:48301193-48490671	1.14E-15	2.30E-13	37.73087071	RB1;LPAR6
	Amplification	11q13.3	chr11:69619505-69661497	3.20E-92	2.20E-79	37.20316623	CCND1
	Deletion	6q21	chr6:108524809-109442510	7.20E-08	4.20E-07	37.20316623	FOXO3;CD164;SESN1;ARMC2;CCDC162P;CEP57L1;PPIL6;LINC00222;LOC100996634;ARMC2-AS1
	Amplification	17q23.1	chr17:59414286-59892473	3.42E-13	1.15E-06	35.35620053	CLTC;TUBD1;PTRH2;DHX40;VMP1;MIR21;LINC01476
	Deletion	1p21.3	chr1:78483454-110633654	2.14E-05	0.00050608	32.98153034	ABCA4;AGL;ALX3;AMPD2;AMY1A;AMY1B;AMY1C;AMY2A;AMY2B;BRDT;CLCA1;CNN3;COL11A1;CSF1;CTBS;DBT;DPYD;DR1;S1PR1;CELSR2;EXTL2;F3;GBP1;GBP2;GBP3;GFI1;GCLM;GNAI3;GNAT2;GNG5;GSTM1;GSTM2;GSTM3;GSTM4;GSTM5;GTF2B;CYR61;KCNA2;KCNA10;KCNC4;PRKACB;PKN2;PSMA5;PTGFR;ABCD3;SNORD21;RPL5;SORT1;SARS;STXBP3;TAF13;TGFBR3;VCAM1;EVI5;CDC7;BCAR3;LMO4;CDC14A;RTCA;BCL10;SLC16A4;SEP15;ARHGAP29;CLCA3P;CLCA2;HS2ST1;PLPPR4;VAV3;LAMTOR5;IFI44;AHCYL1;IFI44L;GLMN;CLCA4;MTF2;NTNG1;WDR47;CLCC1;ADGRL2;KIAA1107;SLC35A3;LRRC8B;LPAR3;DDAH1;RWDD3;SNORA66;GPSM2;SLC25A24;DNTTIP2;TMED5;SH3GLB1;SNX7;DPH5;GPR88;TRMT13;ZNHIT6;PALMD;FNBP1L;PRPF38B;LRRC8D;PRMT6;MCOLN3;RNPC3;KYAT3;TMEM167B;AMIGO1;ODF2L;KIAA1324;PTBP2;DNASE2B;ADGRL4;MFSD14A;RBM15;EPS8L3;TTLL7;RPAP2;RPF1;GPR61;SYDE2;ZNF644;LRRC8C;PROK1;PSRC1;STRIP1;DNAJA1P5;HENMT1;GBP4;GBP5;SSX2IP;OLFM3;WDR63;SLC44A3;ATXN7L2;C1orf194;LRRC39;SAMD13;C1orf52;TMEM56;NBPF4;SLC30A7;GBP6;PLPPR5;FNDC7;SASS6;HFM1;UBL4B;ALG14;EPHX4;AKNAD1;MCOLN2;COL24A1;FAM102B;SYPL2;CYB561D1;ZNF326;BTBD8;LINC01140;CCDC18;MYBPHL;BARHL2;GBP7;C1orf146;FAM69A;SLC6A17;UOX;FRRS1;GBP1P1;FLJ27354;MIR137HG;MIR137;MIR197;LINC01555;FLJ31662;LOC440600;LOC440602;GEMIN8P4;RBMXL1;SPATA42;CYMP;LOC646626;SETSIP;ACTG1P4;NBPF6;SCARNA2;MIR553;LOC729930;LOC729970;LOC729987;MIR760;LINC01349;LOC100129046;LOC100129138;LOC100129620;CCDC18-AS1;MIR548N;SPATA1;LINC01364;RTCA-AS1;TMEM56-RWDD3;MIR378G;MIR2682;MIR4423;DPYD-AS1;DPYD-AS2;VAV3-AS1;KCNC4-AS1;LOC100996630;LOC100996635;LAMTOR5-AS1;LOC101927412;LOC101927434;LINC01361;LOC101927560;LOC101927587;LOC101927844;PKN2-AS1;LINC01057;LOC101928098;LOC101928118;LOC101928241;LOC101928270;LOC101928370;LOC101928436;LOC101928476;MIR7852;MIR7856;LOC102606465;LOC102723661;LINC01461;LINC01397;LINC01307;LOC105378828;LOC105378853
	Deletion	6q27	chr6:163666941-170805979	8.66E-05	8.23E-05	32.45382586	CCR6;GPR31;KIF25;AFDN;PDCD2;PSMB1;RPS6KA2;T;TBP;TCP10;TCTE3;THBS2;RNASET2;PDE10A;FGFR1OP;LINC01558;DLL1;MPC1;UNC93A;PHF10;ERMARD;SMOC2;FRMD1;LINC00574;TTLL2;FAM120B;LINC00473;SFT2D1;LOC154449;DACT2;C6orf118;WDR27;PRR18;LOC285804;C6orf120;TCP10L2;LOC401286;LINC00242;LINC01624;LINC00602;LOC441178;AFDN-AS1;HGC6.3;LOC100131532;LOC100289495;MIR1913;MIR3939;KIF25-AS1;MIR4644;RPS6KA2-AS1;RPS6KA2-IT1;LOC101929297;LOC101929420;LOC101929460;LINC01615;LOC101929504;LOC101929523;MIR7641-2;LOC102724357;LOC102724511;MEAT6;LOC105378123;LOC105378127;LOC105378137;LOC105378146
	Amplification	17q21.33	chr17:50197932-50529175	7.16E-10	9.45E-06	30.34300792	CHAD;COL1A1;MRPL27;RSAD1;LRRC59;XYLT2;ACSF2;MYCBPAP;EME1;TMEM92;LOC101927230;TMEM92-AS1
	Amplification	11q13.4	chr11:71096728-71232255	1.11E-35	2.32E-05	28.23218997	SHANK2
	Deletion	9p21.3	chr9:21555228-23507724	1.86E-07	1.86E-07	27.176781	CDKN2A;CDKN2B;MTAP;CDKN2A-AS1;DMRTA1;LINC01239;CDKN2B-AS1
	Amplification	12q15	chr12:68789499-69645872	6.80E-11	6.80E-11	26.64907652	CPM;LYZ;MDM2;YEATS4;CCT2;FRS2;CPSF6;BEST3;LRRC10;LOC100130075;MIR1279;MIR3913-2;MIR3913-1
	Amplification	17q12	chr17:39502604-39990675	2.91E-18	1.15E-14	26.64907652	ERBB2;GRB7;NEUROD2;PNMT;PSMD3;TCAP;STARD3;IKZF3;CDK12;GSDMB;PPP1R1B;MIEN1;PGAP3;ORMDL3;ZPBP2;GSDMA;LRRC3C;MIR4728
	Amplification	17q11.2	chr17:28348212-29504781	7.23E-08	3.86E-07	26.38522427	ALDOC;CRYBA1;FLOT2;RPL23A;SDF2;SUPT6H;VTN;FOXN1;SLC13A2;UNC119;TIAF1;TRAF4;KIAA0100;SPAG5;SARM1;POLDIP2;ERAL1;SNORD4B;SNORD4A;SNORD42B;SNORD42A;PIPOX;FAM222B;NUFIP2;TAOK1;PHF12;RAB34;PIGS;SLC46A1;TLCD1;SGK494;SEZ6;TMEM199;PROCA1;DHRS13;NEK8;MYO18A;MIR144;MIR451A;SEBOX;SPAG5-AS1;MIR4523;MIR451B;MIR4732;MIR4723;NARR;LOC101927018
	Deletion	9p23	chr9:7799607-10619051	1.79E-06	1.74E-06	25.85751979	PTPRD;PTPRD-AS1;LOC105375972
	Amplification	11q13.2	chr11:67362560-67447733	1.27E-21	3.74E-13	25.3298153	PPP1CA;PTPRCAP;RAD9A;RPS6KB2;CLCF1;CORO1B;CARNS1;TBC1D10C;LOC100130987
	Deletion	19p13.3	chr19:1530035-1846111	8.10E-08	8.10E-08	24.53825858	TCF3;UQCR11;MBD3;ATP8B3;ONECUT3;MEX3D;LOC100288123;MIR1909
	Amplification	19q13.42	chr19:54507513-56420302	2.24E-05	0.00034555	23.21899736	FCAR;IL11;KIR2DL1;KIR2DL2;KIR2DL3;KIR2DL4;KIR2DS1;KIR2DS3;KIR2DS4;KIR2DS5;KIR3DL1;KIR3DL2;KIR3DS1;LAIR2;PTPRH;RPL28;SYT5;TNNI3;TNNT1;NCR1;LILRB1;LILRB4;LILRA1;LILRA2;U2AF2;PPP6R1;HSPBP1;UBE2S;CCDC106;EPN1;ZNF580;GP6;ZNF581;PPP1R12C;EPS8L1;ZNF444;NLRP2;NAT14;ZSCAN5A;LILRP2;ISOC2;BRSK1;KMT5C;FIZ1;GALP;ZNF628;KIR3DX1;RDH13;KIR3DL3;COX6B2;NLRP13;NLRP8;NLRP5;ZNF787;TMEM190;ZNF524;ZNF784;NLRP4;ZNF542P;ZNF582;ZNF583;ZNF579;NLRP7;NLRP11;TMEM86B;SSC5D;TMEM150B;FAM71E2;NLRP9;RFPL4A;ZSCAN5B;DNAAF3;ZNF582-AS1;TMEM238;SBK2;SHISA7;RFPL4AL1;SBK3;KIR2DS2;ZNF865;LOC101928804;LOC101928886;MIR6802;MIR6804;MIR6805;MIR8061;MIR6803;MIR7975
	Deletion	11p15.5	chr11:1-4094731	2.23E-05	2.31E-05	21.10817942	AP2A2;RHOG;ART1;ASCL2;CARS;CD81;CD151;CDKN1C;CTSD;DRD4;DUSP8;HRAS;IGF2;INS;IRF7;KCNQ1;LSP1;MUC2;MUC5AC;MUC6;NAP1L4;NUP98;SLC22A18;SLC22A18AS;POLR2L;PSMD13;RNH1;MRPL23;RPLP2;SCT;STIM1;TALDO1;TH;TSPAN4;TNNI2;TNNT3;TRPC2;PHLDA2;ZNF195;RASSF7;IFITM1;BRSK2;TSPAN32;TSSC4;IFITM3;DEAF1;IFITM2;OR7E12P;KCNQ1OT1;PKP3;SIRT3;PGAP2;C11orf21;TRPM5;IGF2-AS;BET1L;CEND1;CDHR5;TOLLIP;PIDD1;KCNQ1DN;CHRNA10;PNPLA2;PHRF1;SIGIRR;RIC8A;EPS8L2;CHID1;SLC25A22;PGGHG;PTDSS2;MOB2;SYT8;ODF3;OSBPL5;LRRC56;MRGPRE;ART5;LOC143666;SCGB1C1;NLRP6;LOC171391;TOLLIP-AS1;LMNTD2;H19;CRACR2B;TMEM80;MRGPRG-AS1;ANO9;KRTAP5-AS1;KCNQ1-AS1;B4GALNT4;PDDC1;MRGPRG;KRTAP5-1;KRTAP5-3;KRTAP5-4;IFITM5;FAM99A;IFITM10;MIR210;KRTAP5-5;KRTAP5-2;KRTAP5-6;MIR483;SNORA52;TSSC2;SNORA54;INS-IGF2;MUC5B;MIR675;FAM99B;LINC01001;MRPL23-AS1;MIR4298;MIR210HG;MIR4686;MIR4687;PANO1;LOC101927503;LINC01150;CD81-AS1;LOC101927708;MIR6743;MIR7847;MIR6744;HOTS;LINC01219;SNORD131
	Deletion	3p21.31	chr3:45795574-54123975	1.31E-05	0.0007848	20.58047493	ACY1;ALAS1;AMT;APEH;RHOA;CACNA1D;SLC25A20;CAMP;CDC25A;CISH;CCR1;CCR3;CCR5;COL7A1;DAG1;DOCK3;DUSP7;CELSR3;GNAI2;GNAT1;XCR1;GPX1;GRM2;HYAL1;IMPDH2;ITIH1;ITIH3;ITIH4;LAMB2;LTF;MAP4;MST1;MST1R;MYL3;PFKFB4;PLXNB1;PRKAR2A;PRKCD;PTH1R;QARS;RPL29;SEMA3F;SMARCC1;NEK4;TCTA;TDGF1;TKT;TNNC1;UBA7;USP4;UQCRC1;IFRD2;MAPKAPK3;SEMA3B;MANF;BAP1;HYAL3;HYAL2;BSN;CCRL2;RRP9;CACNA2D2;DCAF1;IP6K1;PARP3;RBM6;RBM5;NME6;TRAIP;ARIH2;NPRL2;CXCR6;CSPG5;CCR9;USP19;SLC38A3;CYB561D2;TMEM115;WDR6;RASSF1;NISCH;TREX1;TUSC2;TWF2;DHX30;SCAP;LAMB2P1;RAD54L2;STAB1;NBEAL2;KLHL18;NAT6;ABHD14A;POC1A;NDUFAF3;PTPN23;DNAH1;GNL3;SPCS1;SETD2;PRSS50;RBM15B;GMPPB;C3orf18;SHISA5;ZMYND10;TEX264;TMA7;ZNF589;HEMK1;IP6K2;SFMBT1;NCKIPSD;PHF7;TLR9;LZTFL1;P4HTM;ELP6;QRICH1;DALRD3;PBRM1;CHDH;IL17RB;DCP1A;GLT8D1;SEMA3G;PCBP4;SELK;RNF123;KIF9;CCDC71;NT5DC2;SLC26A6;CAMKV;LRRC2;FYCO1;CCDC51;WDR82;RTP3;LRRC2-AS1;ATRIP;NICN1;MON1A;ABHD14B;UCN2;RFT1;ACTR8;GPR62;IQCF1;GLYCTK;PPM1M;LSMEM2;CCDC12;KLHDC8B;ALS2CL;TMIE;FBXW12;KIF9-AS1;CCDC36;PRSS42;C3orf62;TMEM110;PRSS45;AMIGO3;CDHR4;FAM212A;IQCF2;IQCF5;MUSTN1;IQCF3;MIRLET7G;MIR135A1;MIR191;TMEM89;IQCF6;SMIM4;MIR425;SPINK8;ARIH2OS;C3orf84;SNORD19;SNORD69;MIR566;CCR2;SNORD19B;LINC00696;SEMA3F-AS1;NRADDP;LOC100132146;BSN-AS2;PRSS46;MIR1226;MIR2115;MIR711;MIR4271;PRKAR2A-AS1;IQCF4;ABHD14A-ACY1;TMEM110-MUSTN1;MIR4793;MIR4787;MIR4443;RBM5-AS1;MIR5193;ITIH4-AS1;GLYCTK-AS1;FCF1P2;SEMA3B-AS1;IQCF5-AS1;LOC101929054;RASSF1-AS1;MIR5787;MIR6823;MIR6824;MIR6872;MIR6890;MIR8064;LOC102724297;CELSR3-AS1;SNORD136;SNORA94
	Deletion	10q23.31	chr10:87857401-88274281	5.35E-06	7.99E-06	20.31662269	PTEN;KLLN
	Amplification	11q13.4	chr11:72447532-72465666	2.55E-12	0.00060861	20.05277045	[CLPB]
	Amplification	11q13.4	chr11:75109102-75207902	1.74E-15	5.69E-06	20.05277045	SLCO2B1
	Deletion	11q13.1	chr11:64313535-65917140	0.00025372	0.00094463	19.52506596	ARL2;VPS51;MRPL49;ZNHIT2;CAPN1;CFL1;CTSW;FAU;LTBP3;MEN1;MAP3K11;OVOL1;PPP2R5B;PYGM;MAP4K2;RELA;DPF2;SIPA1;TM7SF2;SF1;ZFPL1;FOSL1;RPS6KA4;FIBP;NRXN2;NAALADL1;KCNK7;RASGRP2;CDC42EP2;KAT5;SSSCA1;EHD1;CCDC85B;ATG2A;FAM89B;POLA2;PRDX5;SAC3D1;SNX15;EFEMP2;TRMT112;CDC42BPG;SLC22A11;SCYL1;MUS81;FRMD8;RNASEH2C;SYVN1;AP5B1;CDCA5;BATF2;SLC22A12;GPHA2;TIGD3;SSSCA1-AS1;EHBP1L1;SNX32;MAJIN;SLC25A45;NEAT1;CCDC88B;MALAT1;SPDYC;PCNX3;MIR192;MIR194-2;SLC22A20;MIR612;TMEM262;MIR1237;ARL2-SNX15;MIR4489;MIR4690;LOC100996455;OVOL1-AS1;MIR6751;MIR6750;MIR6749;MIR6879;MIR7155;MIR194-2HG
	Amplification	11q14.1	chr11:77624192-77931155	8.44E-25	5.87E-17	19.26121372	CLNS1A;AAMDC;RSF1;INTS4
	Amplification	15q26.3	chr15:97693069-99178266	3.14E-09	3.80E-06	15.83113456	IGF1R;SYNM;TTC23;ARRDC4;LINC00923;PGPEP1L;FAM169B;MIR4714;LINC01582;IRAIN;LUNAR1
	Amplification	16q12.1	chr16:47611775-48184086	0.00041404	0.00041404	15.30343008	PHKB;ABCC11;ABCC12;LOC100507534;LOC101927132

	Amplification	13q12.3	chr13:29410801-30909370	0.00031766	0.00031766	12.40105541	ALOX5AP;HMGB1;UBL3;SLC7A1;USPL1;MTUS2;KATNAL1;MEDAG;LINC00544;LINC00545;LINC00426;TEX26-AS1;LINC00572;LINC00297;MTUS2-AS1;LINC00365;LINC00398;LINC00384;LOC102723345;LINC01058
	Amplification	6q21	chr6:104901719-108228932	0.00034555	0.00034555	11.0817942	AIM1;PRDM1;PREP;NR2E1;SNX3;ATG5;BVES;SEC63;OSTM1;C6orf203;SOBP;QRSL1;PDSS2;BEND3;POPDC3;RTN4IP1;BVES-AS1;SCML4;LIN28B;MIR587;LIN28B-AS1;CD24;LOC100422737;LOC105377924
	Amplification	1q22	chr1:154911406-155283791	1.82E-21	0.00030295	75.64102564	CKS1B;CLK2;EFNA1;EFNA3;EFNA4;GBA;GBAP1;MTX1;MUC1;SHC1;THBS3;ADAM15;SCAMP3;PMVK;FAM189B;ZBTB7B;DPM3;LENEP;SLC50A1;PBXIP1;HCN3;TRIM46;FLAD1;PYGO2;DCST2;DCST1;KRTCAP2;MIR92B;MIR4258;LOC100505666;LOC101928120
	Amplification	1q21.3	chr1:150524381-150705580	5.99E-33	4.57E-20	74.61538462	ENSA;MCL1;ADAMTSL4;GOLPH3L;HORMAD1;ADAMTSL4-AS1;MIR4257
	Amplification	1q23.3	chr1:160940678-161292637	8.15E-23	7.87E-13	74.61538462	APOA2;FCER1G;NDUFS2;NIT1;PFDN2;PPOX;USF1;B4GALT3;DEDD;ADAMTS4;NR1I3;USP21;F11R;UFC1;NECTIN4;TOMM40L;KLHDC9;ITLN2;ARHGAP30;PCP4L1;TSTD1;MIR5187;LOC101928372
	Amplification	1q42.3	chr1:234335639-235258746	1.05E-14	1.66E-06	74.1025641	TARBP1;TOMM20;RBM34;ARID4B;IRF2BP2;COA6;SNORA14B;LINC01348;LINC00184;LINC01354;LINC01132;MIR4753;LOC101927765;LOC101927787;LOC101927851
	Amplification	1q44	chr1:242125994-248956422	1.36E-11	0.00038693	72.82051282	ADSS;HNRNPU;ZNF124;CEP170;AKT3;ZBTB18;SDCCAG8;TRIM58;AHCTF1;OR1C1;OR2M4;OR2L2;OR2L1P;OR2T1;DESI2;SCCPDH;KIF26B;ZNF692;ZNF695;TFB2M;SMYD3;ZNF669;ZNF672;SH3BP5L;OR2L5;OR2G3;OR2G2;OR2C3;EFCAB2;ZNF496;ZNF670;NLRP3;COX20;OR2M5;OR2M3;OR2T12;OR14C36;OR2T34;OR2T10;OR2T4;OR2T11;OR2B11;GCSAML;GCSAML-AS1;LINC01341;CNST;PLD5;C1orf100;OR2T6;LOC255654;C1orf101;PGBD2;OR2L13;OR14A16;VN1R5;LOC339529;OR6F1;OR2W3;OR2T8;OR2T3;OR2T29;C1orf229;OR2M1P;OR11L1;OR2L8;OR2AK2;OR2L3;OR2M2;OR2T33;OR2M7;OR2G6;OR2T2;OR2T5;OR14I1;OR2T27;OR2T35;OR2W5;OR13G1;LYPD8;LINC01347;MIR3124;MIR3916;ZNF670-ZNF695;MIR4677;LOC101928068;LOC101928226;SNORA100
	Amplification	8q24.21	chr8:127663843-127758305	1.89E-42	3.06E-31	67.94871795	MYC;CASC11
	Amplification	8q23.3	chr8:114289308-117502442	5.94E-22	1.23E-06	66.41025641	RAD21;TRPS1;EIF3H;UTP23;SLC30A8;AARD;RAD21-AS1;MIR3610;LINC00536;LOC105375713
	Amplification	8q22.3	chr8:100299138-100719913	9.39E-13	2.00E-06	64.35897436	RNF19A;PABPC1;ANKRD46;SNX31;MIR4471;MIR7705
	Deletion	8p21.2	chr8:20994458-30996516	0.00070288	0.00070292	56.92307692	ADRA1A;BMP1;POLR3D;BNIP3L;CHRNA2;CLU;DPYSL2;DUSP4;EGR3;DMTN;EPHX2;EXTL3;PTK2B;GFRA2;GNRH1;GSR;GTF2E2;LOXL2;NEFM;NEFL;NKX3-1;PNOC;PPP2CB;PPP2R2A;PPP3CC;SFTPC;STC1;FZD3;UBXN8;ADAM7;TNFRSF10D;TNFRSF10C;TNFRSF10B;TNFRSF10A;FGF17;DOK2;ENTPD4;PHYHIP;SORBS3;NPM2;DCTN6;PNMA2;ADAM28;RBPMS;XPO7;TRIM35;RHOBTB2;KIF13B;LEPROTL1;SLC39A14;ADAMDEC1;SLC25A37;SCARA3;SARAF;KCTD9;PIWIL2;ELP3;CCDC25;INTS9;HR;PBK;ZNF395;BIN3;TEX15;CCAR2;PDLIM2;EBF2;FAM160B2;HMBOX1;NUDT18;DOCK5;BIN3-IT1;REEP4;STMN4;CHMP7;NKX2-6;PEBP4;CDCA2;ESCO2;FBXO16;R3HCC1;LGI3;LOC254896;LOC286059;SCARA5;FAM183CP;LOC389641;NUGGC;MIR320A;C8orf58;LINC00589;MBOAT4;RBPMS-AS1;MIR548H4;MIR4287;MIR3148;MIR4288;MIR3622A;MIR3622B;LOC100507071;LOC100507156;SMIM18;LOC101929172;LOC101929237;LOC101929294;LOC101929315;EXTL3-AS1;LOC101929450;LOC101929470;MIR6841;MIR6842;MIR6843;MIR6876;MIR7641-2
	Deletion	8p23.2	chr8:2166023-6404670	9.59E-31	2.15E-28	55.38461538	CSMD1;LOC101927815
	Deletion	15q15.1	chr15:37090961-42943258	8.90E-17	2.65E-09	47.69230769	BUB1B;CAPN3;GANC;GCHFR;ITPKA;IVD;LTK;PLCB2;RAD51;SPINT1;SRP14;THBS1;TYRO3;JMJD7-PLA2G4B;SNAP23;RASGRP1;GPR176;CHP1;OIP5;BAHD1;MAPKBP1;RTF1;MGA;VPS39;TMEM87A;RPAP1;RPUSD2;EHD4;NDUFAF1;NUSAP1;SPTBN5;DLL4;INO80;PPP1R14D;HAUS2;RMDN3;DNAJC17;PAK6;KNL1;STARD9;VPS18;ZNF106;CHAC1;ZFYVE19;DISP2;C15orf57;KNSTRN;BMF;CHST14;PLA2G4E;TMCO5A;TTBK2;CDAN1;SPRED1;EXD1;FSIP1;RHOV;PLA2G4F;LRRC57;FAM98B;PLA2G4D;MRPL42P5;C15orf52;C15orf53;C15orf54;EIF2AK4;C15orf62;C15orf56;PHGR1;MIR626;MIR627;OIP5-AS1;SRP14-AS1;ANKRD63;JMJD7;PLA2G4B;MIR4310;INAFM2;RAD51-AS1;LOC101928227;EHD4-AS1;PLA2G4E-AS1;LOC105370792;LOC105370941;LOC105370943;BUB1B-PAK6
	Deletion	4p16.3	chr4:1-1221311	2.60E-06	2.51E-06	46.15384615	ATP5I;DGKQ;GAK;IDUA;MYL5;PDE6B;ZNF141;PCGF3;SPON2;CPLX1;SLC26A1;FGFRL1;PIGG;ABCA11P;MFSD7;TMEM175;ZNF595;ZNF721;ZNF718;CTBP1-AS;RNF212;ZNF876P;ZNF732;MIR571;LOC100129917;LOC100130872;TMED11P;LOC105374344
	Deletion	4q34.3	chr4:177990720-190214555	7.27E-16	5.11E-13	45.38461538	SLC25A4;CASP3;DCTD;F11;ACSL1;FAT1;FRG1;ING2;IRF2;KLKB1;MTNR1A;TLR3;SORBS2;FAM149A;PDLIM3;CLDN22;UFSP2;CDKN2AIP;TENM3;LRP2BP;STOX2;CFAP97;TRAPPC11;CENPU;WWC2;SNX25;LOC90768;ZFP42;ENPP6;WWC2-AS2;RWDD4;PRIMPOL;TRIML2;CCDC110;CYP4V2;F11-AS1;LOC339975;TRIML1;ANKRD37;LOC389247;HELT;LINC01060;FAM92A1P2;C4orf47;FRG2;SLED1;FLJ38576;LINC00290;LOC728175;MIR3945HG;CLDN24;DUX4;MIR1305;DBET;MIR3945;LINC01093;LOC100506272;MIR4455;WWC2-AS1;LINC01262;LOC102723766;LVCAT8;LOC105377590;LINC01596
	Deletion	5q11.2	chr5:58964471-60492158	2.66E-11	5.83E-11	45.38461538	PDE4D;PART1;MIR582
	Deletion	5q21.3	chr5:107006982-107859209	1.27E-08	4.59E-08	44.87179487	EFNA5
	Amplification	3q26.32	chr3:176971727-179951390	3.39E-10	0.00019031	44.35897436	ACTL6A;NDUFB5;PIK3CA;USP13;KCNMB2;KCNMB3;ZNF639;PEX5L;MFN1;MRPL47;GNB4;ZMAT3;TBL1XR1;LINC00578;LINC00501;LINC01014;LOC101928739;PEX5L-AS2;KCCAT211;KCNMB2-AS1
	Deletion	13q14.2	chr13:48301193-48490671	7.76E-24	4.69E-21	44.35897436	RB1;LPAR6
	Amplification	10p15.1	chr10:1-9155702	1.15E-06	1.15E-06	42.82051282	ADARB2;ATP5C1;CALML3;AKR1C4;KLF6;AKR1C1;AKR1C2;GATA3;GDI2;IDI1;IL2RA;IL15RA;ITIH2;PFKFB3;PFKP;PRKCQ;AKR1C3;NET1;PITRM1;ZMYND11;WDR37;KIN;DIP2C;LARP4B;GTPBP4;CALML5;ANKRD16;FAM208B;IDI2-AS1;SFMBT2;ASB13;TUBAL3;ITIH5;AKR1E2;TAF3;FBXO18;RBM17;IDI2;UCN3;LINC00700;LINC00705;AKR1C8P;TUBB8;AKR1C6P;LINC00200;LINC00701;LOC399715;GATA3-AS1;PRR26;PRKCQ-AS1;ADARB2-AS1;CALML3-AS1;LINC00704;MIR3155A;PITRM1-AS1;LINC00703;LINC00707;LINC00708;MIR4454;MIR3155B;LINC00702;LINC00706;MIR5699;LOC101927762;LOC101927964;LOC101928150;MIR6078;MIR7641-2;MIR6072;LOC102723376;LOC105376351;LOC105376360;LOC105376365;LOC105376382;LOC105376398;LOC105755953;SNORD142;SNORD129;LINP1
	Deletion	11q25	chr11:122422832-135086622	8.32E-10	8.40E-10	42.30769231	ACRV1;APLP2;CHEK1;ETS1;FLI1;HSPA8;STT3A;KCNJ1;KCNJ5;VWA5A;NFRKB;NRGN;OPCML;ST3GAL4;SRPRA;ST14;ZNF202;BARX2;EI24;FEZ1;ARHGAP32;ADAMTS8;IGSF9B;NCAPD3;VSIG2;OR8G2;OR8B8;OR8G1;OR8B2;ACAD8;B3GAT1;DCPS;ZBTB44;THYN1;DDX25;NTM;CDON;SPA17;SIAE;ROBO4;FOXRED1;SCN3B;CRTAM;PRDM10;GRAMD1B;PKNOX2;TP53AIP1;ROBO3;FAM118B;MSANTD2;CLMP;C11orf63;CCDC15;PUS3;JAM3;KIRREL3;RPUSD4;TBRG1;UBASH3B;SNORD14C;SNORD14D;SNORD14E;GLB1L2;ESAM;VPS26B;GLB1L3;TIRAP;PANX3;TMEM45B;PATE1;ADAMTS15;C11orf45;HYLS1;TMEM218;SLC37A2;OR8B12;OR8G5;OR10G8;OR10G9;OR10S1;OR6T1;OR4D5;SPATA19;HEPACAM;OR8D1;OR8D2;OR8B4;KIRREL3-AS3;C11orf44;MIR4697HG;LOC283177;TMEM225;OR8D4;LOC341056;BSX;OR6X1;OR6M1;OR10G4;OR10G7;OR8B3;OR8A1;PATE2;PATE4;ST3GAL4-AS1;SNX19;LOC403312;LINC00167;HEPN1;LOC646522;LOC100128239;PATE3;MIR3167;LOC100507283;SENCR;LOC100507431;LOC100507548;MIR4697;MIR4493;KIRREL3-AS2;NTM-IT;LOC101929340;LOC101929427;LOC101929473;LOC101929497;LOC101929517;LOC101929538;LINC01395;NTM-AS1;LOC101929653;MIR7641-2;MIR6090;MIR8052;LOC103611081;PKNOX2-AS1;STT3A-AS1
	Deletion	19p13.3	chr19:1103803-1943799	2.00E-27	2.00E-27	41.02564103	ATP5D;CIRBP;EFNA2;GAMT;GPX4;RPS15;STK11;TCF3;APC2;UQCR11;SBNO2;DAZAP1;MBD3;PCSK4;C19orf24;REXO1;ABHD17A;KLF16;MUM1;MIDN;REEP6;SCAMP4;ADAT3;PLK5;CIRBP-AS1;C19orf25;ATP8B3;CBARP;ADAMTSL5;NDUFS7;ONECUT3;MEX3D;LOC100288123;MIR1909

C2	Deletion	3p14.2	chr3:43617354-77042064	1.25E-08	1.25E-08	40	ACY1;ALAS1;AMT;APEH;ARF4;RHOA;ERC2-IT1;CACNA1D;SLC25A20;CAMP;CDC25A;CISH;CCR1;CCR3;CCR5;COL7A1;DAG1;DNASE1L3;DOCK3;DUSP7;CELSR3;FHIT;FLNB;GNAI2;GNAT1;XCR1;GPR27;GPX1;GRM2;HYAL1;IMPDH2;ITIH1;ITIH3;ITIH4;LAMB2;LTF;MAP4;MITF;MST1;MST1R;MYL3;CNTN3;PDHB;PFKFB4;PLXNB1;PRKAR2A;PRKCD;PTH1R;PTPRG;QARS;RPL29;ATXN7;SEMA3F;SMARCC1;NEK4;TCTA;TDGF1;TGM4;TKT;TMF1;CLEC3B;TNNC1;UBA7;USP4;UQCRC1;WNT5A;ZNF35;IFRD2;MAPKAPK3;SEMA3B;SLMAP;MANF;ACOX2;BAP1;HYAL3;CADPS;HYAL2;SUCLG2;HESX1;BSN;LIMD1;CCRL2;UBA3;RRP9;MAGI1;CACNA2D2;DCAF1;IP6K1;PSMD6;PARP3;ZNF197;RBM6;RBM5;NME6;TRAIP;ARIH2;ARL6IP5;NPRL2;CXCR6;CSPG5;CCR9;USP19;SLC38A3;CYB561D2;TMEM115;RPP14;FAM107A;WDR6;RASSF1;NISCH;TREX1;TUSC2;TWF2;DHX30;SACM1L;SCAP;LAMB2P1;EXOSC7;PDZRN3;RAD54L2;FRMD4B;STAB1;NBEAL2;FAM208A;KLHL18;LARS2;RYBP;NAT6;ABHD14A;POC1A;TMEM158;NDUFAF3;PTPN23;DNAH1;LRIG1;ERC2;APPL1;GNL3;FOXP1;SPCS1;SETD2;PRSS50;RBM15B;GMPPB;ARHGEF3;ABHD5;C3orf18;SHISA5;ZDHHC3;ZMYND10;TEX264;TMA7;ZNF589;HEMK1;IP6K2;SFMBT1;NCKIPSD;PHF7;TLR9;LZTFL1;P4HTM;SLC6A20;IL17RD;ELP6;QRICH1;PXK;FEZF2;EBLN2;DALRD3;SHQ1;PBRM1;CHDH;IL17RB;CACNA2D3;DCP1A;GLT8D1;ZKSCAN7;LMOD3;SEMA3G;KIF15;ADAMTS9;PCBP4;ABHD6;LRTM1;C3orf14;KIAA1143;SELK;PROK2;RNF123;KIF9;CDCP1;CCDC71;NT5DC2;SLC26A6;CAMKV;LRRC2;FYCO1;CCDC51;THOC7;WDR82;RTP3;LRRC2-AS1;ATRIP;NICN1;MON1A;KBTBD8;ABHD14B;UCN2;ZNF502;RFT1;ACTR8;SLC25A26;ZNF501;GPR62;FAM3D;TMEM42;IQCF1;GLYCTK;PPM1M;C3orf49;SNTN;SYNPR;LSMEM2;ASB14;FAM19A4;CCDC12;PPP4R2;PRICKLE2;C3orf67;KCTD6;KLHDC8B;LINC00870;DNAH12;PDE12;DENND6A;ALS2CL;TMIE;EOGT;FBXW12;LINC00877;CCDC66;TCAIM;ZNF852;ZNF660;KIF9-AS1;LINC00698;EIF4E3;CCDC36;PRSS42;ZNF445;SPATA12;TOPAZ1;C3orf62;TMEM110;PRSS45;AMIGO3;CDHR4;FAM212A;IQCF2;IQCF5;MUSTN1;IQCF3;LINC00960;MIRLET7G;MIR135A1;MIR138-1;MIR191;FAM19A1;TMEM89;IQCF6;SMIM4;MIR425;LIMD1-AS1;SPINK8;ARIH2OS;C3orf84;SNORD19;FAM86DP;SNORD69;MIR564;MIR566;GXYLT2;CCR2;ESRG;SNORD19B;LINC00696;SEMA3F-AS1;NRADDP;ZNF717;LOC100132146;BSN-AS2;PRSS46;LINC00994;FRG2C;MIR1284;MIR1324;MIR1226;MIR2115;MIR711;MIR3136;MIR4272;MIR4271;MIR4273;MIR3938;PRKAR2A-AS1;IQCF4;PTPRG-AS1;PSMD6-AS2;ADAMTS9-AS2;ABHD14A-ACY1;TMEM110-MUSTN1;MIR4793;MIR4787;MIR4444-1;MIR4443;PRICKLE2-AS1;RBM5-AS1;MIR5193;MAGI1-AS1;ITIH4-AS1;SYNPR-AS1;THOC7-AS1;ZNF197-AS1;GLYCTK-AS1;ARHGEF3-AS1;CACNA2D3-AS1;PRICKLE2-AS2;PRICKLE2-AS3;LARS2-AS1;FCF1P2;SUCLG2-AS1;SAMMSON;PDZRN3-AS1;LOC101927296;SEMA3B-AS1;IQCF5-AS1;LOC101929054;LOC101929159;C3orf67-AS1;ADAMTS9-AS1;RASSF1-AS1;MIR5787;MIR6823;MIR6824;MIR6872;MIR6890;MIR8064;LOC102724231;LOC102724297;CELSR3-
	Deletion	15q11.2	chr15:1-26972881	1.01E-14	8.87E-07	39.74358974	NBEAP1;GABRA5;GABRB3;IPW;NDN;SNRPN;UBE3A;MKRN3;PWAR5;SNURF;CYFIP1;NPAP1;MAGEL2;ATP10A;NIPA2;SNORD107;TUBGCP5;NIPA1;PWAR1;LOC283683;GOLGA6L2;OR4N4;HERC2P3;GOLGA6L1;GOLGA8IP;SNORD108;SNORD109A;SNORD115-1;WHAMMP3;SNORD64;PWAR4;PWARSN;LINC01193;IGHV1OR15-1;GOLGA8EP;OR4M2;OR4N3P;HERC2P2;NF1P2;GOLGA6L22;LINC00929;CHEK2P2;LOC646214;CXADRP2;IGHV1OR15-3;REREP3;GOLGA8S;GOLGA8J;GOLGA6L6;LOC727924;GOLGA8CP;PWRN1;PWRN2;SNORD116-1;SNORD116-2;SNORD116-3;SNORD116-4;SNORD116-5;SNORD116-8;SNORD116-10;SNORD116-11;SNORD116-12;SNORD116-13;SNORD116-14;SNORD116-15;SNORD116-16;SNORD116-17;SNORD116-18;SNORD116-20;SNORD116-21;SNORD116-22;SNORD116-23;SNORD116-24;SNORD116-25;SNORD115-2;SNORD116-26;SNORD116-27;SNORD115-3;SNORD115-4;SNORD115-5;SNORD115-6;SNORD115-7;SNORD115-8;SNORD115-10;SNORD115-11;SNORD115-12;SNORD115-13;SNORD115-14;SNORD115-15;SNORD115-16;SNORD115-17;SNORD115-18;SNORD115-20;SNORD115-21;SNORD115-22;SNORD115-23;SNORD115-25;SNORD115-26;SNORD115-29;SNORD115-30;SNORD115-31;SNORD115-32;SNORD115-33;SNORD115-34;SNORD115-35;SNORD115-36;SNORD115-37;SNORD115-38;SNORD115-39;SNORD115-41;SNORD115-44;SNORD116-28;SNORD116-29;SNORD115-48;SNORD115-24;SNORD115-27;SNORD115-28;SNORD115-45;SNORD115-47;LOC100128714;HERC2P7;GOLGA8DP;POTEB2;MIR1268A;MIR3118-3;MIR3118-4;MIR3118-2;MIR4509-1;MIR4509-2;MIR4508;MIR4715;MIR5701-2;MIR5701-1;SNORD116-30;SNORD115-46;POTEB;LOC101927079;PWRN3;POTEB3;PWRN4
	Amplification	8p11.21	chr8:42298500-42399085	2.37E-09	1.34E-05	38.71794872	IKBKB;POLB;VDAC3;DKK4
	Amplification	6p21.1	chr6:40744072-44300397	4.61E-07	6.49E-07	37.43589744	BYSL;CCND3;SLC29A1;GUCA1A;GUCA1B;HSP90AB1;MDFI;MEA1;NFKBIE;NFYA;PEX6;PGC;POLH;PPP2R5D;PTK7;PRPH2;SRF;TBCC;VEGFA;TFEB;NCR2;MED20;POLR1C;MAD2L1BP;CUL7;DNPH1;CNPY3;FRS3;SLC22A7;APOBEC2;CAPN11;CUL9;UBR2;GLTSCR1L;ZNF318;YIPF3;USP49;GNMT;PRICKLE4;MRPL2;TREM2;TREM1;GTPBP2;MRPS18A;MRPS10;TMEM63B;TRERF1;AARS2;XPO5;MRPL14;DLK2;TREML2;TTBK1;RRP36;ABCC10;KLC4;TJAP1;FOXP4;KLHDC3;TAF8;PTCRA;TCTE1;C6orf223;RSPH9;LRRC73;TREML5P;ADCY10P1;OARD1;TSPO2;UNC5CL;TREML4;RPL7L1;TREML1;TREML3P;SLC35B2;LOC401261;CRIP3;C6orf226;TMEM151B;ATP6V0CP3;C6orf132;LINC01512;TOMM6;MIR4647;MIR4641;FOXP4-AS1;LOC101929555;LOC101929705;LOC101929726;MIR6780B;LINC01276;CNPY3-GNMT
	Amplification	19q12	chr19:29267133-30037195	3.65E-14	1.43E-13	36.92307692	CCNE1;URI1;POP4;PLEKHF1;C19orf12;LOC284395;VSTM2B
	Deletion	10q23.31	chr10:87746731-87861303	4.82E-26	6.70E-05	36.92307692	ATAD1;CFL1P1
	Deletion	1p36.22	chr1:1-18112435	1.08E-20	3.18E-16	36.66666667	hsa-mir-551a;RERE;CA6;CASP9;TNFRSF8;CDK11B;CLCN6;CLCNKA;CLCNKB;CORT;DFFA;DFFB;DVL1;MEGF6;EPHA2;ENO1;MTOR;GABRD;GNB1;ZBTB48;TNFRSF9;MFAP2;MTHFR;NPPA;NPPB;PEX10;PEX14;PGD;PIK3CD;PLOD1;EXOSC10;PRKCZ;RNU1-4;RPL22;RSC1A1;SCNN1D;SDHB;SKI;SLC2A5;SRM;TNFRSF1B;TP73;TNFRSF4;ZBTB17;PRDM2;MMP23B;MMP23A;KCNAB2;TNFRSF25;TNFRSF14;TNFRSF18;PER3;DHRS3;VAMP3;H6PD;ISG15;PLCH2;CROCC;CEP104;KLHL21;SLC35E2;MFN2;ANGPTL7;UBE4B;MAD2L2;PDPN;MASP2;UTS2;RER1;MST1P2;MST1L;PADI2;PARK7;CTRC;ACOT7;CLSTN1;SPEN;KIF1B;PLEKHM2;KAZN;CAMTA1;DNAJC16;ATP13A2;TARDBP;ICMT;PADI4;CHD5;SZRD1;NOC2L;FBXO2;FBXO6;RNU6-1;RNU5E-1;RNU1-3;RNU1-2;RNU1-1;HSPB7;ARHGEF16;SSU72;UBIAD1;PADI1;WRAP73;SLC45A1;CELA2B;SDF4;PADI3;ERRFI1;FBXO42;MXRA8;HES2;FBLIM1;CASZ1;CPSF3L;C1orf159;AURKAIP1;MRPL20;TMEM51;ARHGEF10L;VPS13D;ATAD3A;PANK4;NBPF1;NECAP2;DNAJC11;RCC2;AJAP1;CTNNBIP1;AGTRAP;TP73-AS1;PLEKHG5;LRRC47;DISP3;HES4;MIIP;CELA2A;PRDM16;NMNAT1;VWA1;PRAMEF1;PRAMEF2;NADK;EFHD2;MMEL1;RSG1;OR4F5;NOL9;AGMAT;LINC00115;MORN1;GPR157;SPSB1;CPTP;TAS1R1;OR4F16;ACTL8;CCNL2;ESPN;TAS1R3;ATAD3B;PLEKHN1;SLC25A33;DDI2;LZIC;PERM1;CROCCP2;CFAP74;KIAA2013;THAP3;C1orf158;FBXO44;CROCCP3;FHAD1;LOC115110;RBP7;ACAP3;UBE2J2;LRRC38;AADACL3;PUSL1;B3GALT6;TPRG1L;FAM213B;ARHGEF19;ACTRT2;MIB2;C1orf127;SAMD11;LOC148413;PHF13;LINC00337;CCDC27;C1orf64;SLC2A7;CALML6;TMEM201;FAAP20;TMEM51-AS1;ATAD3C;LINC01342;TTLL10;NPHP4;C1orf167;FAM41C;LOC284661;SLC25A34;ESPNP;C1orf174;KLHL17;TMEM240;TMEM52;AADACL4;PRAMEF5;HNRNPCL1;PRAMEF10;FAM131C;PADI6;DRAXIN;SPATA21;AGRN;CENPS;GPR153;FAM132A;HES5;SMIM1;RNF207;TMEM82;HES3;PRAMEF12;PRAMEF8;PRAMEF18;PRAMEF17;FAM87B;PRAMEF4;PRAMEF13;RNF223;MIR200A;MIR200B;MIR34A;LINC00982;PRAMEF11;PRAMEF6;HNRNPCL2;UQCRHL;ANKRD65;PRAMEF7;PRAMEF25;MIR429;LINC01128;TMEM88B;FNDC10;PIK3CD-AS1;PRAMEF26;PRAMEF33P;PRAMEF19;PRAMEF20;FAM138A;PRAMEF34P;HNRNPCL3;PRAMEF22;PRAMEF15;WASH7P;FAM138C;FAM138D;SNORA59A;MIR551A;C1orf195;CDK11A;SLC35E2B;LINC01346;PRAMEF14;FAM231A;FAM231C;FLJ37453;LOC729737;OR4F29;LOC100129534;LOC100130417;LOC100132062;LOC100132147;FAM231B;LOC100133331;LINC01134;DDX11L1;TTC34;LOC100288069;MIR1302-2;NPPA-AS1;MIR1302-9;MIR1302-10;MIR1302-11;MIR4251;MIR1273D;MIR4252;MIR3675;LOC100505887;ENO1-AS1;LOC100506022;APITD1-CORT;MIR3972;MIR4689;MIR5096;MIR4632;MIR4417;MIR5697;MTOR-AS1;LOC100996583;HNRNPCL4;LOC101927876;LOC101928626;PIK3CD-AS2;LOC101929181;PRAMEF27;RNU6-7;RNU6-8;MIR6723;MIR6726;MIR6727;MIR6728;MIR7846;MIR6859-2;MIR6859-3;MIR6730;MIR6808;MIR6859-1;MIR6729;LOC102724312;LOC102724450;LOC102724539;LOC102724552;LOC102724571;LOC102724659;RNU6-2;LOC105376736;LOC105376805;LOC105378591;MIR34AHG;SNORD128
	Amplification	12p13.33	chr12:1-1182100	9.75E-09	1.78E-07	35.8974359	NINJ2;RAD52;KDM5A;SLC6A12;SLC6A13;ERC1;WNK1;CCDC77;B4GALNT3;IQSEC3;LOC574538;FAM138D;LOC100049716;LOC100288778;LOC101929384;LOC102723544;LOC105369595

	Deletion	10q23.31	chr10:87855381-88274281	6.00E-34	2.35E-26	35.8974359	PTEN;KLLN
	Deletion	6q26	chr6:155944963-170805979	4.45E-15	4.50E-15	35.64102564	ACAT2;CCR6;GPR31;IGF2R;KIF25;LPA;MAS1;MAP3K4;AFDN;PARK2;PDCD2;PLG;PSMB1;RPS6KA2;SLC22A1;SLC22A3;SLC22A2;SOD2;T;TBP;TCP1;TCP10;TCTE3;DYNLT1;THBS2;EZR;RNASET2;SYNJ2;QKI;WTAP;PDE10A;FGFR1OP;LINC01558;DLL1;MRPL18;SNX9;MPC1;UNC93A;PHF10;ERMARD;AGPAT4;TULP4;ARID1B;TMEM181;SMOC2;ZDHHC14;FRMD1;AGPAT4-IT1;LINC00574;LPAL2;RSPH3;TTLL2;FAM120B;FNDC1;SERAC1;LINC00473;SYTL3;SFT2D1;TAGAP;PACRG;PNLDC1;LOC154449;DACT2;C6orf118;OSTCP1;WDR27;PACRG-AS1;PRR18;LOC285804;C6orf120;DKFZp451B082;TCP10L2;LOC401286;LINC00242;LINC01624;GTF2H5;LINC00602;LOC441178;AFDN-AS1;SNORA20;SNORA29;TMEM242;LOC729603;PACRG-AS3;HGC6.3;LOC100129518;C6orf99;LOC100131532;AIRN;LOC100289495;MIR1913;MIR1202;MIR3918;MIR3939;MIR3692;KIF25-AS1;CAHM;MIR4466;MIR4644;RPS6KA2-AS1;SYNJ2-IT1;RPS6KA2-IT1;EZR-AS1;LOC101929122;PACRG-AS2;LOC101929297;LOC101929420;LOC101929460;LINC01615;LOC101929504;LOC101929523;MIR7161;MIR7641-2;LOC102724053;LOC102724152;LOC102724357;LOC102724511;LOC105378098;MEAT6;LOC105378123;LOC105378127;LOC105378137;LOC105378146
	Deletion	11p15.5	chr11:1-748945	2.35E-07	2.36E-07	35.38461538	DRD4;HRAS;IRF7;PSMD13;RNH1;SCT;RASSF7;IFITM1;IFITM3;DEAF1;IFITM2;PKP3;SIRT3;BET1L;CDHR5;PHRF1;SIGIRR;RIC8A;EPS8L2;PGGHG;PTDSS2;ODF3;LRRC56;LOC143666;SCGB1C1;NLRP6;LMNTD2;TMEM80;ANO9;B4GALNT4;IFITM5;MIR210;LINC01001;MIR210HG;MIR6743
	Amplification	17q23.1	chr17:59841010-60136601	7.66E-10	2.02E-07	33.58974359	RPS6KB1;RNFT1;TUBD1;HEATR6;VMP1;MIR21;WFDC21P;TBC1D3P1-DHX40P1;LOC653653;MIR4737;LOC101927755;LOC105371849
	Amplification	8p11.23	chr8:38286685-38379371	4.13E-20	1.10E-09	32.82051282	WHSC1L1
	Amplification	11q13.3	chr11:70074912-70207380	5.66E-18	3.46E-16	32.56410256	FADD;ANO1;ANO1-AS2
	Deletion	12q13.13	chr12:44388547-55903840	7.86E-07	7.55E-07	32.05128205	ASIC1;ACVR1B;ACVRL1;ADCY6;AMHR2;AQP2;AQP5;AQP6;ARF3;ATF1;ATP5G2;CACNB3;CCNT1;CD63;COL2A1;EIF4B;CELA1;BLOC1S1;GPD1;NCKAP1L;NR4A1;HNRNPA1;HOXC4;HOXC5;HOXC6;HOXC8;HOXC9;HOXC10;HOXC11;HOXC12;HOXC13;IGFBP6;ITGA5;ITGA7;ITGB7;KRT1;KRT2;KRT3;KRT4;KRT5;KRT6A;KRT6B;KRT7;KRT8;KRT18;KRT81;KRT82;KRT83;KRT84;KRT85;KRT86;LALBA;MMP19;NELL2;NFE2;SLC11A2;PCBP2;PDE1B;PFDN5;PFKM;POU6F1;PPP1R1A;PRKAG1;PRPH;RARG;RDH5;SCN8A;SMARCD1;SP1;TARBP2;TMBIM6;TFCP2;VDR;WNT1;WNT10B;MAP3K12;TUBA1A;KMT2D;AAAS;SOAT2;NPFF;ENDOU;KRT75;SCAF11;DDX23;SLC4A8;ESPL1;DAZAP2;TESPA1;TROAP;GDF11;TUBA1B;RAPGEF3;MCRS1;ATF7;GALNT6;COPZ1;FAIM2;DDN;TNS2;KCNH3;CBX5;ANP32D;SMUG1;PRPF40B;METTL7A;LETMD1;ZNF385A;OR7E47P;RND1;ORMDL2;RACGAP1;SENP1;DHH;PLEKHA8P1;FKBP11;KRT76;CSAD;BIN2;LIMA1;HDAC7;GPR84;SLC38A2;PRR13;KANSL2;SLC38A4;SLC48A1;LMBR1L;SMAGP;DIP2B;CALCOCO1;NCKAP5L;NEUROD4;C12orf10;ATG101;SPATS2;TMEM106C;RPAP3;DNAJC22;SLC38A1;CSRNP2;RACGAP1P;FAM186B;SARNP;TUBA1C;SPRYD3;MFSD5;COX14;DNAJC14;CCDC65;LACRT;FMNL3;CERS5;PCED1B;KRT71;LARP4;DCD;MUCL1;FAM186A;OR2AP1;OR10P1;RHEBL1;C12orf54;ZNF641;OR10AD1;SP7;GTSF1;OR10A7;KRT74;ASB8;KRT72;BCDIN3D;KRT80;GRASP;KRT78;METTL7B;ANO6;ARID2;OR6C74;OR6C3;LINC00935;LOC283332;LOC283335;ZNF740;OR6C6;C12orf80;LINC00592;TMPRSS12;KRT6C;KRT73;C1QL4;KRT79;ANKRD33;OR6C2;OR6C4;H1FNT;OR8S1;AMIGO2;KRT77;CCDC184;OR6C1;OR6C75;OR6C76;OR6C70;LINC00938;LOC400043;FIGNL2;OR6C65;OR6C68;MIR196A2;DBX2;FLJ12825;TMEM198B;OR9K2;MIR148B;HIGD1C;GLYCAM1;SNORA2A;SNORA2B;SNORA2C;MIR615;HOTAIR;KRT73-AS1;PCED1B-AS1;LOC100240734;LOC100240735;BCDIN3D-AS1;LOC100288798;MIR1293;MIR1291;LOC100335030;LOC100506125;BLOC1S1-RDH5;MIR4701;MIR3198-2;MIR4494;MIR4698;LOC100652999;HOXC-AS1;HOXC-AS2;HOXC-AS3;HOXC13-AS;LOC101927267;LOC101927292;LOC101927318;PCBP2-OT1;CISTR;MIR6757;MIR6505;LOC102724050;LOC105369747;LOC105369971;SNORD133
	Deletion	2q37.3	chr2:236581262-242193529	4.15E-12	5.85E-05	32.05128205	AGXT;KIF1A;BOK;COL6A3;DTYMK;GPC1;GPR35;HDLBP;NDUFA10;SEPT2;PDCD1;PPP1R7;PER2;LRRFIP1;HDAC4;FARP2;RAMP1;STK25;COPS8;CAPN10;PASK;ATG4B;SNED1;TRAF3IP1;ANO7;PRLH;THAP4;ANKMY1;SCLY;ASB1;HES6;RNPEPL1;GAL3ST2;RAB17;MLPH;C2orf54;ILKAP;ING5;MGC16025;LOC93463;TWIST2;NEU4;MTERF4;UBE2F;OTOS;COPS9;OR6B3;LOC150935;LINC01107;LOC151174;ERFE;LOC200772;RTP5;LOC285095;DUSP28;ESPNL;RBM44;AQP12A;KLHL30;OR6B2;FLJ43879;MIR149;LOC643387;AQP12B;D2HGDH;LOC728323;PP14571;BOK-AS1;MIR3133;MIR4269;UBE2F-SCLY;MIR2467;MIR4440;MIR4786;MIR4441;CAPN10-AS1;LINC01237;LOC101928111;MIR6811;LOC102723927
	Amplification	12p13.2	chr12:11886820-12022919	5.93E-08	1.19E-06	31.79487179	ETV6
	Amplification	8p11.23	chr8:37629559-37708883	7.05E-16	0.0002295	31.02564103	ZNF703
	Deletion	18q23	chr18:63934133-80373285	0.00012871	0.00013424	30.76923077	CDH7;CYB5A;GALR1;MBP;NFATC1;SERPINB8;ZNF236;CTDP1;SOCS6;ZNF516;TSHZ1;CD226;TXNL4A;ADNP2;RTTN;KCNG2;SALL3;CDH19;TIMM21;TMX3;ZNF407;CNDP2;CCDC102B;RBFA;PQLC1;NETO1;PARD6G;CNDP1;DSEL;FAM69C;CBLN2;FBXO15;GTSCR1;DOK6;LINC00305;LOC284241;ZADH2;SMIM21;LINC00908;HMSD;LOC284294;LOC339298;ATP9B;LINC01538;LOC400655;LINC00909;C18orf65;LINC00683;LOC400661;HSBP1L1;LOC643542;C18orf63;PARD6G-AS1;LOC100131655;LINC01541;LOC100505797;LOC100505817;LOC100505853;RBFADN;MIR5011;MIR548AV;LOC101060542;LOC101927481;LOC101927606;LOC101927651;LINC01029;LOC101927989;LOC102724913;LOC105372179
	Deletion	2q37.1	chr2:231891819-238106096	6.53E-12	0.0009195	30.76923077	ALPI;ALPP;ALPPL2;CHRND;CHRNG;COL6A3;GBX2;INPP5D;KCNJ13;NEU2;NPPC;SAG;SPP2;DGKD;LRRFIP1;ECEL1;EIF4E2;ARL4C;RAMP1;COPS8;SH3BP4;NGEF;GIGYF2;PRLH;SCLY;UGT1A10;UGT1A8;UGT1A7;UGT1A6;UGT1A5;UGT1A9;UGT1A4;UGT1A1;UGT1A3;ATG16L1;USP40;HJURP;ACKR3;RAB17;TRPM8;MLPH;IQCA1;EFHD1;LOC93463;AGAP1;DIS3L2;UBE2F;MSL3P1;TIGD1;MROH2A;ECEL1P2;RBM44;C2orf82;ASB18;DNAJB3;PRSS56;SCARNA6;SCARNA5;MIR562;LOC100286922;MIR1471;AGAP1-IT1;UBE2F-SCLY;MIR5001;LOC101928881;MIR6811;LINC01173
	Deletion	9p21.3	chr9:21555228-22448738	3.68E-16	3.68E-16	30.25641026	CDKN2A;CDKN2B;MTAP;CDKN2A-AS1;CDKN2B-AS1
	Deletion	10q26.3	chr10:128316503-133797422	1.51E-05	1.51E-05	29.74358974	ADAM8;BNIP3;CYP2E1;ECHS1;INPP5A;MGMT;UTF1;GLRX3;DPYSL4;TUBGCP2;VENTX;CALY;CFAP46;PPP2R2D;LRRC27;ADGRA1;NKX6-2;KNDC1;MTG1;SYCE1;PRAP1;ZNF511;CTAGE7P;C10orf91;PWWP2B;PAOX;EBF3;TCERG1L;FUOM;JAKMIP3;STK32C;LINC00959;LINC01164;LINC01168;SPRNP1;FRG2B;SPRN;MIR202;SCART1;ADGRA1-AS1;DUX4;MIR378C;MIR4297;MIR3944;TCERG1L-AS1;LINC01166;MIR202HG;LINC01167
	Amplification	6q21	chr6:106760428-106988903	1.05E-07	4.79E-07	28.20512821	MIR587;CD24;LOC100422737
	Amplification	11q14.1	chr11:77857554-78158081	4.54E-10	6.66E-08	27.94871795	NDUFC2;THRSP;AAMDC;KCTD14;ALG8;INTS4;KCTD21-AS1;NDUFC2-KCTD14
	Amplification	11p13	chr11:34617738-34835777	7.42E-06	7.81E-06	25.38461538	EHF
	Amplification	6q23.3	chr6:134959125-135279835	8.80E-07	4.09E-06	24.1025641	MYB;HBS1L;MIR548A2;MIR3662
	Amplification	17q12	chr17:39610187-39743434	2.42E-44	2.42E-44	23.07692308	ERBB2;GRB7;PNMT;TCAP;STARD3;PPP1R1B;MIEN1;PGAP3;MIR4728
	Amplification	13q34	chr13:109072009-114364328	6.46E-06	6.46E-06	22.30769231	ATP4B;COL4A1;COL4A2;F7;F10;GAS6;ING1;LAMP1;GRK1;SOX1;TFDP1;CUL4A;IRS2;PROZ;ARHGEF7;CDC16;TUBGCP3;RASA3;MYO16;ATP11A;MCF2L;TMCO3;DCUN1D2;ANKRD10;RAB20;NAXD;PCID2;UPF3A;CARS2;GRTP1;ADPRHL1;TEX29;SPACA7;LINC00567;LINC00346;CHAMP1;TMEM255B;ATP11AUN;LINC00452;GAS6-AS1;COL4A2-AS2;LINC00552;MCF2L-AS1;LINC00403;LOC100506016;GAS6-AS2;MIR4502;MIR548AR;LINC00565;GRTP1-AS1;LINC00454;COL4A2-AS1;ATP11A-AS1;ARHGEF7-AS1;ARHGEF7-AS2;MYO16-AS1;LOC101060553;LINC00676;LINC00368;LINC00354;LINC01070;LOC101928730;LINC01043;LOC101928841;MIR8073;MIR8075;LINC01044;LINC00399;LINC00431;LINC00396;LINC01054;F10-AS1;LOC105370369
	Amplification	15q26.3	chr15:92435263-101991189	1.30E-05	1.30E-05	22.30769231	ALDH1A3;CHD2;IGF1R;MEF2A;PCSK6;SNRPA1;NR2F2;HSP90B2P;ST8SIA2;CHSY1;SYNM;OR4F4;LINS1;MCTP2;VIMP;RGMA;TTC23;LRRK1;TM2D3;ARRDC4;LINC00923;TARSL2;LRRC28;ASB7;LYSMD4;PGPEP1L;LINC00924;C15orf32;SPATA8;ADAMTS17;DNM1P46;CERS3;LINC01579;FAM169B;WASH3P;SPATA41;OR4F6;OR4F15;OR4F13P;FAM174B;LINC01197;LOC400464;LOC440311;PRKXP1;NR2F2-AS1;ASB9P1;FAM138E;LOC100128108;LINC00930;DDX11L9;MIR1469;MIR1302-2;MIR1302-9;MIR1302-10;MIR1302-11;MIR3175;LINC01578;LOC100507472;MIR4714;SPATA8-AS1;LINC01581;LINC01580;LOC101927153;LOC101927286;LOC101927310;LINC01582;LOC101927751;MIR6859-2;MIR6859-3;MIR6859-1;CERS3-AS1;LOC102723335;IRAIN;LUNAR1;LOC104613533;PCSK6-AS1
	Amplification	17q21.33	chr17:49209927-49544309	5.51E-05	0.0002295	22.05128205	GNGT2;NGFR;PHB;ZNF652;ABI3;FLJ40194;PHOSPHO1;LOC100288866;LOC101927207;MIR6129;MIR6165;LOC102724596
	Deletion	7q36.1	chr7:152120330-152438989	2.33E-11	2.34E-11	19.74358974	KMT2C
	Deletion	6p25.3	chr6:1-3269936	1.98E-05	1.90E-05	18.97435897	BPHL;SERPINB1;FOXF2;FOXC1;GMDS;IRF4;NQO2;SERPINB6;SERPINB9;TUBB2A;RIPK1;EXOC2;WRNIP1;DUSP22;FOXQ1;HUS1B;LINC01600;SERPINB9P1;LOC285766;LINC01622;MYLK4;TUBB2B;PSMG4;LINC01011;HTATSF1P2;LINC00266-3;LOC100507194;GMDS-AS1;MIR4645;LOC101927691;FOXCUT;LOC101927730;LOC101927759;MIR6720

	Deletion	20p13	chr20:1-960668	0.00094831	0.0009195	18.46153846	CSNK2A1;SOX12;TCF15;RBCK1;ANGPT4;TRIB3;NRSN2;DEFB126;FAM110A;ZCCHC3;SCRT2;SLC52A3;TBC1D20;C20orf96;SRXN1;DEFB127;DEFB129;DEFB125;DEFB128;DEFB132;NRSN2-AS1
	Deletion	16p13.3	chr16:3714792-3948259	0.00080811	0.00078331	18.20512821	CREBBP
	Amplification	1q32.1	chr1:202275395-206037049	4.73E-05	4.73E-05	84.70149254	ADORA1;ATP2B4;CHI3L1;CHIT1;CTSE;ELK4;FMOD;KISS1;MDM4;MYBPH;MYOG;PPP1R12B;CDK18;PIK3C2B;PRELP;RABIF;RBBP5;REN;SNRPE;CNTN2;BTG2;PPFIA4;RAB29;SOX13;ZC3H11A;TMCC2;LRRN2;KDM5B;PLEKHA6;NFASC;DSTYK;OPTC;UBE2T;ADIPOR1;CYB5R1;LAX1;KLHDC8A;ETNK2;KLHL12;LGR6;NUCKS1;NUAK2;LINC00260;PPP1R15B;SLC45A3;TMEM183A;LEMD1;SLC26A9;SYT2;LINC00628;GOLT1A;LOC148709;MFSD4A;PM20D1;SLC41A1;LINC00303;LEMD1-AS1;LOC284578;LOC284581;RAB7B;TMEM81;MIR135B;MGAT4EP;SNORA77;LINC01136;ZBED6;PCAT6;LOC100506747;LINC01353;BLACAT1;LOC101929441;LOC103021295;LOC1030212
	Amplification	1q21.3	chr1:119984773-150973130	3.77E-07	3.05E-06	75	ARNT;BCL9;CTSK;CTSS;DRD5P2;ECM1;ENSA;FCGR1A;FCGR1B;FMO5;GJA5;GJA8;MCL1;NOTCH2;PDZK1;PRKAB2;RNU1-4;HIST2H2AA3;HIST2H2AC;HIST2H2BE;HIST2H4A;ITGA10;PEX11B;PRPF3;SEC22B;CHD1L;PDE4DIP;SETDB1;SV2A;RBM8A;SF3B4;PIAS3;POLR3C;TXNIP;MTMR11;CD160;VPS45;RPRD2;CA14;NBPF14;RNVU1-7;RNU1-3;RNU1-2;RNU1-1;RNF115;CERS2;BOLA1;APH1A;PLEKHO1;ACP6;GPR89B;MRPS21;ADAMTSL4;GOLPH3L;OTUD7B;LINC00869;C1orf54;TARS2;ANP32E;HORMAD1;POLR3GL;GNRHR2;LIX1L;CIART;HFE2;ANKRD35;NBPF12;NUDT4P2;PDIA3P1;NBPF11;NUDT17;NBPF15;ANKRD34A;HIST2H2AB;HIST2H3A;HIST2H2BC;HIST2H2BA;NOTCH2NL;LINC01138;LOC388692;NBPF9;NUDT4P1;HIST2H2BF;FAM72C;ADAMTSL4-AS1;PPIAL4G;FAM231D;NBPF13P;PPIAL4D;LOC645166;EMBP1;SRGAP2B;SRGAP2C;PPIAL4A;GPR89A;PPIAL4C;HIST2H3D;FAM72B;FAM72D;NBPF8;LINC00623;LOC728989;PPIAL4E;PFN1P2;PDZK1P1;LOC100132057;NBPF10;FCGR1CP;NBPF20;HYDIN2;LINC00624;MIR4257;LOC100505824;MIR5087;SRGAP2-AS1;FALEC;SRGAP2D;RNVU1-8;LOC101927429;LOC101927468;LOC101928979;NBPF25P;LOC101929798;RNVU1-4;RNVU1-14;RNVU1-15;RNVU1-20;RNVU1-17;RNVU1-3;RNVU1-1;RNVU1-6;RNVU1-19;MIR6878;MIR6736;MIR6077;CH17-408M7.1;LOC103091866;LOC105371433
	Deletion	17p12	chr17:11993313-12552764	5.79E-07	2.95E-06	75	MAP2K4;MIR744
	Amplification	8q24.21	chr8:127395199-127932836	7.95E-19	7.37E-11	72.01492537	MYC;POU5F1B;PVT1;CASC8;CASC11;MIR1204;CCAT2
	Deletion	11q23.2	chr11:104607791-120520763	6.15E-12	1.73E-09	72.01492537	ACAT1;APOA1;APOA4;APOC3;ARCN1;ATM;FXYD2;CXCR5;CASP1;CASP4;CASP5;CBL;CD3D;CD3E;CD3G;CRYAB;DDX6;DDX10;DLAT;DPAGT1;DRD2;FDX1;SLC37A4;GRIA4;GUCY1A2;H2AFX;HMBS;HSPB2;HTR3A;IL10RA;IL18;MCAM;KMT2A;NCAM1;NNMT;NPAT;PAFAH1B2;POU2AF1;PPP2R1B;PTS;NECTIN1;RDX;RPS25;SCN2B;SCN4B;SDHD;SLN;TAGLN;THY1;UPK2;ZBTB16;CUL5;ZPR1;USP2;PCSK7;HTR3B;ZW10;UBE4A;C2CD2L;RBM7;MPZL2;HYOU1;ATP5L;TREH;CEP164;EXPH5;PHLDB1;SIK2;ARHGEF12;SIK3;BACE1;TRIM29;CADM1;POU2F3;HINFP;REXO2;TIMM8B;SIDT2;TRAPPC4;FXYD6;C11orf71;SLC35F2;RAB39A;BTG4;NXPE4;TTC12;C11orf57;ELMOD1;VPS11;TEX12;TMPRSS4;IFT46;DSCAML1;ARHGAP20;USP28;CARD18;AASDHPPT;ABCG4;C11orf1;RNF26;NLRX1;ALG9;PDZD3;TMPRSS5;BCO2;TMPRSS13;MSANTD4;BUD13;TMEM25;DIXDC1;ZC3H12C;ALKBH8;FDXACB1;C11orf52;CARD16;C1QTNF5;APOA5;COLCA2;PIH1D2;NXPE1;NXPE2;JAML;KBTBD3;CWF19L2;KDELC2;LAYN;TTC36;C11orf65;MPZL3;TMEM136;OAF;ANKK1;RNF214;LOC283140;LINC00900;BCL9L;FOXR1;CCDC153;CCDC84;C11orf53;PLET1;LOC387810;C11orf87;COLCA1;C11orf88;MIR34B;MIR34C;CASP1P2;CARD17;LOC643733;LOC643923;CLDN25;LOC649133;RPL23AP64;LOC100131626;LOC100132078;LOC100132686;NCAM1-AS1;BACE1-AS;MIR4301;USP2-AS1;MIR3656;CASP12;TMPRSS4-AS1;HSPB2-C11orf52;FXYD6-FXYD2;MIR4491;MIR4492;LOC100652768;LOC101928535;LOC101928823;LOC101928847;LOC101928940;LOC101928985;LOC101929011;LOC101929089;MIR6756;MIR6716;LOC102724301;APOA1-AS;LOC105369473;LOC105369486;LOC105369507;LOC105369509
	Amplification	8q22.3	chr8:99628117-102067183	3.85E-12	6.69E-05	71.64179104	COX6C;POLR2K;SPAG1;YWHAZ;RNF19A;RGS22;PABPC1;ZNF706;GRHL2;NACAP1;NCALD;ANKRD46;VPS13B;SNX31;FBXO43;FLJ42969;MIR1273A;MIR4471;MIR7705;LOC104054148
	Amplification	8q23.3	chr8:115368839-116276444	1.28E-18	1.74E-07	70.89552239	TRPS1;LINC00536
	Deletion	1p36.11	chr1:16470248-202135967	2.42E-20	1.21E-05	68.65671642	hsa-mir-3119-1;ABCA4;ABL2;ACADM;ADAR;ADORA3;AGL;AK2;AK4;ALDH9A1;ALPL;ALX3;AMPD1;AMPD2;AMY1A;AMY1B;AMY1C;AMY2A;AMY2B;APCS;APOA2;FASLG;RHOC;ARNT;ASTN1;SERPINC1;ATP1A1;ATP1A2;ATP1A4;ATP1B1;ATP5F1;ATP6V0B;ADGRB2;BCL9;BGLAP;BMP8B;BRDT;C1QA;C1QB;C1QC;C8A;C8B;CACNA1E;CACNA1S;CAPZA1;CAPZB;CASQ1;CASQ2;RUNX3;CD1A;CD1B;CD1C;CD1D;CD1E;CD2;CD247;CD5L;CD48;CD53;CD58;CDA;CDC20;CDC42;CDKN2C;CD52;RCC1;CHI3L2;CHRNB2;CKS1B;CLCA1;CLK2;PLK3;CNN3;CNR2;COL8A2;COL9A2;COL11A1;COL16A1;COPA;CPT2;CRABP2;CRP;CRYZ;CSF1;CSF3R;CSRP1;CTBS;CTH;CTPS1;CTSK;CTSS;CYP2J2;CYP4A11;CYP4B1;DAB1;DBT;GADD45A;DDOST;DHX9;DHCR24;DIO1;DLSTP1;DPH2;DPT;DPYD;DR1;DRD5P2;E2F2;ECE1;ECM1;S1PR1;EDN2;PHC2;EFNA1;EFNA3;EFNA4;CELSR2;ELAVL4;ELF3;ENSA;EPB41;EPHA8;EPHB2;EPS15;ETV3;EXTL1;EXTL2;EYA3;F3;F5;F13B;FAAH;FABP3;FCER1A;FCER1G;FCGR1A;FCGR1B;FCGR2A;FCGR2B;FCGR3A;FCGR3B;FDPS;FGR;FHL3;FOXE3;FOXD2;FLG;FMO1;FMO2;FMO3;FMO4;FMO5;NR5A2;FUCA1;ACKR1;IFI6;GALE;GBA;GBAP1;GBP1;GBP2;GBP3;GFI1;GJA4;GJA5;GJA8;GJB3;GJB5;GCLM;GLUL;GNAI3;GNAT2;GNG5;SFN;GPR3;GPR25;GPX7;GRIK3;GSTM1;GSTM2;GSTM3;GSTM4;GSTM5;GTF2B;GUCA2A;GUCA2B;HCRTR1;HDAC1;HDGF;CFH;CFHR1;CFHR2;MR1;HMGN2;HMGCL;HMGCS2;HPCA;HSD3B1;HSD3B2;HSPA6;HSPA7;IGSF3;HSPG2;HTR1D;HTR6;ID3;IFI16;CYR61;IL6R;IL12RB2;ILF2;INPP5B;INSRR;IPP;IVL;JAK1;JUN;KCNA2;KCNA3;KCNA10;KCNC4;KCND3;KCNJ9;KCNJ10;KCNN3;LAD1;LAMC1;LAMC2;STMN1;LCK;LEPR;LMNA;LMX1A;LOR;LY9;TACSTD2;MAGOH;MATN1;MCL1;SMCP;MEF2D;MFAP2;MGST3;MNDA;MOV10;MPL;MPZ;MSH4;MTF1;MTX1;MUC1;MUTYH;MYCL;MYOC;NASP;NBL1;NCF2;NDUFS2;NDUFS5;NFIA;NFYC;NGF;NHLH1;NHLH2;NIT1;NOTCH2;NPR1;NRAS;NRDC;YBX1;NTRK1;ROR1;DDR2;OPRD1;ORC1;OVGP1;PAFAH2;PRDX1;PAX7;PBX1;PDC;PDE4B;PDZK1;PFDN2;PGM1;PIGC;PI4KB;PIN1P1;PKLR;PKP1;PLA2G2A;PLA2G4A;PLA2G5;PRRX1;POU2F1;POU3F1;PPOX;PPP1R8;PPT1;PRCC;PRKAA2;PRKAB2;PRKACB;PKN2;PSMA5;PSMB2;PSMB4;PSMD4;PTAFR;PTGER3;PTGFR;PTGFRN;PTGS2;QSOX1;PTPRC;PTPRF;PEX19;ABCD3;RAB3B;RAB13;RABGGTB;RAP1A;RAP1GAP;RBBP4;RFX5;RGS1;RGS2;RGS4;RGS13;RGS16;RHCE;RHD;RIT1;RLF;RNASEL;RNF2;RNPEP;RNU1-4;SNORA73A;SNORD21;RORC;RPA2;RPE65;RPL5;RPL11;RPS6KA1;RPS8;RPS27;RXRG;S100A1;SORT1;S100A2;S100A3;S100A4;S100A5;S100A6;S100A7;S100A8;S100A9;S100A10;S100A11;S100A12;S100A13;SARS;SCP2;XCL1;SDHB;SDHC;SELE;SELL;SELP;SFPQ;SRSF4;SHC1;ST3GAL3;STIL;SLAMF1;SLC1A7;SLC2A1;SLC6A9;SLC9A1;SLC16A1;SOAT1;UAP1;SPRR1A;SPRR1B;SPRR2A;SPRR2B;SPRR2C;SPRR2D;SPRR2E;SPRR2F;SPRR2G;SPRR3;SPTA1;TROVE2;SSR2;STXBP3;XCL2;SYCP1;TAF12;TAF13;TAL1;TBX15;TCEA3;TCEB3;VPS72;TGFBR3;THBS3;TCHH;TIE1;TNNI1;TNNT2;TNR;TOP1P1;TPM3;TPR;CCT3;TSHB;TTC4;TUFT1;TNFSF4;UCK2;UQCRH;UROD;USF1;USP1;VCAM1;WNT2B;ZSCAN20;SLC30A2;LUZP1;DNALI1;LRP8;LAPTM5;BSND;CSDE1;EVI5;DAP3;NPHS2;PTP4A2;ARID1A;CDC7;HIST2H2AA3;HIST2H2AC;HIST2H2BE;HIST2H4A;PIP5K1A;TAGLN2;BCAR3;ANXA9;SNHG3;NR0B2;RAD54L;TTF2;RGS5;PIK3R3;ITGA10;LMO4;FCN3;BLZF1;CDC14A;YARS;MKNK1;AKR7A2;PLPP3;RTCA;PTCH2;ALDH4A1;EIF3I;EIF4G3;VAMP4;PEA15;B4GALT3;B4GALT2;B3GALT2;ADAM15;PABPC4;FPGT;PEX11B;CREG1;CD84;FUBP1;EIF2B3;BCL10;SELENBP1;TNFSF18;MPZL1;SH2D2A;ARTN;MAP3K6;ANGPTL1;DIRAS3;TBX19;FCGR2C;SLC16A4;PRPF3;KCNQ4;ARHGEF2;DEDD;ZMYM4;ZMYM6;XPR1;GPR37L1;GPR52;SRSF11;ZFYVE9;CD101;SEP15;ZRANB2;SNRNP40;ARHGAP29;AIM2;RASAL2;THEMIS2;ADAMTS4;TMEM59;SEC22B;CHD1L;PRDX6;CLCA3P;CLCA2;HS2ST1;PDE4DIP;IPO13;SDC3;SLC25A44;KIAA0040;KDM4A;CROCC;PUM1;NOS1AP;RIMS3;EFCAB14;ARHGEF11;DNAJC6;CEP350;LRIG2;SETDB1;SMG7;PLPPR4;UBAP2L;SV2A;DENND4B;RABGAP1L;FAM20B;ZBTB40;KIF14;RBM8A;THRAP3;NR1I3;INSL5;PIGK;SCAMP3;PTPRU;ARPC5;TSPAN2;TSPAN1;CELA3A;PDZK1IP1;WASF2;PATJ;PRG4;GPA33;STX6;HNRNPR;SRRM1;CNKSR1;SF3B4;ZMPSTE24;BCAS2;AKR1A1;WARS2;CEPT1;PIAS3;TESK2;TIMM17A;PPIE;VAV3;HAX1;PPIH;C1orf61;CAP1;LRRC41;SEMA6C;LAMTOR5;SLC19A2;IFI44;POLR3C;IVNS1ABP;TXNIP;PMVK;KHDRBS1;GMEB1;FAM189B;AP4B1;NUDC;PHTF1;NES;AHCYL1;SRSF10;CFHR4;CFHR3;WDR3;OCLM;JTB;MTMR11;MAN1A2;SF3A3;MLLT11;IFI44L;EBNA1BP2;SLC27A3;KIF2C;TDRKH;DNAJB4;ADAM30;RCAN3;FAF1;CD160;GLMN;HHLA3;CELF3;MST1P2;DDX20;MST1L;PADI2;PMF1;DUSP12;VPS45;LYPLA2;CLCA4;MTF2;DNAJC8;NTNG1;FOXJ3;KIAA0907;WDR47;KIFAP3;ATF6;SCMH1;AKR7A3;TTC39A;KDM1A;USP33;WDTC1;KIF21B;NMNAT2;EMC1;POGZ;COLGALT2;MAST2;NCDN;CLCC1;SLC35D1;RGL1;SYT11;PRR[illegible]
	Amplification	8q21.13	chr8:79694982-81000091	6.94E-08	5.63E-05	66.41791045	TPD52;HEY1;MRPS28;PAG1;ZBTB10;ZNF704;MIR5708;LOC101927040;LINC01607
	Amplification	20q13.31	chr20:56932752-58743773	5.37E-14	8.20E-06	64.55223881	BMP7;PCK1;RAE1;STX16;VAPB;SPO11;PPP4R1L;RBM38;PMEPA1;RAB22A;LOC79160;NPEPL1;ZBP1;C20orf85;CTCFL;ANKRD60;APCDD1L-AS1;APCDD1L;MIR4325;MTRNR2L3;STX16-NPEPL1;MIR4532;BMP7-AS1;NKILA

	Deletion	1p36.13	chr1:7843171-24748876	2.91E-22	4.09E-05	64.55223881	ALPL;RERE;C1QA;C1QB;C1QC;CA6;CAPZB;CASP9;TNFRSF8;CDA;CDC42;CLCN6;CLCNKA;CLCNKB;CNR2;CORT;DDOST;DFFA;E2F2;ECE1;EPHA2;ENO1;EPHA8;EPHB2;MTOR;FUCA1;GALE;HMGCL;HSPG2;HTR1D;HTR6;ID3;TNFRSF9;MFAP2;MTHFR;NBL1;NPPA;NPPB;PAX7;PEX14;PGD;PIK3CD;PLA2G2A;PLA2G5;PLOD1;EXOSC10;RAP1GAP;RNU1-4;RPL11;RSC1A1;SDHB;SLC2A5;SRM;TCEA3;TCEB3;TNFRSF1B;ZBTB17;LUZP1;PRDM2;AKR7A2;ALDH4A1;EIF4G3;DHRS3;H6PD;CROCC;ZBTB40;MFN2;CELA3A;ANGPTL7;HNRNPR;SRRM1;UBE4B;MAD2L2;PDPN;MASP2;SRSF10;UTS2;RCAN3;MST1P2;MST1L;PADI2;LYPLA2;PARK7;CTRC;CLSTN1;AKR7A3;SPEN;KDM1A;EMC1;KIF1B;PLEKHM2;OTUD3;KAZN;DNAJC16;UBR4;ATP13A2;TARDBP;CELA3B;PADI4;SZRD1;FBXO2;FBXO6;PLA2G2D;RNU6-1;RNU5E-1;RNU1-3;RNU1-2;RNU1-1;HSPB7;LINC00339;UBIAD1;PADI1;PLA2G2E;SLC45A1;HP1BP3;CELA2B;MRTO4;PADI3;ERRFI1;WNT4;FBXO42;RNF186;FBLIM1;PQLC2;CASZ1;TMEM51;ARHGEF10L;VPS13D;CAMK2N1;ASAP3;PNRC2;NBPF1;NECAP2;RCC2;CTNNBIP1;AGTRAP;PITHD1;NIPAL3;DISP3;KIF17;GRHL3;IL22RA1;MIIP;CELA2A;PLA2G2F;NMNAT1;PINK1;PRAMEF1;PRAMEF2;EFHD2;RSG1;MUL1;AGMAT;GPR157;SPSB1;ZNF436;TAS1R2;ACTL8;USP48;NBPF3;SLC25A33;DDI2;LZIC;CROCCP2;IGSF21;KIAA2013;STPG1;C1orf158;FBXO44;CROCCP3;FHAD1;RBP7;LRRC38;AADACL3;IFFO2;MYOM3;KLHDC7A;VWA5B1;UBXN10;ARHGEF19;C1orf127;ZNF436-AS1;C1orf64;SLC2A7;IFNLR1;FAM43B;TMEM201;TMEM51-AS1;AKR7L;TMCO4;MDS2;C1orf167;LOC284632;SLC25A34;ESPNP;LINC01141;AADACL4;PRAMEF5;HNRNPCL1;PRAMEF10;FAM131C;PADI6;DRAXIN;SPATA21;CENPS;TMEM82;PRAMEF12;PRAMEF8;PRAMEF18;PRAMEF17;PLA2G2C;PRAMEF4;PRAMEF13;SH2D5;NCMAP;LDLRAD2;MIR34A;PRAMEF11;PRAMEF6;HNRNPCL2;UQCRHL;MINOS1;PRAMEF7;PRAMEF25;RPS14P3;PIK3CD-AS1;PRAMEF26;PRAMEF33P;PRAMEF19;PRAMEF20;LACTBL1;PRAMEF34P;HNRNPCL3;PRAMEF22;PRAMEF15;SNORA59A;C1orf195;C1orf234;PRAMEF14;FAM231A;FAM231C;FLJ37453;LOC100132147;FAM231B;MIR1256;MIR1290;NPPA-AS1;MIR3115;MIR4253;MIR1273D;MIR3675;ENO1-AS1;LOC100506022;LOC100506730;LOC100506801;TCEB3-AS1;LOC100506985;APITD1-CORT;MINOS1-NBL1;MIR4695;MIR4419A;MIR3972;MIR4684;MIR5096;MIR4418;MIR4632;MIR378F;RCAN3AS;MIR5697;PINK1-AS;MTOR-AS1;LINC01355;HNRNPCL4;LOC101927876;LOC101927895;UBXN10-AS1;LOC101928043;LOC101928163;PIK3CD-AS2;LOC101929181;PRAMEF27;RNU6-7;RNU6-8;MIR6084;MIR6728;MIR7846;MIR6127;MIR6730;MIR6729;LOC102724539;LOC102724552;LOC102724571;LOC102724659;RNU6-2;LOC105376736;LOC105376805;LOC105378614;MIR34AHG;SNORD128
	Amplification	20q13.2	chr20:53545859-53898125	3.48E-22	2.20E-15	64.17910448	ZNF217;SUMO1P1;LOC101927770;LOC105372672
	Deletion	8p21.3	chr8:22266105-25843110	6.74E-06	9.60E-05	62.68656716	EGR3;GNRH1;LOXL2;NEFM;NEFL;NKX3-1;PPP3CC;STC1;ADAM7;TNFRSF10D;TNFRSF10C;TNFRSF10B;TNFRSF10A;ENTPD4;SORBS3;ADAM28;RHOBTB2;SLC39A14;ADAMDEC1;SLC25A37;KCTD9;PIWIL2;BIN3;CCAR2;PDLIM2;DOCK5;BIN3-IT1;CHMP7;NKX2-
	Deletion	1p36.32	chr1:1-48183817	2.91E-22	8.93E-11	61.56716418	hsa-mir-551a;AK2;ALPL;RERE;ATP6V0B;ADGRB2;BMP8B;C1QA;C1QB;C1QC;CA6;CAPZB;CASP9;RUNX3;TNFRSF8;CDA;CDK11B;CDC20;CDC42;CD52;RCC1;CLCN6;CLCNKA;CLCNKB;PLK3;CNR2;COL8A2;COL9A2;COL16A1;CORT;CSF3R;CTPS1;CYP4A11;CYP4B1;DDOST;DFFA;DFFB;DPH2;DVL1;E2F2;ECE1;EDN2;PHC2;MEGF6;EPHA2;ENO1;EPB41;EPHA8;EPHB2;EXTL1;EYA3;FAAH;FABP3;FGR;FHL3;FOXE3;FOXD2;MTOR;FUCA1;IFI6;GABRD;GALE;GJA4;GJB3;GJB5;GNB1;SFN;GPR3;GRIK3;GUCA2A;GUCA2B;HCRTR1;HDAC1;ZBTB48;HMGN2;HMGCL;HPCA;HSPG2;HTR1D;HTR6;ID3;TNFRSF9;INPP5B;IPP;STMN1;LCK;MATN1;MFAP2;MPL;MTF1;MTHFR;MUTYH;MYCL;NASP;NBL1;NDUFS5;NFYC;NPPA;NPPB;YBX1;OPRD1;PAFAH2;PRDX1;PAX7;PEX10;PEX14;PGD;PIK3CD;PLA2G2A;PLA2G5;PLOD1;EXOSC10;POU3F1;PPP1R8;PPT1;PRKCZ;PSMB2;PTAFR;PTPRF;RAP1GAP;RBBP4;RHCE;RHD;RLF;RNU1-4;SNORA73A;RPA2;RPL11;RPL22;RPS6KA1;RPS8;RSC1A1;SCNN1D;SDHB;SFPQ;SRSF4;ST3GAL3;STIL;SKI;SLC2A1;SLC2A5;SLC6A9;SLC9A1;SRM;TAF12;TAL1;TCEA3;TCEB3;TIE1;TNFRSF1B;TP73;TNFRSF4;UQCRH;UROD;ZSCAN20;ZBTB17;SLC30A2;LUZP1;PRDM2;DNALI1;LAPTM5;PTP4A2;ARID1A;SNHG3;NR0B2;RAD54L;PIK3R3;MMP23B;MMP23A;KCNAB2;FCN3;YARS;MKNK1;AKR7A2;PTCH2;ALDH4A1;EIF3I;EIF4G3;B4GALT2;TNFRSF25;PABPC4;TNFRSF14;TNFRSF18;PER3;EIF2B3;ARTN;MAP3K6;KCNQ4;ZMYM4;ZMYM6;DHRS3;VAMP3;SNRNP40;THEMIS2;H6PD;ISG15;PLCH2;IPO13;SDC3;KDM4A;CROCC;PUM1;CEP104;RIMS3;EFCAB14;KLHL21;SLC35E2;ZBTB40;MFN2;THRAP3;PTPRU;TSPAN1;CELA3A;PDZK1IP1;WASF2;ANGPTL7;HNRNPR;SRRM1;CNKSR1;ZMPSTE24;UBE4B;AKR1A1;TESK2;PPIE;MAD2L2;PPIH;CAP1;LRRC41;PDPN;KHDRBS1;GMEB1;NUDC;MASP2;SRSF10;UTS2;SF3A3;EBNA1BP2;KIF2C;RER1;RCAN3;MST1P2;MST1L;PADI2;LYPLA2;PARK7;CTRC;ACOT7;DNAJC8;CLSTN1;FOXJ3;SCMH1;AKR7A3;SPEN;KDM1A;WDTC1;EMC1;KIF1B;MAST2;NCDN;PLEKHM2;OTUD3;KAZN;CAMTA1;SZT2;DNAJC16;UBR4;ATP13A2;TARDBP;CELA3B;ICMT;MACF1;PADI4;TMEM50A;KPNA6;STX12;CLIC4;SYF2;MMACHC;CHD5;SZRD1;LDLRAP1;NOC2L;FBXO2;FBXO6;PLA2G2D;MYCBP;HEYL;AGO1;SNORA73B;SNORD55;RNU11;RNU6-1;RNU5F-1;RNU5E-1;RNU5D-1;RNU1-3;RNU1-2;RNU1-1;TRAPPC3;HSPB7;ARHGEF16;AHDC1;TEKT2;SMPDL3B;LINC00339;SSU72;GNL2;UBIAD1;PADI1;PLA2G2E;WRAP73;SLC45A1;HP1BP3;CELA2B;ZNF593;ZNF691;MECR;UTP11;SDF4;MRTO4;TMEM69;HPCAL4;YTHDF2;ZCCHC17;PADI3;CMPK1;ERRFI1;WNT4;FBXO42;RNF186;MXRA8;HES2;GPN2;FBLIM1;MED18;TRIT1;PQLC2;CASZ1;RHBDL2;ADPRHL2;TRNAU1AP;C1orf109;CPSF3L;C1orf159;AURKAIP1;MRPL20;AIM1L;TMEM51;BSDC1;XKR8;TMEM39B;CDCA8;ARHGEF10L;RNF220;VPS13D;EVA1B;ATAD3A;TMEM57;TRIM62;PANK4;CAMK2N1;ASAP3;POMGNT1;PNRC2;PIGV;NBPF1;MAP7D1;NECAP2;IQCC;DNAJC11;RCC2;DMAP1;AJAP1;TMEM234;MTFR1L;CTNNBIP1;RSRP1;AGTRAP;PITHD1;MAN1C1;NIPAL3;SEPN1;TP73-AS1;PLEKHG5;LRRC47;DISP3;KIF17;ZSWIM5;KIAA1522;HES4;GRHL3;DLGAP3;IL22RA1;HIVEP3;GPBP1L1;MIIP;CELA2A;GPATCH3;CLSPN;PRDM16;OXCT2;RRAGC;TINAGL1;P3H1;PLA2G2F;SMAP2;ATPAF1;S100PBP;MEAF6;EXO5;CEP85;NMNAT1;ELOVL1;VWA1;MRPS15;PINK1;MARCKSL1;PRAMEF1;PRAMEF2;NADK;ZFP69B;PHACTR4;AUNIP;ERI3;C1orf50;CCDC28B;EFHD2;MMEL1;RSG1;OR4F5;NKAIN1;MUL1;TMEM53;AKIRIN1;HECTD3;YRDC;NOL9;PPCS;LIN28A;SH3D21;SNIP1;AGMAT;ZMYM1;LINC00115;MORN1;FAM110D;KIAA0319L;DHDDS;GPR157;ZC3H12A;SPSB1;CPTP;ZNF436;TAS1R2;TAS1R1;GJA9;OR4F16;SYNC;ACTL8;TSSK3;CCNL2;HYI;SH3BGRL3;SESN2;ESPN;TAS1R3;ATAD3B;STK40;TMEM222;PLEKHN1;USP48;ZMYND12;NBPF3;ZDHHC18;SLC25A33;DDI2;LZIC;NT5C1A;TRIM63;FAM167B;FOXD2-AS1;PERM1;CROCCP2;HPDL;MFSD2A;SYTL1;IGSF21;LSM10;C1orf94;SNHG12;CFAP74;KIAA2013;THAP3;STPG1;SPOCD1;UBXN11;C1orf158;FBXO44;ATPIF1;SNORD46;SNORD38A;SNORD38B;MED8;SMIM12;AZIN2;TMEM54;TOE1;ERMAP;CSMD2;CROCCP3;FHAD1;LOC115110;RAB42;FAM46B;RBP7;ACAP3;UBE2J2;CCDC163;KDF1;LRRC38;AADACL3;PUSL1;B3GALT6;IFFO2;TPRG1L;FAM213B;MYOM3;DMBX1;TMCO2;ZNF684;GJB4;HMGB4;RNF19B;A3GALT2;C1orf122;OSCP1;C1orf216;KLHDC7A;VWA5B1;UBXN10;KLF17;TMEM125;ARHGEF19;ACTRT2;MIB2;C1orf127;SAMD11;LOC148413;PHF13;LINC00337;CCDC27;ZNF436-AS1;KNCN;MOB3C;DCDC2B;ZNF362;LINC01225;MANEAL;PDIK1L;CLDN19;CFAP57;C1orf210;CCDC24;BTBD19;CCDC17;C1orf64;SLC2A7;CALML6;IFNLR1;CYP4Z2P;CITED4;FAM43B;PAQR7;OXCT2P1;AGO3;AGO4;FAM76A;TMEM201;CYP4Z1;FAAP20;TXLNA;SLFNL1;TMEM51-
	Deletion	8p23.2	chr8:727306-6404670	2.75E-09	2.75E-09	61.19402985	CLN8;MYOM2;DLGAP2;ARHGEF10;KBTBD11;CSMD1;LOC286083;LOC401442;ERICH1-AS1;MIR596;MIR3674;DLGAP2-AS1;LOC101927752;LOC101927815;LOC101928058;MIR7160;KBTBD11-OT1
	Amplification	11q13.3	chr11:69479372-69558043	7.36E-101	1.60E-85	58.95522388	LINC01488
	Amplification	17q23.1	chr17:60002610-60190275	1.92E-43	1.89E-21	57.8358209	CA4;HEATR6;USP32;WFDC21P;TBC1D3P1-DHX40P1;LOC653653;MIR4737;LOC105371849
	Amplification	17q23.2	chr17:61786255-61969051	1.45E-31	0.00014378	57.08955224	MED13;INTS2;BRIP1
	Amplification	17q23.2	chr17:63029011-63287980	3.65E-31	7.33E-07	57.08955224	TANC2
	Amplification	17q24.1	chr17:64547174-64707563	9.14E-27	1.26E-06	55.97014925	SMURF2
	Amplification	17q24.2	chr17:68062806-68316096	1.27E-26	2.30E-11	55.97014925	SLC16A6;ARSG;AMZ2;LOC440461;LINC00674
	Amplification	17q24.2	chr17:66281489-66648212	3.92E-24	2.70E-05	52.98507463	PRKCA;PRKCA-AS1
	Amplification	17q22	chr17:58296399-58593606	3.48E-22	0.00023984	51.86567164	SEPT4;SUPT4H1;MTMR4;TSPOAP1;RNF43;TEX14;HSF5;C17orf47;MIR142;TSPOAP1-AS1;MIR4736;SEPT4-AS1
	Amplification	17q21.33	chr17:50752375-50926363	1.40E-25	4.36E-10	49.62686567	TOB1;LUC7L3;LINC00483;WFIKKN2;TOB1-AS1;MIR8059
	Amplification	8p11.23	chr8:37541239-37744699	7.62E-54	4.76E-43	48.88059701	ERLIN2;ZNF703;LOC101929622;LOC102723701

C3	Deletion	1p21.3	chr1:75199278-119766351	0.00056603	0.00056603	48.88059701	ABCA4;ACADM;ADORA3;AGL;ALX3;AMPD1;AMPD2;AMY1A;AMY1B;AMY1C;AMY2A;AMY2B;RHOC;ATP1A1;ATP5F1;BRDT;CAPZA1;CASQ2;CD2;CD53;CD58;CHI3L2;CLCA1;CNN3;COL11A1;CSF1;CTBS;DBT;DLSTP1;DPYD;DR1;S1PR1;CELSR2;EXTL2;F3;GBP1;GBP2;GBP3;GFI1;GCLM;GNAI3;GNAT2;GNG5;GSTM1;GSTM2;GSTM3;GSTM4;GSTM5;GTF2B;HSD3B1;HSD3B2;IGSF3;CYR61;KCNA2;KCNA3;KCNA10;KCNC4;KCND3;MOV10;MSH4;NGF;NHLH2;NRAS;OVGP1;PRKACB;PKN2;PSMA5;PTGFR;PTGFRN;ABCD3;RABGGTB;RAP1A;SNORD21;RPL5;SORT1;SARS;SLC16A1;STXBP3;SYCP1;TAF13;TBX15;TGFBR3;TSHB;VCAM1;WNT2B;CSDE1;EVI5;CDC7;BCAR3;TTF2;LMO4;CDC14A;RTCA;FUBP1;BCL10;SLC16A4;CD101;SEP15;ARHGAP29;CLCA3P;CLCA2;HS2ST1;LRIG2;PLPPR4;PIGK;TSPAN2;BCAS2;WARS2;CEPT1;VAV3;LAMTOR5;IFI44;AP4B1;PHTF1;AHCYL1;WDR3;MAN1A2;IFI44L;DNAJB4;GLMN;DDX20;CLCA4;MTF2;NTNG1;WDR47;USP33;CLCC1;ADGRL2;KIAA1107;SLC35A3;LRRC8B;LPAR3;DDAH1;RWDD3;ZZZ3;PTPN22;PHGDH;AK5;SNORA66;SNORD45B;SNORD45A;CHIA;GPSM2;SLC25A24;DNTTIP2;TMED5;SH3GLB1;HAO2;SNX7;TRIM33;DPH5;GPR88;TRMT13;RSBN1;ZNHIT6;GIPC2;GDAP2;FAM46C;PALMD;FNBP1L;ST7L;PRPF38B;LRRC8D;PRMT6;MCOLN3;SLC22A15;RNPC3;LRIF1;CTTNBP2NL;FAM212B;KYAT3;TMEM167B;OLFML3;TMIGD3;AMIGO1;ODF2L;KIAA1324;PTBP2;DNASE2B;ADGRL4;MFSD14A;RBM15;DCLRE1B;WDR77;EPS8L3;VTCN1;TTLL7;RPAP2;DENND2D;RPF1;SIKE1;TRIM45;VANGL1;ST6GALNAC5;GPR61;SYDE2;ZNF644;LRRC8C;PROK1;PSRC1;ATP1A1-AS1;STRIP1;ZNF697;NEXN;DNAJA1P5;HENMT1;GBP4;GBP5;SSX2IP;OLFM3;WDR63;MAB21L3;SLC44A3;ATXN7L2;C1orf194;ASB17;LRRC39;HSD3BP4;DRAM2;PIFO;C1orf162;SYT6;SAMD13;C1orf52;TMEM56;NBPF4;SLC30A7;MGC27382;CHIAP2;DENND2C;GBP6;PLPPR5;FNDC7;SASS6;HFM1;UBL4B;ALG14;SPAG17;HIPK1;SLC44A5;AKR7A2P1;EPHX4;AKNAD1;MCOLN2;COL24A1;ST6GALNAC3;MAGI3;FAM19A3;FAM102B;SYPL2;CYB561D1;ZNF326;BTBD8;PPM1J;LINC01140;CCDC18;MYBPHL;BARHL2;MIGA1;NEXN-AS1;GBP7;C1orf146;FAM69A;SLC6A17;C1orf137;UOX;FRRS1;GBP1P1;FLJ27354;MIR137HG;MIR137;MIR197;LINC01555;FLJ31662;LOC440600;LOC440602;BCL2L15;PGCP1;GEMIN8P4;RBMXL1;SPATA42;CYMP;LOC643355;LOC643441;LINC00622;LOC646626;SETSIP;ACTG1P4;NBPF6;SCARNA2;SNORD45C;MIR553;LOC729930;LOC729970;LOC729987;MIR942;MIR760;LINC01349;LOC100129046;LOC100129138;LINC01160;LOC100129620;CCDC18-AS1;AP4B1-AS1;MIR320B1;MIR548N;MIR4256;SPATA1;LINC01364;RTCA-AS1;FAM212B-AS1;SLC16A1-AS1;TMEM56-RWDD3;MIR378G;MIR548AC;MIR2682;MIR4423;DPYD-AS1;DPYD-AS2;VAV3-AS1;KCNC4-AS1;KCND3-AS1;HAO2-IT1;KCND3-IT1;LOC100996251;LOC100996263;LOC100996630;LOC100996635;LINC01356;LAMTOR5-AS1;LOC101927342;LOC101927412;LOC101927434;LINC01361;LOC101927560;LOC101927587;LOC101927844;PKN2-AS1;LINC01057;LOC101928098;LOC101928118;LOC101928241;LOC101928270;LOC101928370;LOC101928436;LOC101928476;LOC101928718;HIPK1-AS1;LOC101928977;LOC101928995;LOC101929023;LOC101929099;LOC101929147;MIR7852;MIR7856;MIR7156;LOC102606465;LOC102723661;LINC01461;LINC01397;LINC01307;LINC01525;WARS2-IT1;LOC105378828;LOC105378853;LOC105378933
	Amplification	17q12	chr17:39677348-39749916	2.93E-75	1.61E-73	48.13432836	ERBB2;GRB7;MIEN1;PGAP3;MIR4728
	Amplification	17q21.33	chr17:49405569-49412397	3.03E-31	3.69E-20	47.76119403	PHB
	Deletion	9p21.3	chr9:21865499-22448738	7.94E-09	1.54E-08	47.76119403	CDKN2A;CDKN2B;CDKN2A-AS1;CDKN2B-AS1
	Amplification	17q22	chr17:54986383-55117223	4.23E-14	9.19E-07	46.26865672	STXBP4
	Deletion	9p23	chr9:7799607-10619051	0.00040455	0.00054386	46.26865672	PTPRD;PTPRD-AS1;LOC105375972
	Amplification	11q13.4	chr11:71148609-71278299	1.42E-37	6.46E-07	45.89552239	SHANK2
	Deletion	6q16.1	chr6:83198789-104729175	6.11E-08	0.00096875	44.02985075	hsa-mir-2113;CCNC;CGA;CNR1;EPHA7;GABRR1;GABRR2;GRIK2;HTR1E;ME1;NT5E;POU3F2;SIM1;MAP3K7;RNGTT;TBX18;FHL5;SNAP91;CASP8AP2;SYNCRIP;SLC35A1;FUT9;PNRC1;ASCC3;CEP162;ANKRD6;ZNF292;MDN1;UFL1;ORC3;PNISR;FBXL4;SNORD50A;NDUFAF4;CYB5R4;UBE2J1;COQ3;AKIRIN2;RARS2;SMIM8;LYRM2;SNX14;RRAGD;PRDM13;BACH2;LINC01590;MANEA;GPR63;SPACA1;MCHR2;FAXC;GJA10;USP45;MRAP2;KLHL32;RIPPLY2;PM20D2;SRSF12;CFAP206;PRSS35;C6orf163;MMS22L;GJB7;SNHG5;TSG1;SNORD50B;MCHR2-AS1;TSTD3;MIR2113;MIR4464;MIR4643;MIR548AI;MANEA-AS1;LOC101927314;LOC101927365;LOC101928820;LOC101928911;LOC101928936;LOC101929057;CASC6;TBX18-AS1;LOC105377879;LINC01611
	Amplification	11q13.2	chr11:67188527-67236826	4.08E-27	1.05E-11	43.28358209	KDM2A
	Amplification	8p11.22	chr8:39349882-39611299	2.25E-25	1.86E-07	42.53731343	ADAM3A;ADAM18;ADAM5;LOC100130964
	Deletion	6q21	chr6:102069225-116250412	5.87E-07	0.00094435	41.79104478	AIM1;AMD1;PRDM1;COL10A1;FOXO3;FRK;FYN;GPR6;HDAC2;LAMA4;MARCKS;PREP;REV3L;SMPD2;NR2E1;DDO;SNX3;CD164;WISP3;WASF1;ATG5;ZBTB24;FIG4;TRAF3IP2;BVES;SEC63;CDK19;SESN1;OSTM1;TUBE1;C6orf203;CDC40;SOBP;QRSL1;PDSS2;HACE1;BEND3;POPDC3;MICAL1;ARMC2;RPF2;RTN4IP1;SLC22A16;MFSD4B;GTF3C6;SLC16A10;BVES-AS1;CCDC162P;AK9;NT5DC1;HS3ST5;LACE1;SCML4;CEP57L1;PPIL6;LINC01268;FLJ34503;LINC00222;LIN28B;GSTM2P1;RFPL4B;FAM229B;TRAF3IP2-AS1;MIR587;TPI1P3;METTL24;LIN28B-AS1;CD24;LOC100422737;LOC100996634;LOC101927640;LOC101927686;LOC101927768;ARMC2-AS1;MIR7641-2;LOC105377924;LOC105377962
	Amplification	17q11.2	chr17:29026052-29097297	4.15E-22	8.38E-17	41.41791045	TIAF1;PIPOX;MYO18A
	Amplification	8p11.21	chr8:41192085-42131660	9.75E-12	0.00012472	41.41791045	ANK1;SFRP1;KAT6A;GOLGA7;GINS4;GPAT4;NKX6-3;MIR486-1;MIR548AO;MIR486-2;LOC102723729;LOC105379393
	Deletion	19p13.3	chr19:1-2427969	5.37E-07	5.37E-07	41.04477612	AMH;ATP5D;AZU1;HCN2;BSG;CDC34;CIRBP;CNN2;CSNK1G2;CFD;ARID3A;EFNA2;ELANE;GAMT;MKNK2;GPX4;GZMM;OAZ1;PALM;POLR2E;POLRMT;PRTN3;PTBP1;RPS15;STK11;TCF3;MADCAM1;SF3A2;PLPP2;AP3D1;MED16;FSTL3;APC2;ABCA7;UQCR11;SBNO2;ARHGAP45;SHC2;TIMM13;DAZAP1;RNU6-1;FGF22;THEG;LSM7;MBD3;MIER2;PCSK4;C19orf24;PLEKHJ1;BTBD2;RNF126;SPPL2B;WDR18;REXO1;PLPPR3;OR4F17;ABHD17A;KLF16;DOT1L;KISS1R;MUM1;MIDN;R3HDM4;TMEM259;TPGS1;REEP6;IZUMO4;SCAMP4;ADAT3;GRIN3B;JSRP1;MOB3A;MISP;PLK5;C2CD4C;CIRBP-AS1;C19orf25;ATP8B3;CBARP;CSNK1G2-AS1;ODF3L2;ADAMTSL5;TMPRSS9;NDUFS7;C19orf35;WASH5P;ONECUT3;MEX3D;LINC01002;PRSS57;LINGO3;FAM138A;FAM138C;LOC100288123;MIR1909;MIR1302-2;MIR1227;MIR1302-9;MIR1302-10;MIR3187;MIR1302-11;MIR4321;MIR4745;RNU6-7;MIR6789;RNU6-2
	Deletion	17q21.31	chr17:40160020-47459478	1.52E-05	0.00013187	40.67164179	ACLY;AOC2;ARL4D;ATP6V0A1;BRCA1;FMNL1;CDC6;CDC27;CCR7;CNP;CRHR1;DHX8;DUSP3;ETV4;EZH1;GAST;FZD2;G6PC;KAT2A;GFAP;CCR10;GRN;HCRT;HSD17B1;IFI35;IGFBP4;ITGA2B;ITGB3;JUP;KRT9;KRT10;KRT12;KRT13;KRT14;KRT15;KRT16;KRT17;KRT19;KRT31;KRT32;KRT33A;KRT33B;KRT34;KRT35;NBR1;MAPT;ADAM11;MEOX1;MPP2;MPP3;MYL4;NAGLU;NMT1;NSF;PPY;PYY;RAB5C;RARA;RNU2-1;RPL27;SLC4A1;SMARCE1;STAT3;STAT5A;STAT5B;MLX;TOP2A;DNAJC7;TUBG1;UBTF;WNT3;WNT9B;RND2;CNTNAP1;AOC3;BECN1;KRT38;KRT37;KRT36;HAP1;MAP3K14;EFTUD2;GOSR2;PLEKHM1;LRRC37A;HDAC5;GJC1;PSME3;EIF1;NBR2;RAMP2;VAT1;P3H4;HEXIM1;C1QL1;RUNDC3A;GPATCH8;KCNH4;FAM215A;KRT23;TUBG2;NKIRAS2;COA3;PSMC3IP;SOST;RAPGEFL1;ARL17A;SLC25A39;HIGD1B;KRT20;LRRC37A4P;KLHL11;ATXN7L3;FKBP10;WNK4;C17orf53;TMUB2;DHX58;ACBD4;DCAKD;PLEKHH3;DBF4B;COASY;AARSD1;KRTAP1-3;KRTAP1-1;KRTAP9-9;KRTAP4-6;KRTAP2-1;TTC25;KRTAP4-12;KRTAP1-5;KRTAP3-1;KRTAP3-2;KRTAP9-2;KRTAP9-3;KRTAP9-8;KRTAP17-1;VPS25;TMEM101;GHDC;KRTAP4-4;TNS4;KRTAP9-4;KRTAP4-1;KRTAP4-5;KRTAP4-3;KRTAP4-2;KRTAP3-3;KRTAP2-4;AOC4P;G6PC3;ASB16;HSPB9;PLCD3;TMEM106A;NT5C3B;SPATA32;HEXIM2;LSM12;CCDC43;CNTD1;EFCAB13;GJD3;KRT222;KRT40;CD300LG;KIF18B;RUNDC1;CRHR1-IT1;LINC00974;WIPF2;KRT25;TMEM99;NAGS;FAM134C;SPPL2C;KRT28;KRT24;ARHGAP27;ZNF385C;STH;KANSL1;C17orf105;FAM171A2;MEIOC;KRT42P;PTRF;KLHL10;ASB16-AS1;KRT27;KRT26;LINC00671;CCDC103;RPRML;KRT39;LRRC37A2;LOC644172;KANSL1-AS1;KRTAP4-11;KRTAP4-8;KRTAP1-4;KRTAP2-2;KRTAP9-1;NSFP1;KRTAP2-3;MAPT-AS1;MAPT-IT1;LINC00910;KRTAP4-9;KRTAP4-7;MAP3K14-AS1;RAMP2-AS1;MIR2117;MIR4315-2;MIR4315-1;KRTAP9-7;KRTAP16-1;LOC100505782;ARL17B;KRTAP9-6;KRTAP29-1;MIR548AT;MIR5010;MIR5089;LINC00854;PTGES3L;PTGES3L-AARSD1;RUNDC3A-AS1;LINC01180;RARA-AS1;LOC101929767;MIR6781;MIR6782;MIR6784;MIR6867;MIR6780A;MIR6510;MIR6783;MIR6866;THCAT158;CRHR1-IT1-CRHR1;LOC105371789;LOC105371795;LOC107546764

	Deletion	6q27	chr6:155944963-170805979	2.54E-06	1.30E-05	39.17910448	ACAT2;CCR6;GPR31;IGF2R;KIF25;LPA;MAS1;MAP3K4;AFDN;PARK2;PDCD2;PLG;PSMB1;RPS6KA2;SLC22A1;SLC22A3;SLC22A2;SOD2;T;TBP;TCP1;TCP10;TCTE3;DYNLT1;THBS2;EZR;RNASET2;SYNJ2;QKI;WTAP;PDE10A;FGFR1OP;LINC01558;DLL1;MRPL18;SNX9;MPC1;UNC93A;PHF10;ERMARD;AGPAT4;TULP4;ARID1B;TMEM181;SMOC2;ZDHHC14;FRMD1;AGPAT4-IT1;LINC00574;LPAL2;RSPH3;TTLL2;FAM120B;FNDC1;SERAC1;LINC00473;SYTL3;SFT2D1;TAGAP;PACRG;PNLDC1;LOC154449;DACT2;C6orf118;OSTCP1;WDR27;PACRG-AS1;PRR18;LOC285804;C6orf120;DKFZp451B082;TCP10L2;LOC401286;LINC00242;LINC01624;GTF2H5;LINC00602;LOC441178;AFDN-AS1;SNORA20;SNORA29;TMEM242;LOC729603;PACRG-AS3;HGC6.3;LOC100129518;C6orf99;LOC100131532;AIRN;LOC100289495;MIR1913;MIR1202;MIR3918;MIR3939;MIR3692;KIF25-AS1;CAHM;MIR4466;MIR4644;RPS6KA2-AS1;SYNJ2-IT1;RPS6KA2-IT1;EZR-AS1;LOC101929122;PACRG-AS2;LOC101929297;LOC101929420;LOC101929460;LINC01615;LOC101929504;LOC101929523;MIR7161;MIR7641-2;LOC102724053;LOC102724152;LOC102724357;LOC102724511;LOC105378098;MEAT6;LOC105378123;LOC105378127;LOC105378137;LOC105378146
	Deletion	10q26.11	chr10:102850166-133797422	0.00037653	0.00054386	38.43283582	ACADSB;ADAM8;ADD3;ADRA2A;ADRB1;BNIP3;CASP7;COL17A1;CTBP2;CYP2E1;DMBT1;DOCK1;DUSP5;ECHS1;EMX2;FGFR2;GFRA1;PRLHR;GPR26;GRK5;HABP2;HMX2;INPP5A;ABLIM1;MGMT;MKI67;MXI1;NRAP;OAT;PNLIP;PNLIPRP1;PNLIPRP2;HTRA1;PTPRE;RGS10;RPL21;SLC18A2;TAF5;TCF7L2;TECTB;TIAL1;UROS;XPNPEP1;SHOC2;ADAM12;UTF1;EIF3A;INA;SMC3;NEURL1;BUB3;GSTO1;BAG3;SH3PXD2A;FAM53B;SLK;DCLRE1A;SMNDC1;GLRX3;DPYSL4;TACC2;TUBGCP2;PRDX3;VAX1;ATE1;SEC23IP;RAB11FIP2;INPP5F;NT5C2;PDCD11;SORCS3;FAM175B;ATRNL1;EDRF1;PDCD4;VENTX;CUZD1;CALY;CALHM2;CHST15;ACSL5;ZRANB1;CFAP46;NSMCE4A;CNNM2;CCDC186;WDR11;DHX32;PPP2R2D;TDRD1;BCCIP;AS3MT;GPAM;SHTN1;FAM160B1;PLEKHA1;FAM204A;LHPP;IKZF5;ZDHHC6;MCMBP;PLEKHS1;OBFC1;C10orf88;CFAP43;LRRC27;PCGF6;ADGRA1;NKX6-2;AFAP1L2;USMG5;KNDC1;ITPRIP;MTG1;BBIP1;FANK1;SYCE1;SORCS1;PRAP1;ZNF511;C10orf90;BTBD16;FAM24A;PSTK;MMP21;PDZD8;BORCS7;GSTO2;SFR1;CALHM3;CTAGE7P;CLRN3;PNLIPRP3;SFXN4;CPXM2;TRUB1;VTI1A;C10orf82;CACUL1;CFAP58;C10orf91;PWWP2B;EMX2OS;PLPP4;PAOX;FAM24B;EBF3;CALHM1;CASC2;TCERG1L;HSPA12A;FUOM;JAKMIP3;STK32C;RBM20;PDCD4-AS1;LOC283038;WDR11-AS1;KCNK18;VWA2;NANOS1;HMX3;NHLRC2;CCDC172;DMBT1P1;ENO4;ARMS2;TEX36;LINC00959;GUCY2GP;NKX1-2;C10orf120;LOC399815;METTL10;FLJ37035;FOXI2;LINC01164;LINC01168;SPRNP1;LINC01561;FAM45A;FRG2B;SPRN;MIR202;NPS;SCART1;SNORA19;FAM196A;RPL13AP6;MIR609;RPEL1;MIR936;ADGRA1-AS1;ATE1-AS1;TEX36-AS1;DUX4;MIR1307;MIR2110;MIR548E;MIR378C;MIR4297;MIR4295;MIR4296;MIR3941;MIR3663;MIR3944;SH3PXD2A-AS1;CFAP58-AS1;SORCS3-AS1;ADD3-AS1;LINC00867;BORCS7-ASMT;FAM24B-CUZD1;MIR4680;MIR4483;MIR4682;MIR4482;MIR4484;MIR4681;LINC00601;FANK1-AS1;LINC01163;LOC101927472;TCERG1L-AS1;LOC101927523;LOC101927549;LINC01166;MIR202HG;LOC101927692;MIR3663HG;FAM53B-AS1;EDRF1-AS1;MIR6715B;MIR6715A;NEURL1-AS1;LOC102724589;LOC103344931;LINC01435;LINC01167;LOC105378470;AS-PTPRE;SNORA87
	Deletion	17q12	chr17:32993209-35745222	1.18E-07	1.33E-06	38.43283582	ASIC2;AP2B1;LIG3;PEX12;RAD51D;CCL1;CCL2;CCL7;CCL8;CCL11;CCL13;CCT6B;NLE1;FNDC8;SLFN12;ZNF830;SLFN11;RASL10B;RFFL;TMEM132E;SLFN13;SLC35G3;UNC45B;SLFN5;SLFN14;C17orf102;SNORD7;AA06;SLFN12L;RAD51L3-RFFL;LOC101927239;LOC105371743;LOC107985033
	Amplification	12q15	chr12:68341568-70909804	7.10E-07	7.10E-07	38.05970149	CPM;LYZ;MDM2;CNOT2;PTPRB;PTPRR;RAP1B;YEATS4;CCT2;FRS2;CPSF6;KCNMB4;SLC35E3;NUP107;RAB3IP;BEST3;MYRFL;LRRC10;LOC100130075;MIR1279;SNORA70G;MIR3913-2;MIR3913-1;LOC100507195;LOC100507250;LOC101928002;LINC01481
	Amplification	17q11.2	chr17:27340388-27587283	4.00E-12	0.00022049	38.05970149	KSR1;TBC1D3P5
	Amplification	11q14.1	chr11:77511647-78267349	2.01E-32	8.37E-21	35.82089552	CLNS1A;NDUFC2;THRSP;GAB2;AAMDC;RSF1;USP35;KCTD14;ALG8;INTS4;AQP11;KCTD21;LOC646029;KCTD21-AS1;NDUFC2-KCTD14
	Deletion	11p15.5	chr11:1-549958	8.39E-06	8.39E-06	35.07462687	HRAS;PSMD13;RNH1;IFITM1;IFITM3;IFITM2;PKP3;SIRT3;BET1L;SIGIRR;RIC8A;PGGHG;PTDSS2;ODF3;SCGB1C1;NLRP6;ANO9;B4GALNT4;IFITM5;LINC01001;MIR6743
	Deletion	15q13.1	chr15:1-34756318	1.38E-05	1.30E-05	35.07462687	APBA2;NBEAP1;CHRM5;CHRNA7;GABRA5;GABRB3;GABRG3;IPW;TRPM1;NDN;OCA2;RYR3;SCG5;SNRPN;TJP1;UBE3A;MKRN3;PWAR5;HERC2;SNURF;ARHGAP11A;SLC12A6;FAN1;GOLGA8A;CYFIP1;FAM189A1;NPAP1;DKFZP434L187;GREM1;EMC4;KLF13;MAGEL2;MTMR10;NOP10;NSMCE3;EMC7;AVEN;ATP10A;GJD2;KATNBL1;NIPA2;CHRFAM7A;ULK4P3;ULK4P1;ARHGAP11B;SNORD107;TUBGCP5;NIPA1;PWAR1;OTUD7A;PGBD4;LPCAT4;NUTM1;LOC283683;GOLGA6L2;OR4N4;LOC283710;HERC2P3;GOLGA6L1;GOLGA8G;GOLGA8IP;SNORD108;SNORD109A;SNORD115-1;WHAMMP3;FMN1;SNORD64;PWAR4;PWARSN;LINC01193;IGHV1OR15-1;GOLGA8EP;OR4M2;OR4N3P;HERC2P10;HERC2P2;MIR211;NF1P2;GOLGA6L22;HERC2P9;WHAMMP2;GOLGA8B;LINC00929;GOLGA8N;CHEK2P2;LOC646214;CXADRP2;PDCD6IPP2;IGHV1OR15-3;REREP3;GOLGA8S;GOLGA8J;GOLGA8T;GOLGA8K;GOLGA8M;GOLGA6L6;LOC727924;GOLGA8O;GOLGA6L7P;GOLGA8H;GOLGA8CP;PWRN1;PWRN2;SNORD116-1;SNORD116-2;SNORD116-3;SNORD116-4;SNORD116-5;SNORD116-8;SNORD116-10;SNORD116-11;SNORD116-12;SNORD116-13;SNORD116-14;SNORD116-15;SNORD116-16;SNORD116-17;SNORD116-18;SNORD116-20;SNORD116-21;SNORD116-22;SNORD116-23;SNORD116-24;SNORD116-25;SNORD115-2;SNORD116-26;SNORD116-27;SNORD115-3;SNORD115-4;SNORD115-5;SNORD115-6;SNORD115-7;SNORD115-8;SNORD115-10;SNORD115-11;SNORD115-12;SNORD115-13;SNORD115-14;SNORD115-15;SNORD115-16;SNORD115-17;SNORD115-18;SNORD115-20;SNORD115-21;SNORD115-22;SNORD115-23;SNORD115-25;SNORD115-26;SNORD115-29;SNORD115-30;SNORD115-31;SNORD115-32;SNORD115-33;SNORD115-34;SNORD115-35;SNORD115-36;SNORD115-37;SNORD115-38;SNORD115-39;SNORD115-41;SNORD115-44;SNORD116-28;SNORD116-29;SNORD115-48;SNORD115-24;SNORD115-27;SNORD115-28;SNORD115-45;SNORD115-47;LOC100128714;LOC100130111;LOC100131315;HERC2P7;GOLGA8F;GOLGA8DP;POTEB2;WHAMMP1;LOC100288637;LOC100289656;MIR1233-1;MIR1268A;MIR3118-3;MIR1233-2;MIR3118-4;MIR3118-2;MIR4509-1;MIR4509-2;MIR4508;MIR4715;TMCO5B;MIR5701-2;MIR5701-1;SNORD116-30;SNORD115-
	Amplification	17q12	chr17:36700861-36726776	6.48E-09	3.59E-05	34.70149254	[MRM1]
	Amplification	14q21.1	chr14:37269223-37975587	0.00012125	0.00095318	33.58208955	FOXA1;MIPOL1;TTC6;LINC00517
	Deletion	2q37.3	chr2:218928767-242193529	0.00051098	0.00051098	31.71641791	hsa-mir-1244-1;AGXT;ALPI;ALPP;ALPPL2;KIF1A;BOK;CHRND;CHRNG;COL4A3;COL4A4;COL6A3;CRYBA2;DES;DTYMK;EPHA4;ACSL3;GBX2;GPC1;GPR35;HDLBP;AGFG1;DNAJB2;HTR2B;SP110;IHH;INHA;INPP5D;IRS1;KCNJ13;NCL;NDUFA10;SEPT2;NEU2;NPPC;PAX3;PDCD1;PDE6D;SERPINE2;PPP1R7;PSMD1;PTMA;PTPRN;SNORD20;SAG;CCL20;SLC4A3;SP100;SPP2;TUBA4A;SCG2;CUL3;DGKD;STK16;PER2;CDK5R2;LRRFIP1;GPR55;TRIP12;ECEL1;EIF4E2;HDAC4;FARP2;FARSB;ABCB6;ARL4C;RAMP1;SPEG;NMUR1;STK25;COPS8;CAPN10;SP140;PASK;ATG4B;OBSL1;DNPEP;SH3BP4;KCNE4;NGEF;SNORD82;SNED1;GIGYF2;TRAF3IP1;CNPPD1;GMPPA;ANO7;PRLH;THAP4;ANKMY1;SCLY;ASB1;CAB39;UGT1A10;UGT1A8;UGT1A7;UGT1A6;UGT1A5;UGT1A9;UGT1A4;UGT1A1;UGT1A3;FEV;PID1;ATG16L1;ANKZF1;USP40;HJURP;HES6;ASIC4;DOCK10;C2orf83;MFF;ACKR3;RNPEPL1;WDFY1;NYAP2;GAL3ST2;RAB17;COPS7B;MRPL44;TRPM8;ATG9A;MLPH;FAM134A;GLB1L;CHPF;IQCA1;NHEJ1;FAM124B;TM4SF20;C2orf54;TUBA4B;ARMC9;EFHD1;SPHKAP;SLC19A3;ILKAP;ITM2C;RHBDD1;ING5;MGC16025;DNER;B3GNT7;SP140L;LOC93463;STK11IP;MOGAT1;AGAP1;TWIST2;DIS3L2;NEU4;AP1S3;SGPP2;SPATA3;TMEM198;ZFAND2B;FBXO36;MTERF4;UBE2F;OTOS;COPS9;OR6B3;LOC150935;LINC01107;LOC151174;ERFE;CCDC140;SLC23A3;LINC00608;SLC16A14;LOC151475;LINC00471;LOC151484;MSL3P1;DAW1;C2orf57;TIGD1;LOC200772;CFAP65;C2orf72;RTP5;LOC285095;DUSP28;MROH2A;ESPNL;ECEL1P2;SPATA3-AS1;RBM44;AQP12A;KLHL30;RESP18;C2orf82;OR6B2;ASB18;FLJ43879;MIR149;MIR153-1;DNAJB3;LOC440934;MIR375;LOC643387;LOC646736;PRSS56;AQP12B;SNORA75;LOC654841;SCARNA6;SCARNA5;MIR562;D2HGDH;LOC728323;LOC100129175;PP14571;LOC100286922;MIR1471;MIR1244-1;BOK-AS1;MIR1244-3;MIR1244-2;MIR3133;MIR3131;MIR4268;MIR3132;MIR4269;AGAP1-IT1;UBE2F-SCLY;MIR4439;MIR4777;MIR2467;MIR4440;MIR4786;MIR4441;MIR5001;MIR5702;MIR5703;LOC100996693;CAPN10-AS1;LINC01237;LOC101928111;LOC101928881;MIR6811;LOC102723927;LINC01173
	Amplification	11q13.4	chr11:72648832-73662506	7.09E-13	0.00016311	30.59701493	P2RY2;P2RY6;PDE2A;ARHGEF17;FCHSD2;STARD10;FAM168A;PLEKHB1;RELT;ATG16L2;ARAP1;MIR4459;MIR4692
	Amplification	17q11.2	chr17:32857192-32907515	2.33E-05	0.00035329	29.10447761	MYO1D

	Deletion	21q11.2	chr21:1-44254691	4.01E-05	4.09E-05	29.10447761	APP;ATP5J;ATP5O;BACH1;BAGE;TMEM50B;RUNX1;CBR1;CBR3;CBS;CRYAA;CSTB;CXADR;DSCAM;RCAN1;DYRK1A;ERG;ETS2;GABPA;GART;GRIK1;HLCS;HMGN1;IFNAR1;IFNAR2;IFNGR2;IL10RB;KCNE1;KCNJ6;KCNJ15;MX1;MX2;NCAM2;NDUFV3;PCP4;PDE9A;PKNOX1;TMPRSS15;PWP2;SH3BGR;ITSN1;SIM2;SLC5A3;SOD1;SON;HSPA13;TFF1;TFF2;TFF3;TIAM1;TRAPPC10;TMPRSS2;TPTE;TTC3;U2AF1;WRB;NRIP1;CHAF1B;C21orf33;PDXK;RRP1;PSMG1;SYNJ1;CLDN8;ADAMTS1;ABCG1;URB1;CRYZL1;DOPEY2;KCNE2;RWDD2B;OLIG2;DSCR4;DSCR3;B3GALT5;USP16;CCT8;WDR4;BTG3;HSF2BP;ADAMTS5;RRP1B;ICOSLG;MORC3;CLDN14;BACE2;C2CD2;LTN1;CLDN17;N6AMT1;USP25;DONSON;HUNK;ZBTB21;PIGP;UBASH3A;RIPPLY3;BRWD1;SLC37A1;RBM11;CYP4F29P;LINC00160;SMIM11A;C21orf62-AS1;MIS18A;LINC00158;CHODL-AS1;LINC00113;LINC00112;LINC00111;SETD4;FAM3B;RIPK4;CLIC6;LINC00308;MRPL39;C21orf91;DNAJC28;C21orf62;MRAP;C21orf59;AGPAT3;MAP3K7CL;SCAF4;JAM2;EVA1C;PRDM15;SAMSN1;TMPRSS3;MRPS6;RUNX1-IT1;DSCR8;URB1-AS1;BAGE4;BAGE3;BAGE2;RSPH1;UMODL1;ERVH48-1;PAXBP1;LINC00310;LINC00313;B3GALT5-AS1;MIR155HG;CYYR1;OLIG1;LINC00161;KRTAP13-1;TCP10L;CHODL;ANKRD30BP2;LIPI;ABCC13;LOC150051;LCA5L;IGSF5;SIK1;LINC00479;ZNF295-AS1;UMODL1-AS1;PLAC4;LINC00189;C21orf91-OT1;LINC00314;KRTAP15-1;DSCR9;DSCR10;D21S2088E;LINC00307;LINC00515;LOC284825;KRTAP13-4;LINC00323;LINC00319;AATBC;POTED;KRTAP7-1;KRTAP8-1;KRTAP11-1;KRTAP19-1;KRTAP13-2;KRTAP13-3;KRTAP23-1;KRTAP6-1;KRTAP6-2;KRTAP6-3;KRTAP19-2;KRTAP19-3;KRTAP19-4;KRTAP19-5;KRTAP19-6;KRTAP19-7;KRTAP20-1;KRTAP20-2;KRTAP21-1;KRTAP21-2;KRTAP22-1;KRTAP20-3;LOC339622;LINC00317;LINC00320;LOC388813;MIR99AHG;KRTAP26-1;ANKRD20A11P;LINC00114;LOC400867;MIRLET7C;MIR125B2;MIR155;MIR99A;MGC39584;GRIK1-AS1;KRTAP24-1;KRTAP27-1;SNORA80A;MIR663A;KRTAP19-8;LINC01548;MIR802;RNA5-8S5;RNA18S5;RNA28S5;LINC00322;KRTAP25-1;TEKT4P2;LOC100133286;KRTAP20-4;KRTAP22-2;KRTAP21-3;IL10RB-AS1;GRIK1-AS2;MIR4327;MIR548X;MIR3118-1;MIR3156-3;MIR3197;MIR3687-1;MIR3648-1;LINC01549;PAXBP1-AS1;LINC00649;LINC01426;LOC100506403;CBR3-AS1;DSCAM-AS1;LOC100507412;LINC00159;MIR4760;MIR4759;MIR5692B;RNA45S5;TTC3-AS1;BRWD1-AS1;SAMSN1-AS1;BACH1-IT2;DSCAM-IT1;CYYR1-AS1;LINC01436;MIR548XHG;LINC01425;LOC101927843;LOC101927869;LOC101927973;LOC101928107;LOC101928126;C21orf140;LOC101928233;LOC101928269;LOC101928311;LOC101928398;TCONS_00029157;LOC101928435;LOC101930094;LINC00945;MIR6070;MIR6501;MIR6724-1;MIR6814;MIR8069-1;MIR6130;MIR6508;LOC102723360;KCNE1B;SMIM11B;LOC102724188;LOC102724219;LOC102724354;LOC102724428;LOC102724502;U2AF1L5;LOC102724652;LINC01423;LOC102724843;BRWD1-IT2;MIR6724-2;MIR6724-3;LOC105372751;LOC105372795;LOC105372833;LOC105379511;LOC105379514;FRGCA;SNORA91;WRB-SH3BGR
	Deletion	4q35.2	chr4:173038024-190214555	2.24E-05	2.24E-05	25.74626866	AGA;SLC25A4;CASP3;DCTD;F11;ACSL1;FAT1;FRG1;GPM6A;HMGB2;HPGD;ING2;IRF2;KLKB1;MTNR1A;TLR3;VEGFC;GLRA3;SORBS2;SAP30;HAND2;ADAM29;SCRG1;FAM149A;FBXO8;PDLIM3;GALNT7;CLDN22;NEIL3;UFSP2;CDKN2AIP;TENM3;LRP2BP;STOX2;CFAP97;SPCS3;TRAPPC11;CENPU;HAND2-AS1;WWC2;CEP44;SNX25;LOC90768;WDR17;ZFP42;SPATA4;ENPP6;ASB5;WWC2-AS2;RWDD4;PRIMPOL;TRIML2;CCDC110;CYP4V2;F11-AS1;LINC01098;LOC339975;TRIML1;ANKRD37;LOC389247;HELT;LINC01060;FAM92A1P2;C4orf47;FRG2;SLED1;FLJ38576;LINC00290;LOC728175;MIR3945HG;CLDN24;DUX4;MIR1305;DBET;MIR548T;MIR4276;MIR3945;LINC01093;LOC100506272;MIR4455;LOC101928509;LOC101928551;LOC101928590;LINC01099;WWC2-AS1;LINC01262;LOC101930370;LOC102723766;LVCAT8;LOC105377590;LINC01596
	Deletion	8p11.21	chr8:43028712-46549322	3.89E-05	5.99E-05	24.25373134	FNTA;POMK;HGSNAT;POTEA
	Amplification	6q21	chr6:106039258-107707637	0.00026767	0.00026767	15.29850746	AIM1;PRDM1;ATG5;C6orf203;SOBP;QRSL1;PDSS2;BEND3;RTN4IP1;SCML4;MIR587;CD24;LOC100422737;LOC105377924

* notes: Q-values and residual q-values refer to GISTIC2 results. The last column list the genes located in each region of loss or gain. We kept the regions with residual q-value < 0.01.
