# Supplementary material for: DNA methylation profiling deciphers three EMT subtypes with distinct prognoses and therapeutic vulnerabilities in breast cancer
Source: J Cancer. 2024 Jul 16;15(15):4922–38. doi: 10.7150/jca.96096 (PMC11310866; doi:10.7150/jca.96096)
Supplement: Supplementary file 1 — Supplementary methods, figures and tables. [file jcav15p4922s1.zip › Supplemental Methods.doc]

**Supplementary Methods**

**Cohort enrollment and data preprocessing**

DNA methylation data were from promoter regions, which were defined as the genomic transcription initiation sites within 2000 bp upstream and 500 bp downstream. Filtering criteria were employed to remove probes lacking CpG context, those with known SNPs in proximity, those displaying multiple genomic alignments, and those mapping to the X and Y chromosomes via the *ChAMP* package. Furthermore, DNA methylation sites with more than 10% missing data were discarded, and missing values were imputed through the K-Nearest Neighbor (KNN) algorithm. Batch effects between HM27K and HM450K DNA methylation data were counteracted using *limma* package. We averaged beta values of multiple probes per gene to represent its methylation level[1].

Level 4 RNA sequencing data (raw count) in the TCGA-BRCA dataset were acquired from GDC Data Portal via *TCGAbiolinks* package. Raw count values were converted to transcripts per million kilobases (TPM) values and subsequently log2-transformed to enhance comparability with normalized matrices generated from microarrays. Normalized sequencing data and matched clinical information in the Sweden Cancerome Analysis Network - Breast (SCAN-B) clinical trial (GSE96058) were extracted from GEO databases. Notably, duplicate samples were eliminated. We downloaded z-score normalized mRNA profiles of the METABRIC cohort and corresponding clinical data from cBioPortal.

The microarray datasets generated on the Affymetrix, Illumina, and Agilent platforms underwent distinct preprocessing steps. For microarray data derived by Affymetrix, background correction followed by quantile normalization and logarithmic transformation was mainly performed via *affy* package[2]. For Illumina-generated raw data, *lumi* package was employed for preprocessing and normalization[3], whereas microarray data from Agilent were executed through *limma* package[4]. To consolidate the gene expression values represented by multiple probe sets, we adopted a strategy based on the maximum expression level observed across probes in a dataset.

**Derivation of EMT classifier**

In our effort to enhance the universality of EMT subtypes, we developed a reliable EMT gene panel classifier by utilizing a novel computational pipeline based on gene expression profiles.

**Step1: Consensus differential analysis**

Genes shared among multiple datasets were regarded as input features of ML. Subsequently, we performed differential analysis via three different algorithms: Significance Analysis of Microarrays (SAM), resampling-based multiple hypothesis testing (multtest), and Linear Models for Microarray data (limma). These three algorithms were implemented with the *samr[5]*, *multtest[6]*, and *limma[4]* packages, respectively. Features meeting filtering criteria from the aforementioned algorithms were designated as “leaf genes” for future ML framework: 1. limma (|log2FC| >1, corrected *p*-value <0.001) 2. SAM (nPerm =1000, corrected *p*-value <0.001) 3. multtest (nPerm =1000, corrected *p*-value <0.001). Benjamini–Hochberg (BH) method was employed to correct the *p*-value. In total, the numbers of subtype leaf genes are 300 (C1), 666 (C2), and 86 (C3), separately (**Table S5**).

**Step2: 1000 logistic regression to evaluate leaf genes**

Bootstrapping methodology was further conducted to evaluate these leaf genes (from the prior step). We randomly sampled 80% of the samples from the entire dataset and performed univariate logistic regression on these samples to assess the discriminative power of each leaf gene. In order to mitigate the impact of class imbalance on model training, we implemented the synthetic minority oversampling technique (SMOTE) for imbalance adjustments[7, 8]. This iterative procedure was run 1000 times, and 513 genes that consistently met the criteria (P <0.001 in robustness testing) across all resampling executions were retained for subsequent analysis. Subsequently, the gene expression matrix underwent z-score standardization and served as input for machine learning (ML).

**Step3: Feature selection**

Correlation-based unsupervised feature selection was implemented to accomplish data dimensionality reduction and collinearity elimination. The correlation threshold was defined at 0.8, leading to the retention of only one gene from gene pairs with correlations exceeding this threshold. Through this process, 413 genes were retained.

Moreover, Boruta[9] and support vector machine recursive feature elimination (SVM-RFE)[10] algorithms were employed with default parameters to further reduce dimension, yielding 164 genes. Multinomial sparse group-lasso (MSGL) regression analysis[11] with 10-fold cross-validation to identify the best gene combination. We performed 1000 iterations and selected the optimal λ values concerning the one-standard-error rule in 10-fold cross-validation. The optimal λ value was the maximum value within one standard error of the smallest deviance. A total of 35 gene combinations were screened out. The gene list and corresponding occurring frequency during 1000 iterations are shown in **Table S6**. After 1000 iterations, the 96 gene combination with the highest frequency was determined as the most relevant and feasible EMT subtype special gene panel and was titled subtype stem genes (SSGs) (**Table S7**). Consequently, we utilized these 96 SSGs to build our classifier.

**Step4: Multiple ML to build the classifier**

We utilized five classification algorithms to train classifiers for 96 SSGs and evaluated model performance via 5×10-fold cross-validation. These classifiers included Support Vector Machine (SVM), eXtreme Gradient Boosting (XGBoost), Random Forest (RF), LightGBM and naive Bayes (NB)[12-16]. Each classifier performance was conducted across 20 iterations of random training/testing splits on all samples (**Table S8**). The classifier performance was quantified via balanced accuracy, area under the precision-recall curve (PRAUC), and area under the receiver operating characteristic curve (AUROC). The best model was selected based on the maximum mean value across three evaluation metrics for each model. The *tidymodels* package was used to train the ML classifier[17].

**Genomic alteration characteristics related to EMT subtypes**

For copy number variation (CNV) data obtained from the GDC portal, the TCGA SNP pipeline was applied to filter usual germline CNVs. GISTIC 2.0 was subsequently employed to detect significant CNV events with a confidence level set at 0.99[18]. A computational pipeline was developed to uncover CNV events linked to EMT subtypes. Initially, cytoband regions with residual *q* values less than 0.01 were retained grounded on GISTIC results. Furthermore, Fisher's exact test was implemented to analyze the difference of cytoband amplification/deletion frequencies among subtypes, with cytoband segments exhibiting *p*-values < 0.05 considered as subtype-based CNV events. Following this pipeline, 12 cytoband segments (driver genes) were kept for further analysis.

For SNP mutation annotation format (MAF) files downloaded from the GDC portal, we processed them through *maftools* package[19]. The top 30 genes with somatic SNPs across all samples were selected, and Fisher's exact test was applied to identify mutational genes associated with each EMT subtype, ultimately, we retained 11 mutational genes with *p*-values less than 0.05.

We extracted mutation features as described by Alexandrov et al.[20] from the COSMIC database (http://cancer.sanger.ac.uk/cosmic/signatures). Weight scores for each mutational signature were inferred by setting 6% as the cut-off value in *deconstructSigs* package[21]. In line with previous study, 5 mutational signatures relevant to BC were retained for subsequent analysis. In parallel, the relationship between genomic events (mutation and CNV) in each subtype and mutation signature prevalence was examined as previous investigation reported[22].

**References**

1.	Chaudhary K, Poirion OB, Lu L, Garmire LX. Deep Learning-Based Multi-Omics Integration Robustly Predicts Survival in Liver Cancer. Clin Cancer Res. 2018; 24: 1248-59.

2.	Gautier L, Cope L, Bolstad BM, Irizarry RA. affy--analysis of Affymetrix GeneChip data at the probe level. Bioinformatics. 2004; 20: 307-15.

3.	Du P, Kibbe WA, Lin SM. lumi: a pipeline for processing Illumina microarray. Bioinformatics. 2008; 24: 1547-8.

4.	Ritchie ME, Phipson B, Wu D, Hu Y, Law CW, Shi W, et al. limma powers differential expression analyses for RNA-sequencing and microarray studies. Nucleic Acids Res. 2015; 43: e47.

5.	Tusher VG, Tibshirani R, Chu G. Significance analysis of microarrays applied to the ionizing radiation response. Proc Natl Acad Sci U S A. 2001; 98: 5116-21.

6.	Schadow. Bioinformatics and Computational Biology Solutions Using R and Bioconductor.Edited by Robert Gentleman, Wolfgang Huber, Vincent J. Carey, Rafael A. Irizarry and Sandrine Dudoit. Briefings in Bioinformatics. 2006; 8: 136-7.

7.	Chawla NV, Bowyer KW, Hall LO, Kegelmeyer WP. SMOTE: synthetic minority over-sampling technique. Journal of artificial intelligence research. 2002; 16: 321-57.

8.	Pan X, Chen L, Liu M, Niu Z, Huang T, Cai YD. Identifying Protein Subcellular Locations With Embeddings-Based node2loc. IEEE/ACM Trans Comput Biol Bioinform. 2022; 19: 666-75.

9.	Kursa MB, Jankowski A, Rudnicki WR. Boruta–a system for feature selection. Fundamenta Informaticae. 2010; 101: 271-85.

10.	Huang S, Cai N, Pacheco PP, Narrandes S, Wang Y, Xu W. Applications of Support Vector Machine (SVM) Learning in Cancer Genomics. Cancer Genomics Proteomics. 2018; 15: 41-51.

11.	Vincent M, Hansen NR. Sparse group lasso and high dimensional multinomial classification. Computational Statistics & Data Analysis. 2014; 71: 771-86.

12.	Bhasin M, Raghava GP. ESLpred: SVM-based method for subcellular localization of eukaryotic proteins using dipeptide composition and PSI-BLAST. Nucleic Acids Res. 2004; 32: W414-9.

13.	Li W, Yin Y, Quan X, Zhang H. Gene Expression Value Prediction Based on XGBoost Algorithm. Front Genet. 2019; 10: 1077.

14.	Kouzani AZ. Subcellular localisation of proteins in fluorescent microscope images using a random forest. 2008 IEEE International Joint Conference on Neural Networks (IEEE World Congress on Computational Intelligence): IEEE; 2008. p. 3926-32.

15.	Ke G, Meng Q, Finley T, Wang T, Chen W, Ma W, et al. Lightgbm: A highly efficient gradient boosting decision tree. Advances in neural information processing systems. 2017; 30.

16.	Rish I. An empirical study of the naive Bayes classifier. IJCAI 2001 workshop on empirical methods in artificial intelligence; 2001. p. 41-6.

17.	Wickham MKaH. Tidymodels: a collection of packages for modeling and machine learning using tidyverse principles. 2020.

18.	Mermel CH, Schumacher SE, Hill B, Meyerson ML, Beroukhim R, Getz G. GISTIC2.0 facilitates sensitive and confident localization of the targets of focal somatic copy-number alteration in human cancers. Genome Biol. 2011; 12: R41.

19.	Mayakonda A, Lin DC, Assenov Y, Plass C, Koeffler HP. Maftools: efficient and comprehensive analysis of somatic variants in cancer. Genome Res. 2018; 28: 1747-56.

20.	Alexandrov LB, Jones PH, Wedge DC, Sale JE, Campbell PJ, Nik-Zainal S, et al. Clock-like mutational processes in human somatic cells. Nat Genet. 2015; 47: 1402-7.

21.	Rosenthal R, McGranahan N, Herrero J, Taylor BS, Swanton C. DeconstructSigs: delineating mutational processes in single tumors distinguishes DNA repair deficiencies and patterns of carcinoma evolution. Genome Biol. 2016; 17: 31.

22.	Abbas S, Pich O, Devonshire G, Zamani SA, Katz-Summercorn A, Killcoyne S, et al. Mutational signature dynamics shaping the evolution of oesophageal adenocarcinoma. Nat Commun. 2023; 14: 4239.
